# Supplementary material for: Desymmetric homologating annulation to access chiral pentafulvenes and their application in bioimaging
Source: Nat Commun. 2024 Mar 7;15:2101. doi: 10.1038/s41467-024-45346-4 (PMC10920648; doi:10.1038/s41467-024-45346-4)
Supplement: Supplementary file 1 — SUPPLEMENTARY INFO [file 41467_2024_45346_MOESM1_ESM.pdf]

Supplementary Information

Desymmetric Homologating Annulation to Access Chiral Pentafulvenes and Their Application in Bioimaging

Sanjay Singh[1], Ravi Saini [1], Akshay Joshi[2], Neetu Singh[2] and Ravi P. Singh[1]*

[1]*Department of Chemistry, Indian Institute of Technology, Delhi, Hauz Khas, New Delhi-110016, India*

[2]*Center for Biomedical Engineering, Indian Institute of Technology Delhi, Hauz Khas, New Delhi-110016, India.*

E-mail: *ravips@chemistry.iitd.ac.in*

Table of contents

1	General Information	S2
2	Optimization Studies and scope of reaction using 10 mol% catalyst	S4-S5
3	Reaction monitoring through NMR and ESI-MS analysis	S5- S6
4	Emission Spectra of chiral and racemic adducts with excitation at 360 nm and MTT assay for chiral adducts	S7
5	Experimental Section	S8-S13
6	Characterization of starting material and homologated adducts	S13-S44
7	^{1}H and ^{13}C{^{1}H} spectra of compounds	S46-S107
8	HPLC data of the compounds	S109-S169
9.	Single crystal XRD data of **3a**	S170-S171
10.	Supplementary References	S172

1. General Information

^{1}H and ^{13}C NMR spectra were recorded on a Bruker AV-300 instrument (400/500 MHz and 100/125 MHz, respectively) and internally referenced to Tetramethylsilane signal or residual protonated solvent signals. Data for ^{1}H NMR are reported as follows: chemical shift (δ, ppm), multiplicity (s- singlet; d- doublet; t- triplet; q- quartet; m-multiplet), integration, coupling constant (Hz). Data for ^{13}C NMR are reported in terms of chemical shift (δ, ppm). MS-TOF mass spectrometer and ESI mass spectrometer was used to record low resolution and high-resolution mass spectra. Optical rotations were measured on Rudolph, AUTOPOL II AP Dual Wavelength System polarimeter using 10 mm sample cell, and [α]D values are given in deg. cm.g^{-1}dm^{-1}; concentration c is listed in g. (100 mL)$^{-1}$. Column chromatographic separations were carried out on Merck silica gel (100–200 mesh). High performance liquid chromatography (HPLC) analysis was performed on an Agilent 1220 and Agilent 1260-II Infinity LC instrument equipped with a quaternary pump, using a Chiralpak IA-IE, AD-H, AS-H Column (250x4.6 mm). UV absorption was monitored at 260-265 nm.

2. Optimization Studies:

Supplementary Table 1. Catalyst Screening.

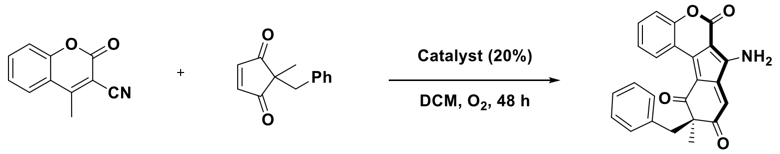

1a 2a 3a

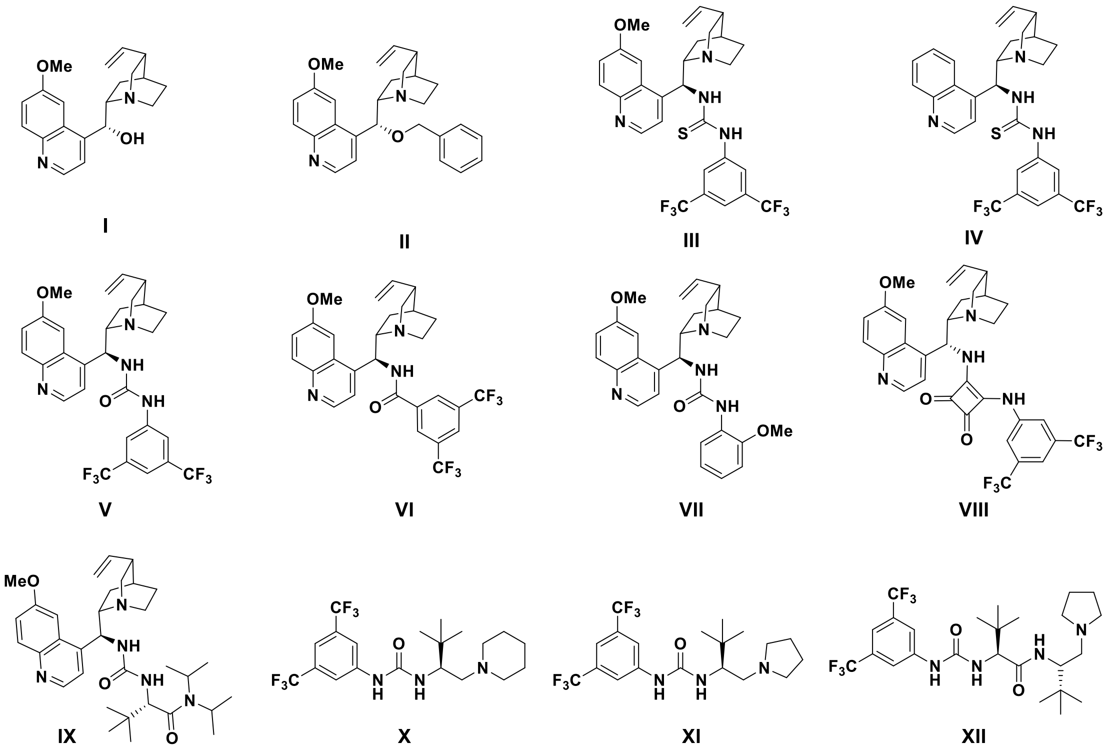

Entry	Catalyst	Solvent	Temp. (°C)	Yield (%)[b]	er[c]
1	**I**	DCM	rt	20	46:54
2	**II**	DCM	rt	84	59:41
3	**III**	DCM	rt	47	81:19
4	**IV**	DCM	rt	36	68:32
5	**V**	DCM	rt	32	78:22
6	**VI**	DCM	rt	Traces	-
7	**VII**	DCM	rt	Traces	-
8	**VIII**	DCM	rt	63	50:50
9	**IX**	DCM	rt	15	56:44
10	**X**	DCM	rt	56	71:29
11	**XI**	DCM	rt	68	69:31
12	**XII**	DCM	rt	55	75:25

[a]unless otherwise noted, reaction condition: **1a** (0.1 mmol), **2a** (0.12 mmol), and **Catalyst** (20 mol%) in DCM 1mL. [b]isolated yield, [c]er calculated by Chiral HPLC.

Supplementary Table 2. Effect of Solvent:

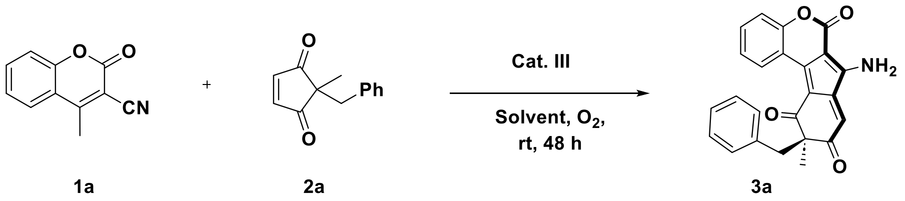

Entry	Solvent	Yield (%)[b]	er[c]
1	DCM	20	46:54
2	$CHCl_3$	68	18:82
3	DCE	32	21:79
4	CCl_4	11	23:77
5	THF	Traces	-
6	EtOAc	NR	-
7	CH_3CN	NR	-
8	Toluene	78	26:74-
9	$PhCF_3$	15	11:89
10	PhCl	32	10:90

[a]unless otherwise noted, reaction condition: **1a** (0.1 mmol), **2a** (0.12 mmol), and **Cat. III** (20 mol%) in 1 mL of solvent. [b]isolated yield, [c]er calculated by Chiral HPLC.

Supplementary Table 3. Oxidant Variation:

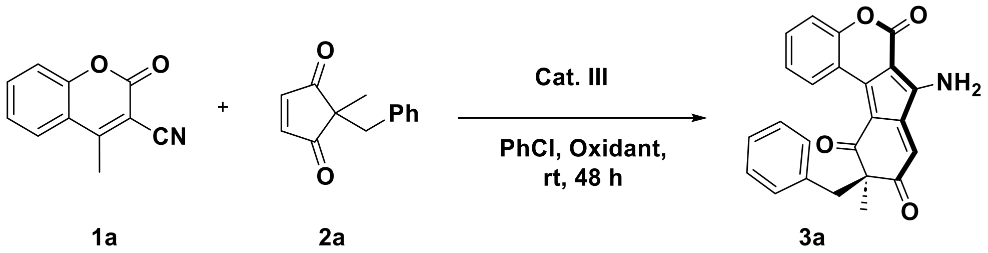

Entry	Oxidant	Yield (%)[b]	er[c]
1	**O_2**	32	90:10
2	**TBHP**	NR	-
3	**H_2O_2**	NR	-
4	**DDQ**	NR	-
5	**$CuBr_2$**	NR	-

[a]unless otherwise noted, reaction condition: **1a** (0.1 mmol), **2a** (0.12 mmol), and **Cat. III** (20 mol%) in mL PhCl. [b]isolated yield, [c]er calculated by Chiral HPLC.

Supplementary Table 4. Scope of reaction at 10 mol% catalyst loading

Additionally, some of the coumarins (**1j**, **1o**, and **1s**) and cyclopent-4-ene-1,3-diketones (**2b**, **2j**, **2n**, and **2p**) substrates were also tested under the slightly modified catalytic system. The homologating annulation reaction with **1j**, **2b,** and **2n** by utilizing 10 mol% of **C6**, at 0 ^{o}C for 3 d, and afforded the corresponding products comparatively in lower yields with similar selectivities. While substrates **1o**, **1s**, **2j**, and **2p** when screened under the same conditions yielded the desired products in similar yields with slightly enhanced selectivities.

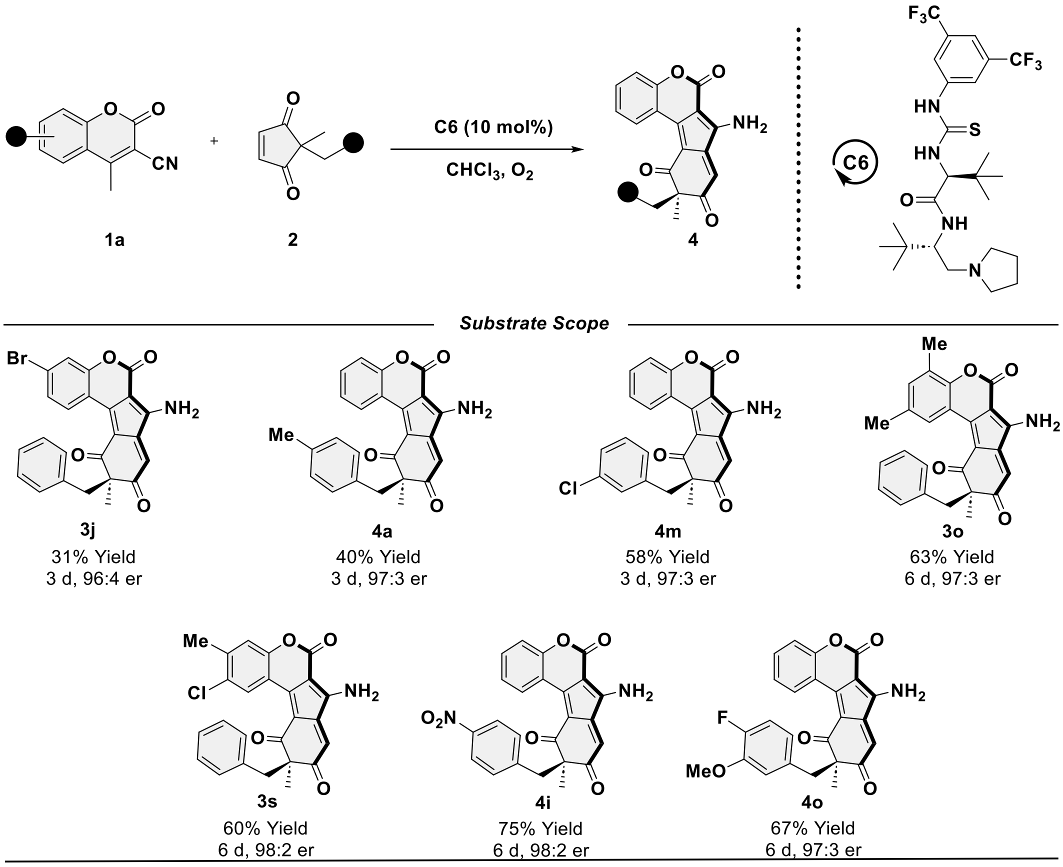

[a]unless otherwise noted, reaction condition: **1** (0.1 mmol), **2** (0.12 mmol), and **Cat. 6** (10 mol%) in 1 mL of solvent. [b]isolated yield, [c]er calculated by Chiral HPLC.

3. Reaction monitoring through ^{1}H NMR and ESI-MS analysis:

To a solution of catalyst **C6** (0.02 mmol, 20 mol %) in $CDCl_3$ (0.5 mL) were added **2a** (0.12 mmol), and **1a** (0. 1 mmol) in an NMR tube. The reaction was monitored for 72 h by recording the NMR after 24 h time intervals. The formation of intermediate **V** is indicated by the emergence of peak at δ = 10.29 ppm after 24 h reaction time (Supplementary Fig. 1A). After 72 h reaction time, the resulting reaction mixture turns deep brown and ^{1}H NMR spectrum recorded at this stage demonstrates no characteristic peak with respect to the desired product

3a formation instead reaction stops proceeding after the formation intermediate **V** (Supplementary Fig. 1B). The ESI-MS of the crude reaction mixture also suggests the formation of intermediate **V** (Supplementary Fig. 1C). Finally, after adding silica gel to the reaction mixture, the deep brown color of the reaction mixture changes to a deep blue which marks the appearance of characteristic peaks of the desired product **3a** at δ = 9.8 ppm (Supplementary Fig. 1B).

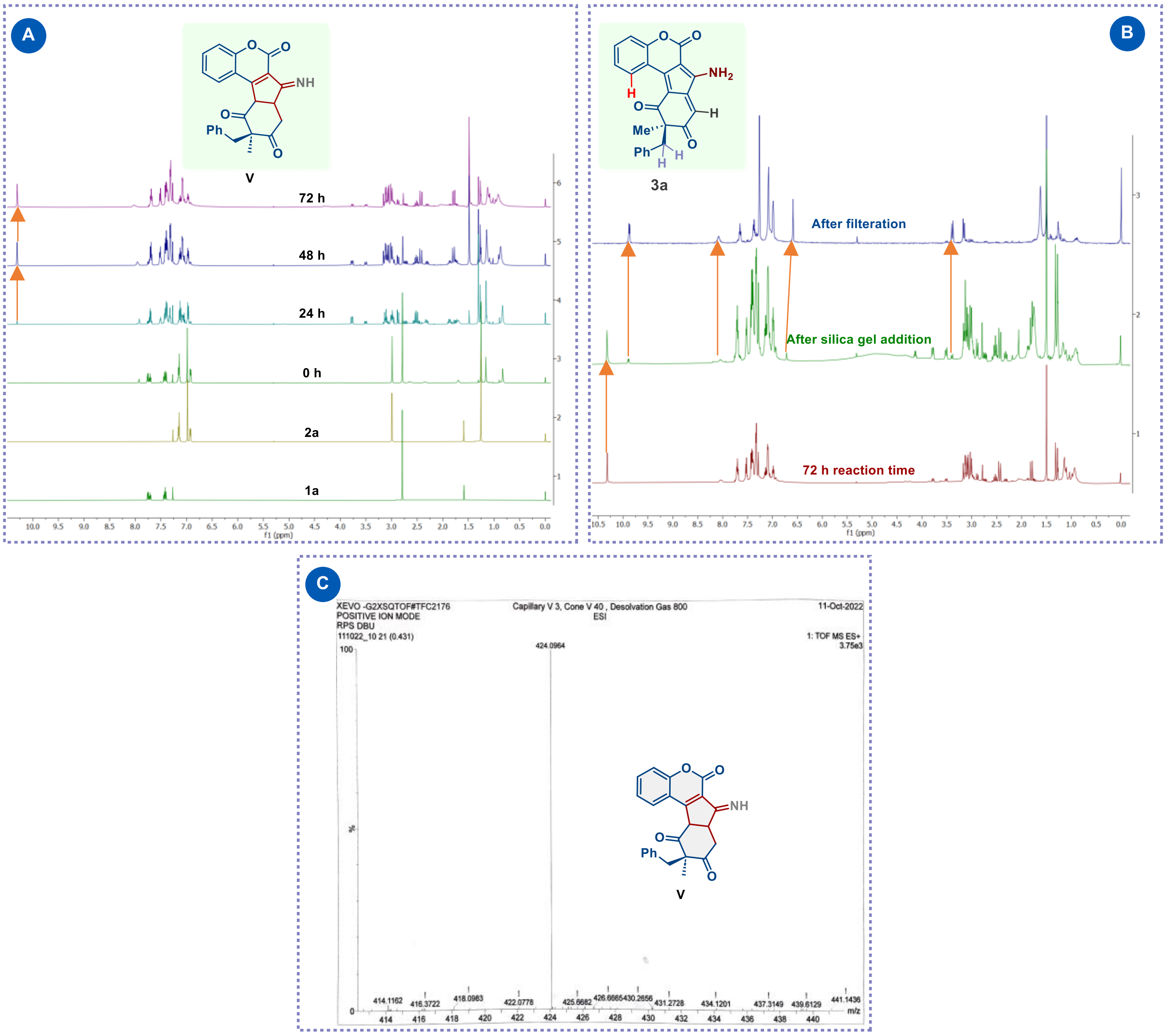

Supplementary Fig. 1. Reaction monitoring through ^{1}H NMR and ESI-MS analysis: A) Real time reaction monitoring through ^{1}H NMR. B) ^{1}H NMR analysis showing silica gel promoted transition of intermediate **V** into **3a**. C) ESI-MS analysis of crude reaction mixture after 72 h.

4. Emission Spectra with excitation at 360 nm and MTT assay

Chiral adducts **Racemic adducts**

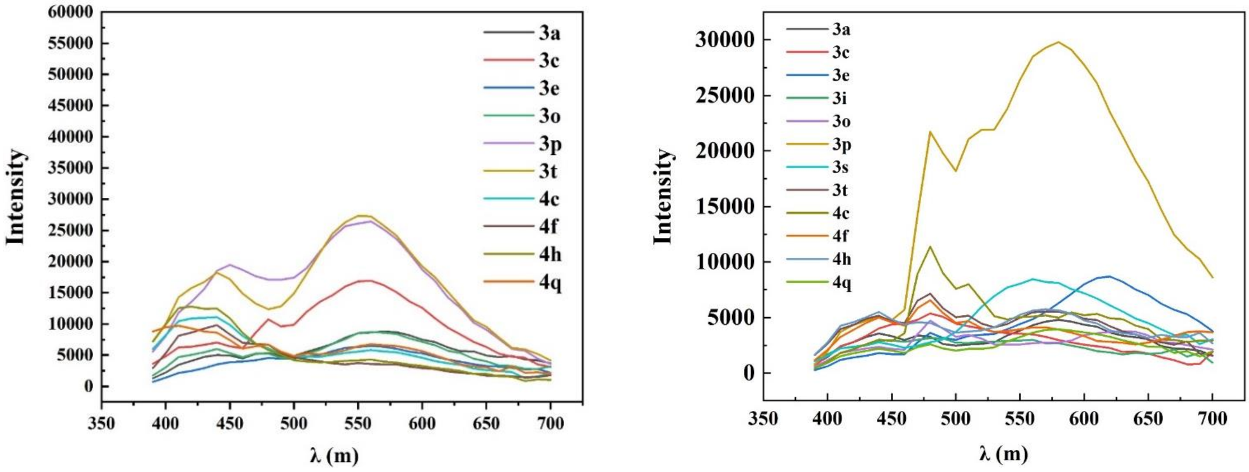

Supplementary Fig. 2a: Emission spectra of chiral and racemic homologated adducts.

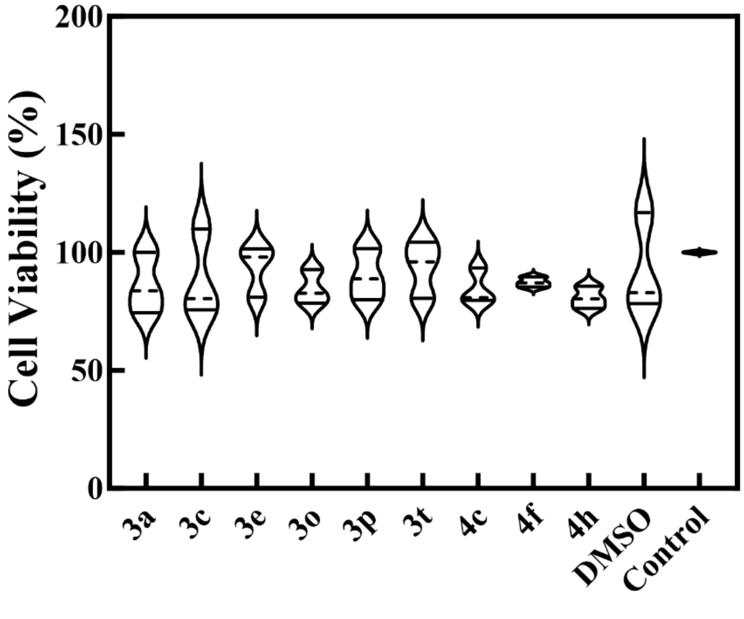

Supplementary Fig. 2b: *In-vitro* cytocompatibility of the developed chiral homologated adducts. The data represents the mean and standard deviation of three independent samples (n=3).

5. Experimental Section:

A. General procedure for the preparation of 3-Cyano-4-methylcoumarins (1a-1s).

3-cyano-4-mthylcoumarins (**1a-1t**) were prepared according to published procedures.[1] *o*-hydroxyacetophenones (1 equiv.), ethyl cyanoacetate (1.5 equiv.), and ammonium acetate (2.5 equiv.) are charged sequentially into a 250 mL round bottom flask under air, fitted with a reflux condenser and heated to 165 °C (oil bath temperature) for 3-5 h. The mixture was then cooled to rt, 95% EtOH (100 mL) was added, and the mixture was triturated overnight at rt. The precipitated solid is then filtered and recrystallized in an acetone/H_2O mixture, filtered, washed with hexane, and dried under a high vacuum pump to yield coumarins.

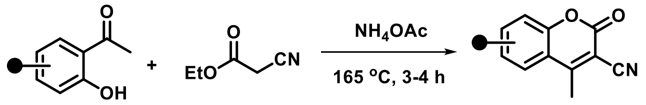

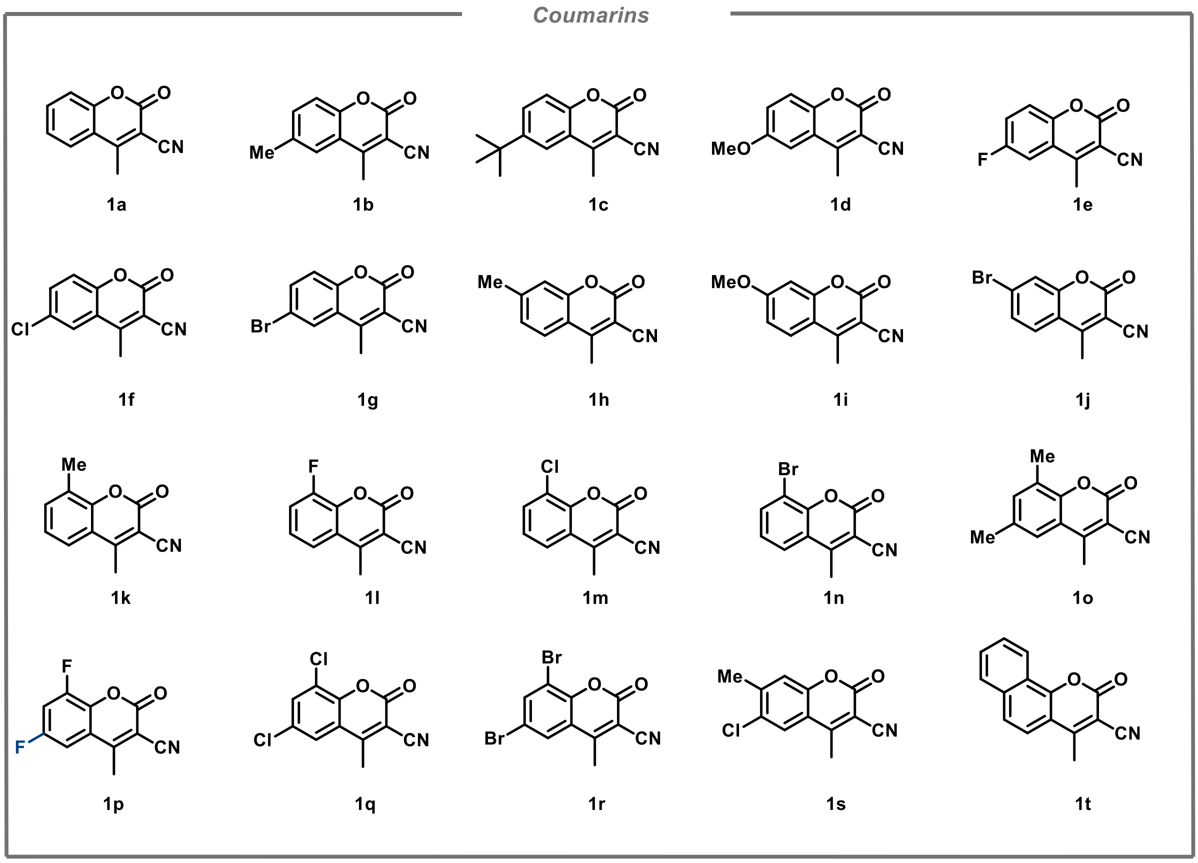

B. General procedure 1: Synthesis of cyclopenta-1,3-dienones (2a-2s):

Cyclopent-4-en e-1,3-diones (**2a-2ac**) were prepared according to the reported procedure.[2]

Step 1: To a suspension of 2-Methyl-1,3-cyclopentanedione in H_2O (1 mol/L) was added $NaHCO_3$ portion wise (1.2 equiv.) and was allowed to stir at ambient temperature until frothing settled. This was followed by addition of benzyl bromide (2 equiv.). the reaction mixture was stirred overnight at 80 ^oC. After that reaction mixture was allowed to cool down to room temperature, the reaction mixture was then extracted with ethyl acetate (30 ml × 3). The combined organic phase was dried over Na_2SO_4. After concentrating the solvent under reduced pressure, the crude mixture was purified through silica gel column chromatography to 2-benzyl-2-methylcyclopentane-1,3-diones.

Step 2: To a solution of 2-benzyl-2-methylcyclopentane-1,3-diones (1.0 equiv.) in MeOH (5 ml /mmol) was added $CuBr_2$ (2 equiv.) and the resulting brown solution was stirred at 90 °C under N_2 atmosphere. After 1 h the reaction mixture was cooled to room temperature and quenched with water followed by 1 M aq. HCl solution. The aqueous phase was extracted with ethyl acetate 3 times. The combined organic phase was dried over Na_2SO_4 and concentrated under reduced pressure. The crude reaction mixture was purified by silica gel column chromatography (10% EtOAc in petroleum ether) to obtain a yellow crystalline solid.

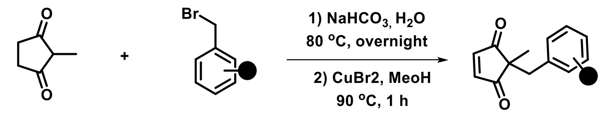

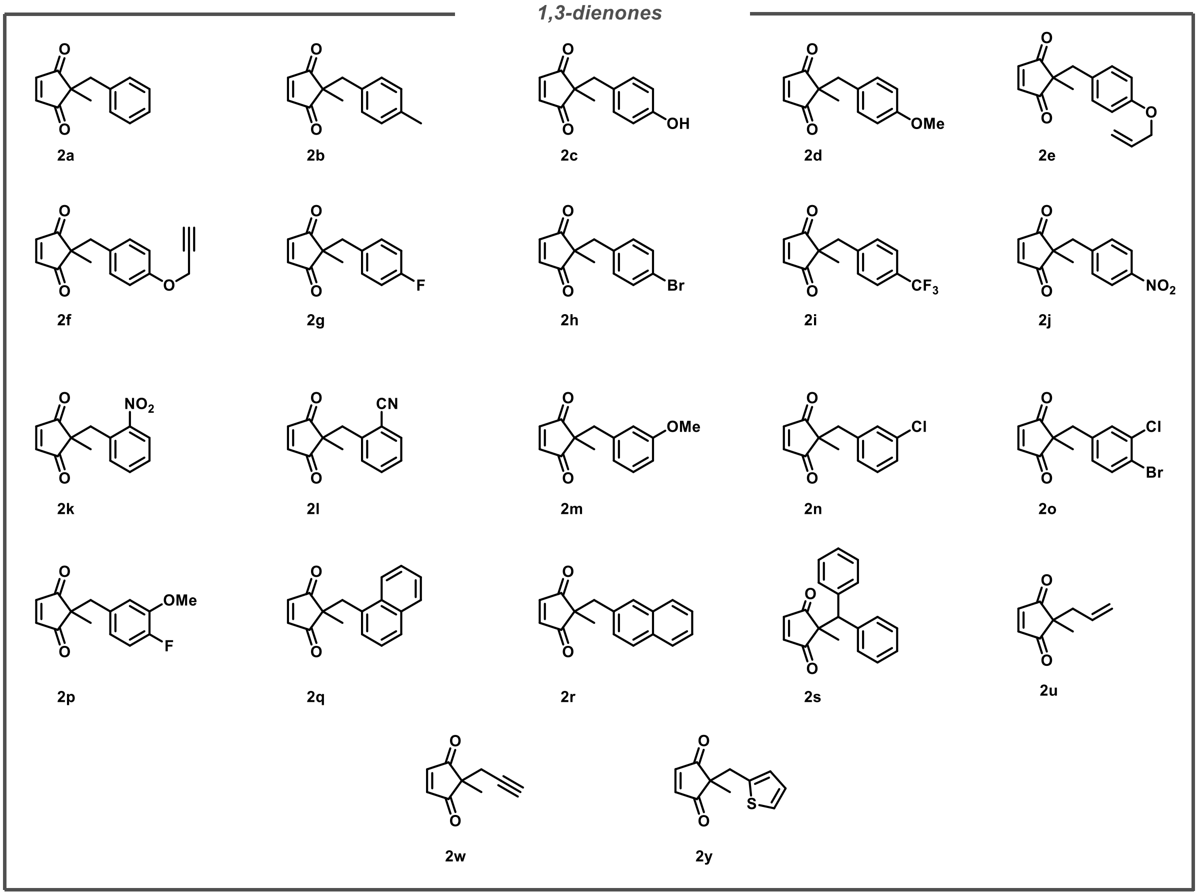

C. General procedure 2: Synthesis of 2t, 2v, and 2x:

Cyclopent-4-ene-1,3-dione (**2t, 2v** and **2x**) were synthesized according to modified literature procedure.[3]

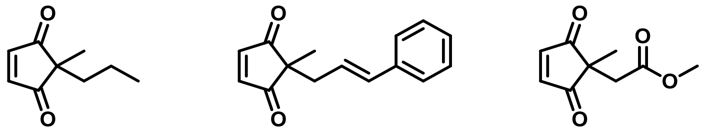

2t **2v** **2x**

D. General procedure 4: Synthesis of 2c.[4]

Step 1: To a suspension of 2-methyl-1,3-cyclopentanedione (4.7 g, 42.3 mmol, 1.5 equiv) in water (40 mL) was added *p*-hydroxybenzyl alcohol (3.6 g, 28.2 mmol, 1.0 equiv), and the solution was stirred at 80 °C for 12 h. The reaction mixture was extracted with EtOAc (2 x 20 mL) and combined organic solvent was dried over anhydrous Na_2SO_4 and purified via column chromatography (1:1 EtOAc/hexanes as the solvent system) to give 93% of **2c.**

Step 2: The olefination of the above intermediate to afford **2c** was performed by following, general procedure 1, step 2.

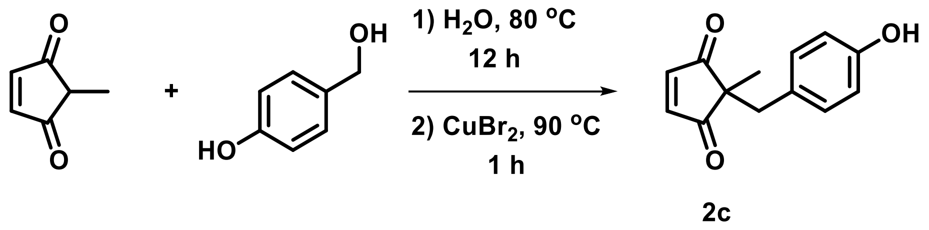

E. General procedure 4: Synthesis of 2e and 2f.

To a suspension of **2c** (1.4 mmol) and K_2CO_3 (2.8 mmol, 2 equiv) in THF (20 mL). Add allyl bromide/propargyl bromide (2.8 mmol, 2 equiv.) to the mixture. Stir the reaction mixture until the reaction completes. Monitor the progress of the reaction by TLC. Filter the reaction mixture over a pad of Celite and evaporate the solvent under reduced pressure to afford crude product and is purified through silica gel chromatography (EtOAc/Hexane 15:85).

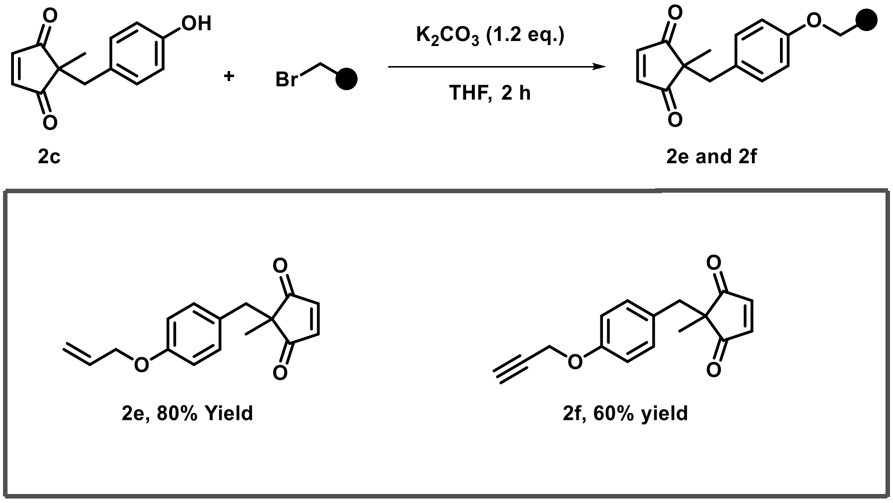

F. General procedure 4: Synthesis of 2z, 2aa, 2ab, 2ac and 2ad:

To a solution of **2c** (1.9 mmol, 1 equiv.) and corresponding acids (1.9 mmol 1 equiv.) in 15 mL dry DCM were added EDC (2.22 mmol, 1.2 equiv.) and DMAP (0.46 mmol, 0.25 equiv.). The reaction mixture was allowed to stir for 18 h at rt. The mixture was quenched with saturated $NaHCO_3$ and diluted with H_2O. Then extract the solution with CH_2Cl_2 (40 ml × 3) and washed with brine solution. The combined organic phase was then concentrated under reduced pressure and purified through silica gel chromatography.

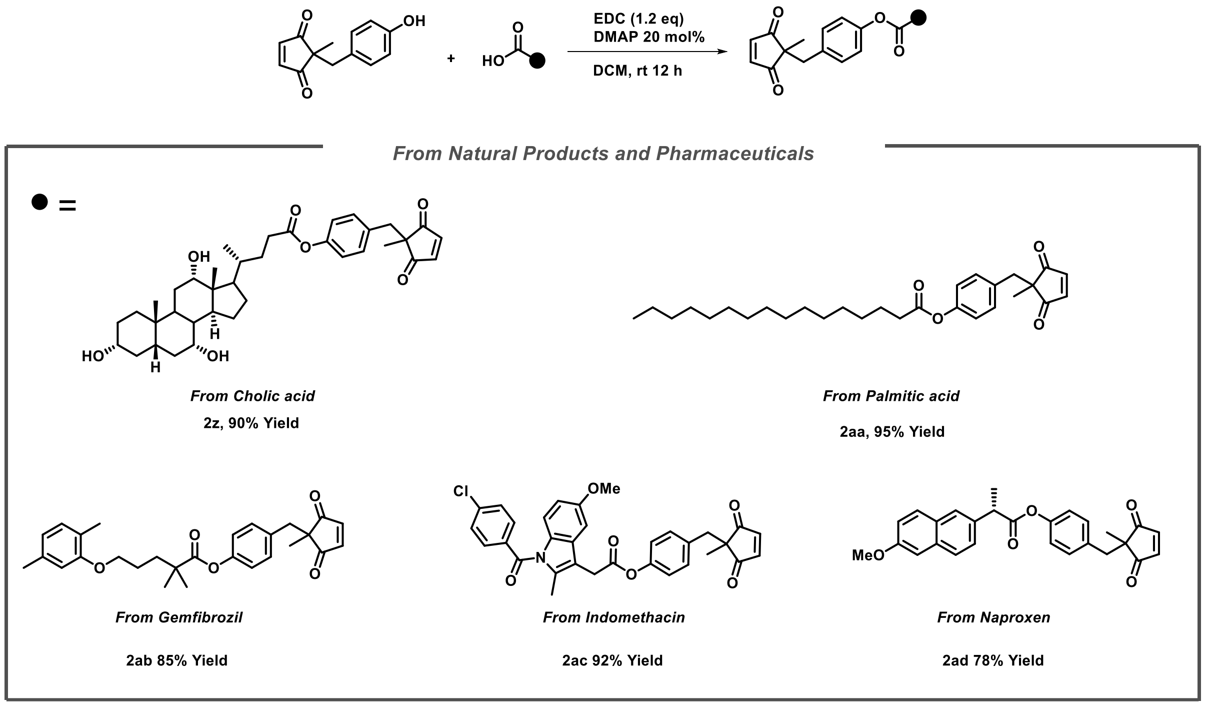

G. General procedure for the preparation of racemic homologated adducts.

In an oven and vacuum-dried reaction tube, 3-cynao-4-methylcoumarin **1** (0.1 mmol, 1.0 equiv) and 1,3 diketone **2** (0.12 mmol, 1.2 equiv) were taken in 2 ml of freshly distilled $CHCl_3$. After stirring for 5 min at rt, DBU (3 µL, 0.01 mmol, 0.1 equiv) was added to the reaction medium. The reaction mixture was allowed to stir in open air for 5 min at rt. Further, the reaction was quenched by adding 200 mg silica gel and kept stirring for 30 min at rt. The reaction was then directly processed for purification by silica gel column chromatography (eluent: EtOAc/Petroleum ether = 35/65, v/v) without any workup **3** and **4**.

H. General procedure for the preparation of chiral homologated adducts.

In an oven and vacuum-dried reaction tube, catalyst **C6** (11.1 mg, 0.02 mmol, 0.2 equiv), 1,3-diketone **2** (0.12 mmol, 1.2 equiv.) were taken in freshly $CHCl_3$ (1 mL) at room temperature. The reaction mixture was cooled at 0 ^{o}C. Subsequently, 3-cynao-4-methylcoumarin **1** (0.1 mmol, 1.0 equiv.) was charged to the reaction mixture in one shot under O_2/open air atmosphere. The resulting mixture was allowed to stir vigorously at the same temperature for 72 h and the progress of the reaction was monitored through TLC. Once the reaction was completed, the reaction mixture was quenched with silica-gel 200 mg and was further stirred at rt for 30 mins. The reaction was then directly processed for purification by silica gel column chromatography (eluent: EtOAc/Petroleum ether = 35/65, v/v) without any workup to afford chiral products **3** and **4**.

Note: After 72 h of reaction time, we observed that the reaction mixture becomes brownish in color. When 200 mg silica gel was added to the reaction mixture, the color of the mixture turned deep blue, which is the color of our products.

6. Characterization of starting materials and products.

(allyloxy)benzyl)-2-methylcyclopent-4-ene-1,3-dione 2d: The title compound was prepared by using the general reaction procedure **E** and purified directly by silica gel column chromatography (eluent: EtOAc/Petroleum ether = 15/85, v/v); light yellow liquid **2e** (287 mg, 80% yield) was obtained.

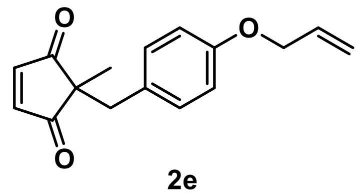

2e

^{1}H NMR (500 MHz, CDCl$_3$) δ 7.00 (s, 2H), 6.84 (d, *J* = 8.2 Hz, 2H), 6.71 (d, *J* = 8.5 Hz, 1H), 6.08 – 5.87 (m, 1H), 5.37 (d, *J* = 18.2 Hz, 1H), 5.26 (d, *J* = 7.5 Hz, 1H), 4.45 (d, *J* = 3.7 Hz, 2H), 2.94 (s, 2H), 1.23 (s, 3H).

^{13}C NMR (126 MHz, CDCl$_3$) δ 207.5, 157.0, 149.6, 133.2, 130.9, 127.8, 118.4, 114.9, 68.7, 52.6, 41.5, 18.7.

HRMS ESI: [M+Na]$^+$, Calcd for $C_{16}H_{16}NaO_3$ 279.0997; found 279.0989.

2-methyl-2-(4-(prop-2-yn-1-yloxy)benzyl)cyclopent-4-ene-1,3-dione: The title compound was prepared by using the general reaction procedure E and purified directly by silica gel column chromatography (eluent: EtOAc/Petroleum ether = 15/85, v/v); light yellow liquid **2f** (214 mg, 60% yield) was obtained.

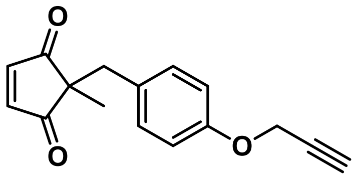

2f

^{1}H NMR (500 MHz, CDCl$_3$) δ 7.01 (s, 2H), 6.87 (d, *J* = 8.9 Hz, 2H), 6.77 (d, *J* = 8.7 Hz, 2H), 4.61 (d, *J* = 2.4 Hz, 2H), 2.95 (s, 2H), 2.50 (t, *J* = 2.4 Hz, 1H), 1.24 (s, 3H).

^{13}C NMR (126 MHz, CDCl$_3$) δ 207.9, 156.6, 148.5, 131.3, 128.6, 113.4, 79.0, 75.5, 55.3, 52.6, 40.0, 18.7.

HRMS ESI: [M+Na]$^+$, Calcd for $C_{16}H_{14}NaO_3$ 277.0841; found 277.0831.

4-((1-methyl-2,5-dioxocyclopent-3-en-1-yl)methyl)phenyl (4S)-4-((3S,5R,7S,10R,12R,13S)-3,7,12-trihydroxy-10,13-dimethylhexadecahydro-1H-cyclopenta[a]phenanthren-17-yl)pentanoate: The title compound was prepared by using the general reaction procedure **F** and purified directly by silica gel column chromatography (eluent: EtOAc/Petroleum ether = 35/65, v/v); yellow solid **2z** (1 gm, 90% yield) was obtained.

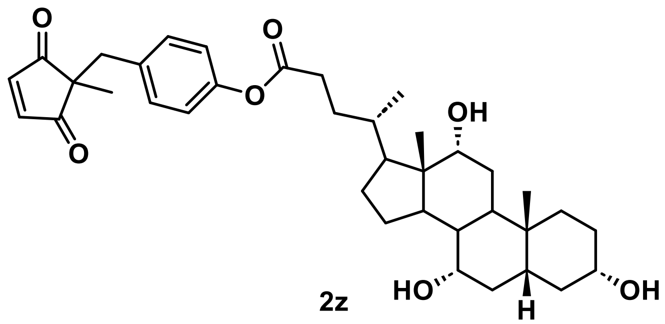

2z

^{1}H NMR (500 MHz, $CDCl_3$) δ 7.01 (s, 2H), 6.93 (d, *J* = 8.1 Hz, 2H), 6.86 (d, *J* = 8.0 Hz, 2H), 3.95 (s, 1H), 3.83 (s, 1H), 3.42 (s, 1H), 2.97 (s, 4H), 2.65 – 2.56 (m, 1H), 2.43 (dt, *J* = 15.4, 7.7 Hz, 1H), 2.21 (dd, *J* = 15.0, 10.1 Hz, 2H), 2.02 – 1.82 (m, 4H), 1.79 – 1.62 (m, 3H), 1.54 (q, *J* = 17.3, 16.1 Hz, 3H), 1.50 – 1.30 (m, 4H), 1.25 (d, *J* = 6.2 Hz, 5H), 1.18 – 1.04 (m, 1H), 1.02 (d, *J* = 5.4 Hz, 3H), 0.88 (s, 3H), 0.68 (s, 3H).

^{13}C NMR (126 MHz, $CDCl_3$) δ 207.2, 172.6, 149.7, 148.8, 133.0, 130.7, 121.5, 73.1, 71.9, 68.4, 60.4, 52.5, 47.0, 46.5, 41.6, 41.5, 40.0, 39.5, 35.3, 34.8, 34.7, 31.4, 30.8, 30.4, 28.2, 27.6, 26.3.

HRMS ESI: $[M+Na]^+$, Calcd for $C_{37}H_{50}NaO_7$ 629.3454; found 629.3447.

4-((1-methyl-2,5-dioxocyclopent-3-en-1-yl)methyl)phenyl palmitate: The title compound was prepared by using the general reaction procedure **F** and purified directly by silica gel column chromatography (eluent: EtOAc/Petroleum ether = 15/85, v/v); yellow oil **2aa** (821 mg, 95% yield) was obtained.

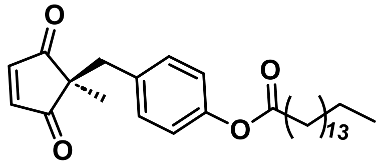

2aa

^{1}H NMR (500 MHz, $CDCl_3$) δ 7.02 (s, 2H), 6.95 (d, *J* = 8.1 Hz, 2H), 6.88 (d, *J* = 8.1 Hz, 2H), 2.99 (s, 2H), 2.51 (t, *J* = 7.6 Hz, 2H), 1.72 (p, *J* = 7.5 Hz, 2H), 1.27 (s, 26H), 0.89 (t, *J* = 6.9 Hz, 3H).

^{13}C NMR (126 MHz, $CDCl_3$) δ 207.9, 172.2, 149.8, 148.8, 133.0, 130.7, 121.5, 52.5, 40.0, 34.3, 31.9, 29.7, 29.6, 29.5, 29.4, 29.3, 29.1, 24.9, 22.7, 19.4, 14.1.

HRMS ESI: $[M+Na]^+$, Calcd for $C_{29}H_{42}NaO_4$ 477.2981; found 477.2971.

4-((1-methyl-2,5-dioxocyclopent-3-en-1-yl)methyl)phenyl 5-(2,5-dimethylphenoxy)-2,2-dimethylpentanoate (2ab): The title compound was prepared by using the general reaction procedure **F** and purified directly by silica gel column chromatography (eluent: EtOAc/Petroleum ether = 15/85, v/v); yellow solid **2ab** (724 mg, 85% yield) was obtained.

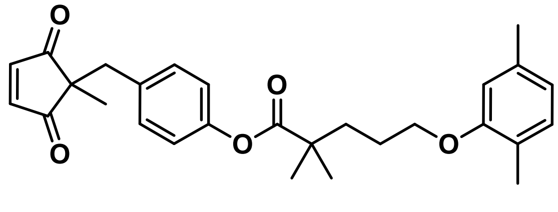

2ab

^{1}H NMR (500 MHz, $CDCl_3$) δ 7.06 – 7.00 (m, 3H), 6.96 (d, *J* = 8.1 Hz, 2H), 6.86 (d, *J* = 8.1 Hz, 2H), 6.70 (d, *J* = 7.4 Hz, 1H), 6.65 (s, 1H), 4.07 – 3.95 (m, 2H), 3.01 (s, 2H), 2.34 (s, 3H), 2.19 (s, 3H), 1.91 – 1.80 (m, 4H), 1.37 (s, 6H), 1.28 (s, 3H).

^{13}C NMR (126 MHz, $CDCl_3$) δ 207.1, 176.3, 156.9, 150.0, 148.8, 136.5, 133.0, 130.7, 130.4, 123.6, 121.5, 120.8, 112.0, 67.8, 52.5, 42.4, 40.1, 37.1, 29.7, 25.3, 25.1, 21.4, 19.4, 15.8.

HRMS ESI: $[M+Na]^+$, Calcd for $C_{28}H_{32}NaO_5$ 471.2147; found 471.2136.

2-(4-(3-(1-(4-chlorobenzoyl)-5-methoxy-2-methyl-1H-indol-3-yl)-2 oxopropoxy)benzyl)-2-methylcyclopent-4-ene-1,3-dione: The title compound was prepared by using the general reaction procedure **F** and purified directly by silica gel column chromatography (eluen t: EtOAc/Petroleum ether = 20/80, v/v); yellow solid **2ac** (972 mg, 92% yield) was obtained.

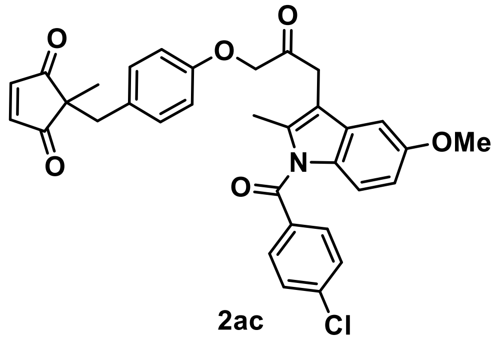

2ac

^{1}H NMR (500 MHz, $CDCl_3$) δ 7.69 (d, *J* = 8.2 Hz, 2H), 7.49 (d, *J* = 8.2 Hz, 2H), 7.03 (d, *J* = 2.6 Hz, 1H), 7.02 (s, 2H), 6.94 (d, *J* = 8.2 Hz, 2H), 6.89 (t, *J* = 7.9 Hz, 3H), 6.71 (dd, *J* = 9.0, 2.6 Hz, 1H), 3.87 (s, 2H), 3.85 (s, 3H), 2.99 (s, 2H), 2.45 (s, 3H), 1.26 (s, 3H).

^{13}C NMR (126 MHz, $CDCl_3$) δ 207.1, 169.2, 168.3, 156.1, 149.7, 148.8, 139.4, 136.2, 133.8, 133.3, 131.2, 130.8, 130.7, 130.5, 129.2, 121.3, 115.0, 111.9, 111.7, 101.3, 55.8, 52.5, 39.9, 30.5, 19.5, 13.4.

HRMS ESI: $[M+Na]^+$, Calcd for $C_{32}H_{26}ClNNaO_6$ 578.1346; found 578.1342.

(*R*)-7-amino-10-benzyl-10-methylindeno[2,1-c]chromene-6,9,11(10H)-trione (3a): The title compound was prepared by using the general reaction procedure and purified directly by silica gel column chromatography (eluent: EtOAc/Petroleum ether = 35/65, v/v); dark blue **3a** (28 mg, 73% yield) was obtained. The *er* of the **3a** was determined to be 97:3 [determined by HPLC, Chiralpak ADH, hexane: isopropanol = 80:20, 1 mL/min, λ = 265 nm, t (major) = 5.4 min, t (minor) = 7.8 min]. $[\alpha]^{25}_D$ = −866.6° (c = 0.03, $CHCl_3$)

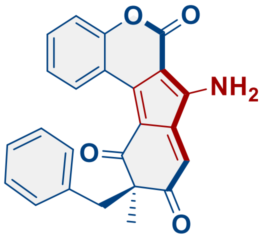

3a

^{1}H NMR (400 MHz, DMSO-*d*$_6$) δ 10.33 (s, 1H), 9.80 (d, *J* = 9.8 Hz, 1H), 9.42 (s, 1H), 7.70 (t, *J* = 7.8 Hz, 1H), 7.35 (t, *J* = 7.6 Hz, 1H), 7.29 (d, *J* = 8.2 Hz, 1H), 7.13 – 7.04 (m, 4H), 6.91 (d, *J* = 7.9 Hz, 2H), 3.24 (d, *J* = 13.3 Hz, 1H), 3.04 (d, *J* = 13.3 Hz, 1H), 1.31 (s, 3H).

^{13}C NMR (126 MHz, DMSO-*d*$_6$) δ 203.0, 190.2, 164.1, 158.2, 156.1, 155.6, 151.3, 137.6, 136.2, 132.4, 129.9, 128.3, 126.7, 124.6, 120.6, 117.9, 117.1, 105.4, 96.8, 62.5, 44.1, 23.8.

HRMS ESI: $[M+Na]^+$, Calcd for $C_{24}H_{17}NNaO_4$ 406.1050; found 406.1049.

(*R*)-7-amino-10-benzyl-2,10-dimethylindeno[2,1-c]chromene-6,9,11(10H)-trione (3b): The title compound was prepared by using the general reaction procedure and purified directly by silica gel column chromatography (eluent: EtOAc/Petroleum ether = 35/65, v/v); deep blue solid **3b** (28.3 mg, 71% yield) was obtained. The *er* of the **3b** was determined to be 97:3 [determined by HPLC, Chiralpak ADH, hexane: isopropanol = 70:30, 1 mL/min, λ = 260 nm, t (major) = 4.5 min, t (minor) = 5.7 min. $[\alpha]^{30}_D$ = –465.9° (c = 0.043, $CHCl_3$)

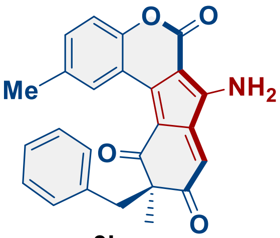

3b

^{1}H NMR (400 MHz, $CDCl_3$) δ 9.55 (d, *J* = 2.1 Hz, 1H), 8.10 (s, 1H), 8.00 (s, 1H), 7.41 (d, *J* = 8.3 Hz, 1H), 7.17 – 7.02 (m, 4H), 7.00-6.97 (m, 2H), 6.74 (s, 1H), 3.35 (d, *J* = 13.3 Hz, 1H), 3.12 (d, *J* = 13.3 Hz, 1H), 2.44 (s, 3H), 1.48 (s, 3H).

^{13}C NMR (126 MHz, $CDCl_3$) δ 203.6, 190.7, 162.6, 160.8, 156.1, 154.0, 150.8, 137.1, 136.6, 134.7, 132.5, 129.8, 128.1, 126.2, 121.5, 117.5, 116.8, 106.4, 98.9, 63.2, 45.6, 22.9, 21.1.

HRMS ESI: $[M+H]^+$, Calcd for $C_{25}H_{20}NO_4$ 398.1387; found 398.1392.

(*R*)-7-amino-10-benzyl-2-methoxy-10-methylindeno[2,1-*c*]chromene-6,9,11(10*H*)-trione

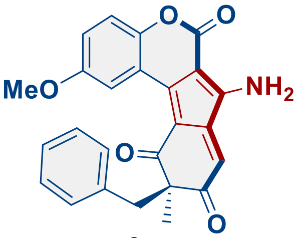

3c

(3c): The title compound was prepared by using the general reaction procedure and purified directly by silica gel column chromatography (eluent: EtOAc/Petroleum ether = 40/60, v/v); green solid **3c** (29.8 mg, 72% yield) was obtained. The *er* of the **3c** was determined to be 98:2 [determined by HPLC, Chiralpak ADH, hexane: isopropanol = 70:30, 1 mL/min, λ = 260 nm, t (major) = 4.6 min, t (minor) = 5.8 min]. $[\alpha]^{31}_{D}$ = –119.5° (c = 0.11, $CHCl_3$)

^{1}H NMR (400 MHz, $CDCl_3$) δ 9.48 (d, *J* = 3.0 Hz, 1H), 8.10 (s, 1H), 7.40 (s, 1H), 7.22 (dd, *J* = 9.0, 3.0 Hz, 1H), 7.20 (d, *J* = 9.0 Hz, 1H), 7.17 – 7.07 (m, 3H), 7.04 – 6.97 (m, 2H), 6.66 (s, 1H), 3.97 (s, 3H), 3.34 (d, *J* = 13.2 Hz, 1H), 3.13 (d, *J* = 13.2 Hz, 1H), 1.52 (s, 3H).

^{13}C NMR (126 MHz, $CDCl_3$) δ 203.0, 190.8, 161.6, 159.8, 156.2, 150.0, 136.8, 129.9, 128.0, 126.8, 124.6, 120.5, 118.7, 116.8, 114.0, 106.1, 99.3, 63.2, 56.9, 45.6, 22.8.

HRMS ESI: $[M+Na]^+$, Calcd for $C_{25}H_{19}NNaO_5$ 436.1161; found 436.1153.

(*R*)-7-amino-10-benzyl-2-(tert-butyl)-10-methylindeno[2,1-*c*]chromene-6,9,11(10*H*)-

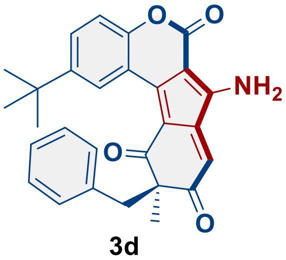

3d

trione (3d): The title compound was prepared by using the general reaction procedure and purified directly by silica gel column chromatography (eluent: EtOAc/Petroleum ether = 35/65, v/v); deep blue solid **3d** (28 mg, 64% yield) was obtained. The *er* of the **3d** was determined to be 96:4 [determined by HPLC, Chiralpak ADH, hexane: isopropanol = 90:10, 1 mL/min, λ = 260 nm, t (major) = 6.7 min, t (minor) = 8.6 min]. $[\alpha]^{27}_{D}$ = –108.7° (c = 0.046, $CHCl_3$)

^{1}H NMR (500 MHz, DMSO-d_6) δ 10.32 (s, 1H), 9.91 (d, *J* = 2.5 Hz, 1H), 9.40 (s, 1H), 7.74 (dd, *J* = 8.7, 2.6 Hz, 1H), 7.22 (d, *J* = 8.7 Hz, 1H), 7.11 (s, 1H), 7.10 – 7.05 (m, 3H), 6.92 (dd, *J* = 7.3, 2.3 Hz, 2H), 3.18 (d, *J* = 13.3 Hz, 1H), 3.05 (d, *J* = 13.3 Hz, 1H), 1.36 (s, 9H), 1.30 (s, 3H).

^{13}C NMR (126 MHz, DMSO-d_6) δ 203.0, 190.3, 164.0, 158.5, 156.5, 153.5, 151.5, 147.2, 137.5, 133.5, 130.0, 129.2, 128.2, 126.7, 120.4, 117.3, 116.6, 105.3, 96.8, 62.7, 44.5, 35.1, 31.6, 23.0.

HRMS ESI: $[M+Na]^+$, Calcd for $C_{28}H_{25}NNaO_4$ 462.1676; found 462.1658.

(*R*)-7-amino-10-benzyl-2-fluoro-10-methylindeno[2,1-*c*]chromene-6,9,11(10*H*)-trione

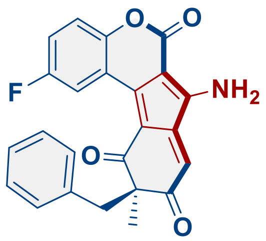

3e

(3e): The title compound was prepared by using the general reaction procedure and purified directly by silica gel column chromatography (eluent: EtOAc/Petroleum ether = 35/65, v/v); deep blue solid **3e** (24.4 mg, 62% yield) was obtained. The *er* of the **3e** was determined to be 95:5 [determined by HPLC, Chiralpak ADH, hexane: isopropanol = 70:30, 1 mL/min, λ = 260 nm, t (major) = 5.1 min, t (minor) = 7.4 min]. $[\alpha]^{29}_{D}$ = –156.9° (c = 0.065, $CHCl_3$) **^{1}H NMR** (500 MHz, DMSO-d_6) δ 10.45 (s, 1H), 9.70 (dd, *J* = 10.7, 3.2 Hz, 1H), 9.53 (s, 1H), 7.66 – 7.53 (m, 1H), 7.36 (dd, *J* = 9.1, 4.8 Hz, 1H), 7.16 – 7.04 (m, 4H), 6.90 (d, *J* = 6.0 Hz, 2H), 3.22 (d, *J* = 13.4 Hz, 1H), 3.04 (d, *J* = 13.4 Hz, 1H), 1.32 (s, 3H).

^{13}C NMR (126 MHz, DMSO-d_6) δ 202.8, 190.6, 164.0, 158.0, 156.9, 155.3, 152.1, 150.9, 137.4, 129.9, 128.3, 126.8, 123.3, 123.1, 121.4, 119.8, 119.7, 118.0, 117.9, 117.3, 117.1, 105.6, 96.6, 62.5, 44.3, 23.5.

HRMS ESI: $[M+H]^+$, Calcd for $C_{24}H_{17}FNO_4$ 402.1142; found 402.1158.

(*R*)-7-amino-10-benzyl-2-chloro-10-methylindeno[2,1-*c*]chromene-6,9,11(10*H*)-trione

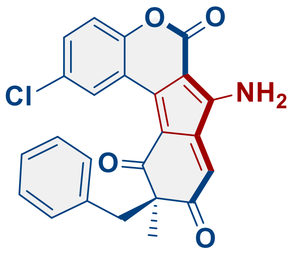

3f

(3f): The title compound was prepared by using the general reaction procedure and purified directly by silica gel column chromatography (eluent: EtOAc/Petroleum ether = 35/65, v/v); deep blue solid **3f** (27.3 mg, 66% yield) was obtained. The *er* of the **3f** was determined to be 94:6 [determined by HPLC, Chiralpak ADH, hexane: isopropanol = 70:30, 1 mL/min, λ = 260 nm, t (major) = 5.4 min, t (minor) = 7.4 min]. $[\alpha]^{29}_{D}$ = +124.4° (c = 0.071, $CHCl_3$).

^{1}H NMR (500 MHz, DMSO-d_6) δ 10.46 (s, 1H), 10.06 (d, *J* = 2.6 Hz, 1H), 9.55 (s, 1H), 7.87 (dd, *J* = 8.8, 2.5 Hz, 1H), 7.29 (d, *J* = 8.8 Hz, 1H), 7.17 – 7.06 (m, 4H), 6.90 (d, *J*=6.3 Hz 2H), 3.25 (d, *J* = 13.4 Hz, 1H), 3.04 (d, *J* = 13.4 Hz, 1H), 1.33 (s, 3H).

^{13}C NMR (126 MHz, DMSO-d_6) δ 202.3, 190.3, 165.9, 163.5, 157.3, 154.2, 150.4, 137.9, 137.0, 133.5, 129.4, 127.9, 126.4, 121.1, 119.8, 118.4, 115.9, 105.3, 96.2, 62.1, 43.8, 23.0.

HRMS ESI: $[M+Na]^+$, Calcd for $C_{24}H_{16}ClNNaO_4$ 417.0768; found 417.0772.

(*R*)-7-amino-10-benzyl-2-bromo-10-methylindeno[2,1-*c*]chromene-6,9,11(10*H*)-trione

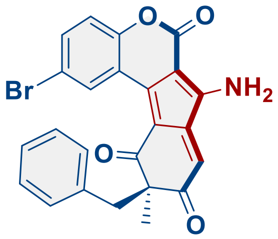

3g

(3g): The title compound was prepared by using the general reaction procedure and purified directly by silica gel column chromatography (eluent: EtOAc/Petroleum ether = 35/65, v/v); deep blue solid **3g** (28.4 mg, 62% yield) was obtained. The *er* of the **3g** was determined to be 97:3 [determined by HPLC, Chiralpak ADH, hexane: isopropanol = 70:30, 1 mL/min, λ = 260 nm, t (major) = 5.4 min, t (minor) = 7.3 min]. $[\alpha]^{29}_{D}$ = –152.6° (c = 0.11, $CHCl_3$).

^{1}H NMR (400 MHz, DMSO-*d*$_6$) δ 10.41 (s, 1H), 10.03 (d, *J* = 2.5 Hz, 1H), 9.48 (s, 1H), 7.84 (dd, *J* = 8.8, 2.6 Hz, 1H), 7.25 (d, *J* = 8.8 Hz, 1H), 7.14 – 7.06 (m, 4H), 6.89 (dd, *J* = 7.5, 2.0 Hz, 2H), 3.24 (d, *J* = 13.4 Hz, 1H), 3.04 (d, *J* = 13.4 Hz, 1H), 1.32 (s, 3H).

^{13}C NMR (126 MHz, DMSO-*d*$_6$) δ 202.7, 190.6, 163.9, 157.7, 154.7, 150.8, 138.2, 137.4, 133.9, 129.9, 128.3, 126.8, 121.4, 120.2, 118.8, 116.3, 105.7, 96.6, 62.5, 44.3, 23.3.

HRMS ESI: $[M+Na]^+$, Calcd for $C_{24}H_{16}BrNNaO_4$ 484.0155; found 484.0155.

(*R*)-7-amino-10-benzyl-3,10-dimethylindeno[2,1-*c*]chromene-6,9,11(10*H*)-trione (3h):

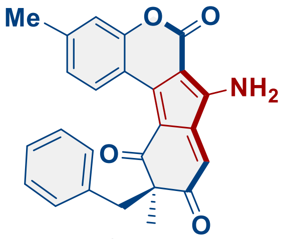

3h

The title compound was prepared by using the general reaction procedure and purified directly by silica gel column chromatography (eluent: EtOAc/Petroleum ether = 35/65, v/v); deep blue solid **3h** (25.4 mg, 63% yield) was obtained. The *er* of the **3h** was determined to be 97:3 [determined by HPLC, Chiralpak ADH, hexane: isopropanol = 70:30, 1 mL/min, λ = 260 nm, t (major) = 5.2 min, t (minor) = 6.8 min]. $[\alpha]^{29}_{D}$ = +41.0° (c = 0.097, $CHCl_3$).

^{1}H NMR (500 MHz, DMSO-*d*$_6$) δ 10.30 (s, 1H), 9.71 (d, *J* = 8.3 Hz, 1H), 9.40 (s, 1H), 7.21 (d, *J* = 8.3, 1.8 Hz, 1H), 7.15 (s, 1H), 7.13 – 7.04 (m, 4H), 6.94 – 6.88 (m, 2H), 3.23 (d, *J* = 13.4 Hz, 1H), 3.05 (d, *J* = 13.3 Hz, 1H), 2.44 (s, 3H), 1.32 (s, 3H).

^{13}C NMR (126 MHz, DMSO-*d*$_6$) δ 203.0, 190.2, 164.1, 158.4, 156.1, 155.7, 151.5, 147.9, 137.7, 132.3, 129.9, 128.3, 126.7, 125.8, 120.1, 118.0, 114.7, 105.0, 96.6, 62.4, 44.2, 23.9, 22.0.

HRMS ESI: $[M+Na]^+$, Calcd for $C_{25}H_{19}NNaO_4$ 420.1206; found 420.1206.

(*R*)-7-amino-10-benzyl-3-methoxy-10-methylindeno[2,1-c]chromene-6,9,11(10H)-trione

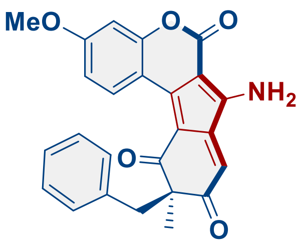

3i

(3i): The title compound was prepared by using the general reaction procedure and purified directly by silica gel column chromatography (eluent: EtOAc/Petroleum ether = 35/65, v/v); deep blue solid **3i** (28.9 mg, 70% yield) was obtained. The *er* of the **3i** was determined to be 97:3 [determined by HPLC, Chiralpak ADH, hexane: isopropanol = 80:20, 1 mL/min, λ = 260 nm, t (major) = 8.4 min, t (minor) = 15.1 min]. $[\alpha]^{29}_{D}$ = +18.6° (c = 0.11, $CHCl_3$).

^{1}H NMR (500 MHz, DMSO-d_6) δ 10.25 (s, 1H), 9.80 (d, *J* = 9.1 Hz, 1H), 9.35 (s, 1H), 7.14–7.04 (m, 3H), 6.99 (s, 1H), 6.96 – 6.89 (m, 3H), 6.86 (d, *J* = 2.6 Hz, 1H), 3.90 (s, 3H), 3.24 (d, *J* = 13.4 Hz, 1H), 3.04 (d, *J* = 13.4 Hz, 1H), 1.31 (s, 3H).

^{13}C NMR (126 MHz, DMSO-d_6) δ 203.2, 190.1, 167.0, 164.2, 158.2, 158.0, 156.1, 151.7, 137.8, 134.4, 129.8, 128.3, 126.7, 118.8, 112.3, 110.5, 104.1, 102.1, 96.0, 62.3, 56.6, 44.0, 24.4.

HRMS ESI: $[M+H]^+$, Calcd for $C_{25}H_{20}NO_5$ 414.1336; found 414.1321.

(*R*)-7-amino-10-benzyl-3-bromo-10-methylindeno[2,1-*c*]chromene-6,9,11(10*H*)-trione

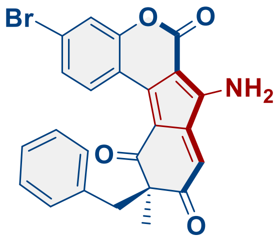

3j

(3j): The title compound was prepared by using the general reaction procedure and purified directly by silica gel column chromatography (eluent: EtOAc/Petroleum ether = 35/65, v/v); deep blue solid **3j** (27.7 mg, 60% yield) was obtained. The *er* of the **3j** was determined to be 97:3 [determined by HPLC, Chiralpak ADH, hexane: isopropanol = 70:30, 1 mL/min, λ = 260 nm, t (major) = 5.6 min, t (minor) = 8.5 min]. $[\alpha]^{30}_{D}$ = –66.1° (c = 0.13, $CHCl_3$).

^{1}H NMR (400 MHz, DMSO-d_6) δ 10.42 (s, 1H), 9.75 (d, *J* = 8.7 Hz, 1H), 9.50 (s, 1H), 7.64 – 7.53 (m, 2H), 7.17 – 7.04 (m, 4H), 6.90 (dd, *J* = 7.5, 2.0 Hz, 2H), 3.22 (d, *J* = 13.4 Hz, 1H), 3.05 (d, *J* = 13.4 Hz, 1H), 1.32 (s, 3H).

^{13}C NMR (126 MHz, DMSO-d_6) δ 202.8, 190.5, 164.0, 157.6, 155.9, 155.0, 151.1, 137.5, 133.6, 129.9, 129.2, 128.3, 127.7, 126.8, 121.0, 120.8, 116.4, 105.6, 96.5, 62.5, 44.2, 23.7.

HRMS ESI: $[M+Na]^+$, Calcd for $C_{24}H_{16}BrNNaO_4$ 484.0155; found 484.0139.

(*R*)-7-amino-10-benzyl-4,10-dimethylindeno[2,1-*c*]chromene-6,9,11(10*H*)-trione (3k):

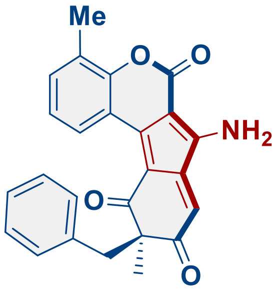

3k

The title compound was prepared by using the general reaction procedure and purified directly by silica gel column chromatography (eluent: EtOAc/Petroleum ether = 35/65, v/v); deep blue solid **3k** (21.9 mg, 55% yield) was obtained. The *er* of the **3k** was determined to be 97:3 [determined by HPLC, Chiralpak ADH, hexane: isopropanol = 70:30, 1 mL/min, λ = 260 nm, t (major) = 4.5 min, t (minor) = 5.6 min]. $[\alpha]^{29}_{D}$ = –235.5° (c = 0.09, $CHCl_3$).

^{1}H NMR (500 MHz, DMSO-*d_6*) δ 10.36 (s, 1H), 9.68 (d, *J* = 6.3 Hz, 1H), 9.44 (s, 1H), 7.59 (d, *J* = 6.3 Hz, 1H), 7.24 (t, *J* = 7.8 Hz, 1H), 7.13 – 7.02 (m, 4H), 6.91 (dd, *J* = 7.2, 2.1 Hz, 2H), 3.22 (d, *J* = 13.4 Hz, 1H), 3.04 (d, *J* = 13.3 Hz, 1H), 2.33 (s, 3H), 1.31 (s, 3H).

^{13}C NMR (126 MHz, DMSO-*d_6*) δ 203.0, 190.2, 164.1, 158.2, 156.7, 153.8, 151.4, 137.6, 137.4, 130.2, 129.9, 128.3, 126.7, 126.5, 124.0, 120.4, 116.9, 105.3, 96.9, 62.5, 44.3, 23.8, 16.5.

HRMS ESI: $[M+H]^+$, Calcd for $C_{25}H_{20}NO_4$ 398.1392; found 398.1403.

(*R*)-7-amino-10-benzyl-4-fluoro-10-methylindeno[2,1-*c*]chromene-6,9,11(10*H*)-trione (3l):

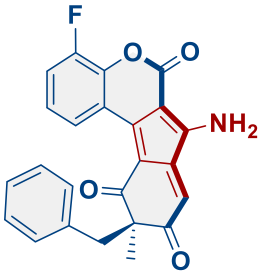

3l

The title compound was prepared by using the general reaction procedure and purified directly by silica gel column chromatography (eluent: EtOAc/Petroleum ether = 35/65, v/v); light yellow solid **3l** (20 mg, 50% yield) was obtained. The *er* of the **3l** was determined to be 97:3 [determined by HPLC, Chiralpak ADH, hexane: isopropanol = 70:30, 1 mL/min, λ = 260 nm, t (major) = 4.7 min, t (minor) = 6.1 min]. $[\alpha]^{29}_{D}$ = –34.4° (c = 0.059, $CHCl_3$).

^{1}H NMR (400 MHz, DMSO-*d_6*) δ 10.42 (s, 1H), 9.58 (d, *J* = 8.3 Hz, 1H), 9.51 (s, 1H), 7.67 (t, *J* = 9.4 Hz, 1H), 7.38 – 7.28 (m, 1H), 7.14 (s, 1H), 7.13 – 7.04 (m, 3H), 6.90 (dd, *J* = 7.4, 2.2 Hz, 2H), 3.21 (d, *J* = 13.4 Hz, 1H), 3.04 (d, *J* = 13.3 Hz, 1H), 1.31 (s, 3H)..

^{13}C NMR (126 MHz, DMSO-*d_6*) δ 202.7, 190.5, 164.1, 157.1, 154.8, 150.9, 149.0, 143.8, 137.5, 129.9, 128.3, 127.6, 126.8, 124.3, 122.1, 121.9, 121.4, 119.0, 105.9, 96.3, 62.6, 44.3, 23.5.

HRMS ESI: $[M+H]^+$, Calcd for $C_{24}H_{17}FNO_4$ 402.1142; found 402.1150.

(*R*)-7-amino-10-benzyl-4-chloro-10-methylindeno[2,1-*c*]chromene-6,9,11(10*H*)-trione 3n:

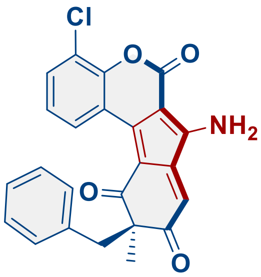

3m

The title compound was prepared by using the general reaction procedure and purified directly by silica gel column chromatography (eluent: EtOAc/Petroleum ether = 35/65, v/v); blue deep solid **3m** (28.4 mg, 68% yield) was obtained. The *er* of the **3m** was determined to be 96:4 [determined by HPLC, Chiralpak ADH, hexane: isopropanol = 70:30, 1 mL/min, λ = 260 nm, t (major) = 5.1 min, t (minor) = 6.7 min]. $[\alpha]^{29}_{D}$ = +26.1° (c = 0.22, $CHCl_3$).

^{1}H NMR (500 MHz, DMSO-d_6) δ 10.44 (s, 1H), 9.77 (d, *J* = 8.2 Hz, 1H), 9.52 (s, 1H), 7.84 (d, *J* = 7.9 Hz, 1H), 7.33 (t, *J* = 8.0 Hz, 1H), 7.13 (s, 1H), 7.12 – 7.05 (m, 3H), 6.91 (d, *J* = 2.4 Hz, 2H), 3.20 (d, *J* = 13.4 Hz, 1H), 3.04 (d, *J* = 13.4 Hz, 1H), 1.31 (s, 3H).

^{13}C NMR (126 MHz, DMSO-d_6) δ 202.7, 190.4, 164.1, 157.1, 155.1, 151.0, 137.4, 135.9, 131.1, 129.9, 128.3, 126.8, 124.9, 121.4, 121.3, 118.8, 105.9, 96.5, 62.6, 44.4, 23.4.

HRMS ESI: $[M+H]^+$, Calcd for $C_{24}H_{17}ClNO_4$ 418.0846; found 418.0840.

(*R*)-7-amino-10-benzyl-4-bromo-10-methylindeno[2,1-*c*]chromene-6,9,11(10*H*)-trione

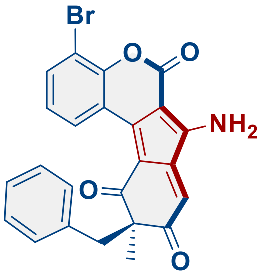

3n

(3n): The title compound was prepared by using the general reaction procedure and purified directly by silica gel column chromatography (eluent: EtOAc/Petroleum ether = 35/65, v/v); deep blue solid **3n** (21.7 mg, 47% yield) was obtained. The *er* of the **3n** was determined to be 97:3 [determined by HPLC, Chiralpak ADH, hexane: isopropanol = 70:30, 1 mL/min, λ = 260 nm, t (major) = 5.2 min, t (minor) = 6.7 min]. $[\alpha]^{30}_{D}$ = –315.9° (c = 0.097, $CHCl_3$).

^{1}H NMR (500 MHz, DMSO-d_6) δ 10.45 (s, 1H), 9.85 (d, *J* = 8.1 Hz, 1H), 9.54 (s, 1H), 8.01 (d, *J* = 7.8 Hz, 1H), 7.31 (t, *J* = 8.0 Hz, 1H), 7.14 (s, 1H), 7.12 – 7.06 (m, 3H), 6.91 (d, *J* = 7.2 Hz, 2H), 3.22 (d, *J* = 13.4 Hz, 1H), 3.06 (d, *J* = 13.4 Hz, 1H), 1.32 (s, 3H).

^{13}C NMR (126 MHz, DMSO-d_6) δ 202.7, 190.5, 164.1, 157.9, 155.3, 152.1, 151.1, 139.1, 137.5, 131.7, 129.9, 128.3, 126.8, 125.5, 121.3, 118.9, 110.8, 105.9, 96.7, 62.6, 44.8, 23.5.

HRMS ESI: $[M+H]^+$, Calcd for $C_{24}H_{17}BrNO_4$ 462.0341; found 462.0343.

(*R*)- 7-amino-10-benzyl-2,4,10-trimethylindeno[2,1-c]chromene-6,9,11(10H)-trione (3o):

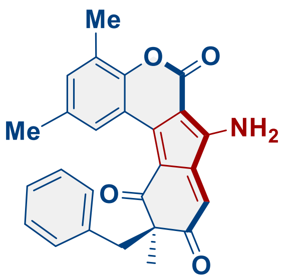

3o

The title compound was prepared by using the general reaction procedure and purified directly by silica gel column chromatography (eluent: EtOAc/Petroleum ether = 35/65, v/v); green solid **3o** (26.3 mg, 64% yield) was obtained. The *er* of the **3o** was determined to be 97:3 [determined by HPLC, Chiralpak ADH, hexane: isopropanol = 70:30, 1 mL/min, λ = 260 nm, t (major) = 4.0 min, t (minor) = 4.7 min]. $[\alpha]^{29}_{D}$ = –25.1° (c = 0.087, $CHCl_3$)

^{1}H NMR (400 MHz, $CDCl_3$) δ 9.38 (d, *J* = 2.3 Hz, 1H), 8.45 (s, 1H), 8.06 (s, 1H), 7.26 (s, 1H), 7.18 – 7.01 (m, 3H), 6.96 (dd, *J* = 6.7, 2.9 Hz, 2H), 6.77 (s, 1H), 3.29 (d, *J* = 13.3 Hz, 1H), 3.11 (d, *J* = 13.3 Hz, 1H), 2.36 (s, 3H), 2.31 (s, 3H), 1.46 (s, 3H).

^{13}C NMR (126 MHz, $CDCl_3$) δ 204.0, 190.7, 163.0, 160.2, 156.7, 152.0, 139.1, 136.7, 134.1, 130.3, 130.0, 128.2, 126.9, 126.6, 119.9, 116.8, 106.2, 98.9, 63.5, 22.1, 21.1, 16.5.

HRMS ESI: $[M+Na]^+$, Calcd for $C_{26}H_{21}NNaO_4$ 434.1363; found 434.1345.

(*R*)-7-amino-10-benzyl-2,4-floro-10-methylindeno[2,1-c]chromene-6,9,11(10H)-trione (3p):

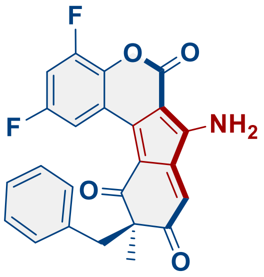

3p

The title compound was prepared by using the general reaction procedure and purified directly by silica gel column chromatography (eluent: EtOAc/Petroleum ether = 35/65, v/v); deep blue solid **3p** (22.6 mg, 57% yield) was obtained. The *er* of the **3p** was determined to be 95:5 [determined by HPLC, Chiralpak ADH, hexane: isopropanol = 70:30, 1 mL/min, λ = 260 nm, t (major) = 5.0 min, t (minor) = 7.3 min]. $[\alpha]^{31}_{D}$ = –20.0° (c = 0.10, $CHCl_3$).

^{1}H NMR (400 MHz, DMSO-*d*$_6$) δ 10.49 (s, 1H), 9.57 (s, 1H), 9.55-9.49 (m, 1H), 7.88 – 7.78 (m, 1H), 7.17 (s, 1H), 7.15 – 7.04 (m, 3H), 6.90 – 6.85 (m, 2H), 3.21 (d, *J* = 13.3 Hz, 1H), 3.04 (d, *J* = 13.3 Hz, 1H), 1.32 (s, 3H).

^{13}C NMR (101 MHz, DMSO-*d*$_6$) δ 203.2, 190.4, 164.1, 156.9, 154.1, 150.6, 141.0, 137.5, 130.0, 128.5, 127.0, 122.3, 119.1, 112.9, 112.6, 106.3, 96.5, 62.8, 44.6, 23.3.

HRMS ESI: $[M-H]^+$, Calcd for $C_{24}H_{14}F_2NO_4$ 418.0891; found 418.0894.

(*R*)-7-amino-10-benzyl-2,4-dichloro-10-methylindeno[2,1-c]chromene-6,9,11(10H)-trione (3q):

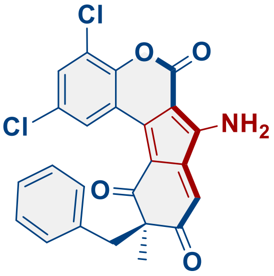

3q

The title compound was prepared by using the general reaction procedure and purified directly by silica gel column chromatography (eluent: EtOAc/Petroleum ether = 35/65, v/v); light yellow solid **3q** (21.5 mg, 49% yield) was obtained. The *er* of the **3q** was determined to be 94:6 [determined by HPLC, Chiralpak ADH, hexane: isopropanol = 70:30, 1 mL/min, λ = 260 nm, t (major) = 4.7 min, t (minor) = 6.3 min]. $[\alpha]^{29}_{D}$ = –224.0° (c = 0.10, $CHCl_3$).

^{1}H NMR (400 MHz, DMSO-d_6) δ 10.52 (s, 1H), 9.93 (d, *J* = 2.6 Hz, 1H), 9.60 (s, 1H), 8.04 (d, *J* = 2.5 Hz, 1H), 7.17 (s, 1H), 7.15 – 7.05 (m, 3H), 6.92 – 6.84 (m, 2H), 3.22 (d, *J* = 13.4 Hz, 1H), 3.04 (d, *J* = 13.4 Hz, 1H), 1.32 (s, 3H).

^{13}C NMR (101 MHz, DMSO) δ 2 02.5, 190.9, 164.0, 156.4, 153.9, 150.5, 150.1, 137.3, 134.8, 128.3, 128.1, 126.9, 122.7, 122.2, 119.6, 106.3, 96.5, 62.7, 44.4, 23.1.

HRMS ESI: $[M-H]^+$, Calcd for $C_{24}H_{14}Cl_2NO_4$ 450.0300; found 450.0303.

(*R*)-7-amino-10-benzyl-2,4-dibromo-10-methylindeno[2,1-c]chromene-6,9,11(10H)-trione (3r):

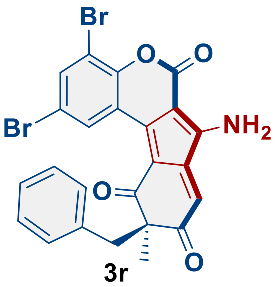

3r

The title compound was prepared by using the general reaction procedure and purified directly by silica gel column chromatography (eluent: EtOAc/Petroleum ether = 35/65, v/v); light yellow solid **3r** (21.5 mg, 49% yield) was obtained. The *er* of the **3r** was determined to be 90.5:9.5 [determined by HPLC, Chiralpak ADH, hexane: isopropanol = 70:30, 1 mL/min, λ = 260 nm, t (major) = 5.0 min, t (minor) = 6.3 min]. $[\alpha]^{29}_{D}$ = –117.1° (c = 0.07, $CHCl_3$)

^{1}H NMR (500 MHz, DMSO-d_6) δ 10.54 (s, 1H), 10.07 (d, *J* = 2.6 Hz, 1H), 9.62 (s, 1H), 8.23 (d, *J* = 2.6 Hz, 1H), 7.16 (s, 1H), 7.15 – 7.09 (m, 3H), 6.89 (d, *J* = 7.9 Hz, 2H), 3.21 (d, *J* = 13.6 Hz, 1H), 3.03 (d, *J* = 13.4 Hz, 1H), 1.32 (s, 3H).

^{13}C NMR (126 MHz, DMSO-d_6) δ 202.5, 190.8, 163.9, 156.8, 154.0, 151.4, 150.7, 140.7, 137.3, 133.7, 129.9, 128.8, 126.9, 121.9, 119.9, 116.2, 112.2, 106.1, 96.6, 62.7, 44.4, 22.9.

HRMS ESI: $[M-H]^+$, Calcd for $C_{24}H_{14}Br_2NO_4$ 537.9290; found 537.9281.

(*R*)-7-amino-10-benzyl-2-chloro-3,10-dimethylindeno[2,1-c]chromene-6,9,11(10H)-trione (3s): The title compound was prepared by using the general reaction procedure and purified directly by silica gel column chromatography (eluent: EtOAc/Petroleum ether = 35/65, v/v); deep blue solid **3s** (28.1 mg, 65% yield) was obtained. The *er* of the **3s** was determined to be 95:5 [determined by HPLC, Chiralpak ADH, hexane: isopropanol = 70:30, 1 mL/min, λ = 260 nm, t (major) = 5.1 min, t (minor) = 7.3 min]. $[\alpha]^{29}_D$ = –22.9° (c = 0.11, $CHCl_3$).

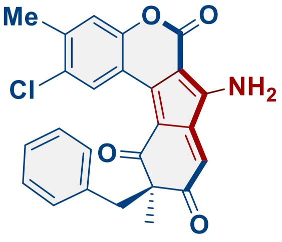

3s

^{1}**H NMR** (500 MHz, DMSO-d_6) δ 10.37 (s, 1H), 9.91 (s, 1H), 9.46 (s, 1H), 7.34 (s, 1H), 7.17 – 7.05 (m, 4H), 6.90 (d, *J* = 8.2 Hz, 2H), 3.24 (d, *J* = 13.4 Hz, 1H), 3.04 (d, *J* = 13.4 Hz, 1H), 2.44 (s, 3H), 1.33 (s, 3H).

^{13}**C NMR** (126 MHz, DMSO-d_6) δ 202.8, 190.6, 164.0, 157.9, 154.8, 154.2, 151.0, 144.6, 142.9, 131.3, 129.8, 129.0, 128.3, 126.8, 121.0, 120.1, 116.2, 105.3, 96.5, 62.4, 44.4, 23.5, 20.7.

HRMS ESI: $[M+H]^+$, Calcd for $C_{25}H_{19}ClNO_4$ 432.1003.; found 432.1012.

(*R*)-7-amino-10-benzyl-10-methylbenzo[h]indeno[2,1-c]chromene-6,9,11(10H)-trione (3t): The title compound was prepared by using the general reaction procedure and purified directly by silica gel column chromatography (eluent: EtOAc/Petroleum ether = 35/65, v/v); light yellow solid **3t** (29.7 mg, 71% yield) was obtained. The *er* of the **3t** was determined to be 98:2 [determined by HPLC, Chiralpak ADH, hexane: isopropanol = 80:20, 1 mL/min, λ = 260 nm, t (major) = 8.4 min, t (minor) = 11.9 min]. $[\alpha]^{29}_D$ = +29.8° (c = 0.11, $CHCl_3$).

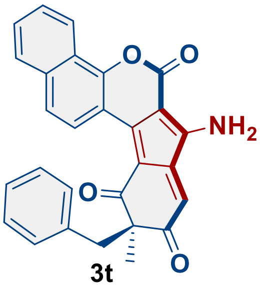

3t

^{1}**H NMR** (500 MHz, DMSO-d_6) δ 10.37 (s, 1H), 9.65 (d, *J* = 8.9 Hz, 1H), 9.47 (s, 1H), 8.31 (d, *J* = 8.4 Hz, 1H), 7.96 (d, *J* = 8.1 Hz, 1H), 7.78 – 7.69 (m, 2H), 7.67 – 7.61 (m, 1H), 7.16 – 7.09 (m, 3H), 7.08 (s, 1H), 6.95 (d, 2H), 3.24 (d, *J* = 13.4 Hz, 1H), 3.07 (d, *J* = 13.4 Hz, 1H), 1.34 (s, 3H).

^{13}**C NMR** (126 MHz, DMSO) δ 202.9, 190.3, 164.3, 157.7, 156.3, 153.0, 151.5, 137.7, 136.8, 130.4, 130.0, 128.3, 127.6, 127.1, 126.7, 123.7, 123.4, 123.2, 120.0, 112.8, 105.4, 96.8, 62.6, 44.2, 23.7.

HRMS ESI: $[M+H]^+$, Calcd for $C_{28}H_{20}NO_4$ 434.1392; found 434.1396.

(*R*)-7-amino-10-methyl-10-(4-methylbenzyl)indeno[2,1-c]chromene-6,9,11(10H)-trione

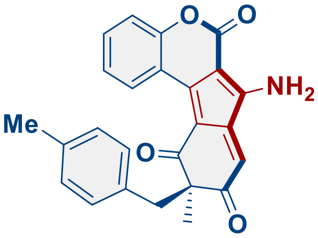

4a

(4a): The title compound was prepared by using the general reaction procedure and purified directly by silica gel column chromatography (eluent: EtOAc/Petroleum ether = 35/65, v/v); deep blue solid **4a** (31 mg, 80% yield) was obtained. The *er* of the **4a** was determined to be 97:3 [determined by HPLC, Chiralpak ADH, hexane: isopropanol = 80:20, 1 mL/min, λ = 260 nm, t (major) = 5.9 min, t (minor) = 7.9 min]. $[\alpha]^{29}_{D}$ = –292.2° (c = 0.05, $CHCl_3$).

^{1}H NMR (500 MHz, DMSO-*d*$_6$) δ 10.32 (s, 1H), 9.78 (d, *J* = 8.1 Hz, 1H), 9.40 (s, 1H), 7.69 (t, *J* = 7.7 Hz, 1H), 7.34 (t, *J* = 7.7 Hz, 1H), 7.27 (d, *J* = 7.0 Hz, 1H), 7.09 (s, 1H), 6.88 (d, *J* = 8.1 Hz, 2H), 6.78 (d, *J* = 8.1 Hz, 2H), 3.16 (d, *J* = 13.3 Hz, 1H), 2.98 (d, *J* = 13.3 Hz, 1H), 2.13 (s, 3H), 1.29 (s, 3H).

^{13}C NMR (126 MHz, DMSO) δ 202.9, 190.3, 164.0, 158.2, 156.0, 155.6, 151.3, 136.2, 135.7, 134.5, 132.4, 129.8, 128.9, 124.6, 120.7, 117.9, 117.2, 105.4, 96.8, 62.6, 43.8, 23.6, 21.0.

HRMS ESI: $[M+H]^+$, Calcd for $C_{25}H_{20}NO_4$ 398.1392; found 398.1384.

(*R*)-7-amino-10-(4-hydroxybenzyl)-10-methylindeno[2,1-c]chromene-6,9,11(10H)-trione

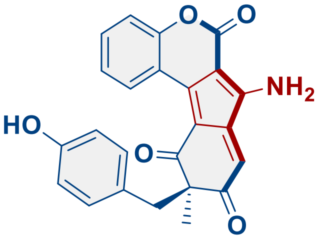

4b

(4b): The title compound was prepared by using the general reaction procedure and purified directly by silica gel column chromatography (eluent: EtOAc/Petroleum ether = 50/50, v/v); deep blue solid **4b** (32 mg, 80% yield) was obtained. The *er* of the **4b** was determined to be 98:2 [determined by HPLC, Chiralpak ADH, hexane: isopropanol = 90:10, 1 mL/min, λ = 260 nm, t (major) = 20.3 min, t (minor) = 23.7 min]. $[\alpha]^{22}_{D}$ = –110.8° (c = 0.04, $CHCl_3$).

^{1}H NMR (500 MHz, DMSO-*d*$_6$) δ 10.32 (s, 1H), 9.81 (d, *J* = 8.1 Hz, 1H), 9.47 (s, 1H), 9.15 (s, 1H), 7.72 (t, *J* = 6.9 Hz, 1H), 7.37 (t, *J* = 7.7 Hz, 1H), 7.31 (d, *J* = 8.2 Hz, 1H), 7.08 (s, 1H), 6.70 (d, *J* = 8.5 Hz, 2H), 6.48 (d, *J* = 8.5 Hz, 2H), 3.08 (d, *J* = 13.3 Hz, 1H), 2.92 (d, *J* = 13.3 Hz, 1H), 1.28 (s, 3H).

^{13}C NMR (126 MHz, DMSO-*d*$_6$) δ 203.2, 190.6, 164.0, 158.3, 156.2, 155.6, 151.2, 136.2, 132.4, 130.9, 127.5, 124.6, 120.8, 117.9, 117.2, 115.0, 105.7, 96.7, 62.8, 22.9.

HRMS ESI: $[M+H]^+$, Calcd for $C_{24}H_{18}NO_5$ 400.1185; found 400.1189.

(*R*)-7-amino-10-(4-methoxybenzyl)-10-methylindeno[2,1-c]chromene-6,9,11(10H)-trione

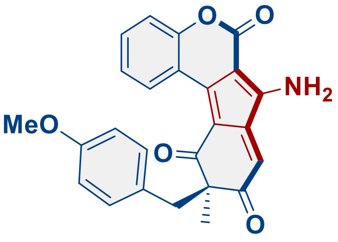

4c

(4c): The title compound was prepared by using the general reaction procedure and purified directly by silica gel column chromatography (eluent: EtOAc/Petroleum ether = 40/60, v/v); deep blue solid **4c** (33.5 mg, 81% yield) was obtained. The *er* of the **4c** was determined to be 97:3 [determined by HPLC, Chiralpak ADH, hexane: isopropanol = 80:20, 1 mL/min, λ = 260 nm, t (major) = 7.9 min, t (minor) = 9.7 min]. $[\alpha]^{32}_D$ = –424.0° (c = 0.025, $CHCl_3$).

^{1}H NMR (500 MHz DMSO-*d*$_6$) δ 10.33 (s, 1H), 9.80 (d, *J* = 8.0 Hz, 1H), 9.42 (s, 1H), 7.71 (t, *J* = 7.6 Hz, 1H), 7.35 (t, *J* = 7.6 Hz, 1H), 7.29 (d, *J* = 8.3 Hz, 1H), 7.08 (s, 1H), 6.82 (d, *J* = 8.2 Hz, 2H), 6.65 (d, *J* = 8.2 Hz, 2H), 3.61 (s, 3H), 3.14 (d, *J* = 13.4 Hz, 1H), 2.96 (d, *J* = 13.4 Hz, 1H), 1.29 (s, 3H).

^{13}C NMR (126 MHz, DMSO) δ 203.1, 190.4, 164.0, 158.3, 158.2, 155.9, 155.6, 151.3, 136.2, 132.4, 131.0, 129.4, 124.6, 120.7, 117.9, 117.2, 113.7, 105.5, 96.8, 62.8, 55.3, 43.6, 23.3.

HRMS ESI: $[M+H]^+$, Calcd for $C_{25}H_{20}NO_5$ 414.1341; found 414.1341.

(*R*)-10-(4-(allyloxy)benzyl)-7-amino-10-methylindeno[2,1-c]chromene-6,9,11(10H)-

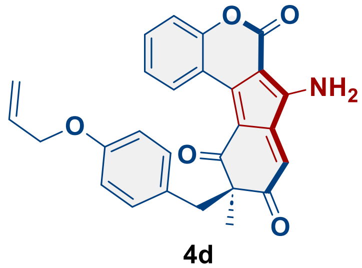

4d

trione (4d): The title compound was prepared by using the general reaction procedure and purified directly by silica gel column chromatography (eluent: EtOAc/Petroleum ether = 35/65, v/v); light yellow solid **4d** (24.6 mg, 56% yield) was obtained. The *er* of the **4d** was determined to be 97:3 [determined by HPLC, Chiralpak ADH, hexane: isopropanol = 80:20, 1 mL/min, λ = 260 nm, t (major) = 9.0 min, t (minor) = 11.2 min]. $[\alpha]^{29}_D$ = –324.6° (c = 0.13, $CHCl_3$).

^{1}H NMR (500 MHz $CDCl_3$) δ 9.87 (d, *J* = 7.9 Hz, 1H), 8.14 (s, 1H), 7.80 – 7.57 (m, 2H), 7.37 (t, *J* = 7.2 Hz, 1H), 7.28 (s, 1H), 6.90 (d, *J* = 8.5 Hz, 2H), 6.71 (d, *J* = 7.2 Hz, 1H), 6.64 (d, *J* = 8.5 Hz, 2H), 5.95 (m, 1H), 5.31 (d, *J* = 15.6 Hz, 1H), 5.21 (d, *J* = 9.8 Hz, 1H), 4.39 (d, *J* = 5.3 Hz, 2H), 3.33 (d, *J* = 13.4 Hz, 1H), 3.11 (d, *J* = 13.3 Hz, 1H), 1.49 (s, 3H).

^{13}C NMR (126 MHz, $CDCl_3$) δ 203.6, 191.0, 162.1, 160.0, 157.8, 155.5, 154.7, 150.7, 136.1, 133.2, 133.0, 130.8, 129.0, 125.0, 120.6, 117.8, 117.6, 117.2, 114.2, 106.7, 98.9, 68.7, 63.3, 44.8, 22.8.

HRMS ESI: $[M+H]^+$, Calcd for $C_{27}H_{22}NO_5$ 440.1498; found 440.1482.

(*R*)-7-amino-10-methyl-10-(4-(prop-2-yn-1-yloxy)benzyl)indeno[2,1-c]chromene-6,9,11(10H)-trione (4e): The title compound was prepared by using the general reaction procedure and purified directly by silica gel column chromatography (eluent: EtOAc/Petroleum ether = 35/65, v/v);deep blue solid **4e** (29.7 mg, 68% yield) was obtained. The *er* of the **4e** was determined to be 94.5:5.5 [determined by HPLC, Chiralpak ADH, hexane: isopropanol = 80:20, 1 mL/min, λ = 260 nm, t (major) = 6.9 min, t (minor) = 9.2 min]. $[\alpha]^{29}_{D}$ = –371.2° (c = 0.11, $CHCl_3$).

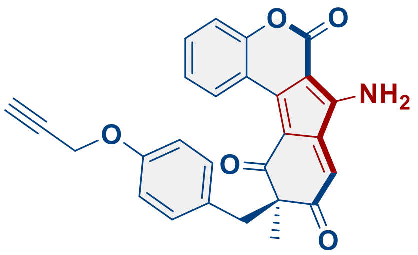

4e

^{1}H NMR (500 MHz $CDCl_3$) δ 9.79 (d, *J* = 6.4 Hz, 1H), 8.25 (s, 1H), 8.17 (s, 1H), 7.62 (t, *J* = 7.8 Hz, 1H), 7.32 (t, *J* = 7.0 Hz, 1H), 7.23 (d, *J* = 9.6 Hz, 1H), 6.92 (d, *J* = 8.9 Hz, 2H), 6.81 (s, 1H), 6.69 (d, *J* = 8.9 Hz, 2H), 4.54 (d, *J* = 2.4 Hz, 2H), 3.29 (d, *J* = 13.4 Hz, 1H), 3.10 (d, *J* = 13.4 Hz, 1H), 2.44 (t, *J* = 2.4 Hz, 1H), 1.47 (s, 3H).

^{13}C NMR (126 MHz, $CDCl_3$) δ 203.9, 191.0, 162.8, 160.2, 156.4, 155.5, 150.9, 136.2, 133.0, 131.0, 129.8, 125.1, 120.7, 117.9, 117.2, 114.5, 106.6, 98.6, 63.5, 55.8, 44.4, 22.6.

HRMS ESI: $[M+H]^+$, Calcd for $C_{27}H_{19}NaNO_5$ 460.1161; found 460.1165.

(*R*)-7-amino-10-(4-florobenzyl)-10-methylindeno[2,1-c]chromene-6,9,11(10H)-trione (4f): The title compound was prepared by using the general reaction procedure and purified directly by silica gel column chromatography (eluent: EtOAc/Petroleum ether = 35/65, v/v); deep blue solid **4f** (34.9 mg, 87% yield) was obtained. The *er* of the **4f** was determined to be 96:4 [determined by HPLC, Chiralpak ADH, hexane: isopropanol = 80:20, 1 mL/min, λ = 260 nm, t (major) = 6.3 min, t (minor) = 8. min]. $[\alpha]^{29}_{D}$ = –122.8° (c = 0.12, $CHCl_3$).

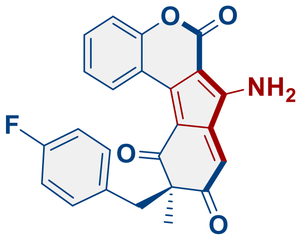

4f

^{1}H NMR (500 MHz, DMSO-*d*$_6$) δ 10.34 (s, 1H), 9.77 (d, *J* = 7.9 Hz, 1H), 9.40 (s, 1H), 7.67 (t, *J* = 7.5 Hz, 1H), 7.31 (t, *J* = 7.5 Hz, 1H), 7.24 (d, *J* = 8.2 Hz, 1H), 7.07 (s, 1H), 6.98 – 6.81 (m, 4H), 3.19 (d, *J* = 13.2 Hz, 1H), 3.01 (d, *J* = 13.3 Hz, 1H), 1.30 (s, 3H).

^{13}C NMR (126 MHz, DMSO-*d*$_6$) δ 202.9, 190.0, 164.1, 162.2, 160.3, 158.2, 156.2, 155.6, 151.3, 136.1, 133.8, 132.4, 131.8, 131.7, 124.6, 120.5, 117.8, 117.1, 115.0, 114.9, 105.3, 96.9, 62.6, 43.1, 23.7.

HRMS ESI: $[M+H]^+$, Calcd for $C_{24}H_{17}FNO_4$ 402.1142; found 402.1138.

(*R*)-7-amino-10-(4-bromobenzyl)-10-methylindeno[2,1-c]chromene-6,9,11(10H)-trione

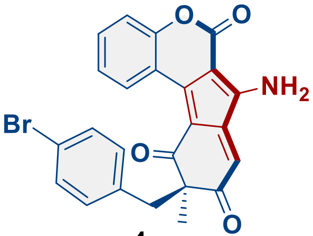

4g

(4g): The title compound was prepared by using the general reaction procedure and purified directly by silica gel column chromatography (eluent: EtOAc/Petroleum ether = 35/65, v/v); deep blue solid **4g** (37 mg, 80% yield) was obtained. The *er* of the **4g** was determined to be 96:4 [determined by HPLC, Chiralpak ADH, hexane: isopropanol = 80:20, 1 mL/min, λ = 260 nm, t (major) = 6.8 min, t (minor) = 8.2 min]. $[\alpha]^{29}_{D}$ = –259.4° (c = 0.062, $CHCl_3$).

^{1}H NMR (500 MHz, DMSO-*d_6*) δ 10.38 (s, 1H), 9.77 (d, *J* = 7.9 Hz, 1H), 9.45 (s, 1H), 7.69 (t, *J* = 7.5 Hz, 1H), 7.33 (t, *J* = 7.6 Hz, 1H), 7.27 (t, *J* = 7.7 Hz, 3H), 7.10 (s, 1H), 6.87 (d, *J* = 8.1 Hz, 2H), 3.20 (d, *J* = 13.4 Hz, 1H), 3.02 (d, *J* = 13.4 Hz, 1H), 1.31 (s, 3H).

^{13}C NMR (126 MHz, DMSO-*d_6*) δ 202.7, 189.9, 164.1, 158.2, 156.5, 155.6, 151.4, 137.2, 136.2, 132.4, 132.2, 131.2, 124.6, 120.4, 120.0, 117.8, 117.1, 105.1, 97.0, 62.5, 42.7, 24.3.

HRMS ESI: $[M+H]^+$, Calcd for $C_{24}H_{17}BrNO_4$ 462.0341; found 462.0340.

(*R*)-7-amino-10-methyl-10-(4-(trifluoromethyl)benzyl)indeno[2,1-c]chromene-

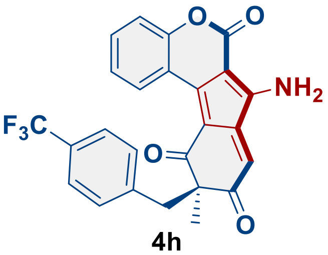

4h

6,9,11(10H)-trione (4h): The title compound was prepared by using the general reaction procedure and purified directly by silica gel column chromatography (eluent: EtOAc/Petroleum ether = 35/65, v/v); deep blue solid **4h** (28.9 mg, 64% yield) was obtained. The *er* of the **4h** was determined to be 92:8 [determined by HPLC, Chiralpak ADH, hexane: isopropanol = 95:5, 1 mL/min, λ = 260 nm, t (major) = 23.4 min, t (minor) = 26.3 min]. $[\alpha]^{29}_{D}$ = –154.1° (c = 0.12, $CHCl_3$).

^{1}H NMR (500 MHz, DMSO-*d_6*) δ 10.42 (s, 1H), 9.78 (d, *J* = 8.1 Hz, 1H), 9.49 (s, 1H), 7.70 (t, *J* = 8.4 Hz, 1H), 7.47 (d, *J* = 8.1 Hz, 2H), 7.33 (t, *J* = 7.7 Hz, 1H), 7.28 (d, *J* = 8.2 Hz, 1H), 7.15 (d, *J* = 8.1 Hz, 2H), 7.13 (s, 1H), 3.33 (d, *J* = 13.6 Hz, 1H), 3.16 (d, *J* = 13.6 Hz, 1H), 1.35 (s, 3H).

^{13}C NMR (126 MHz, DMSO-*d_6*) δ 202.5, 189.7, 164.2, 158.2, 156.8, 155.7, 151.5, 142.9, 136.3, 132.5, 130.7, 125.8, 125.1, 125.1, 124.6, 123.6, 120.4, 117.9, 117.1, 105.0, 97.0, 62.5, 42.5, 24.8.

HRMS ESI: $[M+H]^+$, Calcd for $C_{25}H_{17}F_3NO_4$ 452.1110; found 452.1113.

(*R*)-7-amino-10-methyl-10-(4-nitrobenzyl)indeno[2,1-c]chromene-6,9,11(10H)-trione

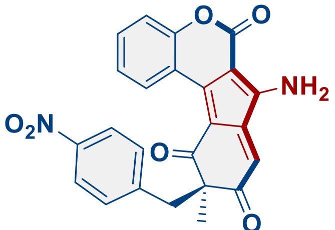

4i

(4i): The title compound was prepared by using the general reaction procedure and purified directly by silica gel column chromatography (eluent: EtOAc/Petroleum ether = 40/60, v/v); deep blue solid **4i** (30 mg, 70% yield) was obtained. The *er* of the **4i** was determined to be 97:3 [determined by HPLC, Chiralpak ADH, hexane: isopropanol = 80:20, 1 mL/min, λ = 260 nm, t (major) = 10.1 min, t (minor) = 12.1 min]. $[\alpha]^{29}_{D}$ = –622.9° (c = 0.035, $CHCl_3$).

^{1}H NMR (500 MHz, DMSO-d_6) δ 10.47 (s, 1H), 9.80 (d, *J* = 8.1 Hz, 1H), 9.56 (s, 1H), 8.01 (d, *J* = 8.7 Hz, 2H), 7.73 (t, *J* = 7.4 Hz, 1H), 7.37 (t, *J* = 7.3 Hz, 1H), 7.32 (d, *J* = 8.2 Hz, 1H), 7.21 (d, *J* = 8.7 Hz, 2H), 7.15 (s, 1H), 3.41 (d, *J* = 13.7 Hz, 1H), 3.23 (d, *J* = 13.7 Hz, 1H), 1.37 (s, 3H).

^{13}C NMR (126 MHz, DMSO-d_6) δ 202.5, 189.5, 164.4, 158.2, 157.1, 155.7, 151.6, 146.5, 146.4, 136.4, 132.5, 131.1, 124.7, 123.5, 120.2, 117.9, 117.1, 104.9, 97.1, 62.6, 42.0, 25.4.

HRMS ESI: $[M-H]^+$, Calcd for $C_{24}H_{15}N_2O_6$ 427.0930; found 427.0938.

(*R*)-7-amino-10-methyl-10-(2-nitrobenzyl)indeno[2,1-c]chromene-6,9,11(10H)-trione

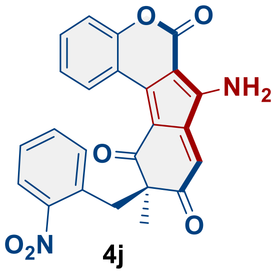

4j

(4j): The title compound was prepared by using the general reaction procedure and purified directly by silica gel column chromatography (eluent: EtOAc/Petroleum ether = 40/60, v/v); deep blue solid **4j** (30.8 mg, 72% yield) was obtained. The *er* of the **4j** was determined to be 97:3 [determined by HPLC, Chiralpak ADH, hexane: isopropanol = 80:20, 1 mL/min, λ = 260 nm, t (major) = 11.7 min, t (minor) = 16.5 min]. $[\alpha]^{29}_{D}$ = –85.0° (c = 0.13, $CHCl_3$).

^{1}H NMR (500 MHz, DMSO-d_6) δ 10.52 (s, 1H), 9.73 (d, *J* = 9.4 Hz, 1H), 9.58 (s, 1H), 7.81 (d, *J* = 8.0 Hz, 1H), 7.72 (t, *J* = 8.5 Hz, 1H), 7.46 (t, *J* = 7.5 Hz, 1H), 7.41 – 7.28 (m, 3H), 7.18 (s, 1H), 6.96 (d, *J* = 7.5 Hz, 1H), 3.62 (d, *J* = 15.1 Hz, 1H), 3.42 (d, *J* = 15.1 Hz, 1H), 1.32 (s, 3H).

^{13}C NMR (126 MHz, DMSO-d_6) δ 202.3, 189.4, 164.4, 158.2, 157.3, 155.7, 151.4, 150.5, 136.4, 133.1, 132.6, 132.0, 131.5, 128.1, 124.8, 124.6, 120.2, 117.9, 117.1, 104.7, 97.2, 61.7, 37.1, 25.3.

HRMS ESI: $[M+Na]^+$, Calcd for $C_{24}H_{16}N_2NaO_6$ 451.0906; found 451.0902.

(*R*)-2-((7-amino-10-methyl-6,9,11-trioxo-6,9,10,11-tetrahydroindeno[2,1-c]chromen-10-yl)methyl)benzonitrile (4k):

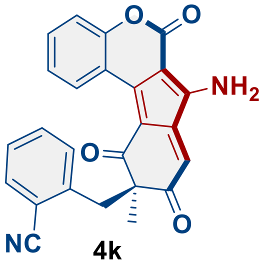

4k

The title compound was prepared by using the general reaction procedure and purified directly by silica gel column chromatography (eluent: EtOAc/Petroleum ether = 40/60, v/v); deep blue solid **4k** (28.9 mg, 70% yield) was obtained. The *er* of the **4k** was determined to be 95:5 [determined by HPLC, Chiralpak ADH, hexane: isopropanol = 90:10, 1 mL/min, λ = 260 nm, t (major) = 34.1 min, t (minor) = 47.2 min]. $[\alpha]^{28}_D$ = –640.0° (c = 0.05, $CHCl_3$).

^{1}H NMR (500 MHz, DMSO-*d_6*) δ 10.51 (s, 1H), 9.73 (d, *J* = 9.6 Hz, 1H), 9.57 (s, 1H), 7.75 – 7.67 (m, 2H), 7.46 (t, *J* = 7.1 Hz, 1H), 7.36 – 7.27 (m, 3H), 7.22 (s, 1H), 6.92 (d, *J* = 7.9 Hz, 1H), 3.45 (d, *J* = 14.8 Hz, 1H), 3.31 (d, *J* = 14.8 Hz, 1H), 1.38 (s, 3H).

^{13}C NMR (126 MHz, DMSO-*d_6*) δ 202.0, 189.3, 164.4, 158.2, 157.1, 155.7, 151.4, 141.4, 136.4, 133.3, 132.5, 129.7, 127.6, 124.7, 120.4, 118.3, 117.9, 117.1, 113.1, 104.9, 97.1, 62.0, 40.6, 24.2.

HRMS ESI: $[M+Na]^+$, Calcd for $C_{25}H_{16}N_2NaO_4$ 431.1008; found 431.1011.

(*R*)-7-amino-10-(3-methoxybenzyl)-10-methylindeno[2,1-c]chromene-6,9,11(10H)-trione (4l):

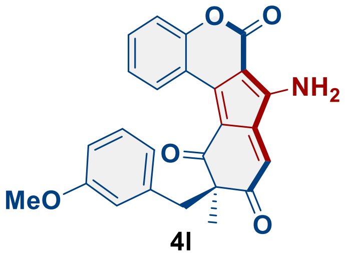

4l

The title compound was prepared by using the general reaction procedure and purified directly by silica gel column chromatography (eluent: EtOAc/Petroleum ether = 40/60, v/v); deep blue solid **4l** (22.7 mg, 55% yield) was obtained. The *er* of the **4l** was determined to be 96:4 [determined by HPLC, Chiralpak ADH, hexane: isopropanol = 80:20, 1 mL/min, λ = 260 nm, t (major) = 7.7 min, t (minor) = 11.9 min]. $[\alpha]^{29}_D$ = –170.0° (c = 0.10, $CHCl_3$).

^{1}H NMR (500 MHz, DMSO-*d_6*) δ 10.34 (s, 1H), 9.82 (d, *J* = 7.9 Hz, 1H), 9.43 (s, 1H), 7.71 (t, *J* = 7.5 Hz, 1H), 7.35 (t, *J* = 7.6 Hz, 1H), 7.30 (d, *J* = 8.2 Hz, 1H), 7.10 (s, 1H), 7.01 (t, *J* = 7.8 Hz, 1H), 6.64 (d, *J* = 6.7 Hz, 1H), 6.53 – 6.41 (m, 2H), 3.53 (s, 3H), 3.20 (d, *J* = 13.3 Hz, 1H), 3.02 (d, *J* = 13.3 Hz, 1H), 1.32 (s, 3H).

^{13}C NMR (126 MHz, DMSO-*d_6*) δ 203.0, 190.3, 164.0, 159.1, 158.2, 156.0, 155.6, 151.3, 139.1, 136.2, 132.4, 129.3, 124.6, 122.1, 120.7, 117.9, 117.1, 115.5, 112.2, 105.5, 96.8, 62.4, 55.1, 44.4, 23.8.

HRMS ESI: [M+H]$^+$, Calcd for $C_{25}H_{20}NO_5$ 414.1341; found 414.1336.

(*R*)-7-amino-10-(3-chlorobenzyl)-10-methylindeno[2,1-c]chromene-6,9,11(10H)-trione

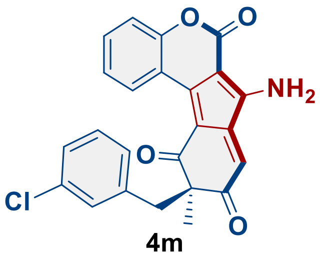

4m

(4m): The title compound was prepared by using the general reaction procedure and purified directly by silica gel column chromatography (eluent: EtOAc/Petroleum ether = 40/60, v/v); deep blue solid **4m** (29.7 mg, 71% yield) was obtained. The *er* of the **4m** was determined to be 96:4 [determined by HPLC, Chiralpak ADH, hexane: isopropanol = 80:20, 1 mL/min, λ = 260 nm, t (major) = 6.6 min, t (minor) = 9.2 min]. $[\alpha]^{29}_{D}$ = –284.4° (c = 0.11, $CHCl_3$).

^{1}H NMR (500 MHz, DMSO-*d*$_6$) δ 10.38 (s, 1H), 9.77 (d, *J* = 6.7 Hz, 1H), 9.47 (s, 1H), 7.71 (t, *J* = 7.0 Hz, 1H), 7.35 (t, *J* = 7.7 Hz, 1H), 7.29 (d, *J* = 8.2 Hz, 1H), 7.16 – 7.12 (m, 2H), 7.11 (s, 1H), 6.95 (d, *J* = 2.1 Hz, 1H), 6.90 – 6.85 (m, 1H), 3.20 (d, *J* = 13.4 Hz, 1H), 3.05 (d, *J* = 13.4 Hz, 1H), 1.31 (s, 3H).

^{13}C NMR (126 MHz, DMSO-*d*$_6$) δ 202.7, 189.8, 164.1, 158.2, 156.4, 155.6, 151.4, 140.1, 136.3, 132.8, 132.3, 130.1, 129.9, 128.6, 126.8, 124.7, 120.4, 117.9, 117.1, 105.3, 96.9, 62.6, 43.3, 23.7.

HRMS ESI: [M+Na]$^+$, Calcd for $C_{24}H_{17}ClNO_4$ 418.0846; found 418.0846.

(*R*)-7-amino-10-(2-bromo-3-fluorobenzyl)-10-methylindeno[2,1-c]chromene-

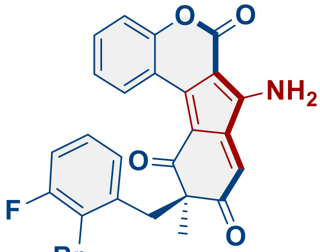

4n

6,9,11(10H)-trione (4n): The title compound was prepared by using the general reaction procedure and purified directly by silica gel column chromatography (eluent: EtOAc/Petroleum ether = 35/65, v/v); deep blue solid **4n** (22.4 mg, 45% yield) was obtained. The *er* of the **4n** was determined to be 89:11 [determined by HPLC, Chiralpak ADH, hexane: isopropanol = 90:10, 1 mL/min, λ = 260 nm, t (major) = 15.2 min, t (minor) = 17.6 min]. $[\alpha]^{29}_{D}$ = –245.6° (c = 0.11, $CHCl_3$).

^{1}H NMR (500 MHz, DMSO-*d*$_6$) δ 10.53 (s, 1H), 9.73 (d, *J* = 8.0 Hz, 1H), 9.58 (s, 1H), 7.69 (t, *J* = 7.7 Hz, 1H), 7.30 (t, *J* = 8.4 Hz, 2H), 7.26 (s, 1H), 7.20 – 7.08 (m, 2H), 6.50 (d, *J* = 7.5 Hz, 1H), 3.40 (d, *J* = 16.1 Hz, 1H), 3.25 (d, *J* = 15.6 Hz, 1H), 1.39 (s, 3H).

^{13}C NMR (126 MHz, DMSO-*d*$_6$) δ 202.0, 189.3, 164.5, 159.7, 158.2, 157.8, 157.1, 155.7, 151.3, 140.0, 136.3, 132.5, 129.0, 128.9, 125.6, 124.6, 120.5, 117.9, 117.1, 114.6, 114.5, 111.8, 111.6, 104.9, 97.1, 61.4, 41.4, 25.2.

HRMS ESI: $[M+H]^+$, Calcd for $C_{24}H_{16}BrFNO_4$ 480.0247; found 480.0261.

(*R*)-7-amino-10-(4-fluoro-3-methoxybenzyl)-10-methylindeno[2,1-*c*]chromene-6,9,11(10*H*)-trione (4o): The title compound was prepared by using the general reaction procedure and purified directly by silica gel column chromatography (eluent: EtOAc/Petroleum ether = 35/65, v/v); deep blue solid **4o** (28.5 mg, 66% yield) was obtained. The *er* of the **4o** was determined to be 96:4 [determined by HPLC, Chiralpak ADH, hexane: isopropanol = 80:20, 1 mL/min, λ = 260 nm, t (major) = 7.1 min, t (minor) = 9.5 min]. $[\alpha]^{29}_D$ = –297.8° (c = 0.092, $CHCl_3$).

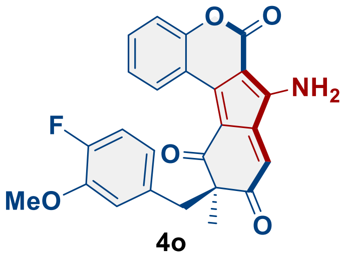

4o

^{1}H NMR (500 MHz, DMSO-d_6) δ 10.35 (s, 1H), 9.83 (d, *J* = 7.3 Hz, 1H), 9.45 (s, 1H), 7.72 (t, *J* = 7.2 Hz, 1H), 7.36 (t, *J* = 7.6 Hz, 1H), 7.30 (d, *J* = 8.2 Hz, 1H), 7.09 (s, 1H), 7.00 – 6.88 (m, 1H), 6.67 (d, *J* = 7.2 Hz, 1H), 6.47 – 6.36 (m, 1H), 3.54 (s, 3H), 3.17 (d, *J* = 13.3 Hz, 1H), 3.01 (d, *J* = 13.3 Hz, 1H), 1.32 (s, 3H).

^{13}C NMR (126 MHz, DMSO-d_6) δ 203.1, 190.2, 164.1, 158.2, 156.0, 155.6, 151.3, 149.7, 146.6, 146.5, 136.3, 134.2, 132.4, 124.6, 122.1, 122.0, 120.7, 117.9, 117.1, 115.6, 115.5, 105.6, 96.9, 62.4, 55.9, 44.4, 23.3.

HRMS ESI: $[M+H]^+$, Calcd for $C_{25}H_{19}FNO_5$ 432.1247; found 432.1244.

(*R*)-7-amino-10-methyl-10-(naphthalen-2-ylmethyl)indeno[2,1-c]chromene-6,9,11(10H)-trione (4p): The title compound was prepared by using the general reaction procedure and purified directly by silica gel column chromatography (eluent: EtOAc/Petroleum ether = 35/65, v/v); deep blue solid **4p** (24.3 mg, 56% yield) was obtained. The *er* of the **4p** was determined to be 96:4 [determined by HPLC, Chiralpak ADH, hexane: isopropanol = 70:30, 1 mL/min, λ = 260 nm, t (major) = 5.9 min, t (minor) = 6.9 min]. $[\alpha]^{27}_D$ = –144.0° (c = 0.12, $CHCl_3$).

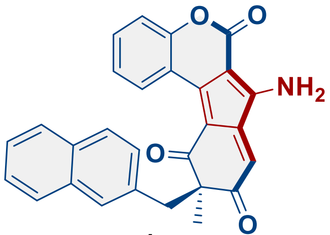

4p

^{1}H NMR (500 MHz, DMSO-d_6) δ 10.31 (s, 1H), 9.80 (d, *J* = 6.9 Hz, 1H), 9.40 (s, 1H), 7.78 – 7.68 (m, 2H), 7.66 – 7.58 (m, 2H), 7.41 (s, 1H), 7.39 – 7.32 (m, 3H), 7.29 (d, *J* = 8.2 Hz, 1H), 7.13 – 7.07 (m, 2H), 3.38 (d, *J* = 13.3,1H), 3.22 (d, *J* = 13.3 Hz, 1H), 1.36 (s, 3H).

^{13}C NMR (126 MHz, DMSO-d_6) δ 202.9, 190.2, 164.0, 158.2, 156.1, 155.6, 151.4, 136.2, 135.3, 133.0, 132.4, 132.1, 128.5, 128.5, 128.4, 127.8, 127.6, 126.4, 126.0, 124.7, 120.5, 117.9, 117.1, 105.4, 96.8, 62.8, 44.4, 23.4.

HRMS ESI: $[M+H]^+$, Calcd for $C_{28}H_{20}NO_4$ 434.1392; found 434.1386.

(*R*)-7-amino-10-methyl-10-(naphthalen-1-ylmethyl)indeno[2,1-c]chromene-6,9,11(10H)-trione (4q):

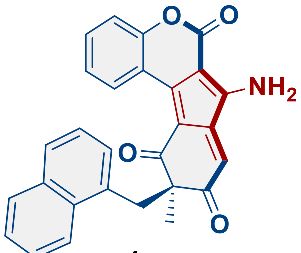

4q

The title compound was prepared by using the general reaction procedure and purified directly by silica gel column chromatography (eluent: EtOAc/Petroleum ether = 35/65, v/v); light yellow solid **4q** (21.7 mg, 50% yield) was obtained. The *er* of the **4q** was determined to be 96:4 [determined by HPLC, Chiralpak ADH, hexane: isopropanol = 80:20, 1 mL/min, λ = 260 nm, t (major) = 8.8 min, t (minor) = 11.4 min]. $[\alpha]^{28}_D$ = –769.5° (c = 0.077, $CHCl_3$).

^{1}H NMR (500 MHz, DMSO-d_6) δ 10.38 (s, 1H), 9.71 (d, *J* = 6.8 Hz, 1H), 9.47 (s, 1H), 8.08 (d, *J* = 9.1 Hz, 1H), 7.81 (d, *J* = 7.0 Hz, 1H), 7.70 (t, *J* = 7.0 Hz, 1H), 7.66 (d, *J* = 8.2 Hz, 1H), 7.44 – 7.35 (m, 2H), 7.33 – 7.25 (m, 2H), 7.25 – 7.18 (m, 1H), 7.13 (s, 1H), 6.79 (d, *J* = 7.1 Hz, 1H), 3.74 (d, *J* = 15.1 Hz, 1H), 3.59 (d, *J* = 15.1 Hz, 1H), 1.45 (s, 3H).

^{13}C NMR (126 MHz, DMSO-d_6) δ 202.9, 190.3, 164.2, 158.3, 156.3, 155.6, 151.3, 136.2, 133.9, 133.5, 132.6, 132.3, 128.8, 127.2, 126.5, 126.2, 125.9, 125.5, 124.5, 124.3, 120.8, 117.8, 117.1, 105.5, 96.9, 61.6, 40.6, 24.5.

HRMS ESI: $[M+H]^+$, Calcd for $C_{28}H_{20}NO_4$ 434.1392; found 434.1396.

(*R*)-7-amino-10-benzhydryl-10-methylindeno[2,1-c]chromene-6,9,11(10H)-trione (4r):

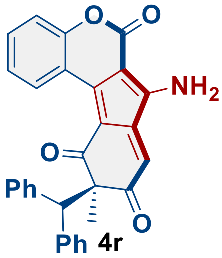

4r

The title compound was prepared by using the general reaction procedure and purified directly by silica gel column chromatography (eluent: EtOAc/Petroleum ether = 35/65, v/v); light yellow solid **4r** (23 mg, 50% yield) was obtained. The *er* of the **4r** was determined to be 99:1 [determined by HPLC, Chiralpak ADH, hexane: isopropanol = 95:5, 1 mL/min, λ = 260 nm, t (major) = 36.3 min, t (minor) = 41.3 min]. $[\alpha]^{30}_D$ = –300.0° (c = 0.083, $CHCl_3$).

^{1}H NMR (500 MHz, DMSO-d_6) δ 10.29 (s, 1H), 9.59 (d, *J* = 6.6 Hz, 1H), 9.40 (s, 1H), 7.72 (t, *J* = 8.7 Hz, 1H), 7.37 – 7.30 (m, 2H), 7.30 – 7.26 (m, 2H), 7.25 – 7.21 (m, 2H), 7.19 – 7.16 (m, 5H), 7.16 – 7.13 (m, 1H), 7.08 (s, 1H), 4.72 (s, 1H), 1.45 (s, 3H).

^{13}C NMR (126 MHz, DMSO-d_6) δ 202.7, 190.3, 163.9, 158.3, 155.6, 155.4, 150.6, 140.9, 140.7, 136.1, 132.1, 130.6, 130.2, 128.2, 128.1, 127.0, 126.9, 124.6, 121.6, 117.9, 117.1, 106.2, 96.7, 65.3, 59.9, 40.6, 21.0.

HRMS ESI: $[M+H]^+$, Calcd for $C_{30}H_{22}NO_4$ 460.1549; found 460.1547.

(*R*)-7-amino-10-methyl-10-propylindeno[2,1-c]chromene-6,9,11(10H)-trione (4s): The title compound was prepared by using the general reaction procedure and purified directly by silica gel column chromatography (eluent: EtOAc/Petroleum ether = 35/65, v/v); deep blue solid **4s** (29.2 mg, 87% yield) was obtained. The *er* of the **4s** was determined to be 92:8 [determined by HPLC, Chiralpak ADH, hexane: isopropanol = 90:10, 1 mL/min, λ = 260 nm, t (major) = 8.7 min, t (minor) = 10.7 min]. $[\alpha]^{32}_D$ = –20.0° (c = 0.1, $CHCl_3$).

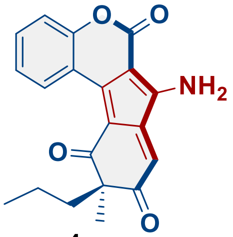

4s

^{1}H NMR (500 MHz, DMSO-d_6) δ 10.39 (s, 1H), 9.81 (d, *J* = 7.6 Hz, 1H), 9.44 (s, 1H), 7.70 (t, *J* = 7.6 Hz, 1H), 7.33 (t, *J* = 7.1 Hz, 1H), 7.28 (d, *J* = 7.8 Hz, 1H), 7.19 (s, 1H), 1.86 – 1.75 (m, 1H), 1.74 – 1.63 (m, 1H), 1.23 (s, 3H), 1.11 – 0.95 (m, 2H), 0.74 (t, *J* = 7.2 Hz, 3H).

^{13}C NMR (126 MHz, DMSO-d_6) δ 203.6, 191.0, 164.1, 158.3, 155.8, 155.6, 151.4, 136.1, 132.5, 124.6, 121.0, 117.9, 117.2, 105.2, 96.8, 61.4, 41.4, 23.2, 18.7, 14.8.

HRMS ESI: $[M+H]^+$, Calcd for $C_{20}H_{18}NO_4$ 336.1236; found 336.1231.

(*R*)- 10-allyl-7-amino-10-methylindeno[2,1-c]chromene-6,9,11(10H)-trione (4t): The title compound was prepared by using the general reaction procedure and purified directly by silica gel column chromatography (eluent: EtOAc/Petroleum ether = 35/65, v/v); light yellow solid **4t** (26 mg, 78% yield) was obtained. The *er* of the **4t** was determined to be 80:20 [determined by HPLC, Chiralpak ADH, hexane: isopropanol = 90:10, 1 mL/min, λ = 260 nm, t (major) = 9.9 min, t (minor) = 11.9 min]. $[\alpha]^{30}_D$ = +88.9° (c = 0.056, $CHCl_3$).

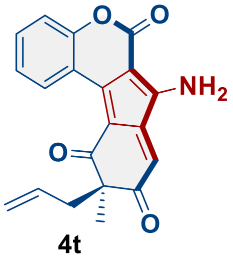

4t

^{1}H NMR (500 MHz, DMSO-d_6) δ 10.43 (s, 1H), 9.81 (d, *J* = 7.9 Hz, 1H), 9.50 (s, 1H), 7.72 (t, *J* = 7.4 Hz, 1H), 7.36 (t, *J* = 7.6 Hz, 1H), 7.31 (d, *J* = 8.2 Hz, 1H), 7.20 (s, 1H), 5.57 – 5.43 (m, 1H), 4.92 (s, 1H), 4.89 (d, *J* = 4.2 Hz, 1H), 2.60 (dd, *J* = 13.6, 6.8 Hz, 1H), 2.45 (dd, *J* = 13.6, 6.8 Hz, 1H), 1.25 (s, 3H).

^{13}C NMR (126 MHz, DMSO-d_6) δ 202.8, 190.3, 164.1, 158.3, 156.1, 155.6, 151.5, 136.2, 134.0, 132.4, 124.6, 120.7, 118.5, 117.9, 117.2, 105.1, 96.9, 61.2, 42.7, 22.8.

HRMS ESI: $[M+H]^+$, Calcd for $C_{20}H_{16}NO_4$ 334.1079; found 334.1074.

(*R*)-7-amino-10-cinnamyl-10-methylindeno[2,1-c]chromene-6,9,11(10H)-trione (4u): The

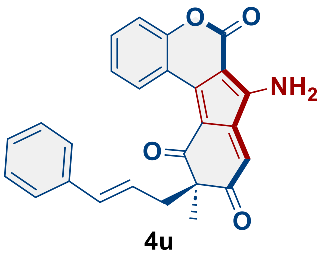

4u

title compound was prepared by using the general reaction procedure and purified directly by silica gel column chromatography (eluent: EtOAc/Petroleum ether = 35/65, v/v); deep blue solid **4u** (31.1 mg, 76% yield) was obtained. The *er* of the **4u** was determined to be 86:14 [determined by HPLC, Chiralpak ADH, hexane: isopropanol = 90:10, 1 mL/min, λ = 260 nm, t (major) = 12.5 min, t (minor) = 17.6 min]. $[\alpha]^{31}_{D}$ = –61.11° (c = 0.11, $CHCl_3$).

^{1}H NMR (500 MHz, DMSO-*d*$_6$) δ 10.41 (s, 1H), 9.81 (d, *J* = 6.6 Hz, 1H), 9.47 (s, 1H), 7.71 (t, *J* = 7.0 Hz, 1H), 7.34 (t, *J* = 7.2 Hz, 1H), 7.30 (d, *J* = 8.3 Hz, 1H), 7.23 (d, *J* = 4.3 Hz, 4H), 7.20 (s, 1H), 7.19 – 7.13 (m, 1H), 6.30 (d, *J* = 15.8 Hz, 1H), 6.01 – 5.91 (m, 1H), 2.75 (dd, *J* = 13.8, 6.9 Hz, 1H), 2.61 (dd, *J* = 13.6, 7.7 Hz, 1H), 1.30 (s, 3H).

^{13}C NMR (126 MHz, DMSO-*d*$_6$) δ 202.7, 190.3, 164.1, 158.3, 156.1, 155.6, 151.5, 137.2, 136.2, 133.1, 132.4, 129.0, 127.7, 126.4, 125.6, 124.6, 120.7, 117.9, 117.2, 105.1, 96.9, 61.9, 41.9, 22.5.

HRMS ESI: $[M+H]^+$, Calcd for $C_{26}H_{20}NO_4$ 410.1392; found 410.1390.

(*R*)-7-amino-10-methyl-10-(prop-2-yn-1-yl)indeno[2,1-c]chromene-6,9,11(10H)-trione

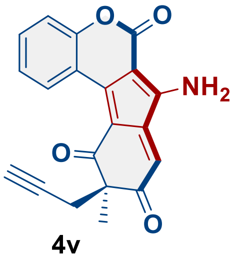

4v

(4v): The title compound was prepared by using the general reaction procedure and purified directly by silica gel column chromatography (eluent: EtOAc/Petroleum ether = 35/65, v/v); deep blue solid **4v** (26.5 mg, 80% yield) was obtained. The *er* of the **4v** was determined to be 80:20 [determined by HPLC, Chiralpak ADH, hexane: isopropanol = 90:10, 1 mL/min, λ = 260 nm, t (major) = 20.5 min, t (minor) = 25.2 min]. $[\alpha]^{29}_{D}$ = –52.8° (c = 0.14, $CHCl_3$).

^{1}H NMR (500 MHz, DMSO-*d*$_6$) δ 10.51 (s, 1H), 9.84 (d, *J* = 8.1 Hz, 1H), 9.57 (s, 1H), 7.74 (t, *J* = 7.7 Hz, 1H), 7.38 (t, *J* = 7.6 Hz, 1H), 7.33 (d, *J* = 8.2 Hz, 1H), 7.27 (s, 1H), 2.76 (dd, *J* = 16.1, 2.4 Hz, 1H), 2.67 (t, *J* = 2.2 Hz, 1H), 2.58 (dd, *J* = 16.1, 2.5 Hz, 1H), 1.26 (s, 3H).

^{13}C NMR (126 MHz, DMSO-*d*$_6$) δ 201.9, 189.2, 164.2, 158.3, 156.7, 155.7, 151.9, 136.4, 132.5, 124.7, 120.5, 117.9, 117.1, 104.6, 97.1, 81.7, 72.9, 60.8, 25.6, 24.8

HRMS ESI: $[M+H]^+$, Calcd for $C_{20}H_{14}NO_4$ 332.0923; found 332.0920.

methyl (*R*)-2-(7-amino-10-methyl-6,9,11-trioxo-6,9,10,11-tetrahydroindeno[2,1-c]chromen-10-yl)acetate (4w): The title compound was prepared by using the general reaction procedure and purified directly by silica gel column chromatography (eluent: EtOAc/Petroleum ether = 35/65, v/v); deep blue solid **4w** (14.6 mg, 40% yield) was obtained. The *er* of the **4w** was determined to be 92:8 [determined by HPLC, Chiralpak ADH, hexane: isopropanol = 70:30, 1 mL/min, λ = 260 nm, t (major) = 6.9 min, t (minor) = 14.9 min]. $[\alpha]^{29}_{D}$ = –26.0° (c = 0.10, $CHCl_3$).

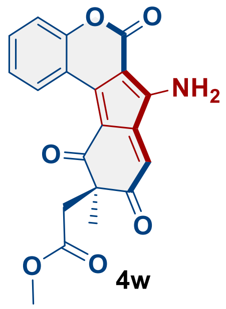

4w

¹H NMR (500 MHz, DMSO-d_6) δ 10.53 (s, 1H), 9.74 (d, *J* = 8.0 Hz, 1H), 9.60 (s, 1H), 7.72 (t, *J* = 7.5 Hz, 1H), 7.36 – 7.30 (m, 2H), 7.26 (s, 1H), 3.49 (s, 3H), 3.17 (d, *J* = 17.4 Hz, 1H), 2.99 (d, *J* = 17.3 Hz, 1H), 1.22 (s, 3H).

¹³C NMR (126 MHz, DMSO-d_6) δ 202.2, 190.2, 172.2, 164.3, 158.3, 156.6, 155.6, 151.4, 136.3, 132.5, 124.6, 120.2, 117.9, 117.1, 104.4, 96.8, 58.7, 52.0, 38.7, 25.9.

HRMS ESI: $[M+H]^+$, Calcd for $C_{20}H_{16}NO_6$ 366.0978; found 366.0971.

(*R*)-7-amino-10-methyl-10-(thiophen-2-ylmethyl)indeno[2,1-c]chromene-6,9,11(10H)-trione (4x): The title compound was prepared by using the general reaction procedure and purified directly by silica gel column chromatography (eluent: EtOAc/Petroleum ether = 35/65, v/v); deep blue solid **4x** (23.4 mg, 60% yield) was obtained. The *er* of the **4x** was determined to be 98:2 [determined by HPLC, Chiralpak ADH, hexane: isopropanol = 80:20, 1 mL/min, λ = 260 nm, t (major) = 7.6 min, t (minor) = 13.3 min]. $[\alpha]^{29}_{D}$ = –235.9° (c = 0.064, $CHCl_3$).

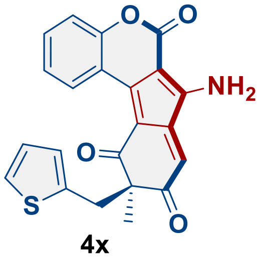

4x

¹H NMR (500 MHz, DMSO-d_6) δ 10.43 (s, 1H), 9.86 (d, *J* = 7.2 Hz, 1H), 9.51 (s, 1H), 7.72 (t, *J* = 7.2 Hz, 1H), 7.38 (t, *J* = 7.6 Hz, 1H), 7.31 (d, *J* = 8.2 Hz, 1H), 7.16 (d, *J* = 4.6 Hz, 2H), 6.86 – 6.70 (m, 1H), 6.61 (d, *J* = 3.1 Hz, 1H), 3.49 (d, *J* = 14.2 Hz, 1H), 3.31 (d, *J* = 14.2 Hz, 1H), 1.34 (s, 3H).

¹³C NMR (126 MHz, DMSO-d_6) δ 202.8, 189.9, 164.2, 158.2, 156.6, 155.7, 151.6, 140.0, 136.3, 132.5, 127.0, 126.9, 125.0, 124.4, 120.4, 117.9, 117.1, 105.1, 97.0, 62.7, 36.4, 26.1.

HRMS ESI: $[M+H]^+$, Calcd for $C_{22}H_{16}NO_4S$ 390.0800; found 390.0799.

4-(((*R*)-7-amino-10-methyl-6,9,11-trioxo-6,9,10,11-tetrahydroindeno[2,1-c]chromen-10-yl)methyl)phenyl (4R)-4-((3R,5S,7R,10S,12S,13R,17R)-3,7,12-trihydroxy-10,13-dimethylhexadecahydro-1H-cyclopenta[a]phenanthren-17-yl)pentanoate (4y): The title compound was prepared by using the general reaction procedure and purified directly by silica gel column chromatography (eluent: EtOAc/Petroleum ether = 60/40, v/v); deep blue **4y** (47 mg, 59% yield) was obtained. $[\alpha]^{28}_D$ = –131.5° (c = 0.087, $CHCl_3$).

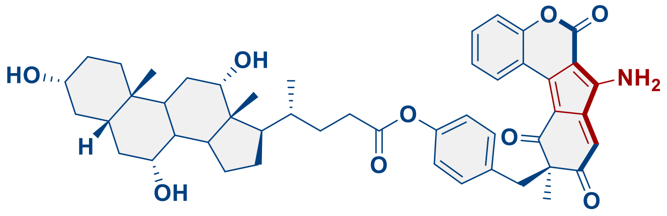

From Cholic Acid

4y

^{1}H NMR (500 MHz, $CDCl_3$) δ 9.82 (t, *J* = 7.8 Hz, 1H), 9.04 (s, 1H), 8.33 (s, 1H), 7.57 (t, *J* = 7.8 Hz, 1H), 7.30 (d, *J* = 8.1 Hz, 1H), 7.17 (d, *J* = 8.4 Hz, 1H), 7.01 – 6.93 (m, 2H), 6.80 – 6.71 (m, 3H), 3.97 (s, 1H), 3.84 (s, 1H), 3.44 (s, 1H), 3.43 – 3.31 (m, 2H), 3.18 – 3.01 (m, 2H), 2.85 (s, 2H), 2.54 – 2.44 (m, 2H), 2.38 (t, *J* = 8.2 Hz, 2H), 2.09 – 1.98 (m, 3H), 1.81 – 1.67 (m, 2H), 1.81 – 1.67 (m, 5H), 1.47 (s, 4H), 1.40 – 1.32 (m, 5H), 1.31 – 1.27 (m, 7H), 0.92 – 0.87 (m, 6H).

^{13}C NMR (126 MHz, $CDCl_3$) δ 203.7, 190.6, 174.7, 173.0, 172.9, 163.7, 159.8, 155.7, 155.5, 150.9, 150.1, 149.0, 136.6, 135.7, 134.6, 132.9, 130.7, 124.7, 120.9, 117.6, 117.2, 113.7, 106.5, 106.3, 98.3, 73.1, 71.9, 68.5, 62.9, 49.5, 46.4, 41.9, 41.4, 39.5, 35.2, 34.7, 34.1, 29.7, 26.6, 22.4, 22.3, 17.7, 17.4, 17.3, 14.0, 12.5, 12.4, 8.6.

HRMS ESI: $[M+H]^+$, Calcd for $C_{48}H_{56}NO_9$ 790.3955; found 790.3958.

(*R*)-4-((7-amino-10-methyl-6,9,11-trioxo-6,9,10,11-tetrahydroindeno[2,1-c]chromen-10-yl)methyl)phenyl palmitate (4z): The title compound was prepared by using the general reaction procedure and purified directly by silica gel column chromatography (eluent: EtOAc/Petroleum ether = 35/65, v/v); light yellow solid **4z** (45.9 mg, 72% yield) was obtained. The *er* of the **4z** was determined to be 97:3 [determined by HPLC, Chiralpak ADH, hexane: isopropanol = 80:20, 1 mL/min, λ = 260 nm, t (major) = 4.3 min, t (minor) = 5.9 min]. $[\alpha]^{29}_D$ = –380.8° (c = 0.10, $CHCl_3$).

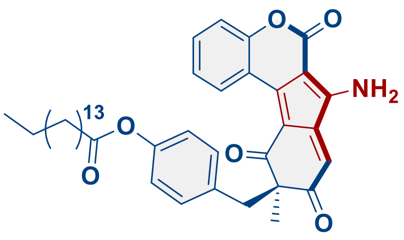

From Palmitic Acid

4z

^{1}H NMR (500 MHz, $CDCl_3$) δ 9.82 (d, *J* = 7.9 Hz, 1H), 8.05 (s, 2H), 7.60 (t, *J* = 7.8 Hz, 1H), 7.32 (t, *J* = 7.6 Hz, 1H), 7.21 (d, *J* = 8.2 Hz, 1H), 7.01 (d, *J* = 8.4 Hz, 2H), 6.80 (d, *J* = 8.4 Hz,

1H), 6.68 (s, 1H), 3.37 (d, J = 13.3 Hz, 1H), 3.13 (d, J = 13.3 Hz, 1H), 2.46 (t, J = 7.6 H, 2H), 1.65 (q, J = 7.5 Hz, 2H), 1.24 (d, J = 8.7 Hz, 27H), 0.87 (t, J = 7.2 Hz, 2H).

^{13}C NMR ^{13}C NMR (126 MHz, $CDCl_3$) δ 203.6, 191.8, 172.8, 163.3, 160.0, 155.4, 150.3, 149.4, 136.6, 134.7, 132.9, 130.9, 124.9, 121.1, 120.7, 117.7, 117.2, 106.4, 98.6, 63.2, 44.1, 34.4, 31.9, 31.4, 30.2, 29.7, 29.6, 29.5, 24.9, 23.6, 22.7, 14.1.

HRMS ESI: $[M+H]^+$, Calcd for $C_{40}H_{48}NO_6$ 638.3482; found 638.3488

(*R*)-4-((7-amino-10-methyl-6,9,11-trioxo-6,9,10,11-tetrahydroindeno[2,1-*c*]chromen-10-yl)methyl)phenyl 5-(2,5-dimethylphenoxy)-2,2 dimethylpentanoate (4aa): The title compound was prepared by using the general reaction procedure and purified directly by silica gel column chromatography (eluent: EtOAc/Petroleum ether = 35/65, v/v); deep blue solid **4aa** (43 mg, 68% yield) was obtained. The *er* of the **4aa** was determined to be 99:1 [determined by HPLC, Chiralpak ADH, hexane: isopropanol = 80:20, 1 mL/min, λ = 260 nm, t (major) = 5.9 min, t (minor) = 12.9 min]. $[\alpha]^{29}_D$ = –296.1° (c = 0.10, $CHCl_3$).

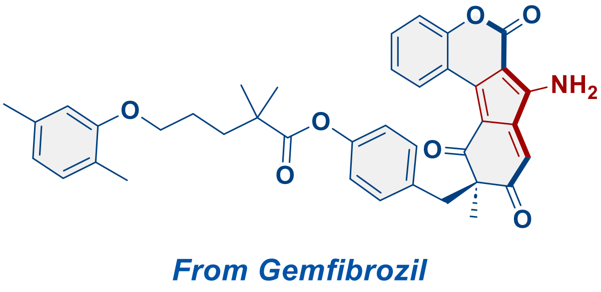

4aa

^{1}H NMR (500 MHz, $CDCl_3$) δ 9.85 (d, J = 8.1 Hz, 1H), 8.09 (s, 1H), 7.96 (s, 1H), 7.62 (t, J = 6.9 Hz, 1H), 7.34 (t, J = 7.1 Hz, 1H), 7.22 (d, J = 7.5 Hz, 1H), 7.05 (d, J = 8.5 Hz, 2H), 6.96 (d, J = 7.5 Hz, 1H), 6.80 (d, J = 8.7 Hz, 2H), 6.71 (s, 1H), 6.64 (d, J = 7.6 Hz, 1H), 6.60 (s, 1H), 3.94 (t, J = 5.6 Hz, 2H), 3.40 (d, J = 13.4 Hz, 1H), 3.16 (d, J = 13.4 Hz, 1H), 2.29 (s, 3H), 2.13 (s, 3H), 1.86 – 1.77 (m, 4H), 1.50 (s, 3H), 1.32 (s, 6H).

^{13}C NMR (126 MHz, $CDCl_3$) δ 203.1, 190.6, 176.9, 162.7, 160.0, 156.8, 155.4, 150.6, 149.6, 146.0, 136.5, 136.0, 134.8, 132.9, 130.9, 130.3, 125.0, 123.5, 121.1, 120.8, 117.7, 117.2, 112.0, 106.4, 98.7, 67.7, 63.2, 43.9, 42.4, 37.1, 25.2, 25.1, 23.7, 21.4, 15.8.

HRMS ESI: $[M+H]^+$, Calcd for $C_{39}H_{38}NO_7$ 632.2648; found 632.2650.

(*R*)-4-((7-amino-10-methyl-6,9,11-trioxo-6,9,10,11-tetrahydroindeno[2,1-c]chromen-10-yl)methyl)phenyl 2-(1-(4-chlorobenzoyl)-5-methoxy-2-methyl-1H-indol-3-yl)acetate (4ab). The title compound was prepared by using the general reaction procedure and purified directly by silica gel column chromatography (eluent: EtOAc/Petroleum ether = 35/65, v/v); deep blue solid **4ab** (59.9 mg, 81% yield) was obtained. The *er* of the **4ab** was determined to be 97:3 [determined by HPLC, Chiralpak ADH, hexane: isopropanol = 40:60, 1 mL/min, λ = 260 nm, t (major) = 15.2 min, t (minor) = 18.9 min]. $[\alpha]^{29}_{D}$ = –153.3° (c = 0.074, $CHCl_3$).

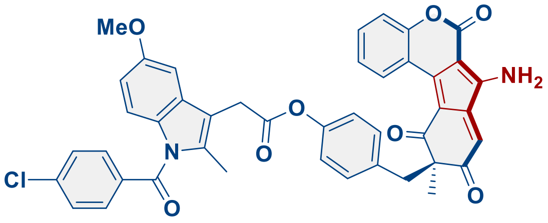

From Indomethacin

4ab

^{1}H NMR (500 MHz, $CDCl_3$) δ 9.82 (d, *J* = 8.1 Hz, 1H), 7.99 (s, 1H), 7.89 (s, 1H), 7.64 – 7.58 (m, 3H), 7.46 – 7.41 (m, 2H), 7.33 (t, *J* = 7.0 Hz, 1H), 7.21 (d, *J* = 7.0 Hz, 1H), 7.04 – 6.98 (m, 2H), 6.93 (d, *J* = 2.6 Hz, 1H), 6.84– 6.77 (m, 3H), 6.60 (dd, *J* = 9.0, 2.6 Hz, 1H), 6.56 (s, 1H), 3.82 (s, 2H), 3.74 (s, 3H), 3.36 (d, *J* = 13.3 Hz, 1H), 3.11 (d, *J* = 13.4 Hz, 1H), 2.37 (s, 3H), 1.46 (s, 3H).

^{13}C NMR (126 MHz, $CDCl_3$) δ 203.0, 190.5, 169.9, 168.3, 162.5, 159.8, 156.0, 155.4, 150.5, 149.3, 139.4, 136.3, 136.0, 135.0, 133.7, 132.9, 131.2, 130.9, 130.8, 130.4, 129.2, 124.9, 120.9, 120.5, 117.7, 117.1, 115.0, 111.9, 111.5, 106.3, 101.4, 98.5, 63.1, 55.7, 43.9, 30.4, 23.8, 13.4.

HRMS ESI: $[M+H]^+$, Calcd for $C_{43}H_{32}ClN_2O_8$ 739.1847; found 739.1879.

4-(((*R*)-7-amino-10-methyl-6,9,11-trioxo-6,9,10,11-tetrahydroindeno[2,1-c]chromen-10-yl)methyl)phenyl (S)-2-(6-methoxynaphthalen-2-yl)propanoate (4ac): The title compound was prepared by using the general reaction procedure and purified directly by silica gel column chromatography (eluent: EtOAc/Petroleum ether = 50/50, v/v); light yellow solid **4ac** (53 mg, 87% yield) was obtained. The *er* of the **4ac** was determined to be 97:3 [determined by HPLC, Chiralpak ADH, hexane: isopropanol = 40:60, 1 mL/min, λ = 260 nm, t (major) = 8.1 min, t (minor) = 13.1 min]. $[\alpha]^{29}_{D}$ = –186.7° (c = 0.067, $CHCl_3$).

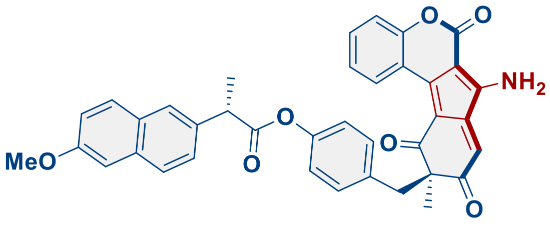

From Naproxen

4ac

^{1}H NMR (500 MHz, $CDCl_3$) δ 9.86 – 9.72 (m, 1H), 7.94 (s, 1H), 7.88 (s, 1H), 7.66 – 7.59 (m, 3H), 7.56 (q, *J* = 8.7, 7.3, 5.7, 1.6 Hz, 1H), 7.36 (d, *J* = 10.5 Hz, 1H), 7.33 – 7.25 (m, 1H), 7.18 (dd, *J* = 7.7, 3.9 Hz, 1H), 7.09 – 7.04 (m, 1H), 7.03 – 6.96 (m, 3H), 6.76 (d, *J* = 6.9 Hz, 2H), 6.57 (d, *J* = 11.3 Hz, 1H), 4.00 (q, *J* = 7.2 Hz, 1H), 3.84 (d, *J* = 2.7 Hz, 3H), 3.35 (d, *J* = 10.7 Hz, 1H), 3.11 (d, *J* = 10.2 Hz, 1H), 1.62 (d, *J* = 5.6 Hz, 3H), 1.46 (s, 3H).

^{13}C NMR (126 MHz, $CDCl_3$) δ 203.1, 190.5, 173.9, 173.8, 162.6, 160.3, 157.7, 155.4, 150.5, 149.4, 135.9, 134.9, 134.8, 133.8, 132.8, 130.9, 129.2, 128.8, 127.4, 126.1, 126.0, 124.9, 121.0, 120.5, 119.1, 117.7, 117.1, 106.3, 105.5, 98.5, 63.1, 55.3, 45.5, 44.0, 23.7, 19.0.

HRMS ESI: $[M+H]^+$, Calcd for $C_{38}H_{30}NO_7$ 612.2022; found 612.2028.

(*R*)-1-(10-benzyl-10-methyl-6,9,11-trioxo-6,9,10,11-tetrahydroindeno[2,1-c]chromen-7-yl)-3-(3,5-bis(trifluoromethyl)phenyl)thiourea (5): The title compound was prepared by using modified reported procedure.[5] to a solution of **3a**, (0.1 mmol, 38.3 mg 1 equiv) in 1 ml of dry DCM, was added triethyl amine (0.1 mmol, 14 µl, 1.5 equiv) and 3,5-Bis(trifluoromethyl)phenyl Isothiocyanate (0.11 mmol, 20 µl, 1.1 equiv) at room temperature. After stirring for 5 min at ambient temperature, the reaction mixture was shifted to 35 °C for 8 h. After the completion of reaction, the reaction mixture was directly processed for purification through silica gel chromatography (eluent: EtOAc/Hexane 35:65); green solid (28.6 mg, 44% yield). The *er* of the **5** was determined to be 99.5:0.5 [determined by HPLC, Chiralpak IB, hexane: isopropanol = 85:15, 1 mL/min, λ = 260 nm, t (major) = 28.6 min, t (minor) = 50.2 min]. $[\alpha]^{29}_{D}$ = –106.7° (c = 0.11, $CHCl_3$).

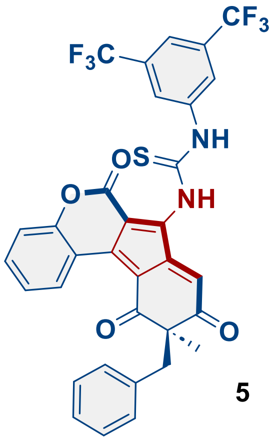

5

^{1}H NMR (500 MHz, $CDCl_3$) δ 9.84 (d, *J* = 7.8 Hz, 1H), 8.43 (s, 2H), 7.78 (s, 1H), 7.70 – 7.58 (m, 1H), 7.40 (d, *J* = 6.4 Hz, 1H), 7.26 (s, 2H), 6.95 (d, *J* = 6.6 Hz, 3H), 6.87 (d, *J* = 5.8 Hz, 2H), 3.45 (d, *J* = 13.3 Hz, 1H), 3.18 (d, *J* = 13.2 Hz, 1H), 1.63 (s, 3H)

^{13}C NMR (126 MHz, $CDCl_3$) δ 198.2, 189.3, 172.9, 160.3, 157.8, 154.6, 151.9, 140.6, 135.8, 135.2, 133.2, 133.0, 132.0, 130.9, 129.4, 128.0, 127.0, 126.0, 124.8, 124.1, 121.9, 119.0, 117.5, 117.4, 106.4, 64.7, 46.9, 22.8.

^{19}F NMR (471 MHz, $CDCl_3$) δ -62.80.

HRMS ESI: $[M–H]^+$, Calcd for $C_{33}H_{19}F_6N_2O_4S$ 653.0970; found 653.0930.

(*R*)-N-(10-benzyl-10-methyl-6,9,11-trioxo-6,9,10,11-tetrahydroindeno[2,1-c]chromen-7-yl)-3,5-bis(trifluoromethyl)benzamide (6): The title compound was prepared by using modified reported procedure.[6] to a solution of **3a**, (0.1 mmol, 38.3 mg 1 equiv) in 1 ml of dry DCM, was added triethyl amine (0.3 mmol, 41.8 μl, 3 equiv) and 3,5-bis(trifloromethylbenzoyl chloride) (0.12 mmol, 21.7 μl, 1.2 equiv) at -78 °C. After stirring for 1 h at -78 °C, the reaction mixture was shifted to room temperature. Further stithe reaction quenched by saturated solution of $NaHCO_3$. The aqueous phase was extracted with DCM (2 ×10 ml). The combine d organic phase was washed with brine, dried over Na_2SO_4, and concentrated under reduced pressure. The crude reaction mixture was purified by silica gel chromatography (eluent: EtOAc/Hexane = 60:40); obtained **6** blue solid (28.5 mg, 45% yield). The *er* of the **6** was determined to be 99:1 [determined by HPLC, Chiralpak IB, hexane: isopropanol = 80:20, 1 mL/min, λ = 260 nm, t (major) = 10.3 min, t (minor) = 24.8 min]. $[\alpha]^{29}_D$ = –76.8° (c = 0.11, $CHCl_3$).

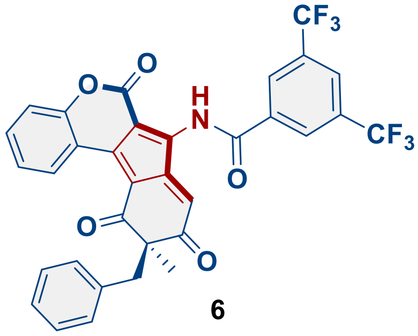

6

^{1}H NMR (500 MHz, DMSO-d_6) δ 10.04 (s, 1H), 8.43 (d, *J* = 20.1 Hz, 1H), 7.65 (t, *J* = 7.9 Hz, 1H), 7.29 (t, *J* = 7.6 Hz, 1H), 7.22 (d, *J* = 8.2 Hz, 2H), 7.08 (t, *J* = 7.3 Hz, 3H), 7.03 (dd, *J* = 8.5, 5.8 Hz, 1H), 6.93 (d, *J* = 7.2 Hz, 2H), 5.89 (s, 1H), 3.25 (s, 1H), 3.05 (d, *J* = 13.4 Hz, 1H), 1.28 (s, 3H).

^{13}C NMR (126 MHz, DMSO-d_6) δ 204.6, 189.5, 188.3, 163.5, 156.1, 153.6, 138.7, 134.9, 132.8, 129.7, 128.1, 126.3, 123.5, 117.9, 117.1, 110.1, 101.8, 62.2, 43.6, 26.0.

^{19}F NMR (471 MHz, $CDCl_3$) δ -61.37.

LRMS ESI: $[M]^+$, Calcd for $C_{33}H_{19}F_6NO_5$ 623.1167; found 623.0936.

(*R*)-10-benzyl-7-bromo-10-methylindeno[2,1-c]chromene-6,9,11(10H)-trione (7): In an oven dry reaction tube **3a** (38.3 mg, 0.1 mmol, 1 equiv) was added to 0.3 ml of 48% HBr. A pink suspension thus formed was shifted to -50 °C. Next, sodium nitrite (10.7 mg, 0.15 mmol, 1.5 equiv) was added portion wise After stirring for 2 h, 2 ml diethyl ether was added slowly into the reaction tube and the temperature was adjusted to -8 °C over 2 h. and further the resulting mixture was kept at -8 °C additional 2 h. Then, the reaction mixture was again cooled to -40 °C, 100 mg of Na_2CO_3 was added, then the reaction was allowed to warm up to room temperature over 3 h

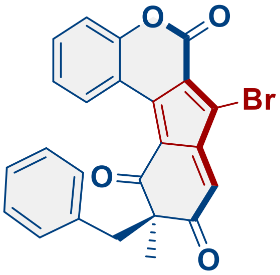

7

and stirred overnight. Then the reaction mixture was diluted with water and extracted with ethyl acetate. The organic layer was washed with water, sodium bicarbonate, concentrated under reduced pressure and purified through silica gel chromatography (eluent: EtOAc/Hexane = 30:70); yellow solid (13.4 mg, 30% yield). The er of the **7** was determined to be 90:10 [determined by HPLC, Chiralpak ADH, hexane: isopropanol = 70:30, 1 mL/min, λ = 280 nm, t (major) = 4.9 min, t (minor) = 7.6 min]. $[\alpha]^{30}_{D}$ = –68.8° (c = 0.12, $CHCl_3$).

^{1}H NMR (500 MHz, DMSO-d_6) δ 10.04 (d, J = 6.3 Hz, 1H), 7.66 (t, J = 6.8 Hz, 1H), 7.30 (t, J = 7.7 Hz, 1H), 7.24 (d, J = 8.2 Hz, 1H), 7.12 – 7.01 (m, 4H), 6.95 – 6.89 (m, 2H), 3.28 (d, J = 13.3 Hz, 1H), 3.06 (d, J = 13.3 Hz, 1H), 1.33 (s, 3H).

^{13}C NMR (126 MHz, DMSO-d_6) δ 197.9, 188.2, 186.3, 160.3, 156.2, 156.0, 147.6, 137.9, 134.6, 132.9, 129.8, 128.1, 126.6, 123.4, 117.6, 117.1, 108.0, 104.2, 101.3, 62.9, 45.2, 25.2.

LRMS ESI: $[M+Na]^+$, Calcd for $C_{24}H_{15}BrNaO_4$ 469.0051; found 469.3035

(*R*)-7-amino-8,10-dibenzyl-10-methylindeno[2,1-c]chromene-6,9,11(10H)-trione (8): In

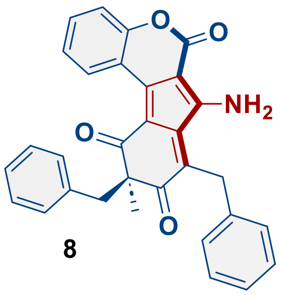

8

an oven dried seal tube, **3a** (38.3 mg, 0.1 mmol, 1 equiv) was dissolved in toluene 1 ml. The resulting reaction mixture was heated at 180 °C for 16 h. After the complete consumption of **3a**, the crude reaction mixture was purified by silica gel column chromatography (eluent: EtOAc/Petroleum ether = 70/30, v/v); blue solid **8** (14.7 mg, 31% yield) was obtained. The *er* of the **8** was determined to be 94:6 [determined by HPLC, Chiralpak ADH, hexane: isopropanol = 90:10, 1 mL/min, λ = 265 nm, t (major) = 19.5 min, t (minor) = 21.9 min]. $[\alpha]^{29}_{D}$ = –60.0° (c = 0.09, $CHCl_3$).

^{1}H NMR (500 MHz, DMSO-d_6) δ 9.84 (d, J = 8.1 Hz, 1H), 9.18 (s, 1H), 8.90 (s, 1H), 7.72 (t, J = 7.7 Hz, 1H), 7.39 (t, J = 7.8 Hz, 1H), 7.34 (d, J = 8.2 Hz, 1H), 7.29 – 7.24 (m, 2H), 7.23 – 7.19 (m, 1H), 7.12 – 7.02 (m, 5H), 6.80 (d, J = 7.3 Hz, 2H), 4.35 – 4.18 (m, 2H), 3.20 (d, J = 13.4 Hz, 1H), 3.06 (d, J = 13.3 Hz, 1H), 1.30 (s, 3H).

^{13}C NMR (126 MHz, DMSO-d_6) δ 202.0, 190.1, 163.2, 159.3, 155.1, 151.6, 145.5, 137.5, 137.1, 136.9, 135.7, 132.2, 130.0, 129.1, 128.7, 128.3, 127.0, 126.8, 124.7, 117.9, 117.2, 107.1, 98.2, 61.8, 44.6, 30.8, 22.7.

HRMS ESI: $[M+H]^+$, Calcd for $C_{31}H_{24}NO_4$ 474.1705; found 474.1728.

(*R*)-7-amino-10-benzyl-8-bromo-10-methylindeno[2,1-c]chromene-6,9,11(10H)-trione 9:

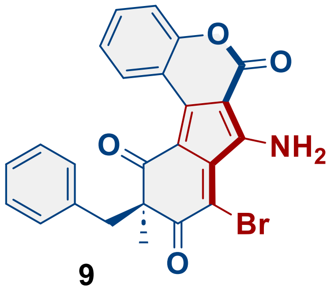

9

In an oven dried reaction tube, **3a** (38.3 mg, 0.1 mmol, 1 equiv) was dissolved in DMF (1 ml). Next, NBS (*N*-bromosuccinimide) (18 mg, 0.1 mmol, 1 equiv) was added portion wise into the above resulting solution. The reaction was allowed to stir at room temperature for 5 min. After completion of the reaction, the solution was diluted with water and extracted with ethyl acetate (10 ml ×3). The combined organic phase was washed first with water three times followed by washing with brine and finally dried over Na_2SO_4. The crude reaction mixture was purified by silica gel column chromatography (eluent: EtOAc/Petroleum ether = 70/30, v/v); greenish blue solid **9** (40.2 mg, 87% yield) was obtained. The *er* of the **9** was determined to be 99:1 [determined by HPLC, Chiralpak ADH, hexane: isopropanol = 90:10, 1 mL/min, λ = 265 nm, t (major) = 23.6 min, t (minor) = 25.8 min]. $[\alpha]^{29}_{D}$ = –484.0° (c = 0.10, $CHCl_3$).

^{1}H NMR (500 MHz, $CDCl_3$) δ 9.92 (d, J = 8.1 Hz, 1H), 8.71 (s, 1H), 8.13 (s, 1H), 7.67 (t, J = 7.7 Hz, 1H), 7.38 (t, J = 7.7 Hz, 1H), 7.28 (d, J = 8.5 Hz, 1H), 7.12 – 7.01 (m, 3H), 6.97 – 6.87 (m, 2H), 3.35 (d, J = 13.1 Hz, 1H), 3.16 (d, J = 13.1 Hz, 1H), 1.58 (s, 3H).

^{13}C NMR NMR (126 MHz, $CDCl_3$) δ 195.3, 189.4, 161.6, 160.0, 155.4, 152.6, 146.3, 136.0, 135.9, 135.8, 133.1, 129.6, 128.0, 127.0, 125.0, 124.1, 117.9, 116.9, 108.9, 99.3, 63.6, 47.5, 22.6

HRMS ESI: $[M+H]^+$, Calcd for $C_{24}H_{17}BrNO_4$ 462.0341; found 462.0359.

7. ^{1}H NMR and $^{13}C\{H\}$ NMR spectra

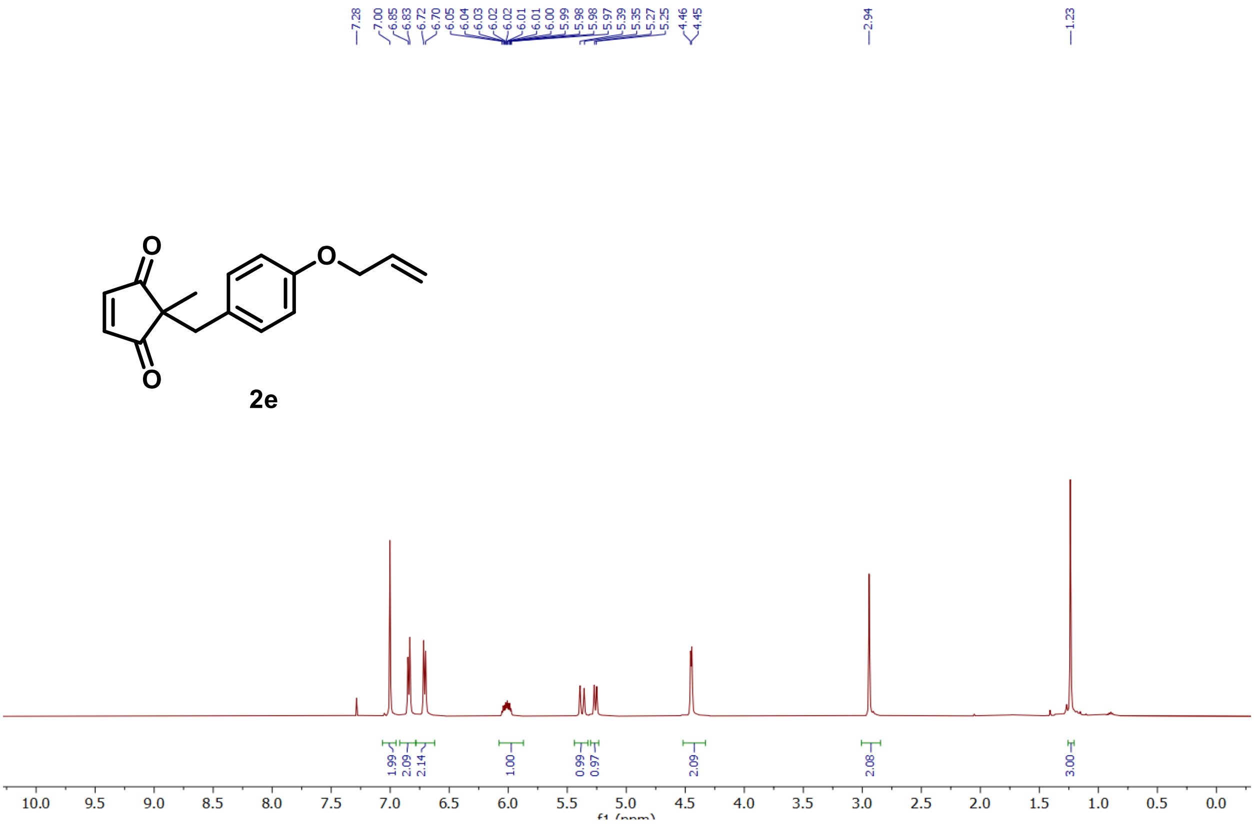

Supplementary Fig. 3. **^{1}H NMR (500 MHz, $CDCl_3$) spectrum for 2e.**

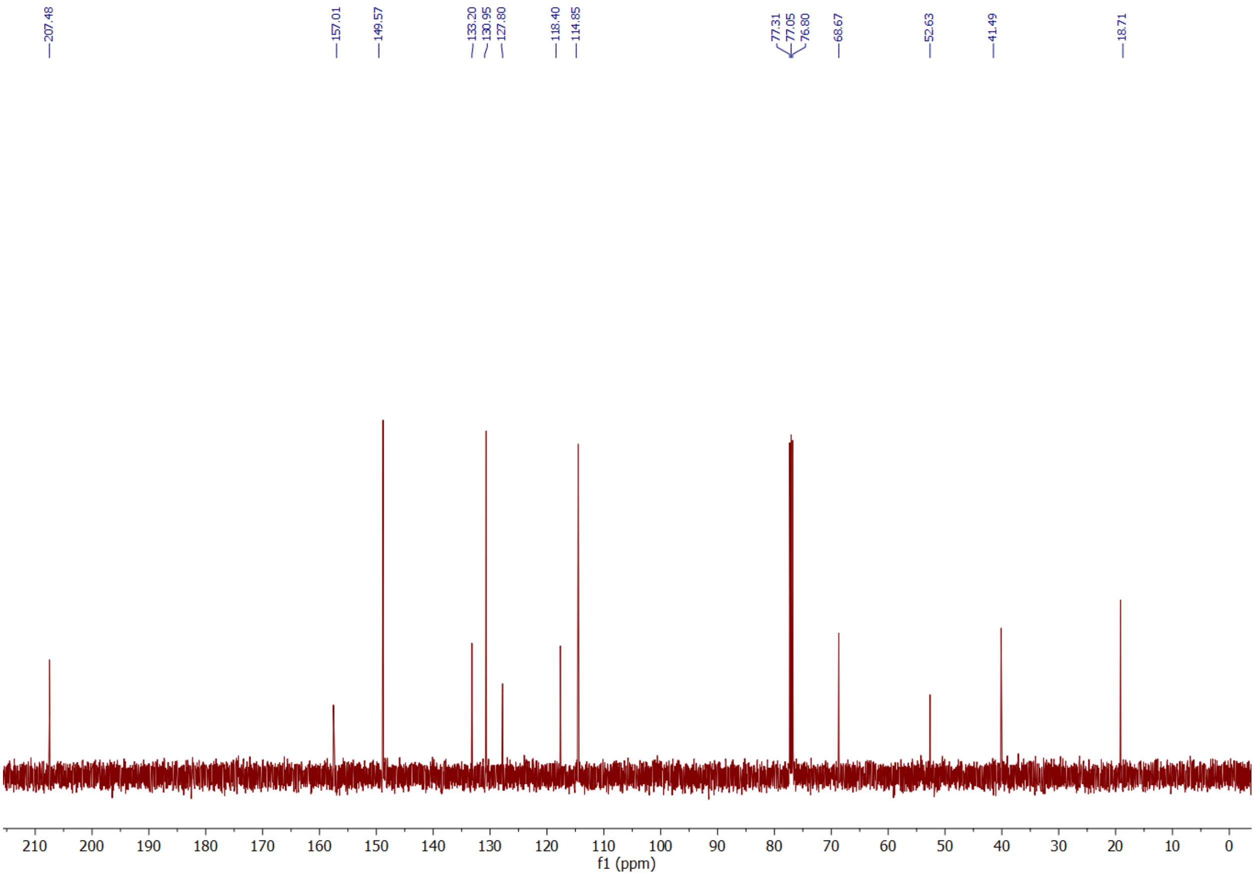

Supplementary Fig. 4. **^{13}C NMR (126 MHz, $CDCl_3$) spectrum for 2e.**

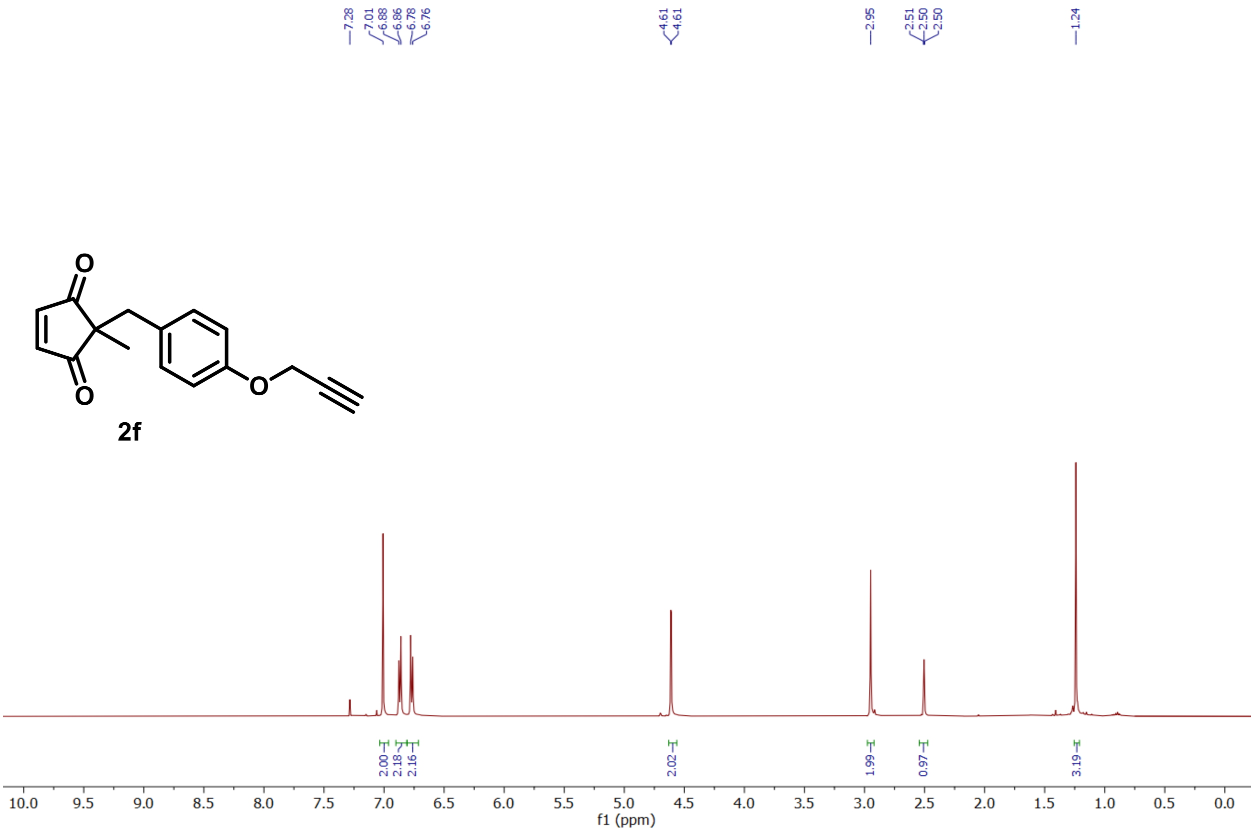

Supplementary Fig. 5. ^{1}H NMR (500 MHz, $CDCl_3$) spectrum for 2f.

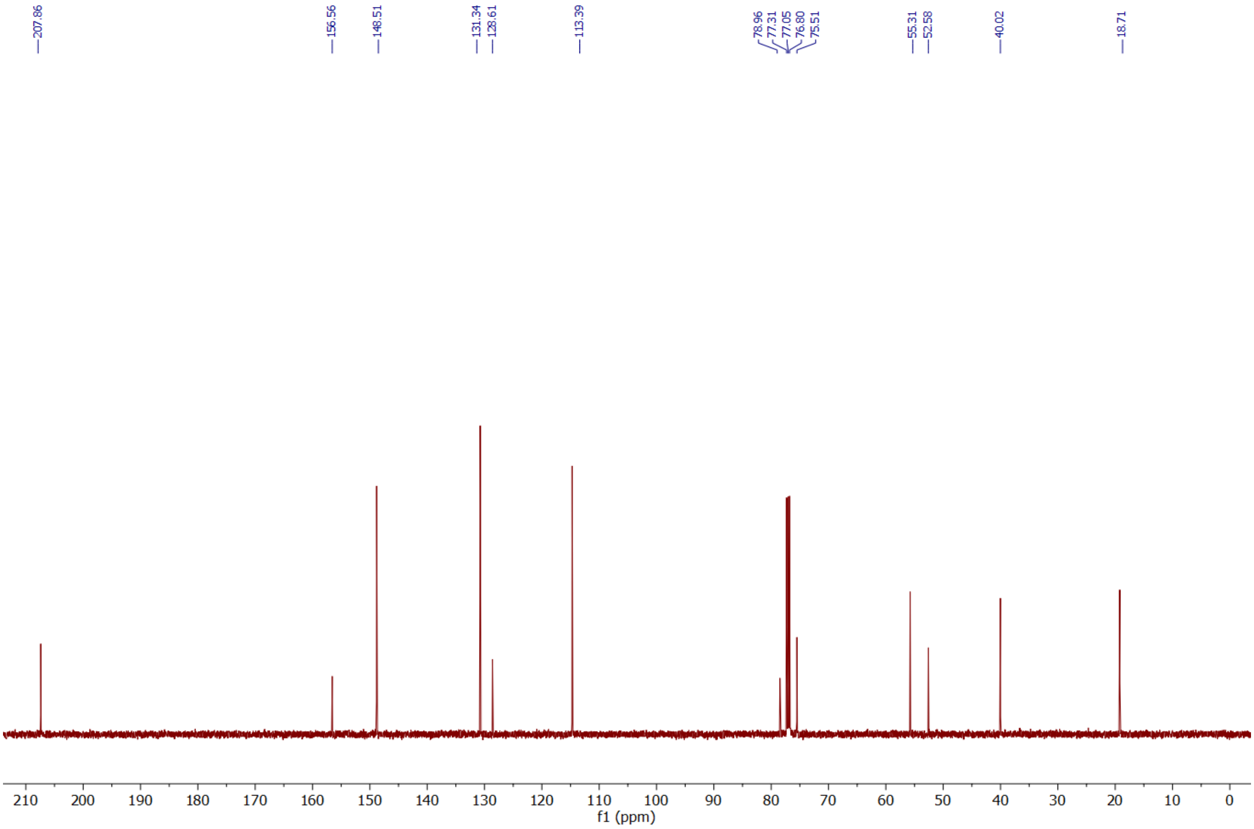

Supplementary Fig. 6. ^{13}C NMR (126 MHz, $CDCl_3$) spectrum for 2f.

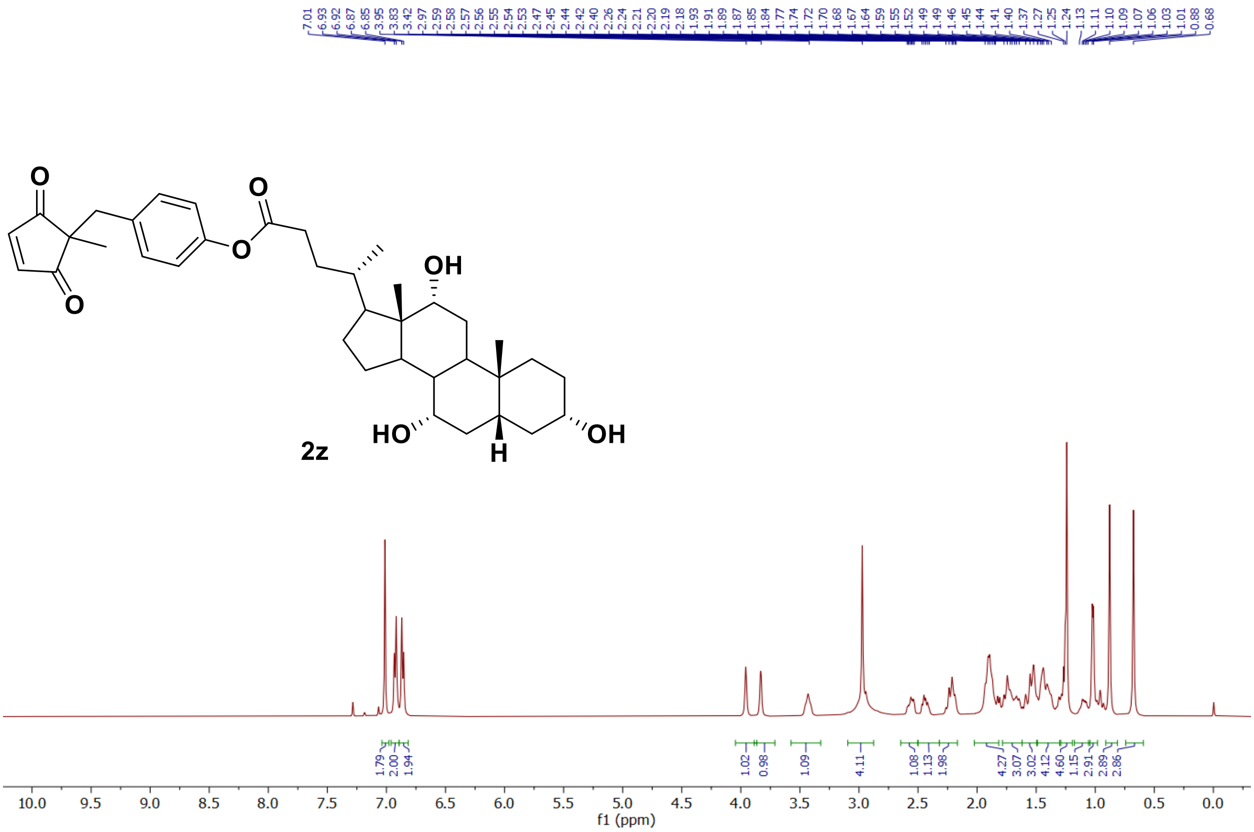

Supplementary Fig. 7. ^{1}H NMR (500 MHz, $CDCl_3$) spectrum for 2z.

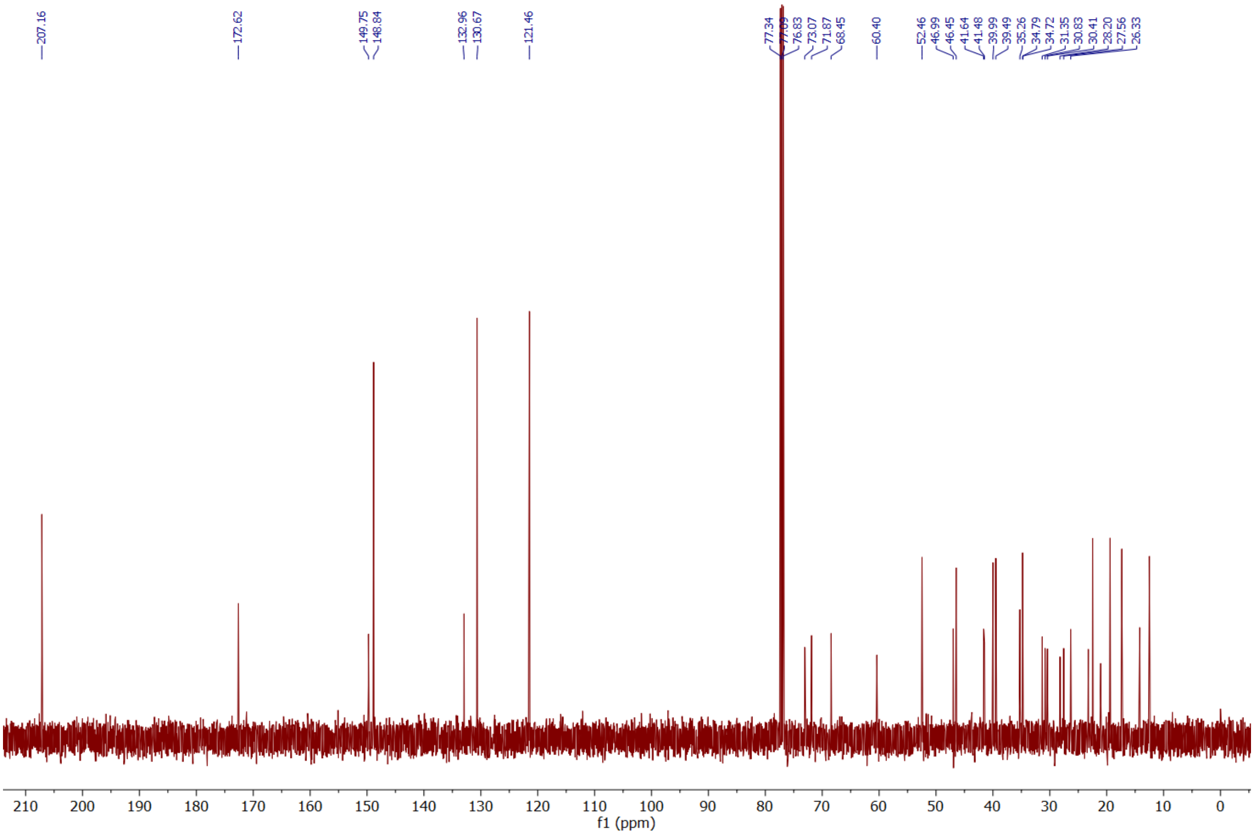

Supplementary Fig. 8. ^{13}C NMR (126 MHz, $CDCl_3$) spectrum for 2z.

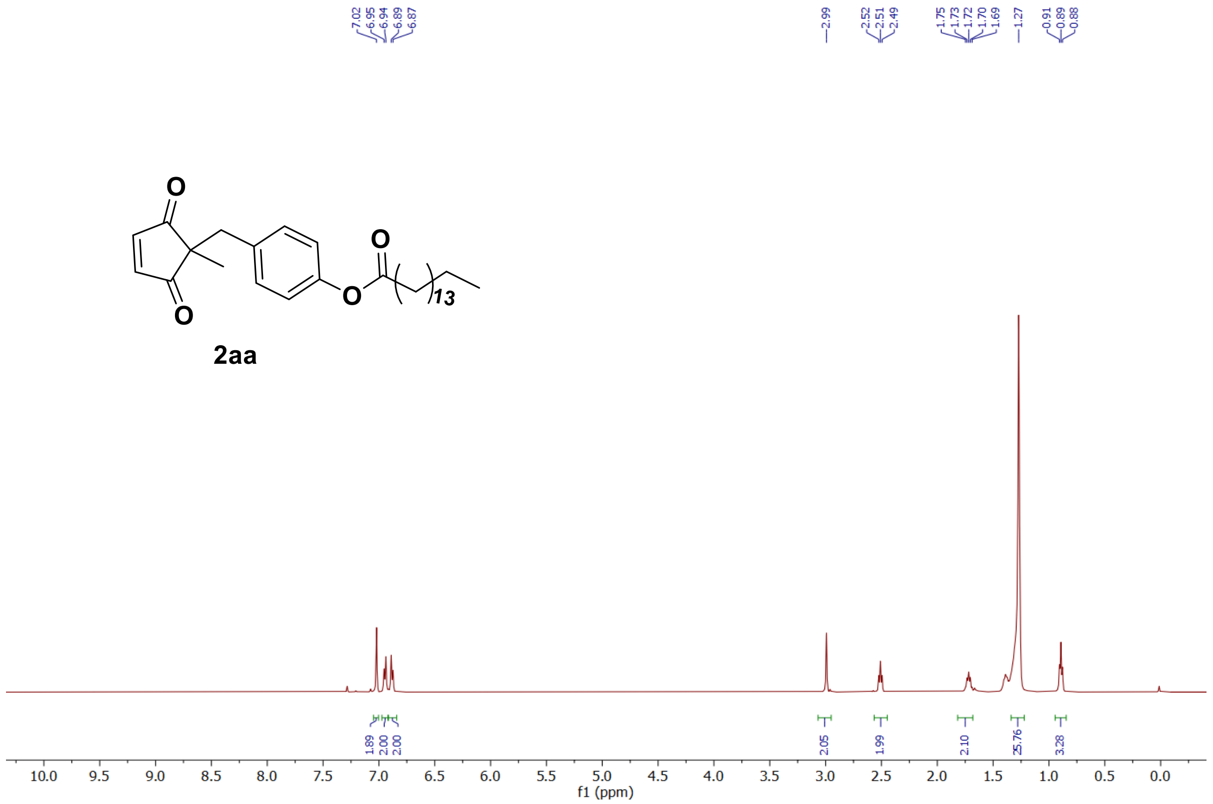

Supplementary Fig. 9. ^{1}H NMR (500 MHz, $CDCl_3$) spectrum for 2aa.

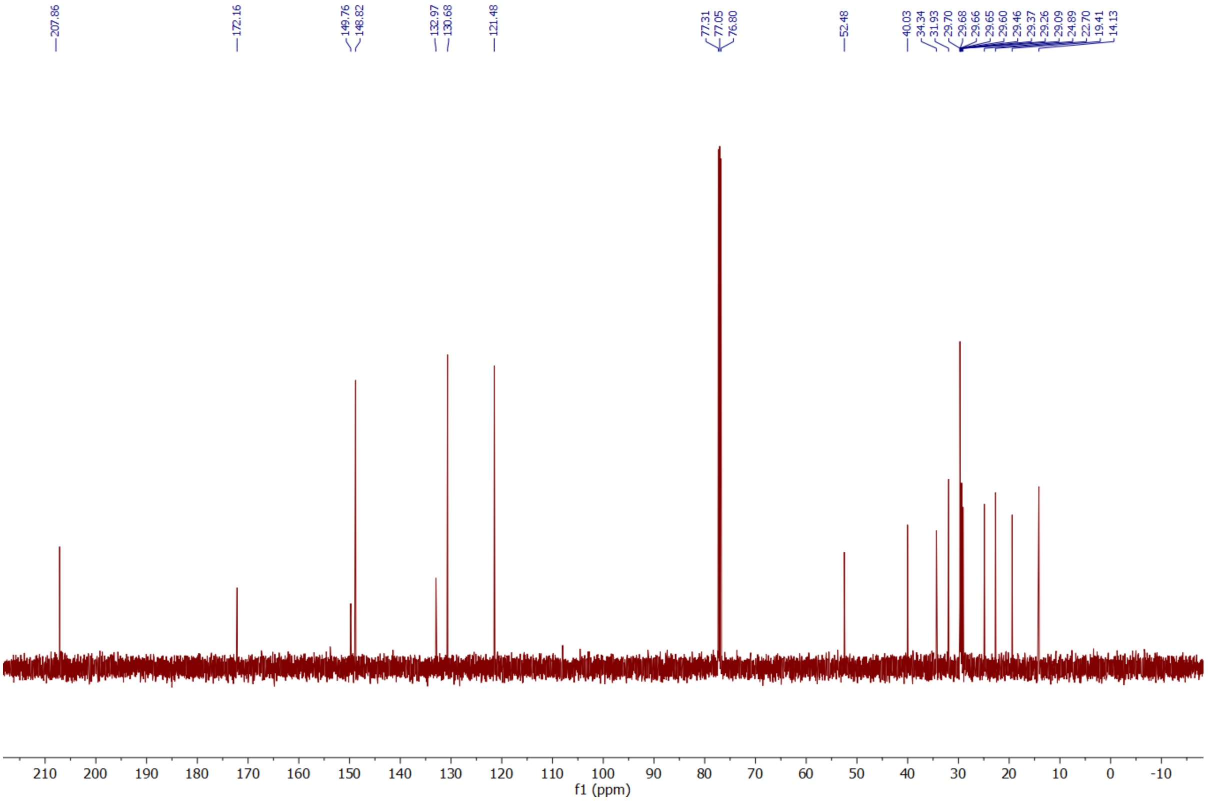

Supplementary Fig. 10. ^{13}C NMR (126 MHz, $CDCl_3$) spectrum for 2aa.

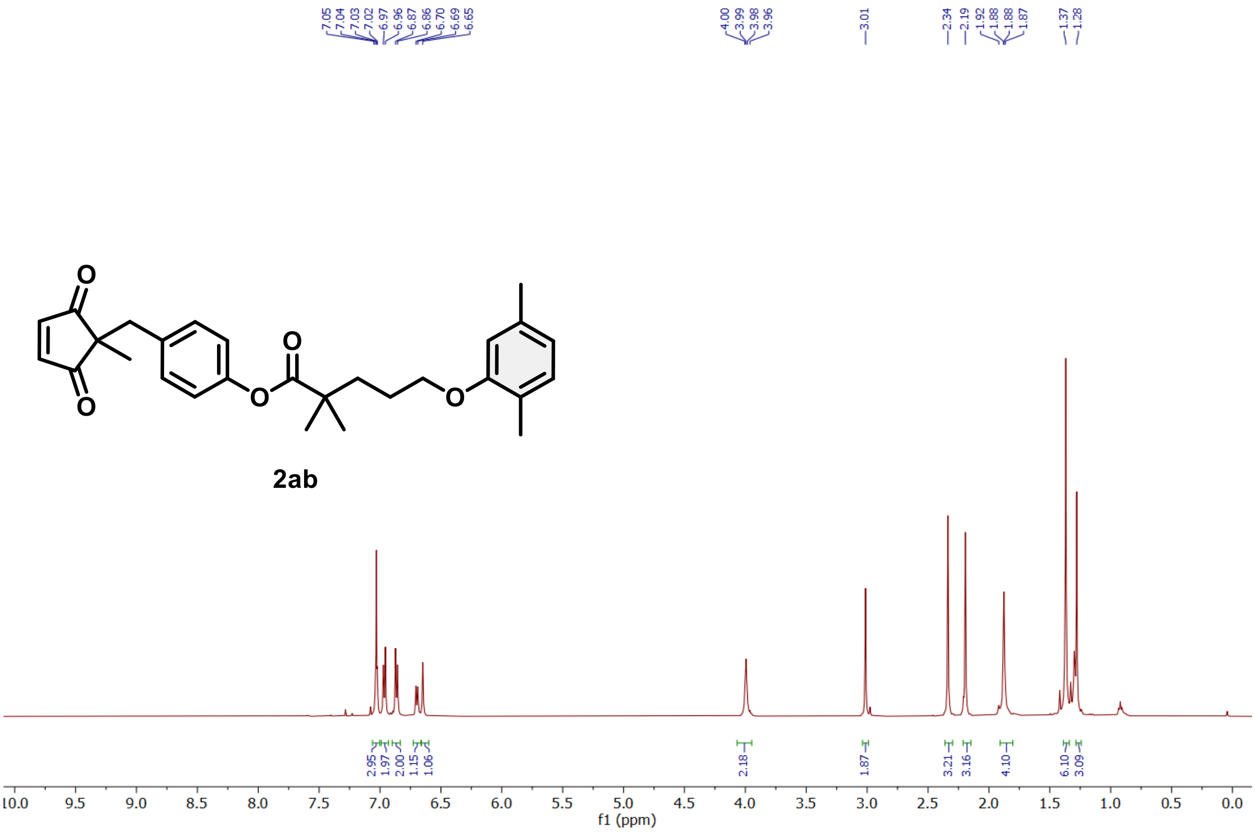

Supplementary Fig. 11. ^{1}H NMR (500 MHz, $CDCl_3$) spectrum for 2ac.

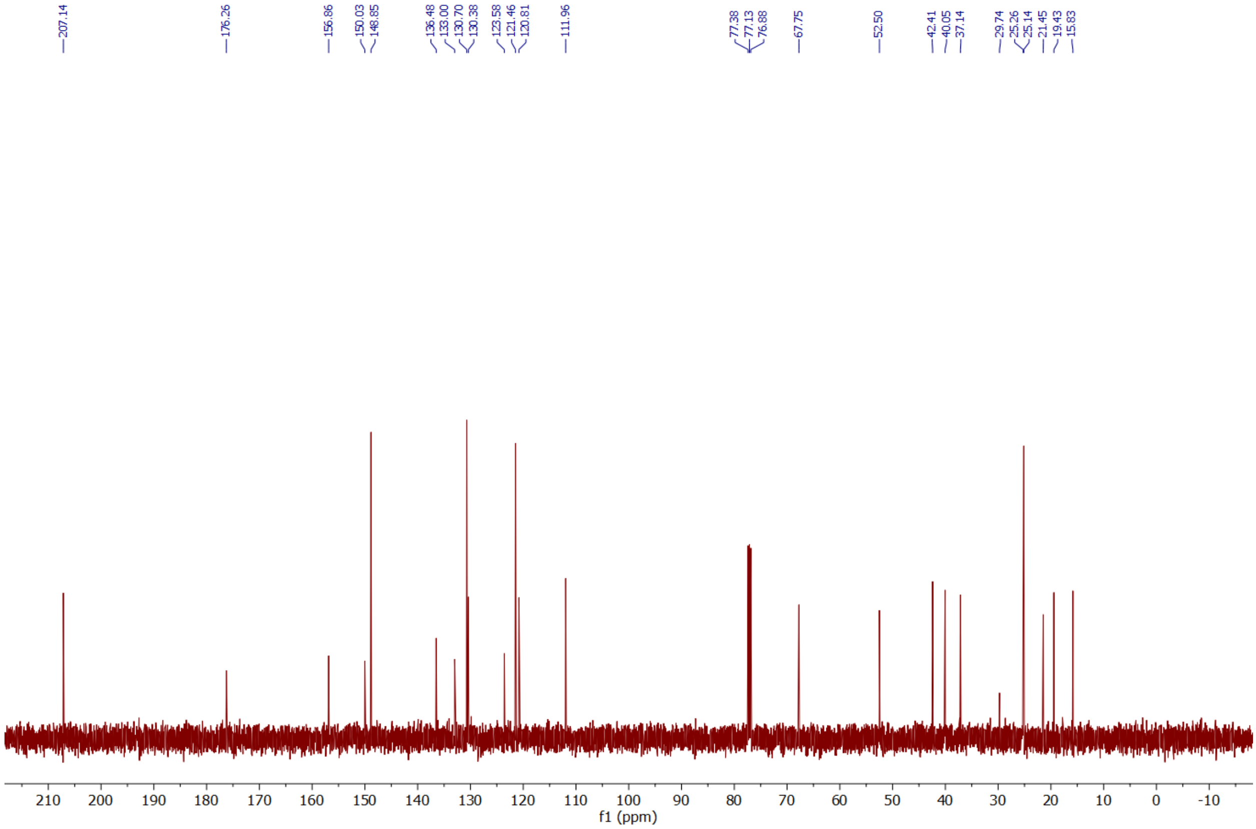

Supplementary Fig. 12. ^{13}C NMR (126 MHz, $CDCl_3$) spectrum for 2ac.

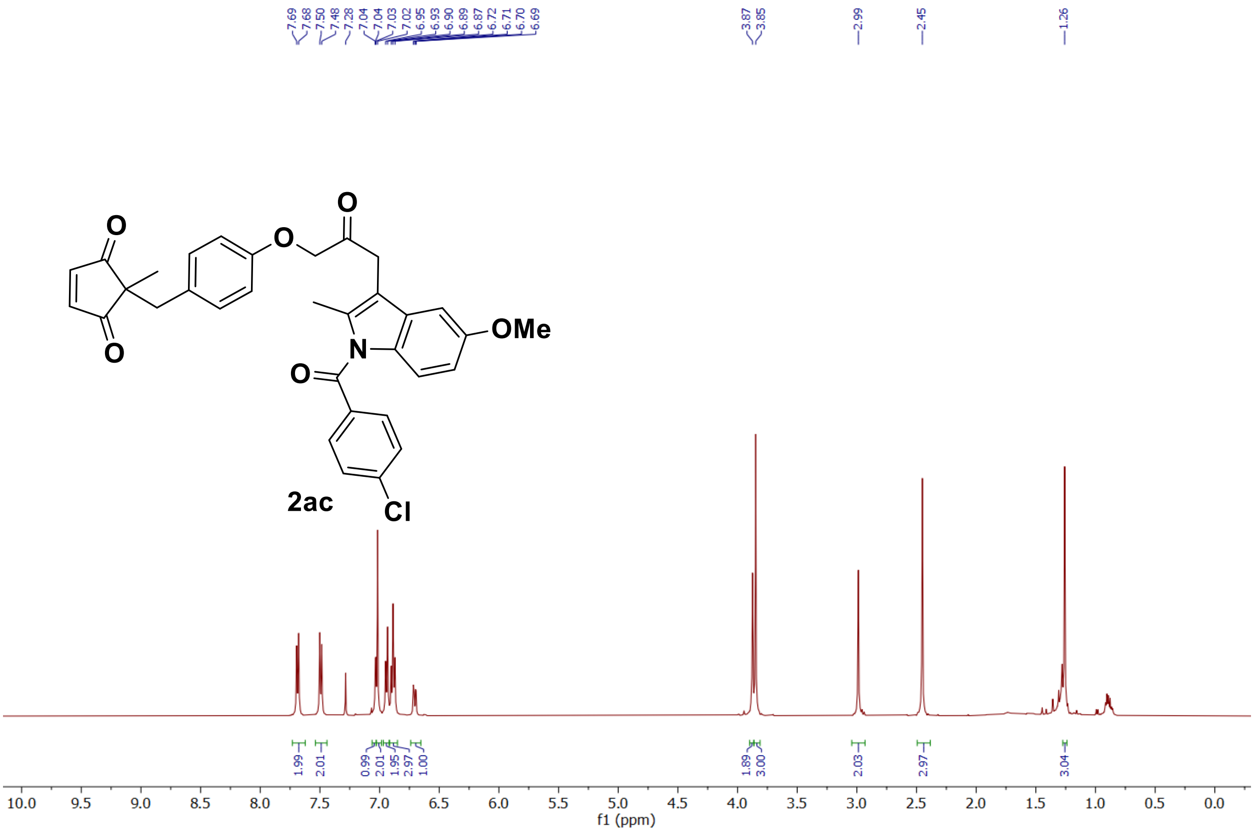

Supplementary Fig. 13. ^{1}H NMR (500 MHz, $CDCl_3$) spectrum for 2ac.

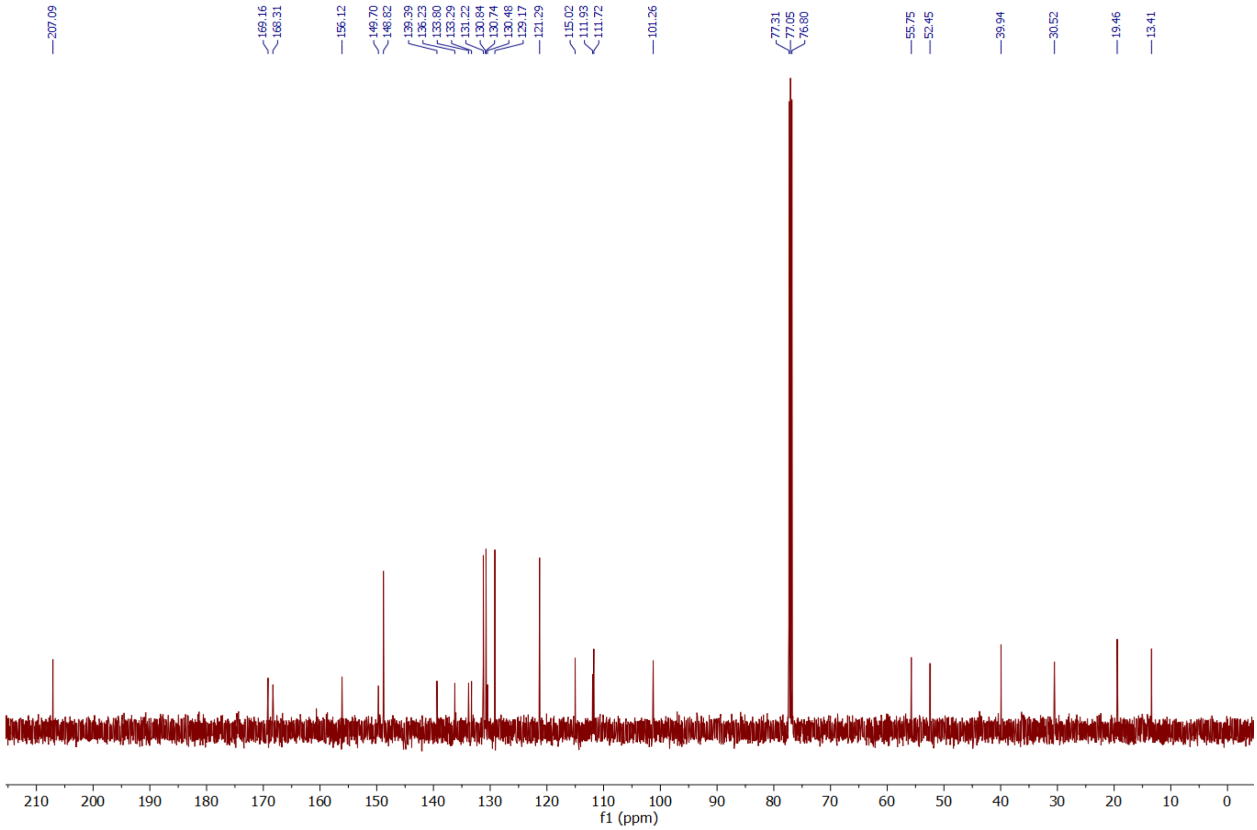

Supplementary Fig. 14. ^{13}C NMR (126 MHz, $CDCl_3$) spectrum for 2ac.

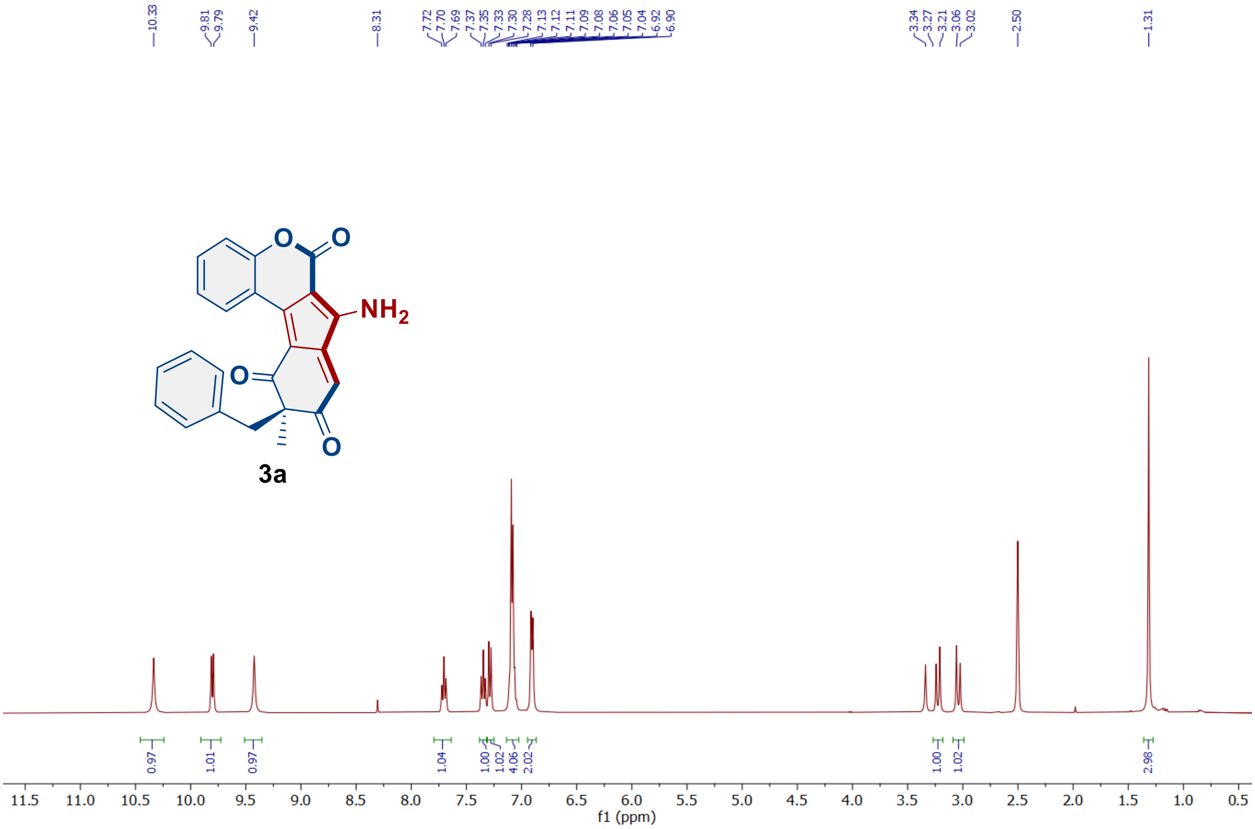

Supplementary Fig. 15. ^{1}**H NMR (500 MHz, , DMSO-*d*$_{6}$) spectrum for 3a.**

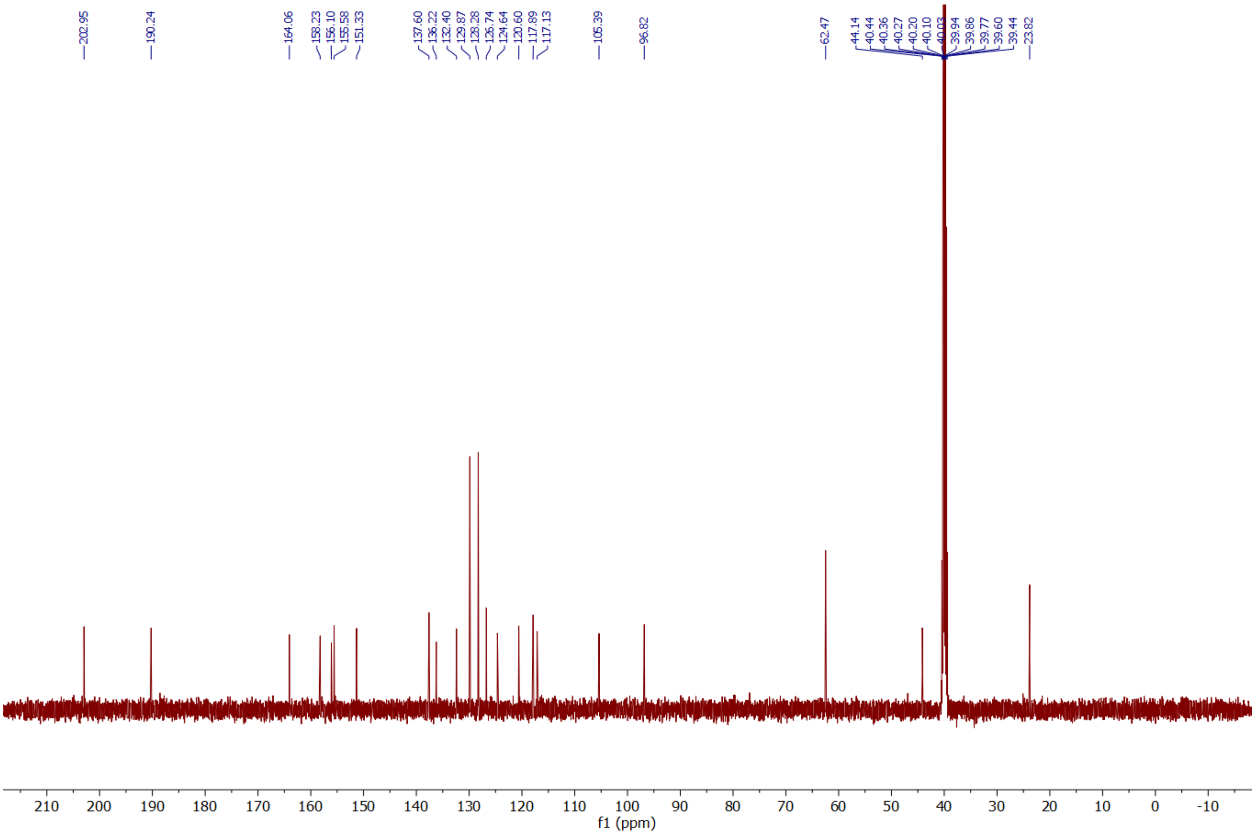

Supplementary Fig. 16. ^{13}**C NMR (126 MHz, , DMSO-*d*$_{6}$) spectrum for 3a.**

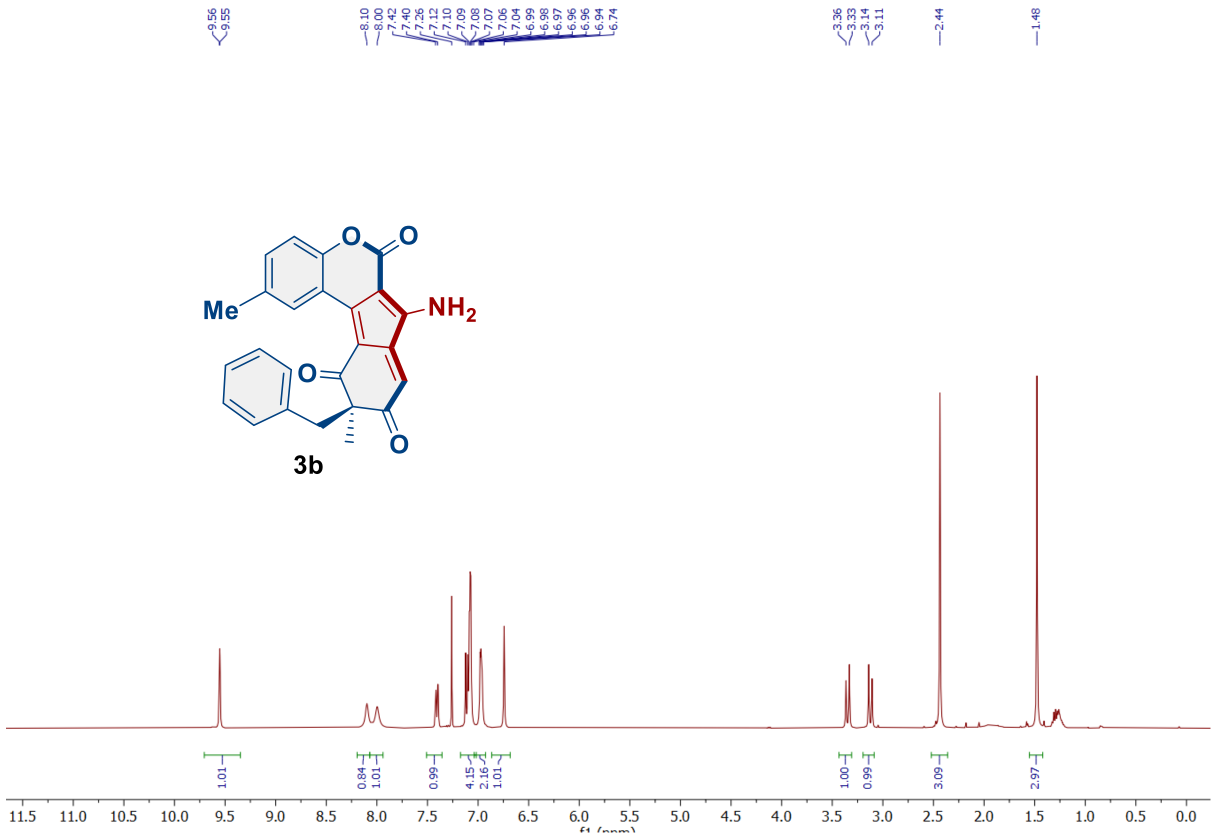

Supplementary Fig. 17. ^{1}H NMR (500 MHz, $CDCl_3$) spectrum for 3b.

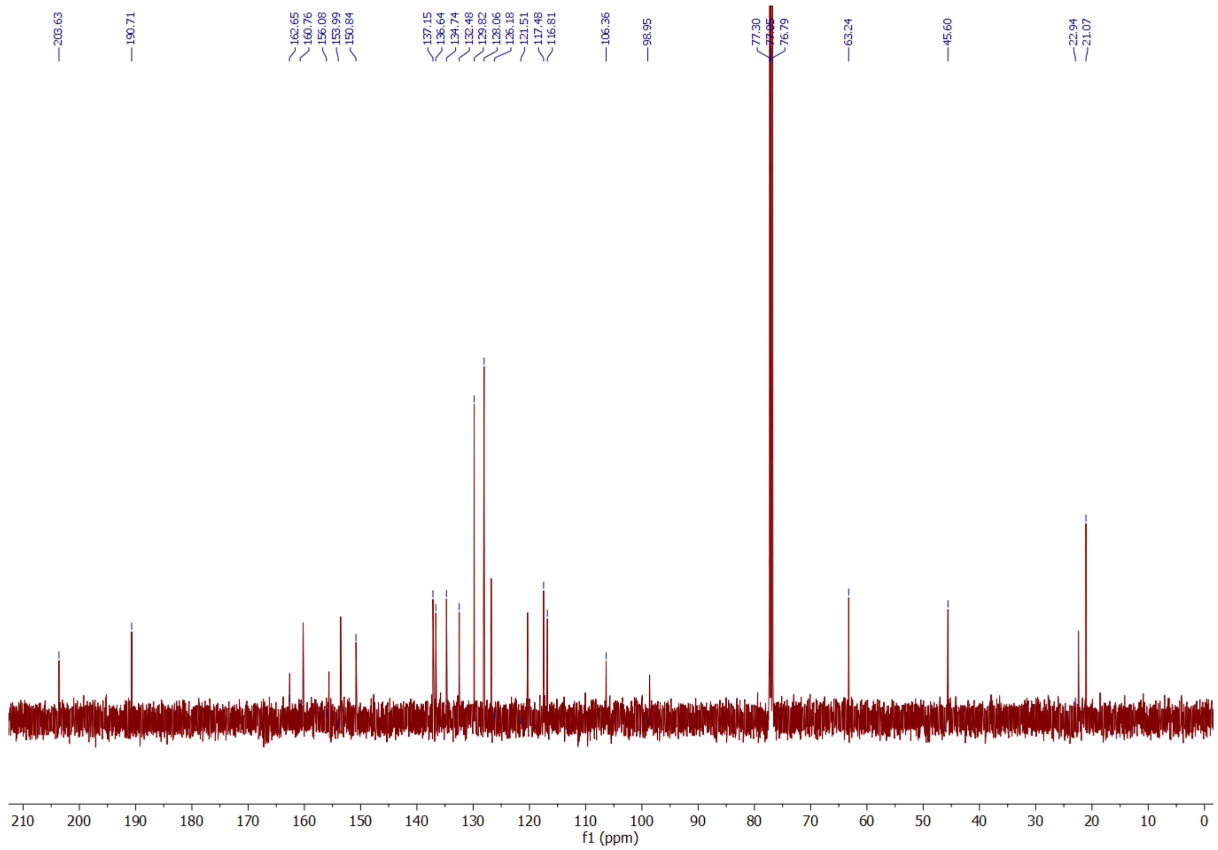

Supplementary Fig. 18. ^{13}C NMR (126 MHz, $CDCl_3$) spectrum for 3b.

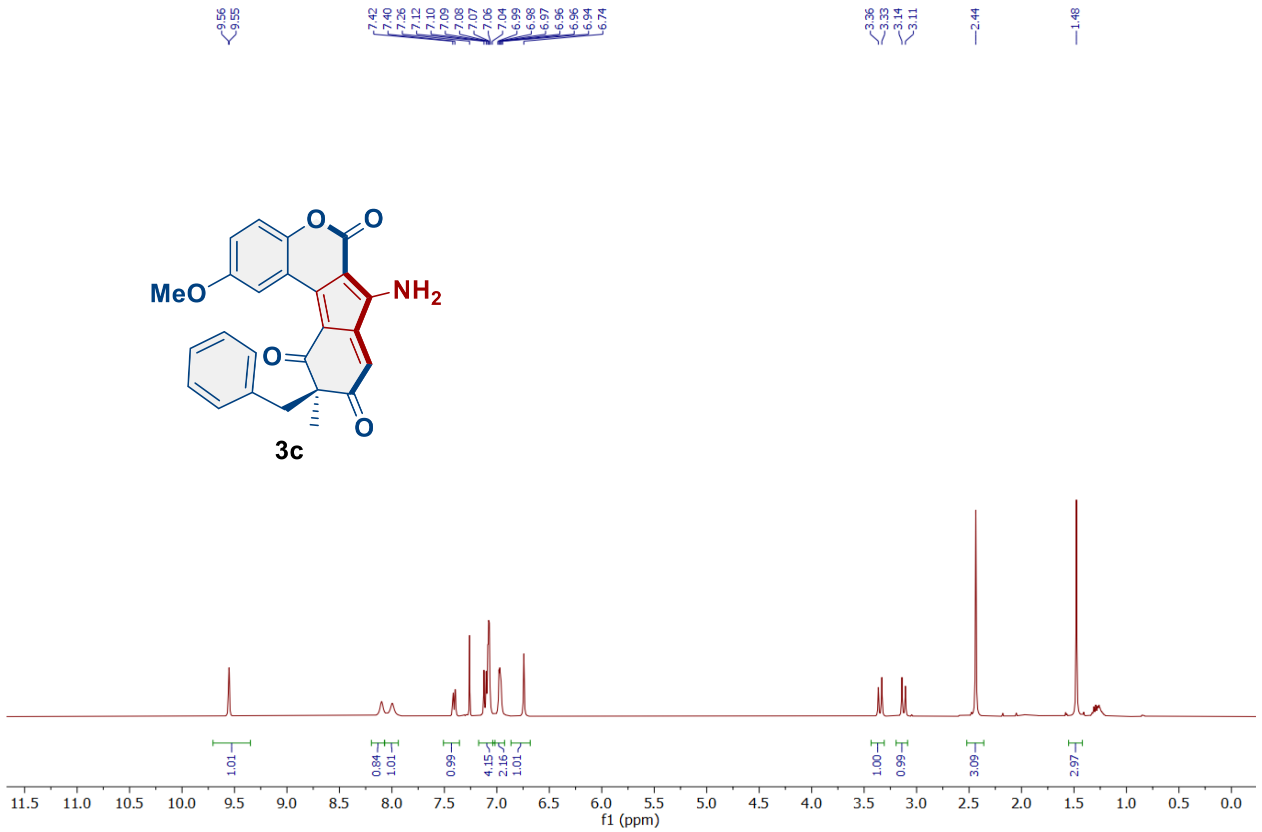

Supplementary Fig. 19. ^{1}H NMR (500 MHz, $CDCl_3$) spectrum for 3c.

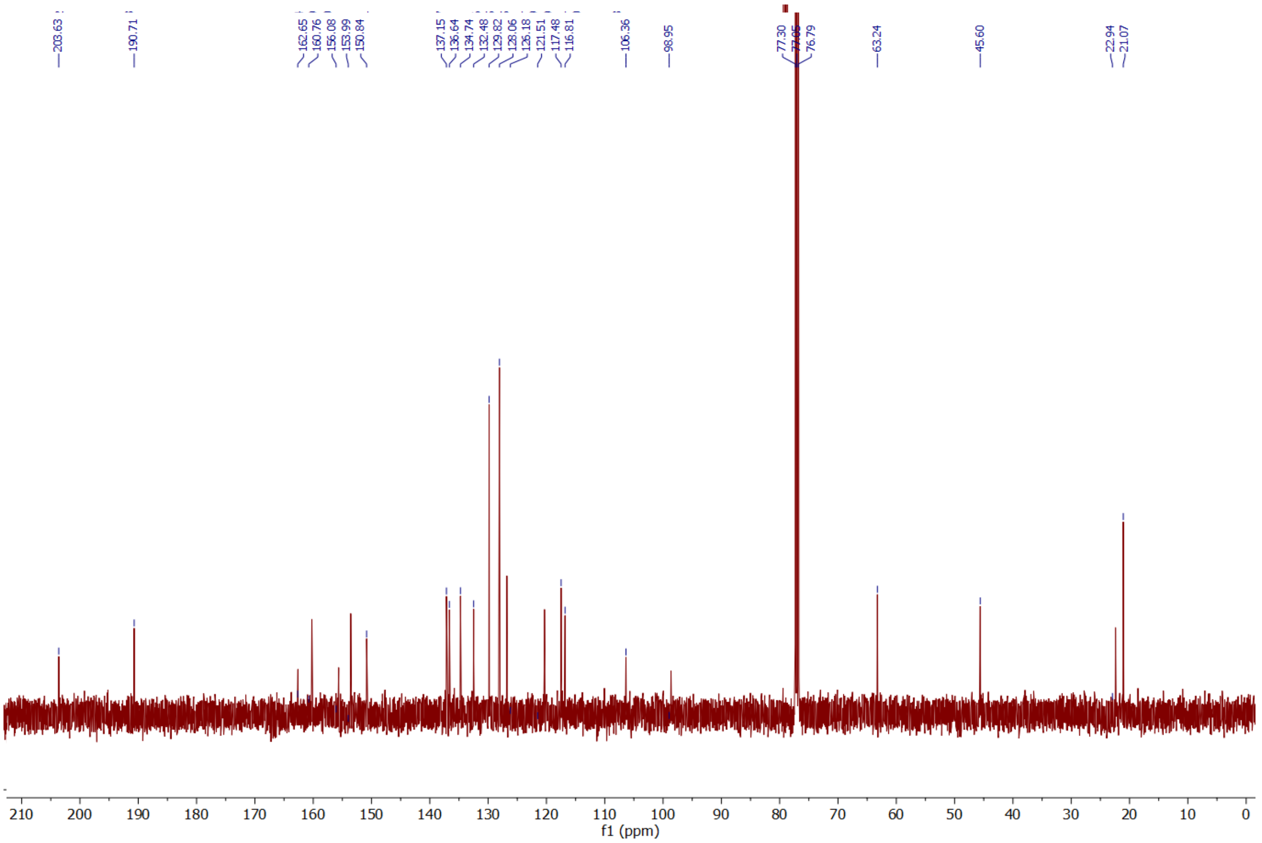

Supplementary Fig. 20. ^{13}C NMR (126 MHz, $CDCl_3$) spectrum for 3c.

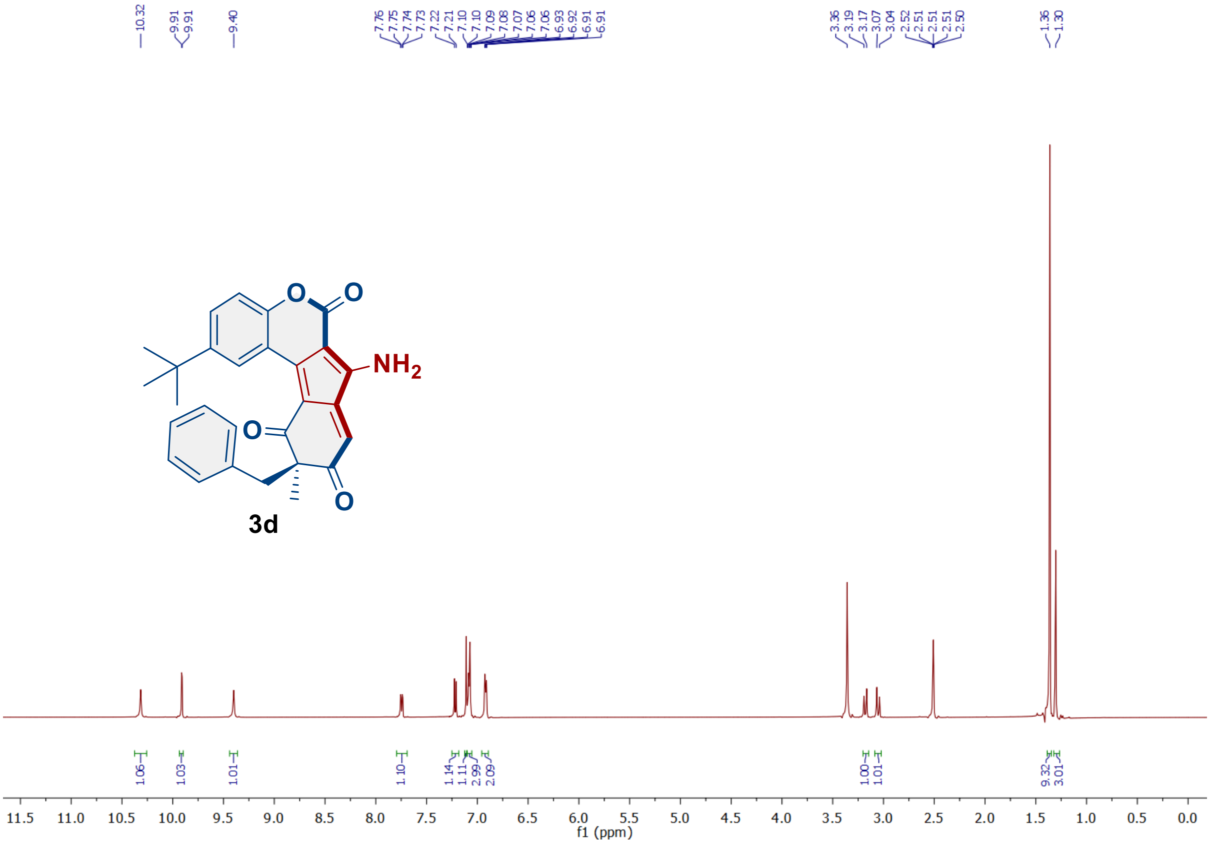

Supplementary Fig. 21. ^{1}H NMR (500 MHz, DMSO-d_6) spectrum for 3d.

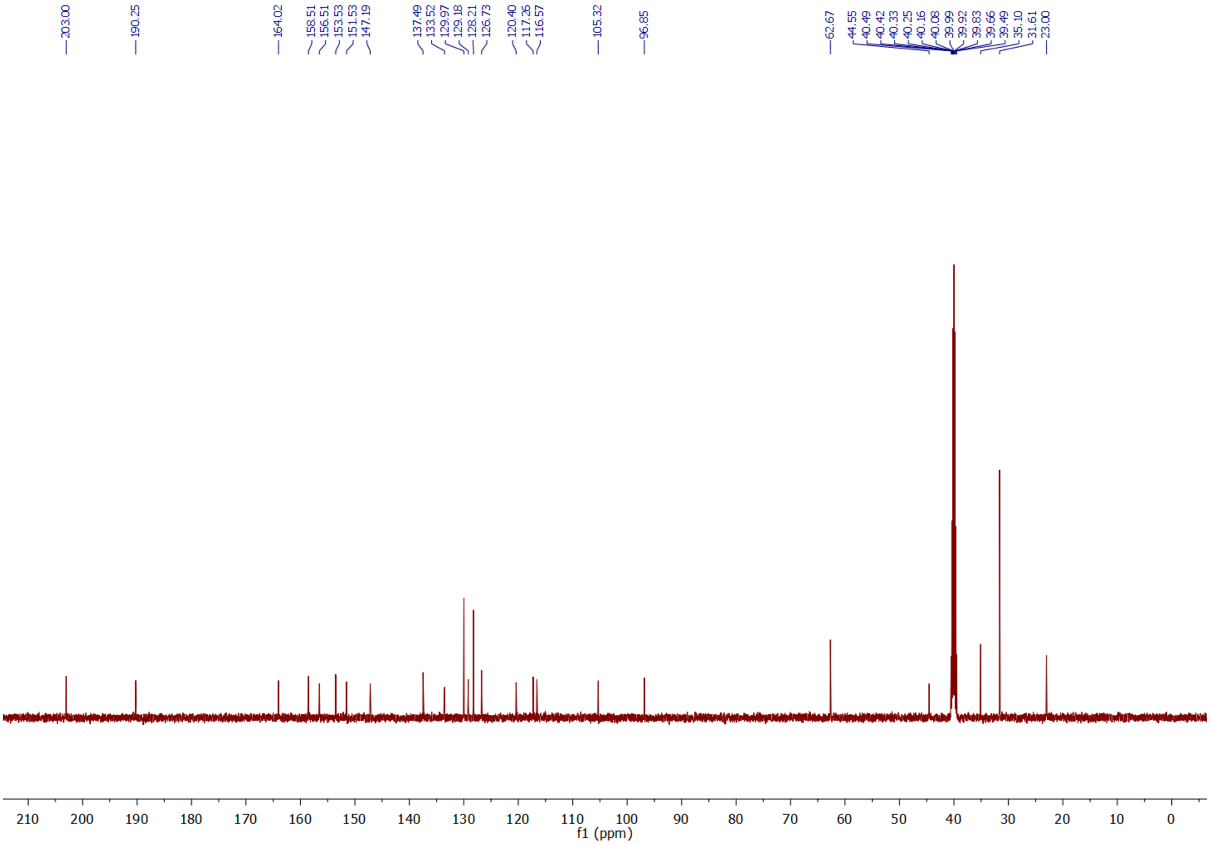

Supplementary Fig. 22. ^{13}C NMR (126 MHz, , DMSO-d_6) spectrum for 3d.

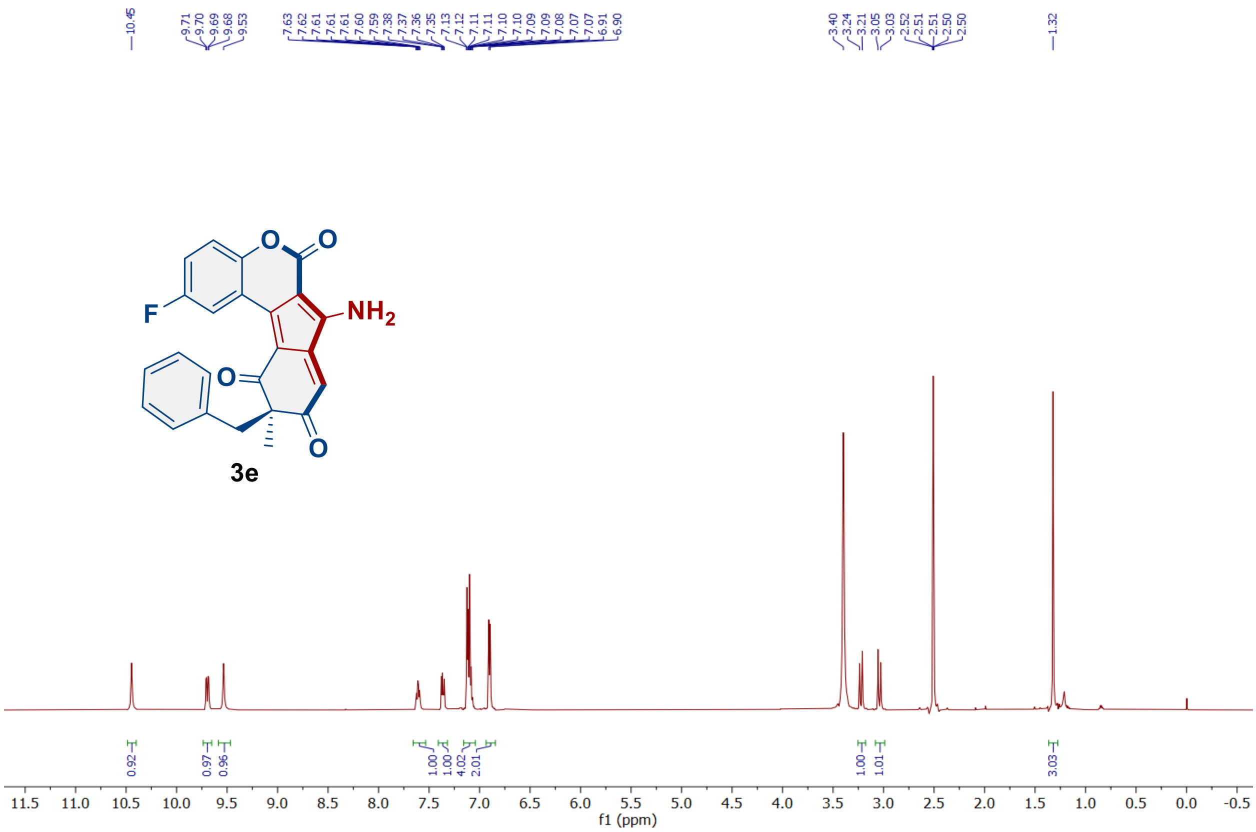

Supplementary Fig. 23. ^{1}H NMR (500 MHz, DMSO-d_6) spectrum for 3e.

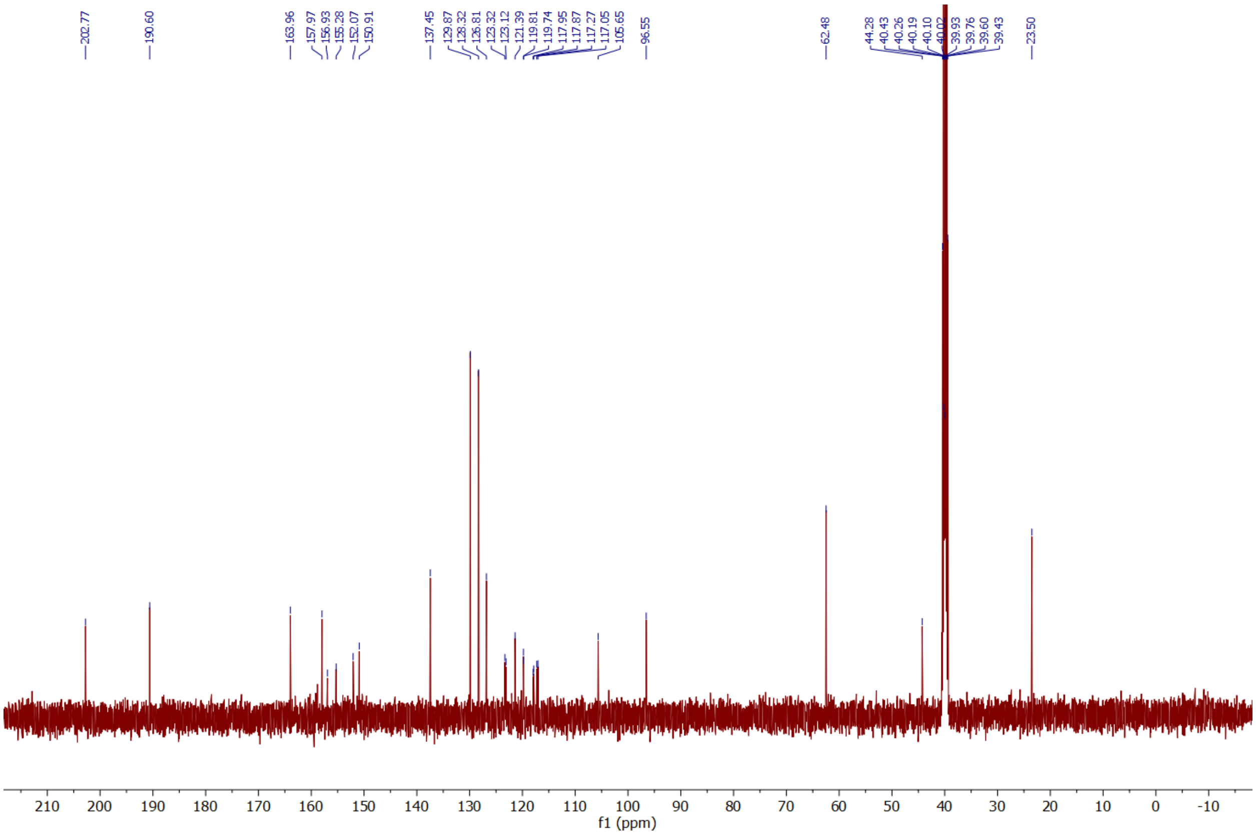

Supplementary Fig. 24. ^{13}C NMR (126 MHz, DMSO-d_6) spectrum for 3e.

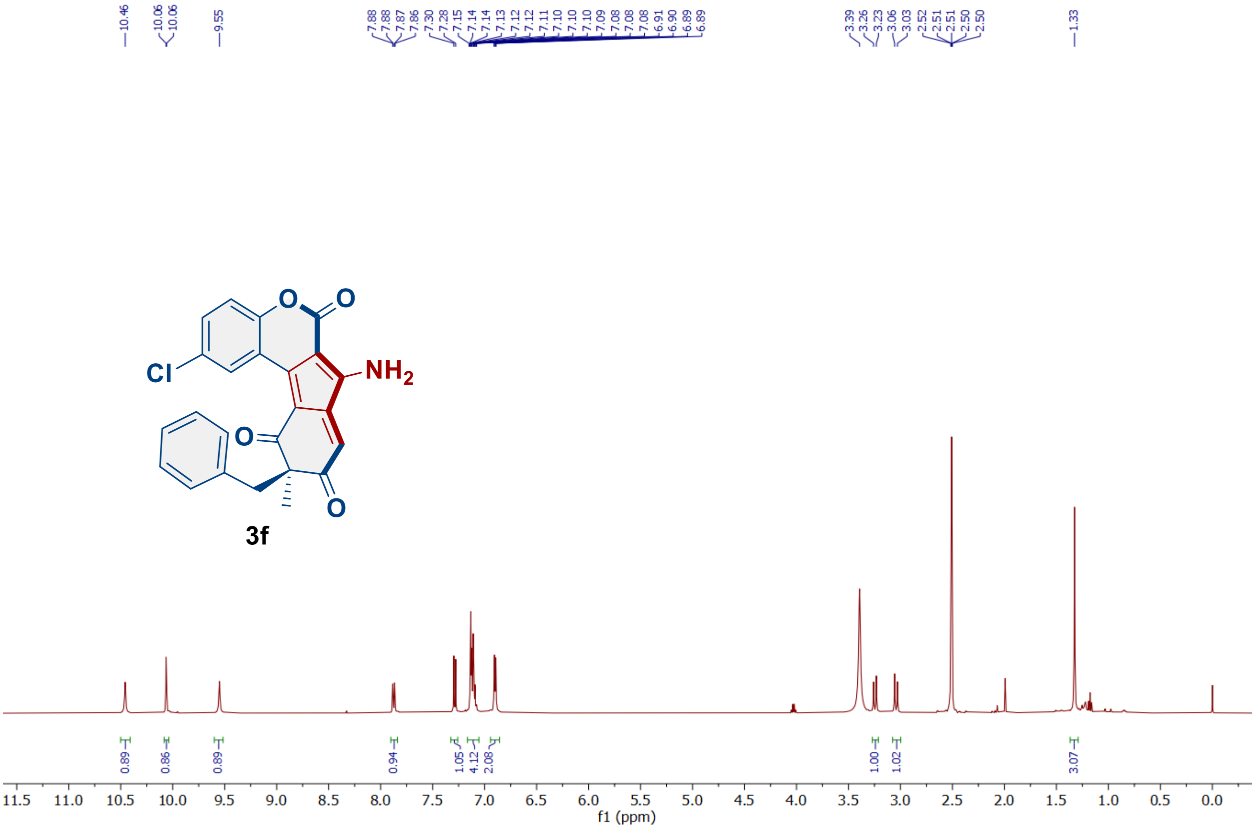

Supplementary Fig. 25. **^{1}H NMR (500 MHz, DMSO-d_6) spectrum for 3f.**

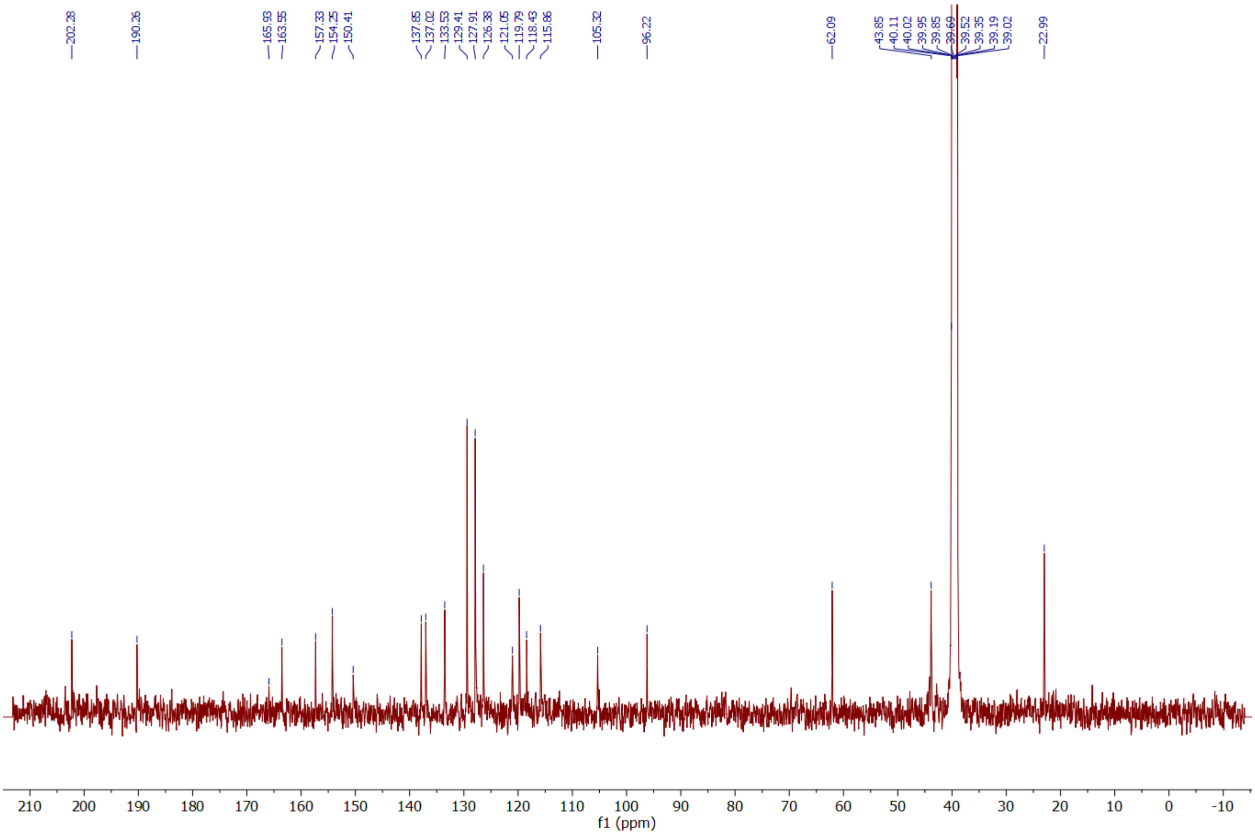

Supplementary Fig. 26. **^{13}C NMR (126 MHz, DMSO-d_6) spectrum for 3f.**

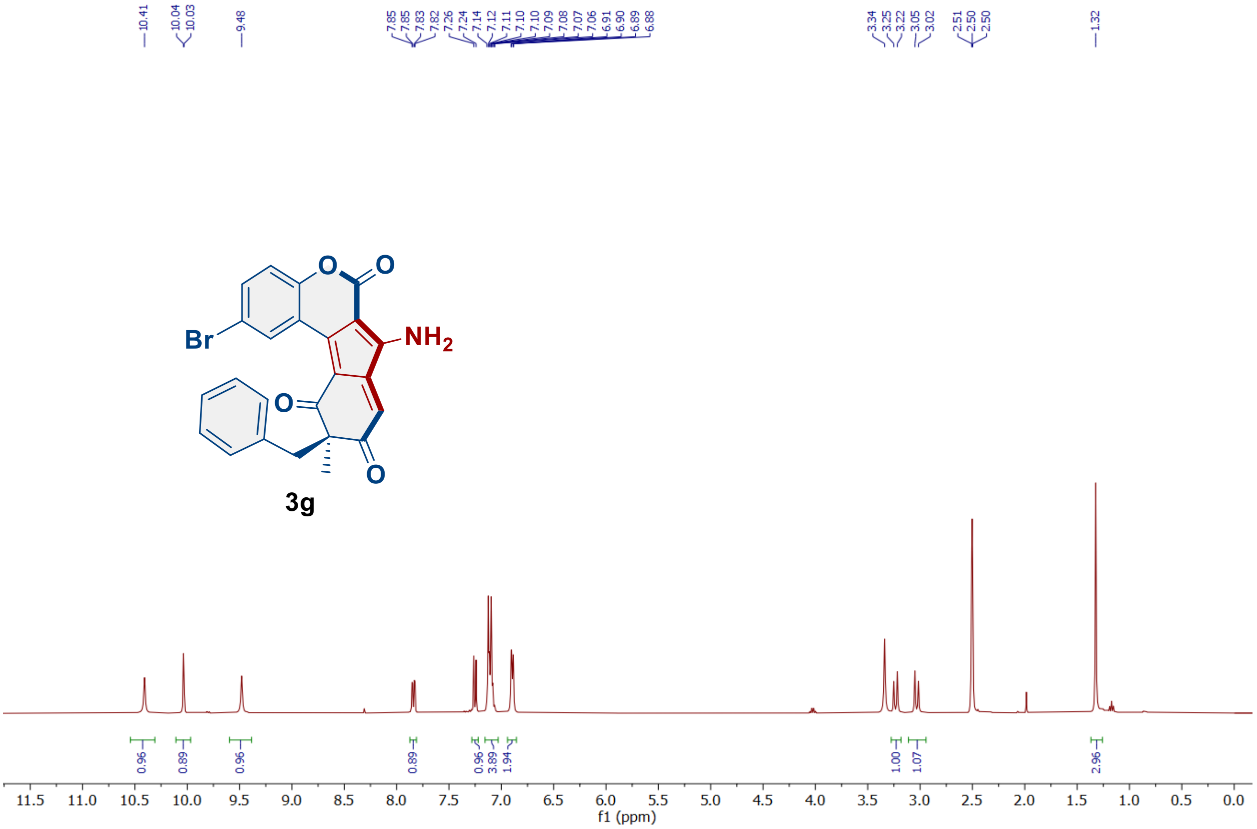

Supplementary Fig. 27. ^{1}H **NMR (500 MHz, DMSO-*d*$_6$) spectrum for 3g.**

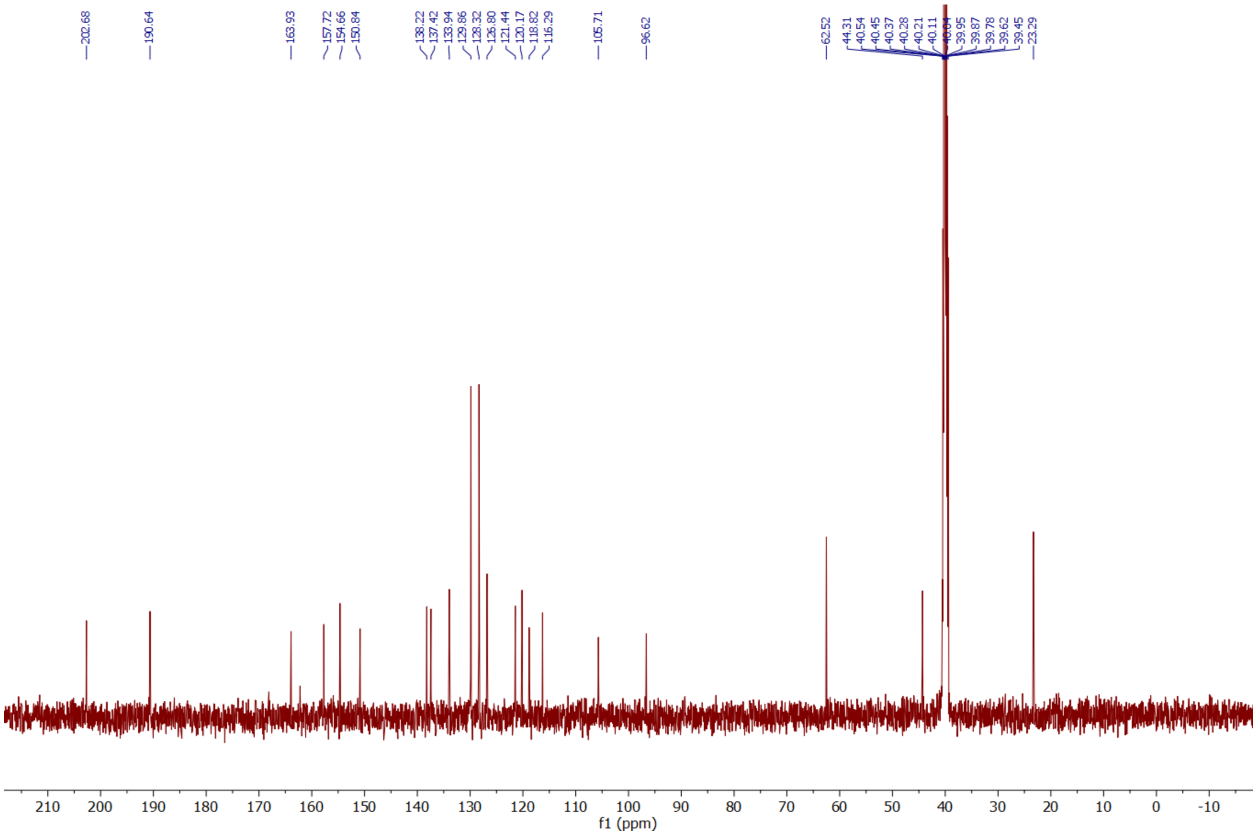

Supplementary Fig. 28. ^{13}C **NMR (126 MHz, DMSO-*d*$_6$) spectrum for 3g.**

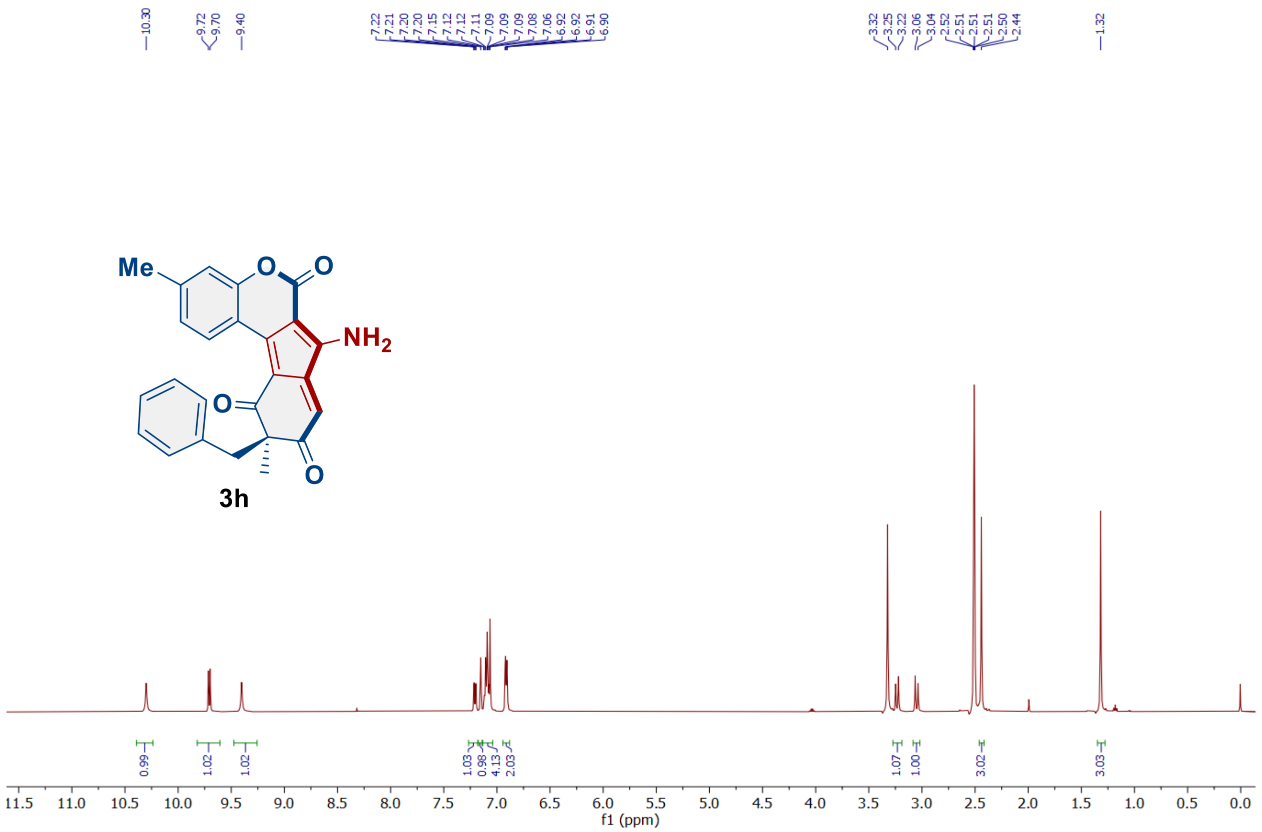

Supplementary Fig. 29. **^{1}H NMR (500 MHz, DMSO-d_6) spectrum for 3h.**

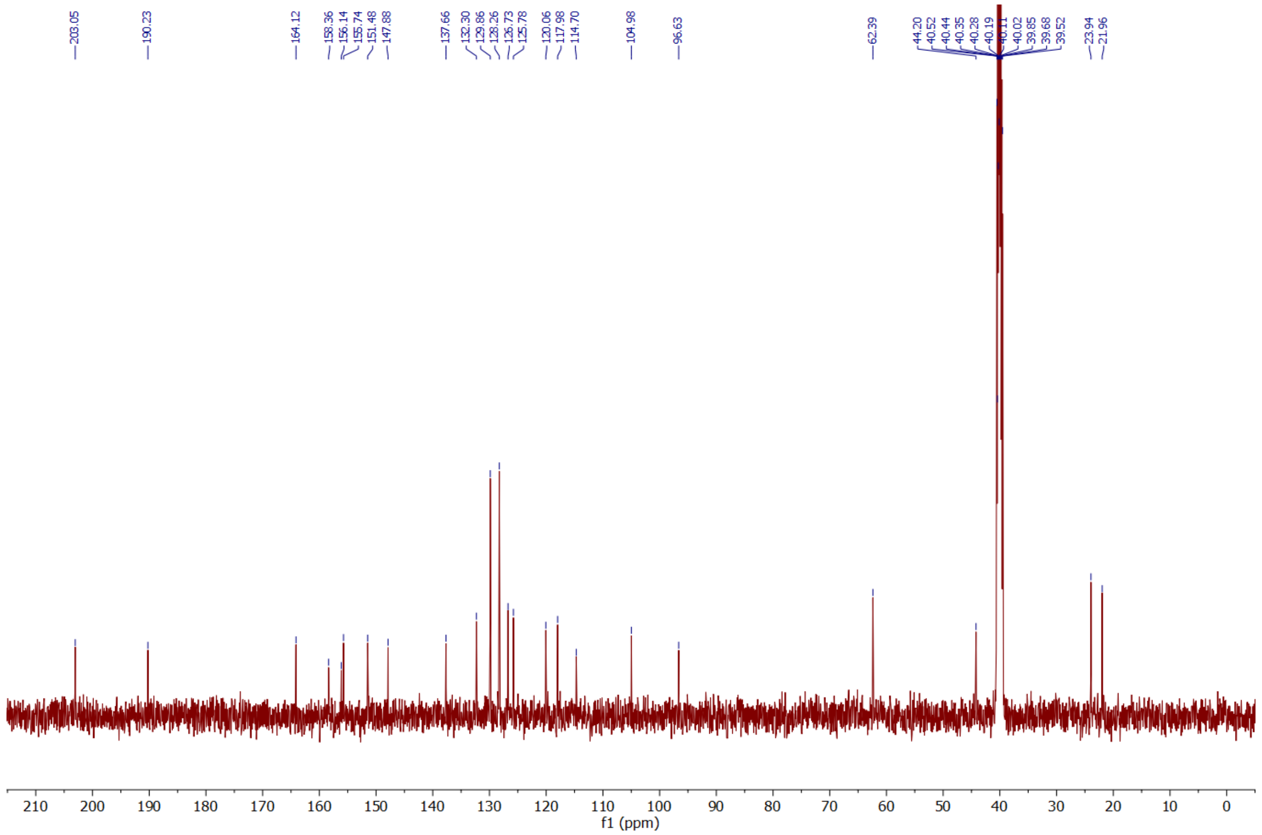

Supplementary Fig. 30. **^{13}C NMR (126 MHz, DMSO-d_6) spectrum for 3h.**

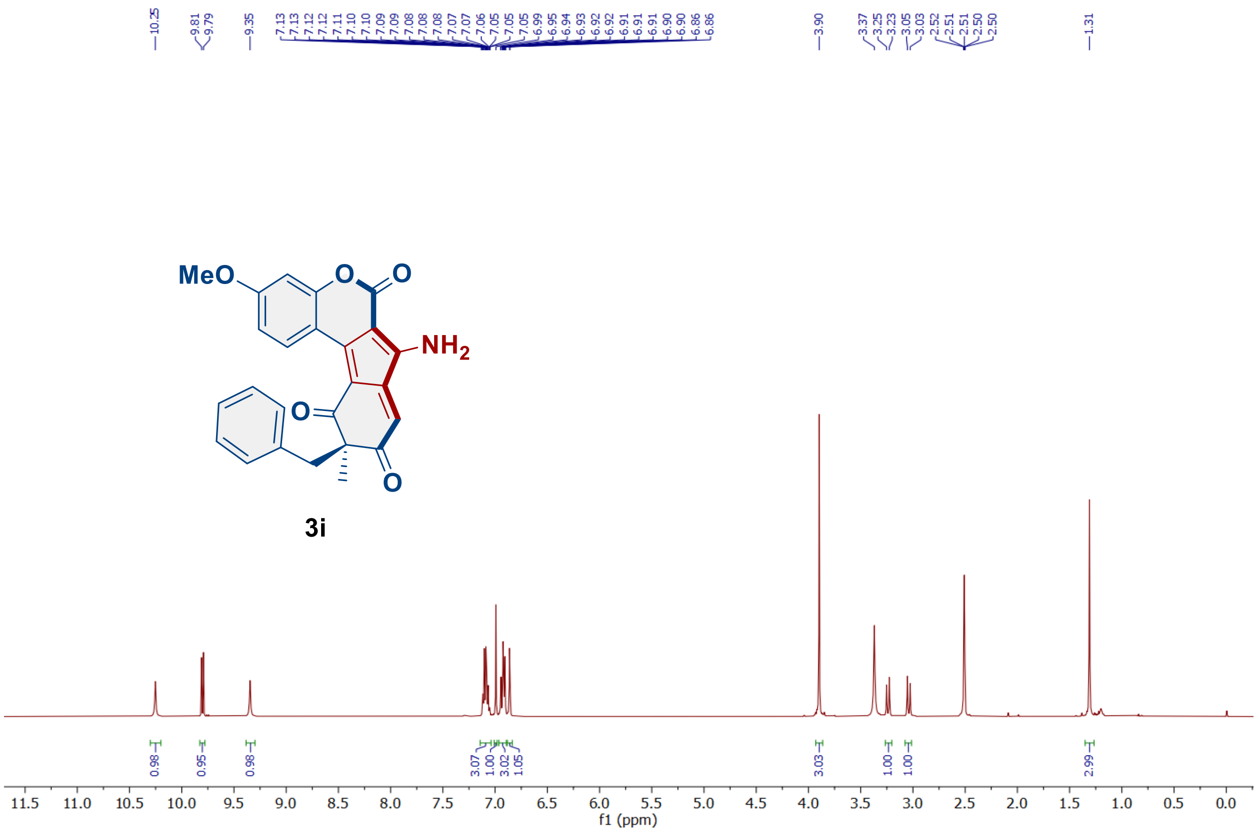

Supplementary Fig. 31. ^{1}H NMR (500 MHz, DMSO-d_6) spectrum for 3i.

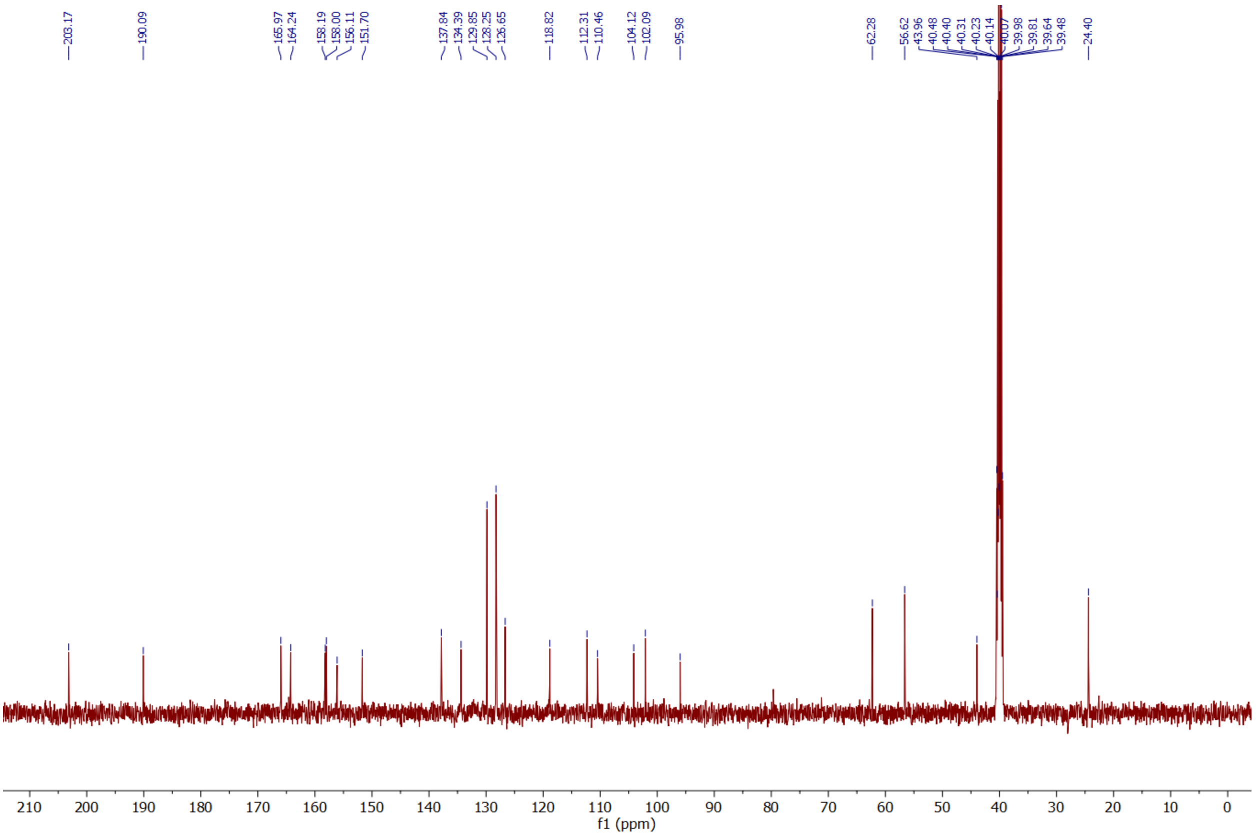

Supplementary Fig. 32. ^{13}C NMR (126 MHz, DMSO-d_6) spectrum for 3i.

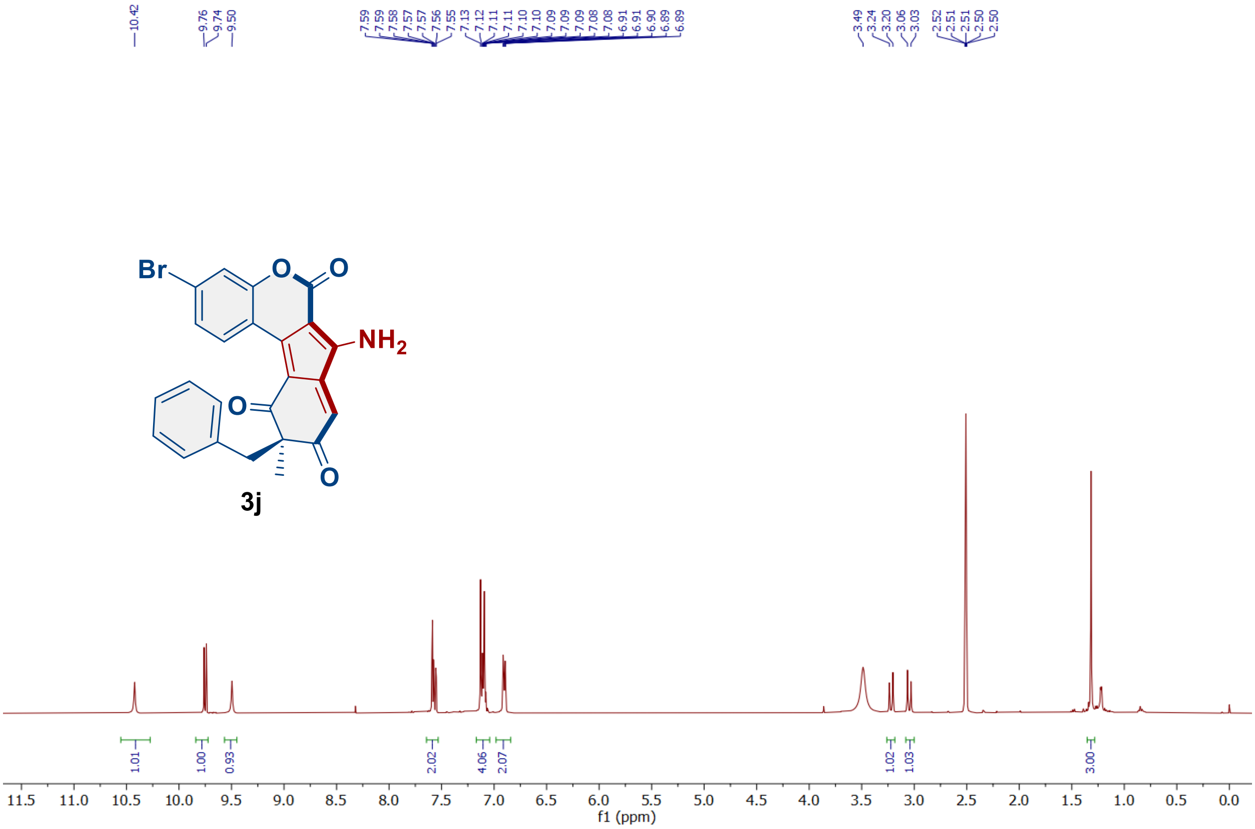

Supplementary Fig. 33. ^{1}**H NMR (500 MHz, DMSO-*d*$_6$) spectrum for 3j.**

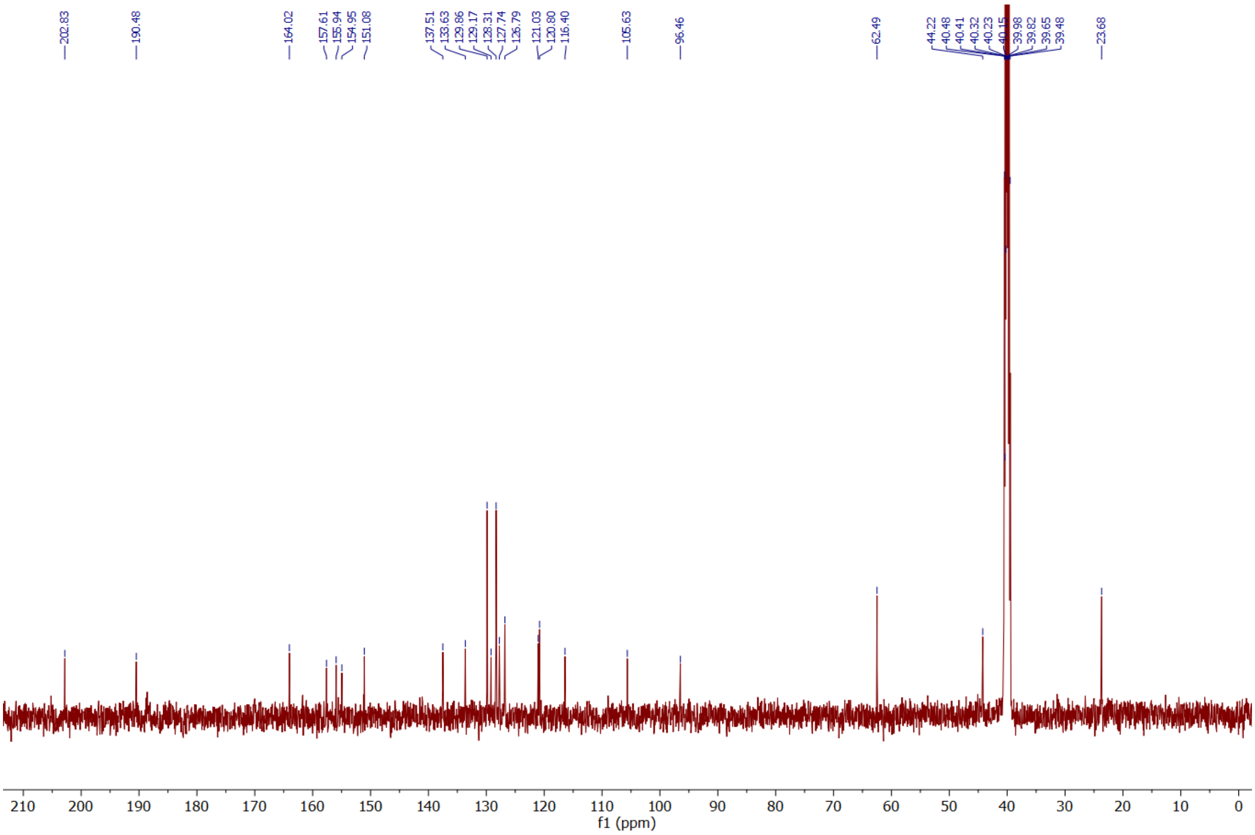

Supplementary Fig. 34. ^{13}**C NMR (126 MHz, DMSO-*d*$_6$) spectrum for 3j.**

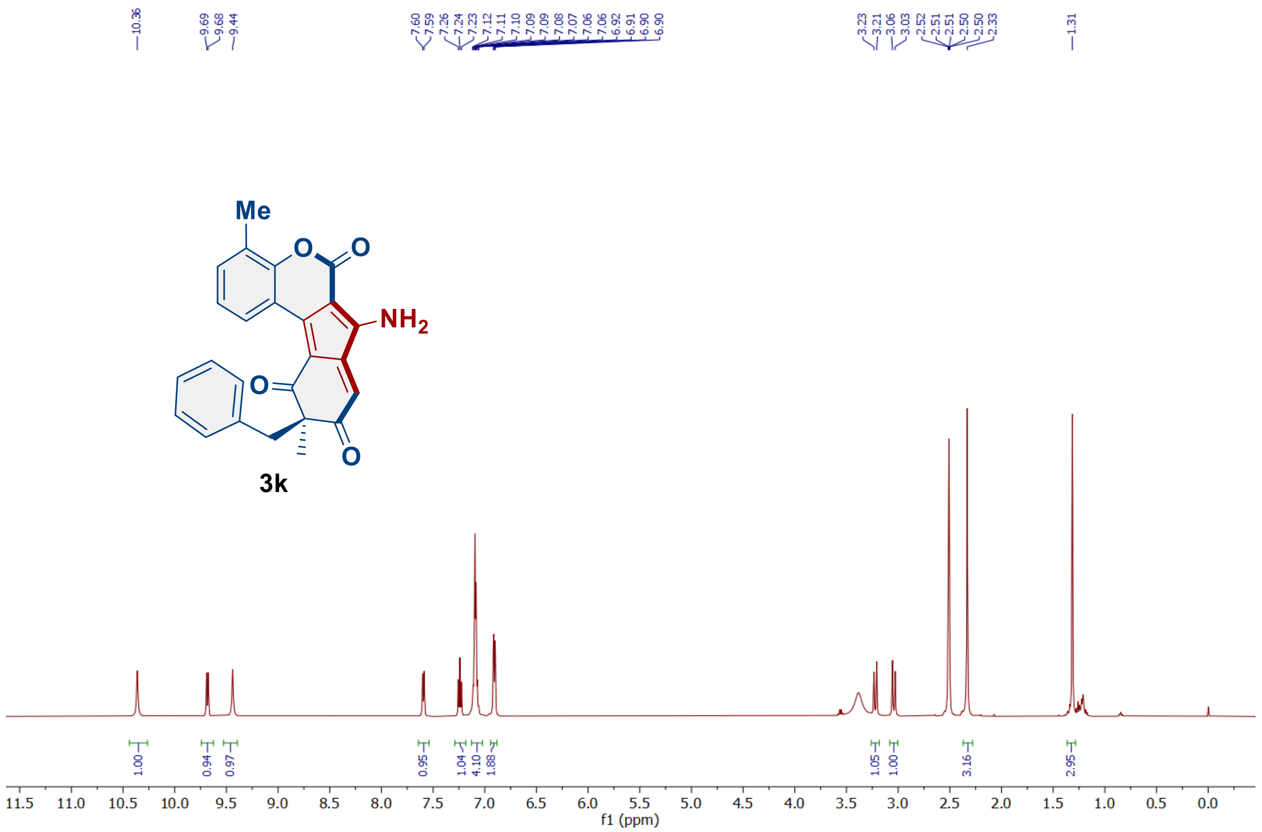

Supplementary Fig. 35. ^{1}H **NMR (500 MHz, DMSO-*d*$_6$) spectrum for 3k.**

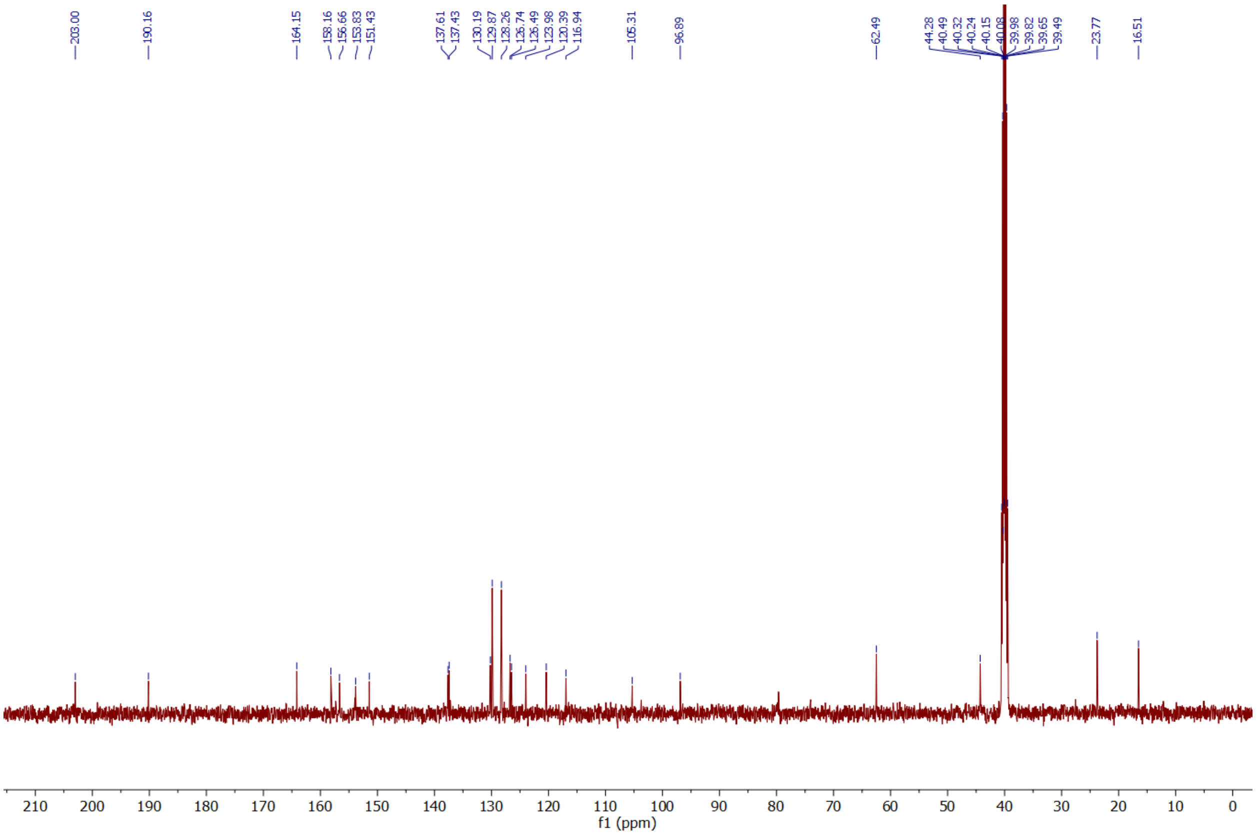

Supplementary Fig. 36. ^{13}C **NMR (126 MHz, DMSO-*d*$_6$) spectrum for 3k.**

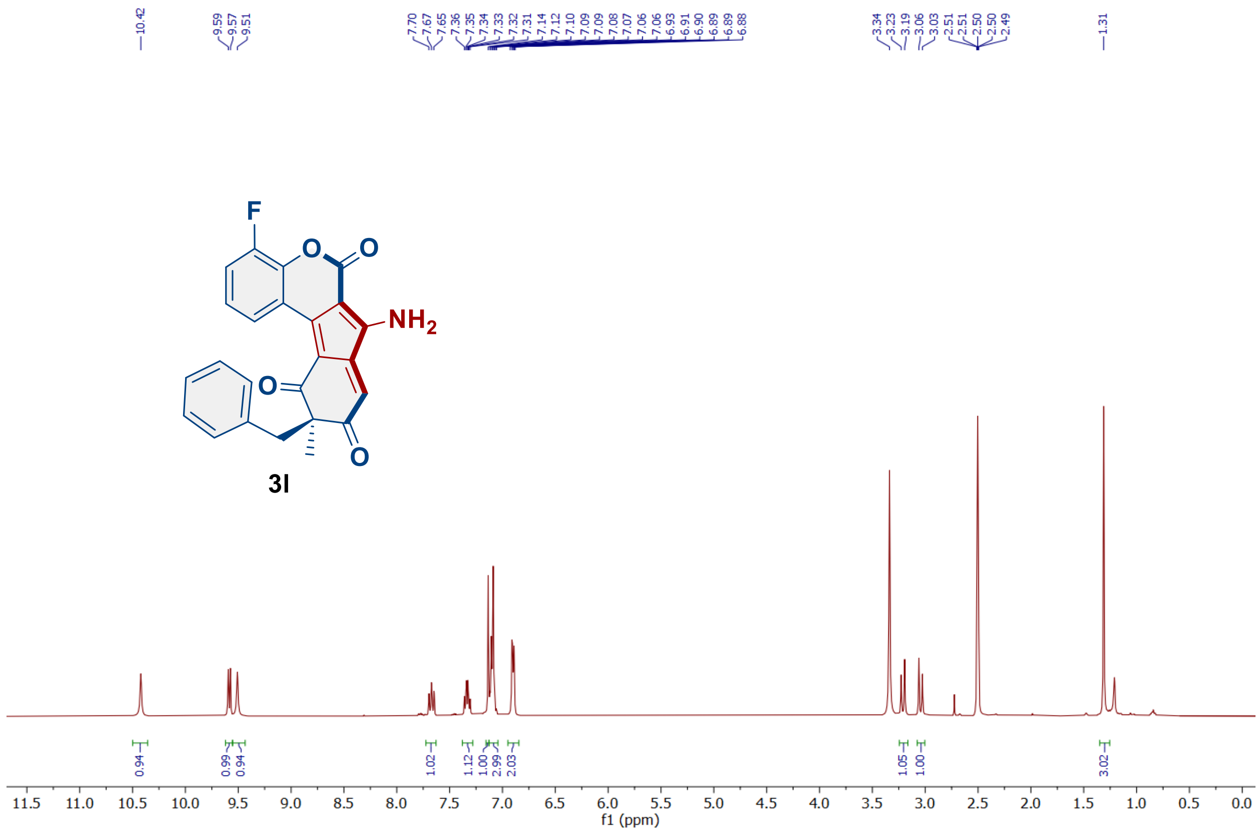

Supplementary Fig. 37. **^{1}H NMR (500 MHz, DMSO-d_6) spectrum for 3l.**

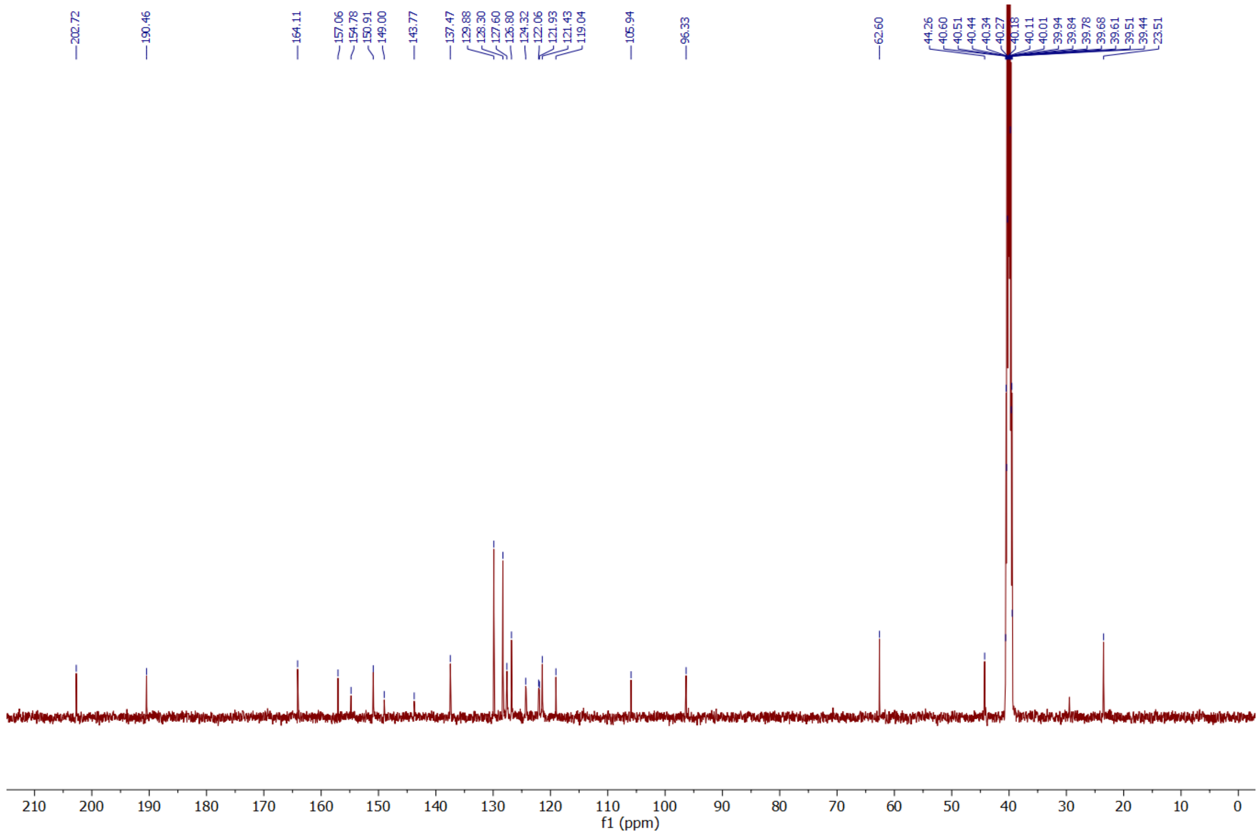

Supplementary Fig. 38. **^{13}C NMR (126 MHz, DMSO-d_6) spectrum for 3l.**

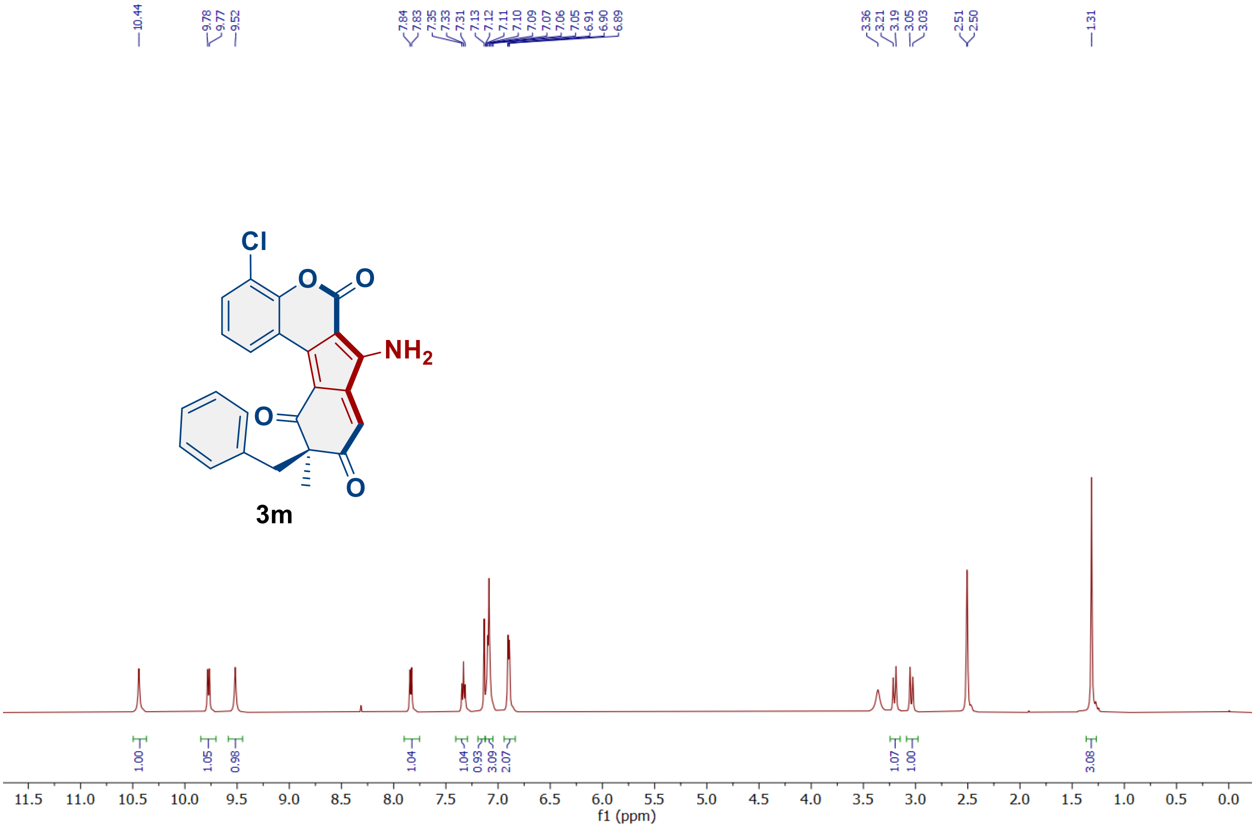

Supplementary Fig. 39. ^{1}**H NMR (500 MHz, DMSO-*d*$_6$) spectrum for 3m.**

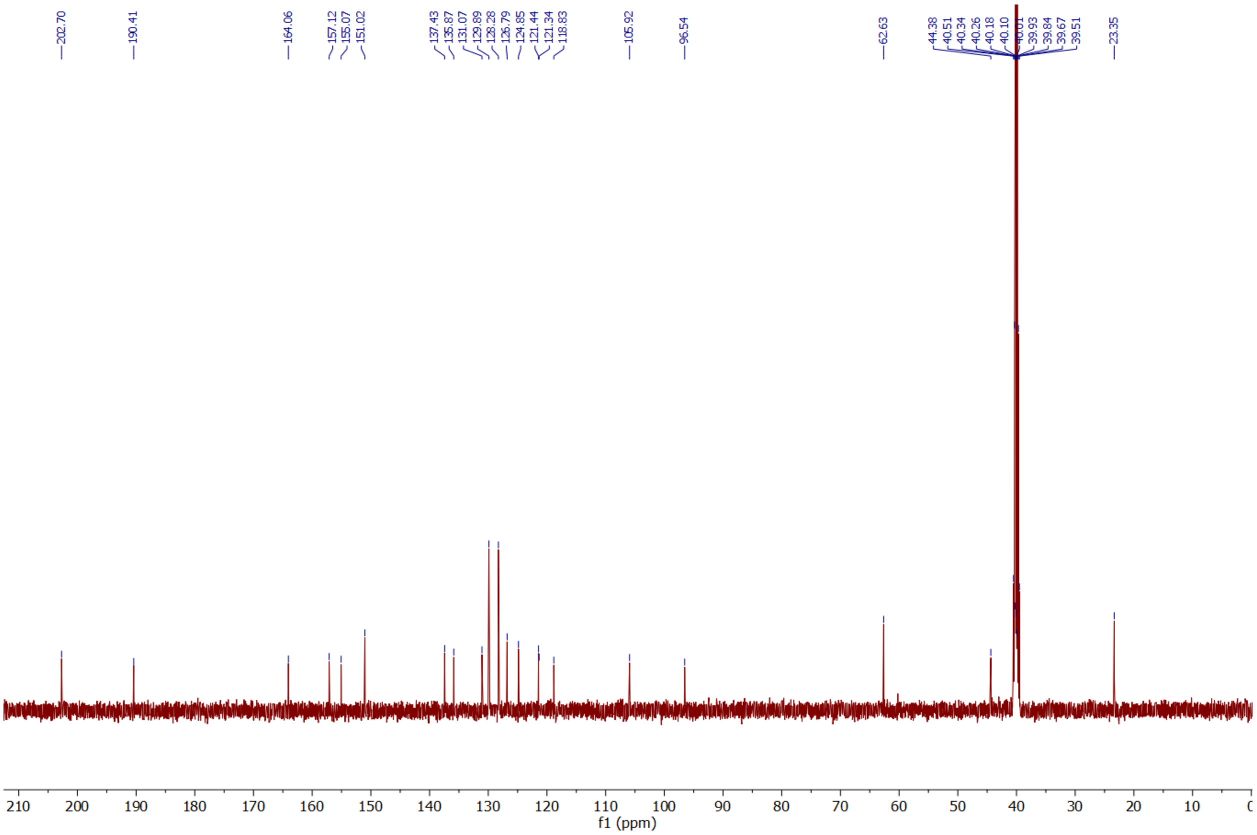

Supplementary Fig. 40. ^{13}**C NMR (126 MHz, DMSO-*d*$_6$) spectrum for 3m.**

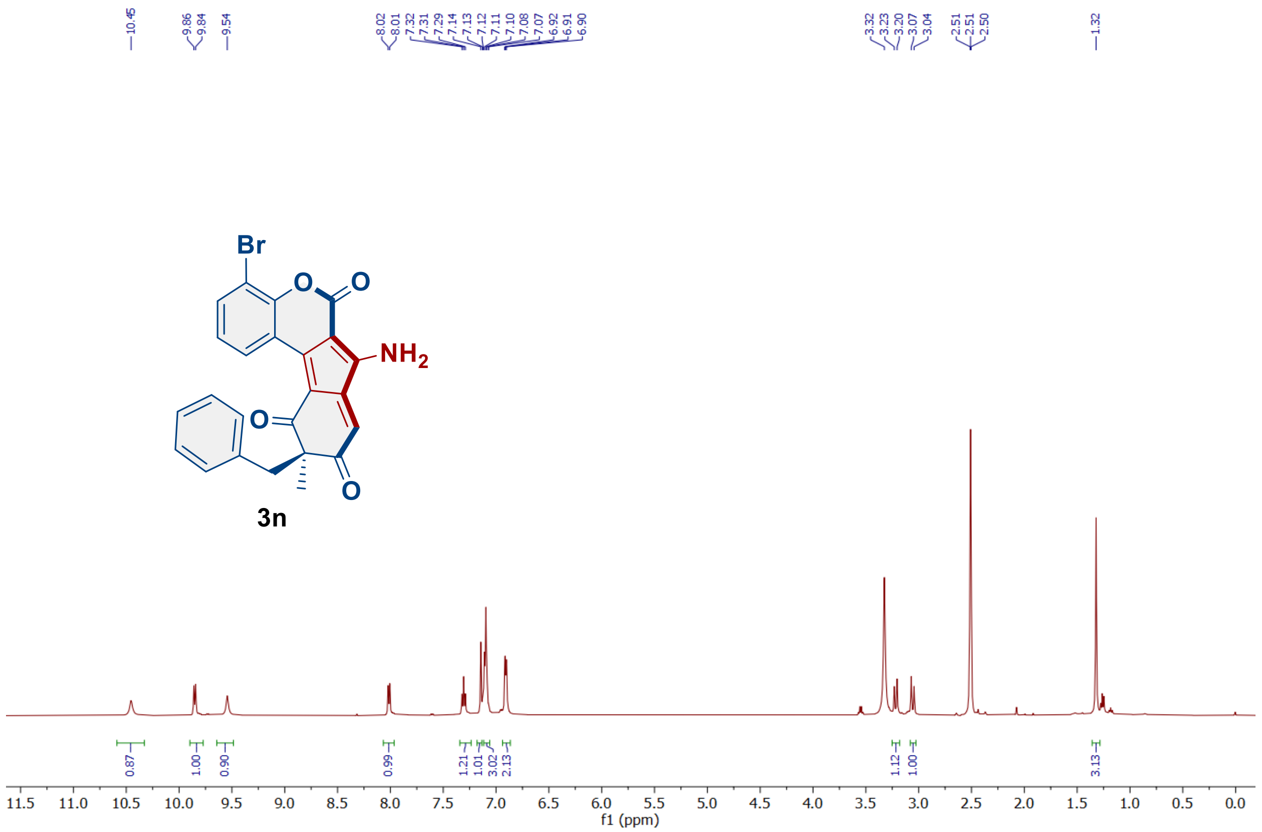

Supplementary Fig. 41. **^{1}H NMR (500 MHz, DMSO-*d*$_6$) spectrum for 3n.**

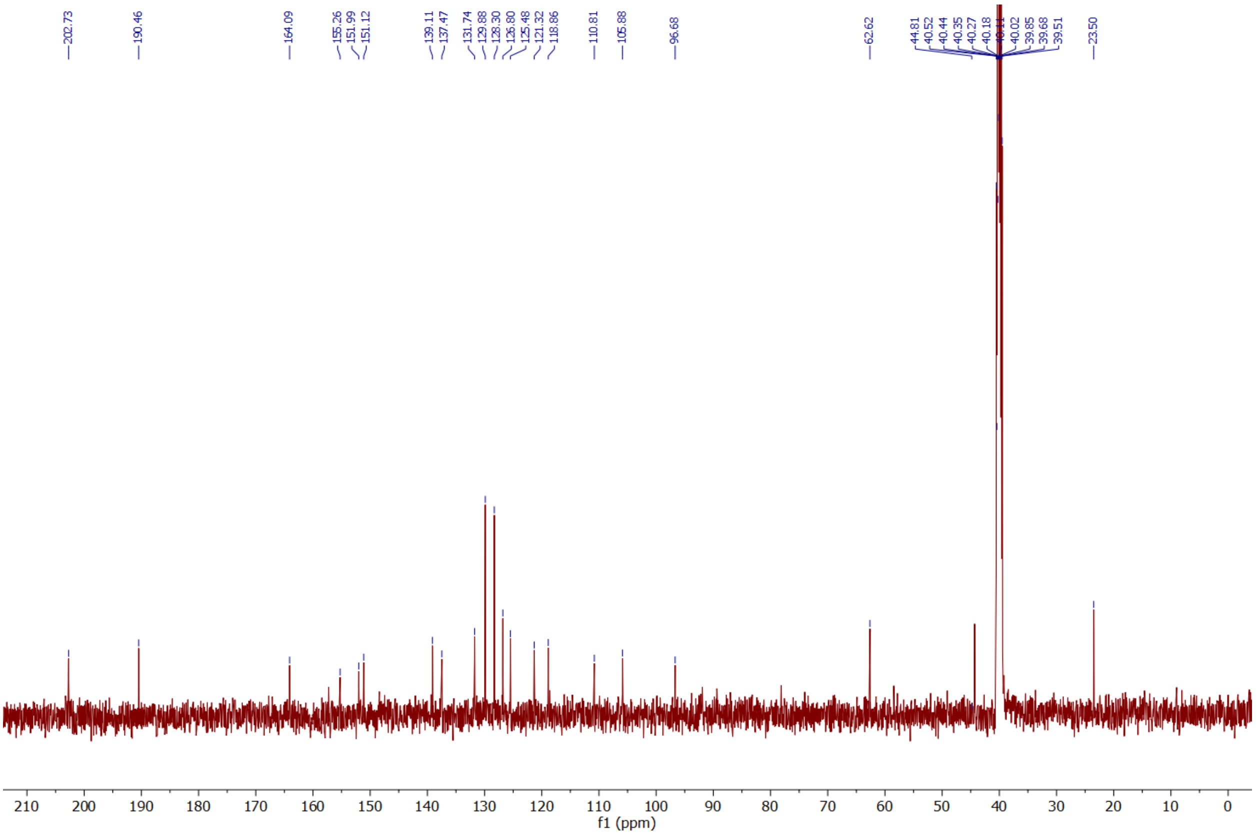

Supplementary Fig. 42. **^{13}C NMR (126 MHz, DMSO-*d*$_6$) spectrum for 3n.**

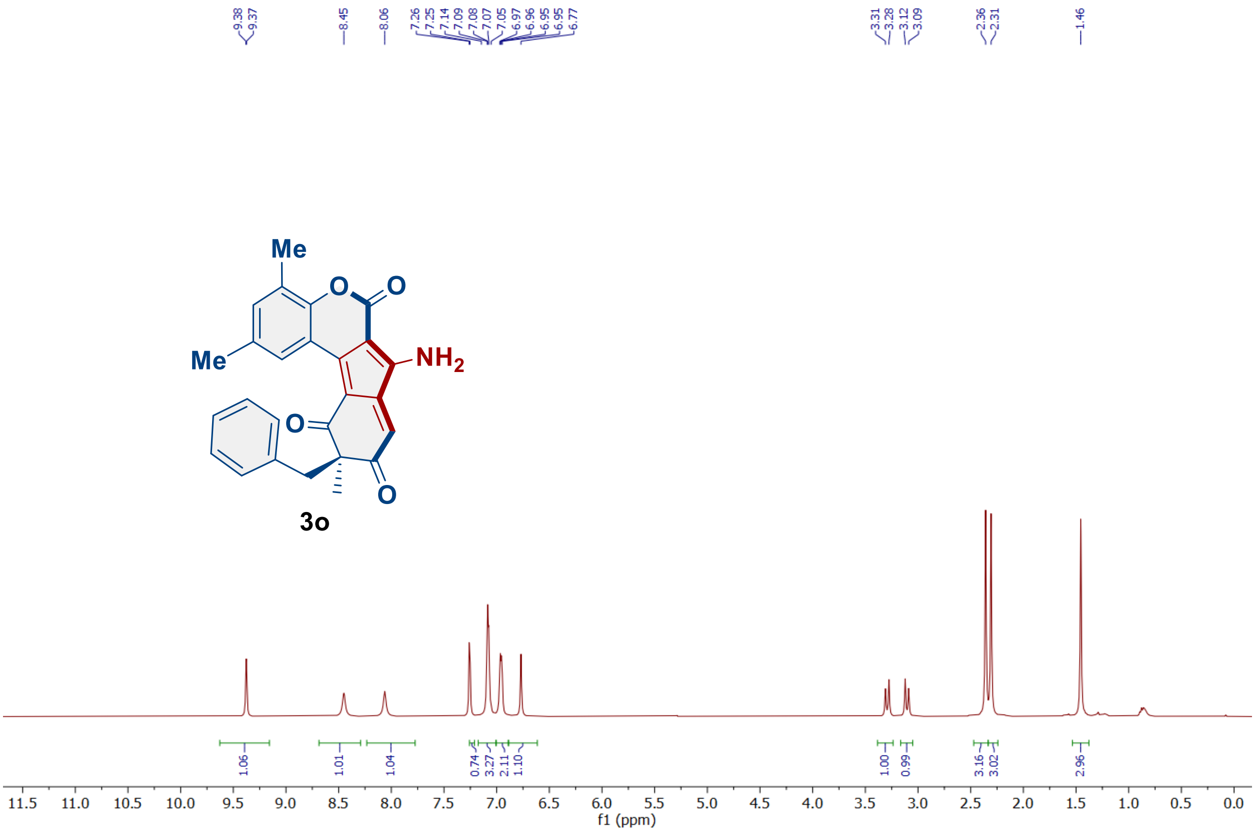

Supplementary Fig. 43. ^{1}**H NMR (500 MHz, CDCl$_3$) spectrum for 3o.**

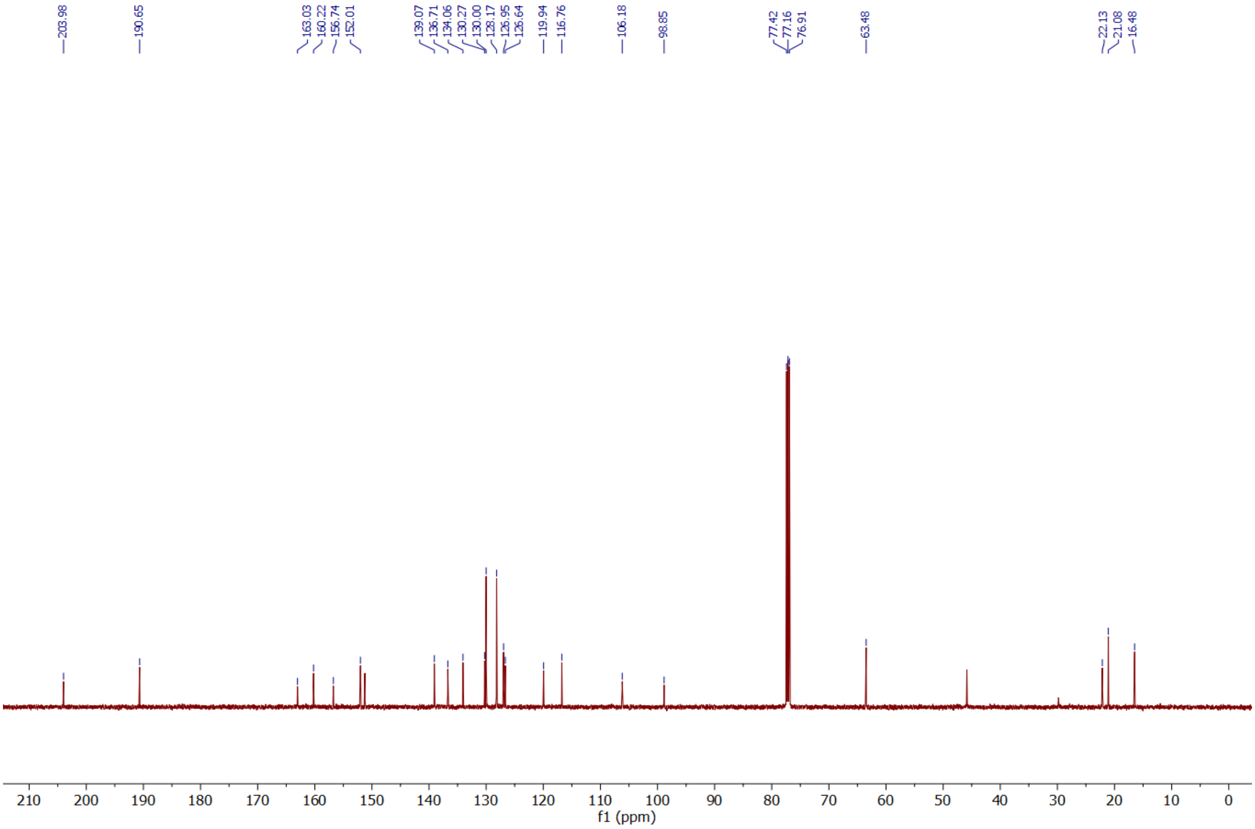

Supplementary Fig. 44. ^{13}**C NMR (126 MHz, CDCl$_3$) spectrum for 3o.**

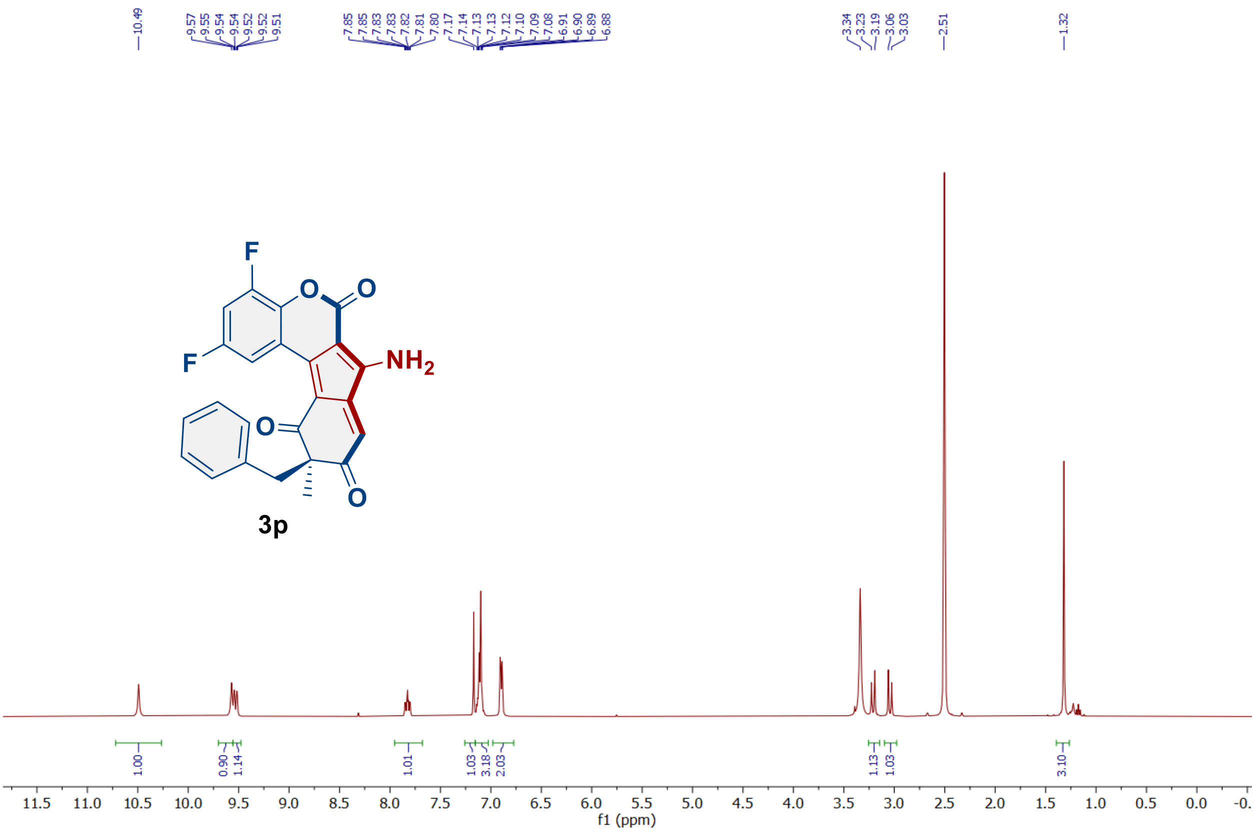

Supplementary Fig. 45. **^{1}H NMR (500 MHz, DMSO-d_6) spectrum for 3p.**

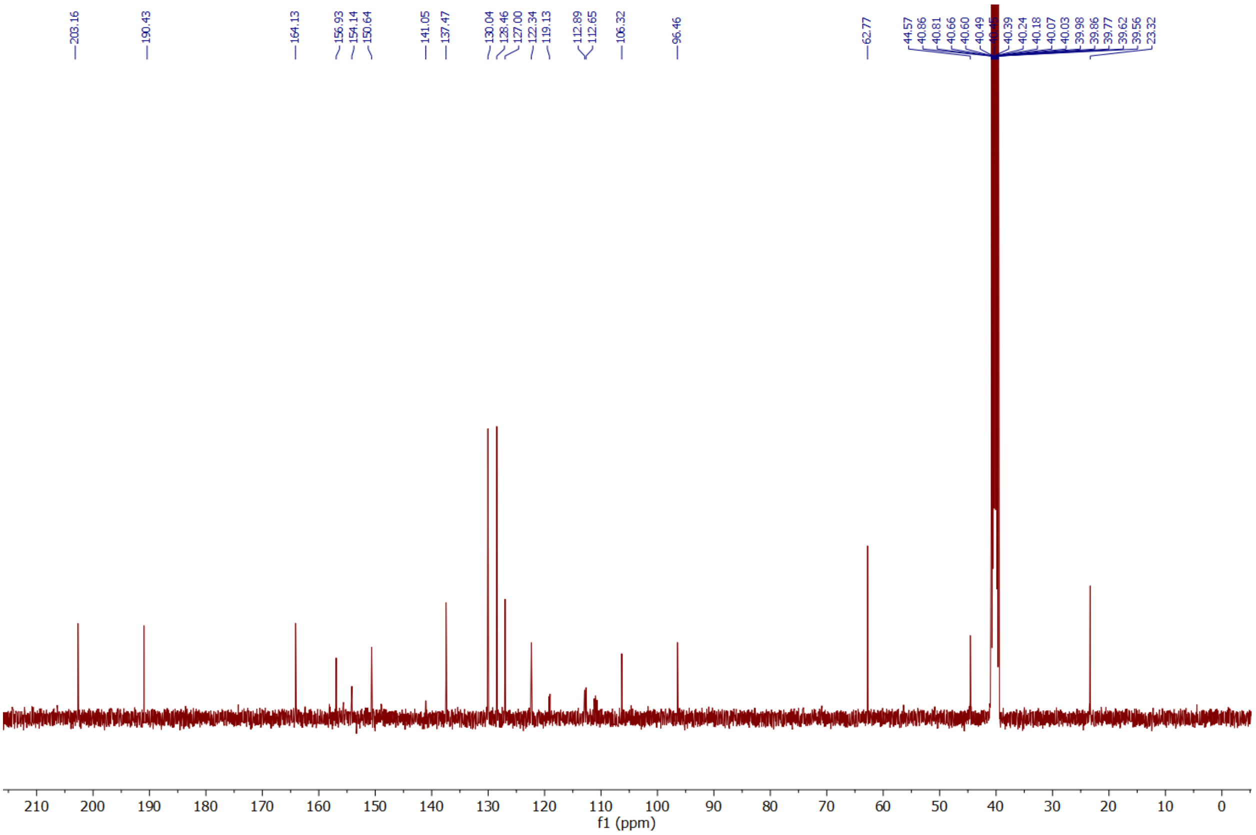

Supplementary Fig. 46. **^{13}C NMR (126 MHz, DMSO-d_6) spectrum for 3p.**

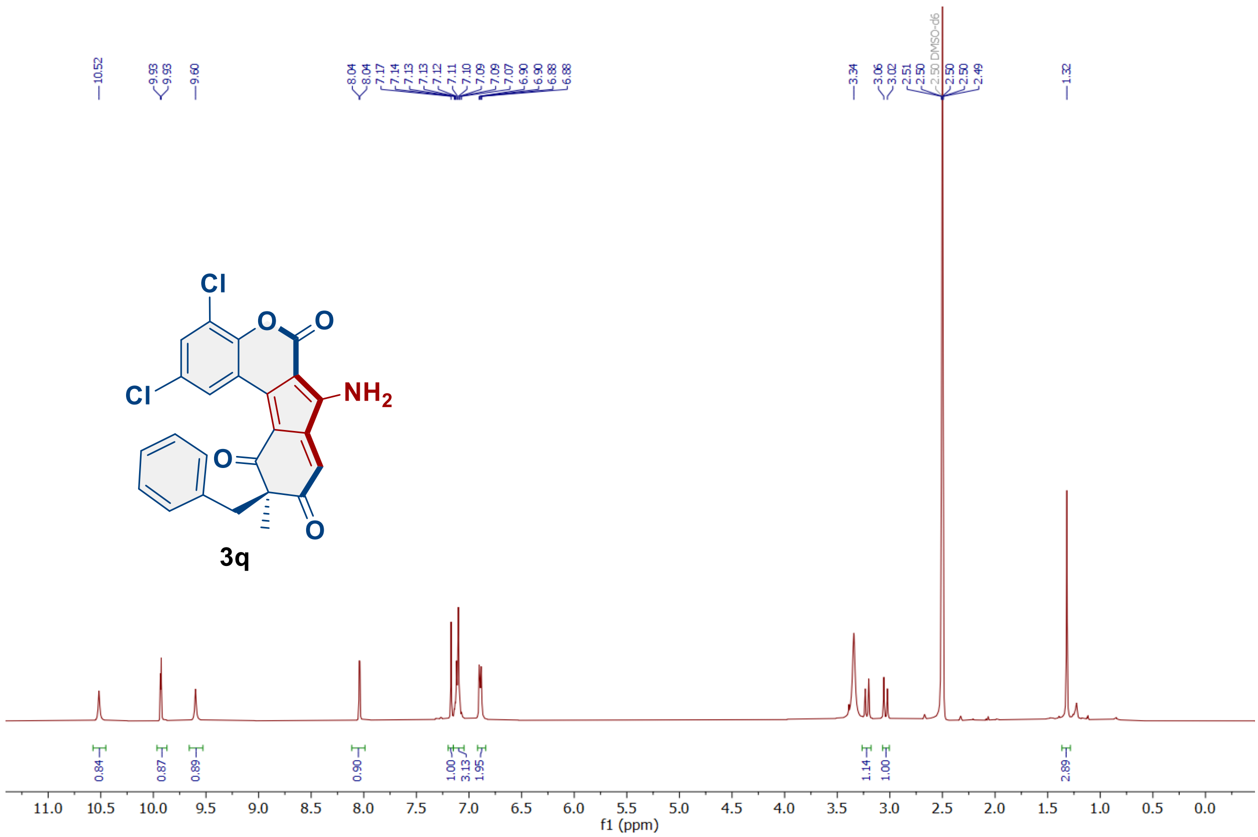

Supplementary Fig. 47. ^{1}**H NMR (500 MHz, DMSO-d_6) spectrum for 3q.**

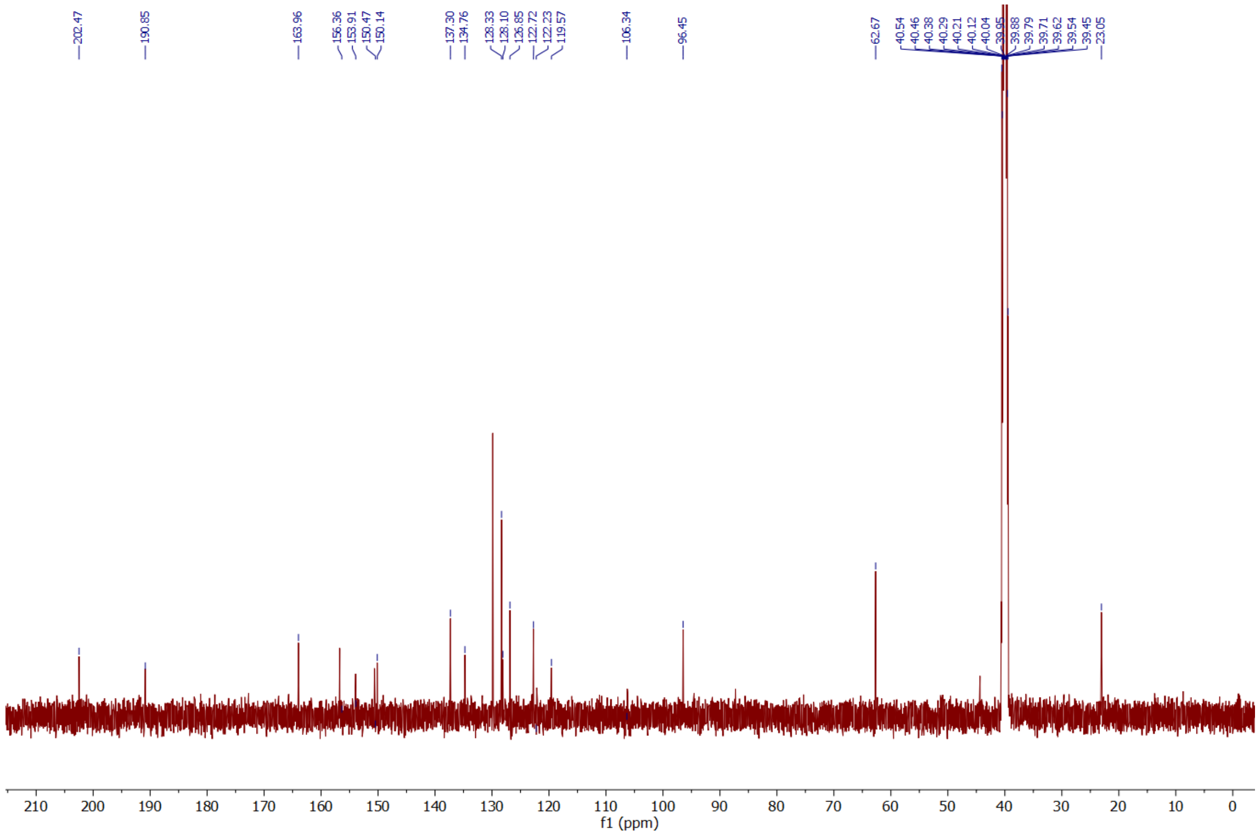

Supplementary Fig. 48. ^{13}**C NMR (126 MHz, DMSO-d_6) spectrum for 3q.**

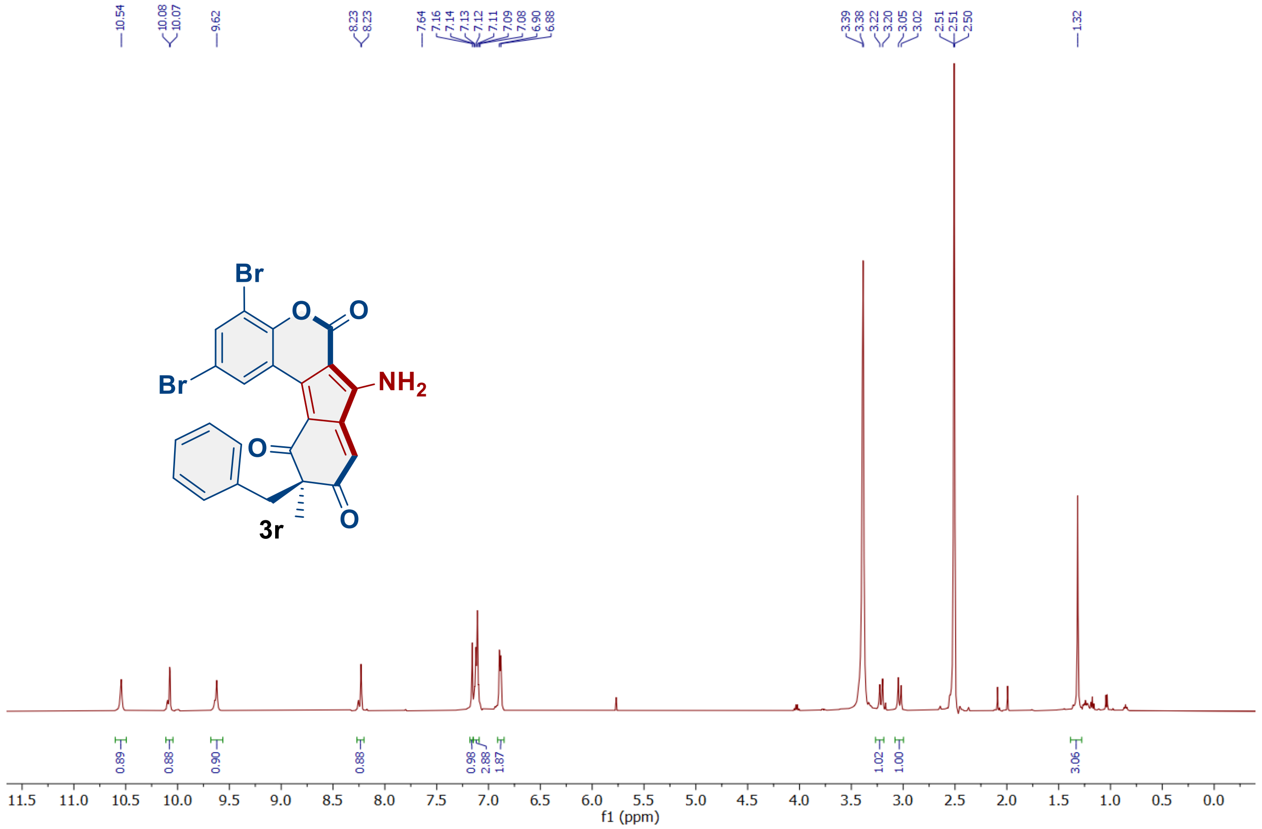

Supplementary Fig. 49. ^{1}H **NMR (500 MHz, DMSO-d_6) spectrum for 3r.**

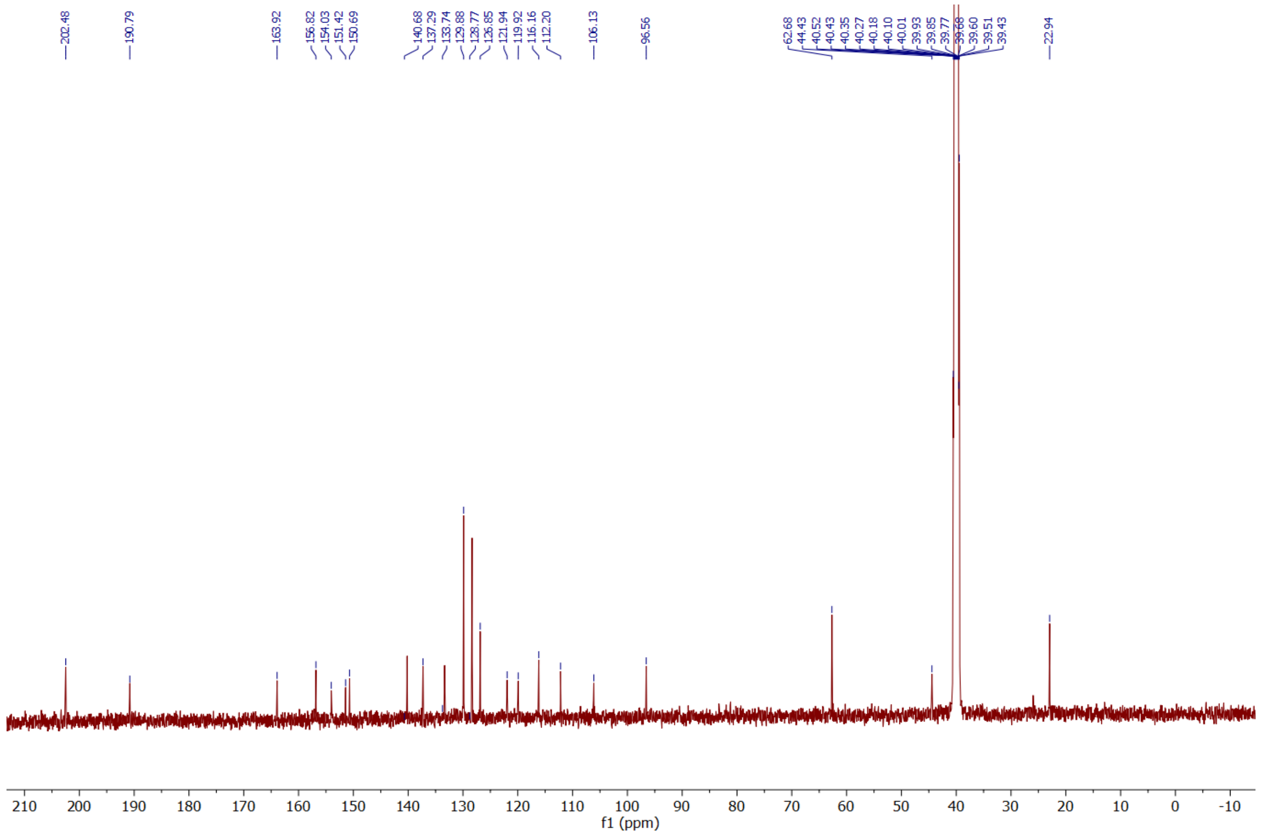

Supplementary Fig. 50. ^{13}C **NMR (126 MHz, DMSO-d_6) spectrum for 3r.**

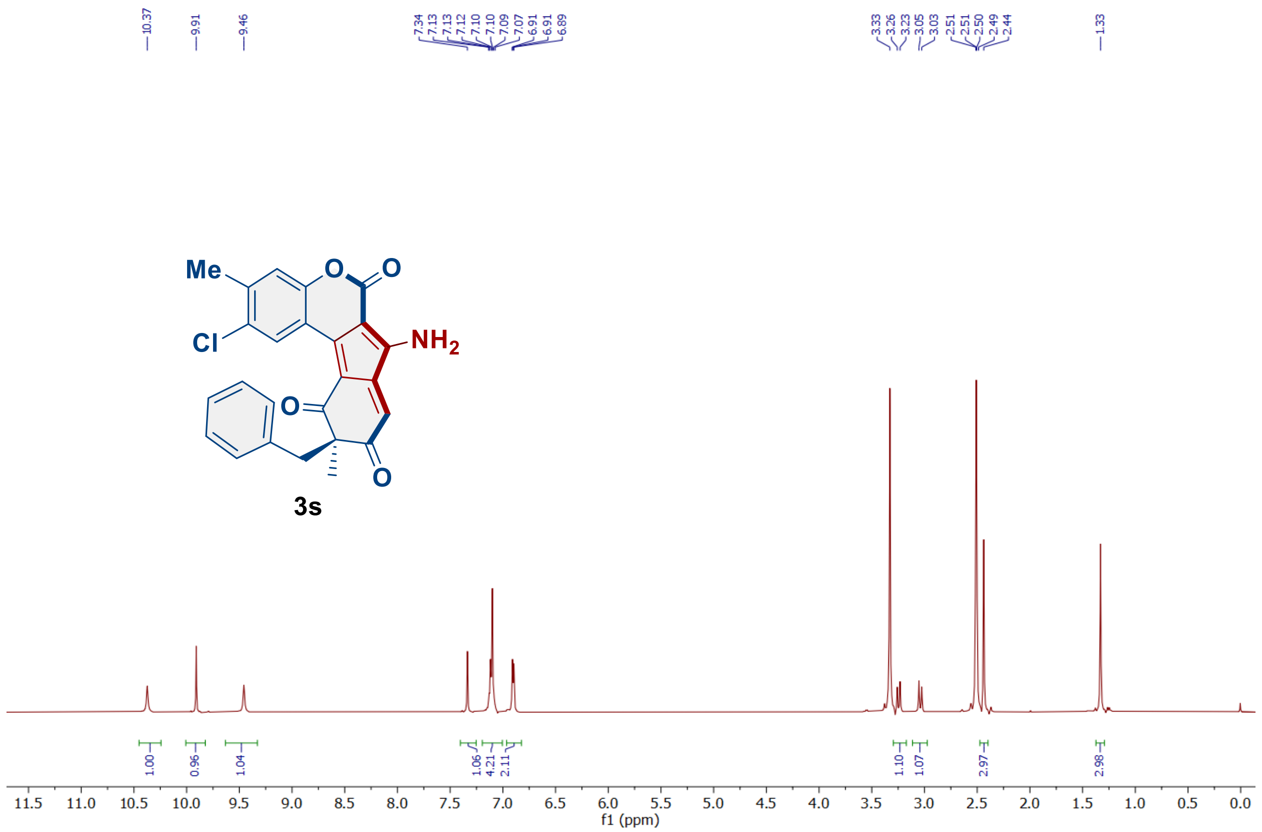

Supplementary Fig. 51. ^{1}H NMR (500 MHz, DMSO-d_6) spectrum for 3s.

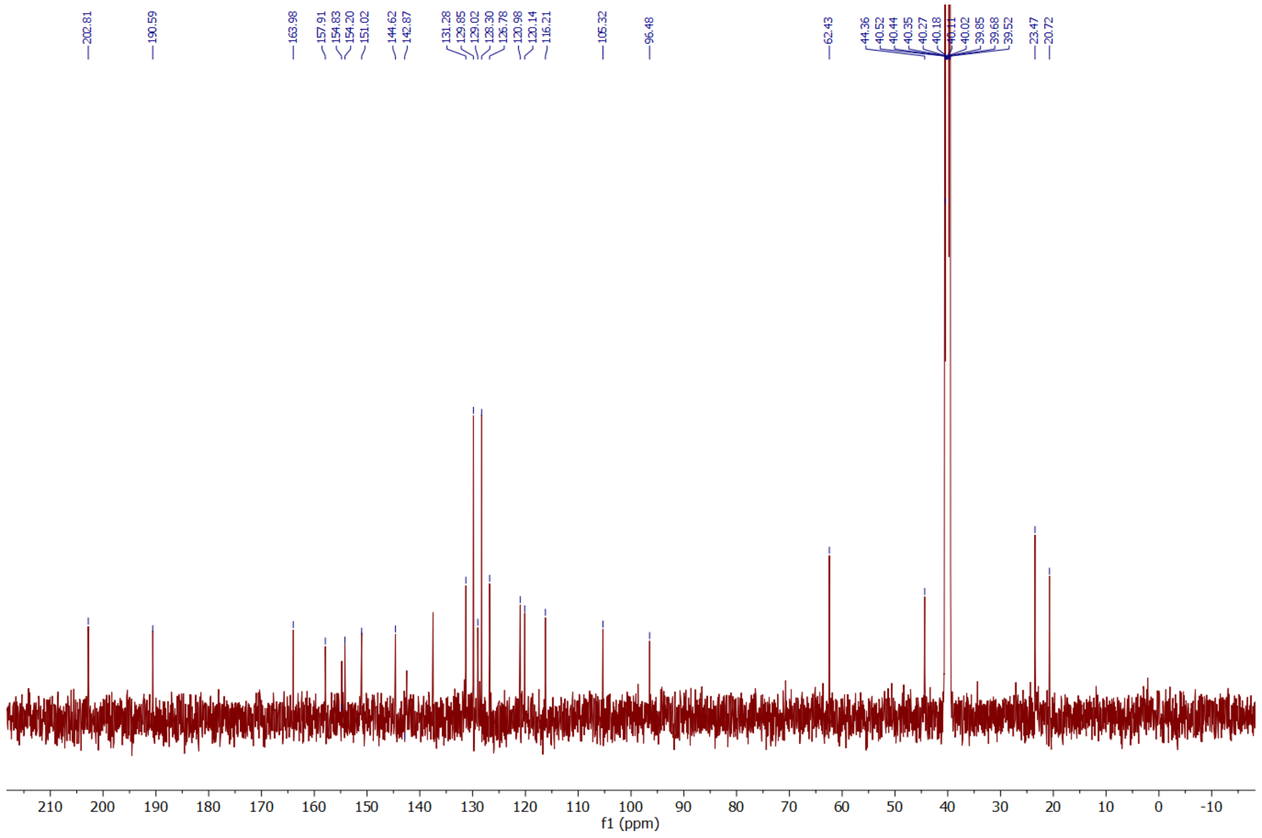

Supplementary Fig. 52. ^{13}C NMR (126 MHz, DMSO-d_6) spectrum for 3s.

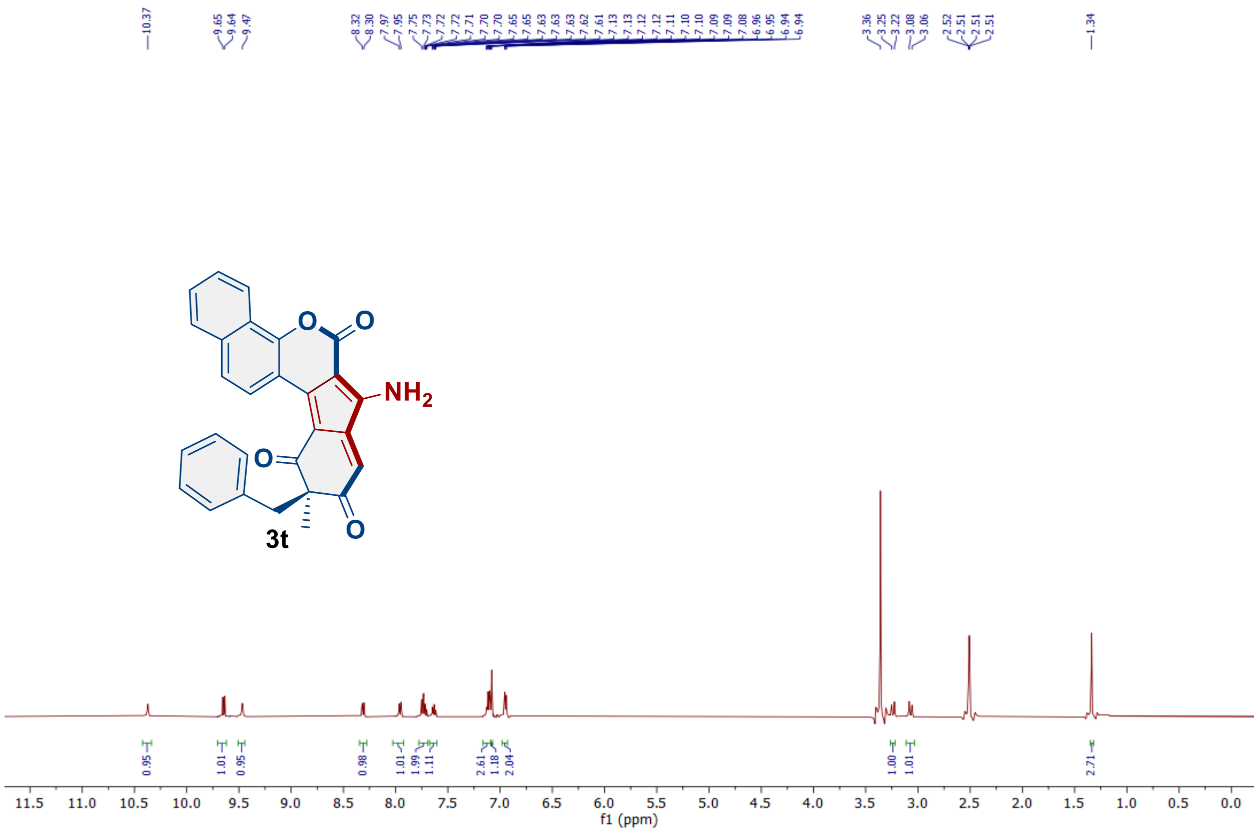

Supplementary Fig. 53. ^{1}**H NMR (500 MHz, DMSO-d_6) spectrum for 3t.**

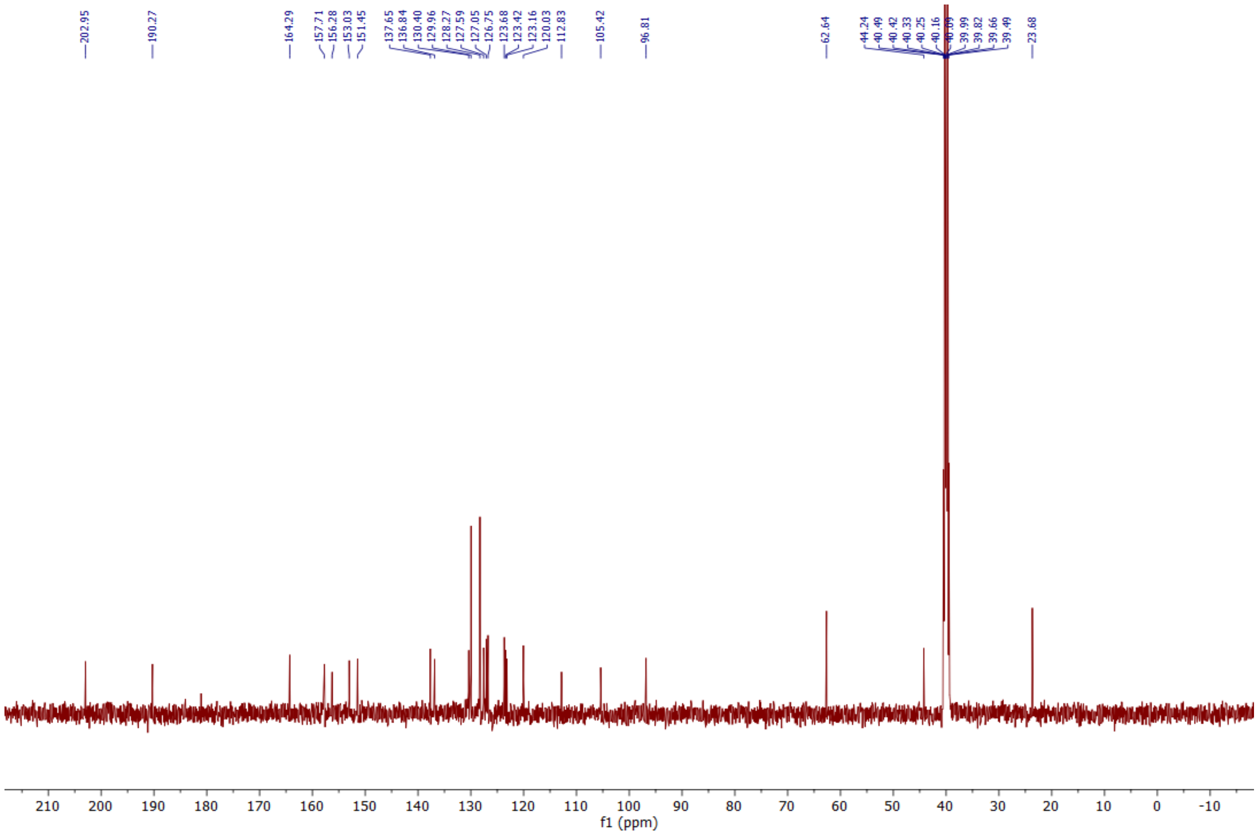

Supplementary Fig. 54. ^{13}**C NMR (126 MHz, DMSO-d_6) spectrum for 3t.**

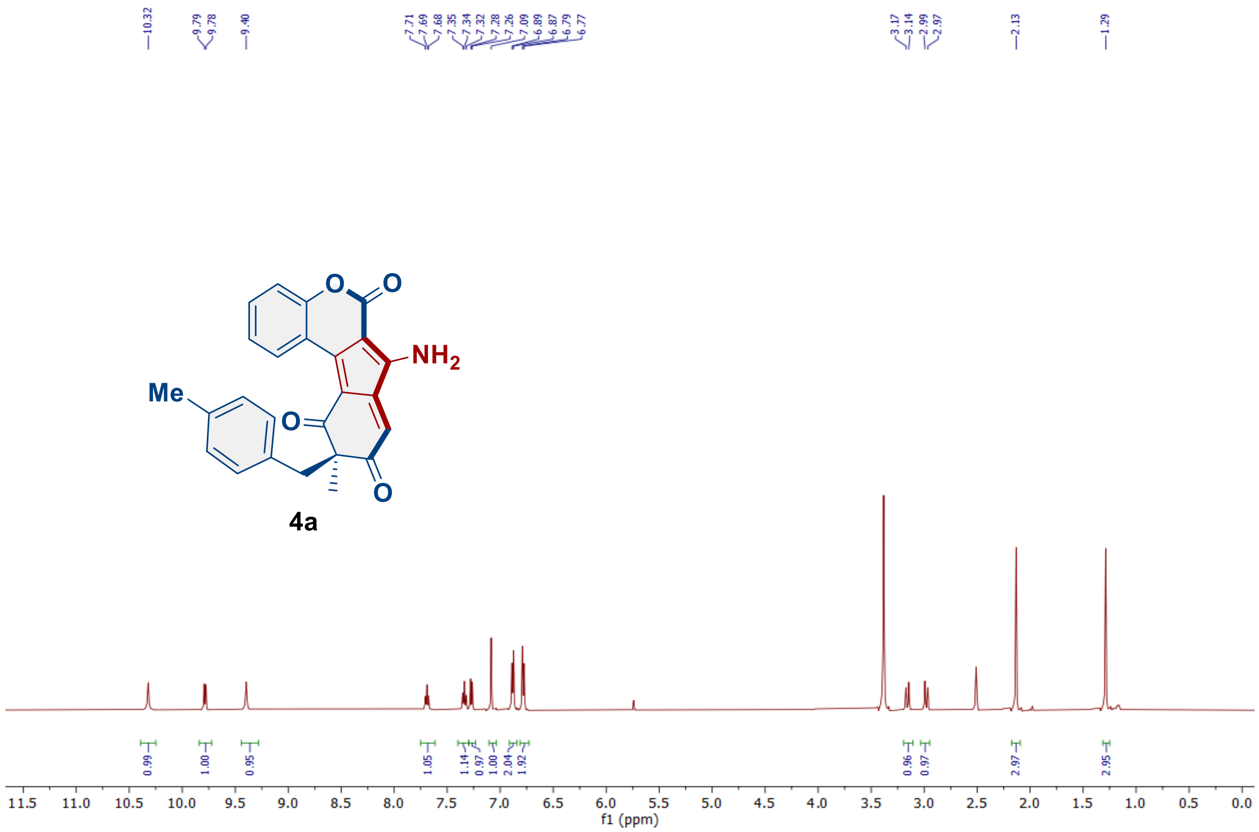

Supplementary Fig. 55. ^{1}H NMR (500 MHz, DMSO-d_6) spectrum for 4a.

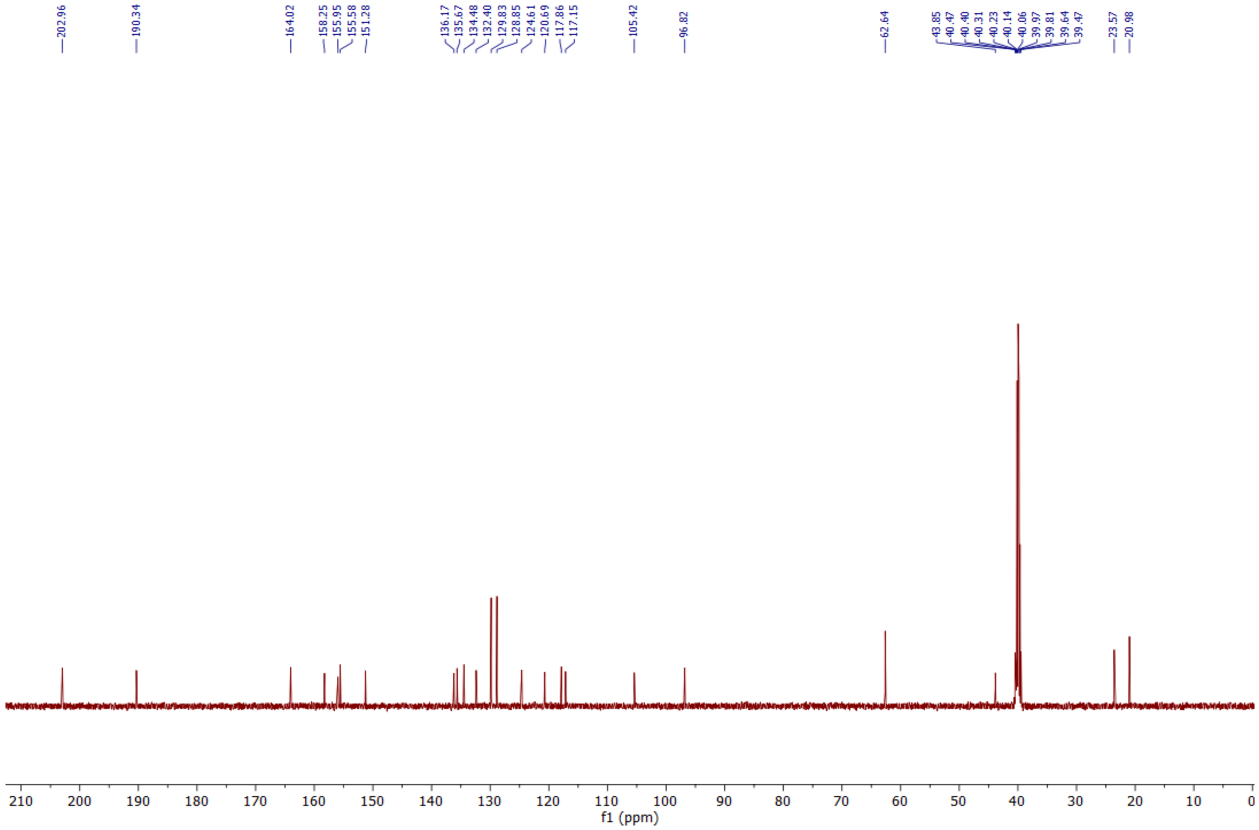

Supplementary Fig. 56. ^{13}C NMR (126 MHz, DMSO-d_6) spectrum for 4a.

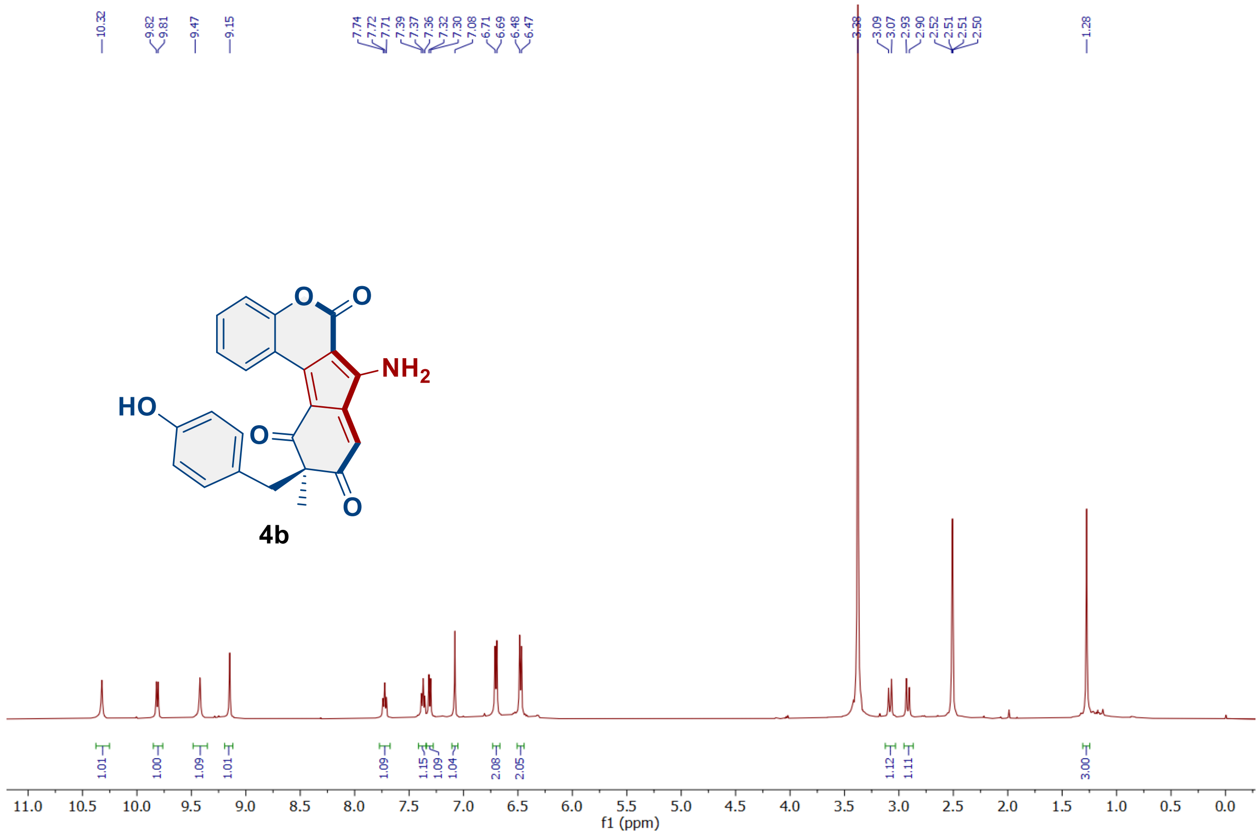

Supplementary Fig. 57. ^{1}**H NMR (500 MHz, DMSO-*d*$_6$) spectrum for 4b.**

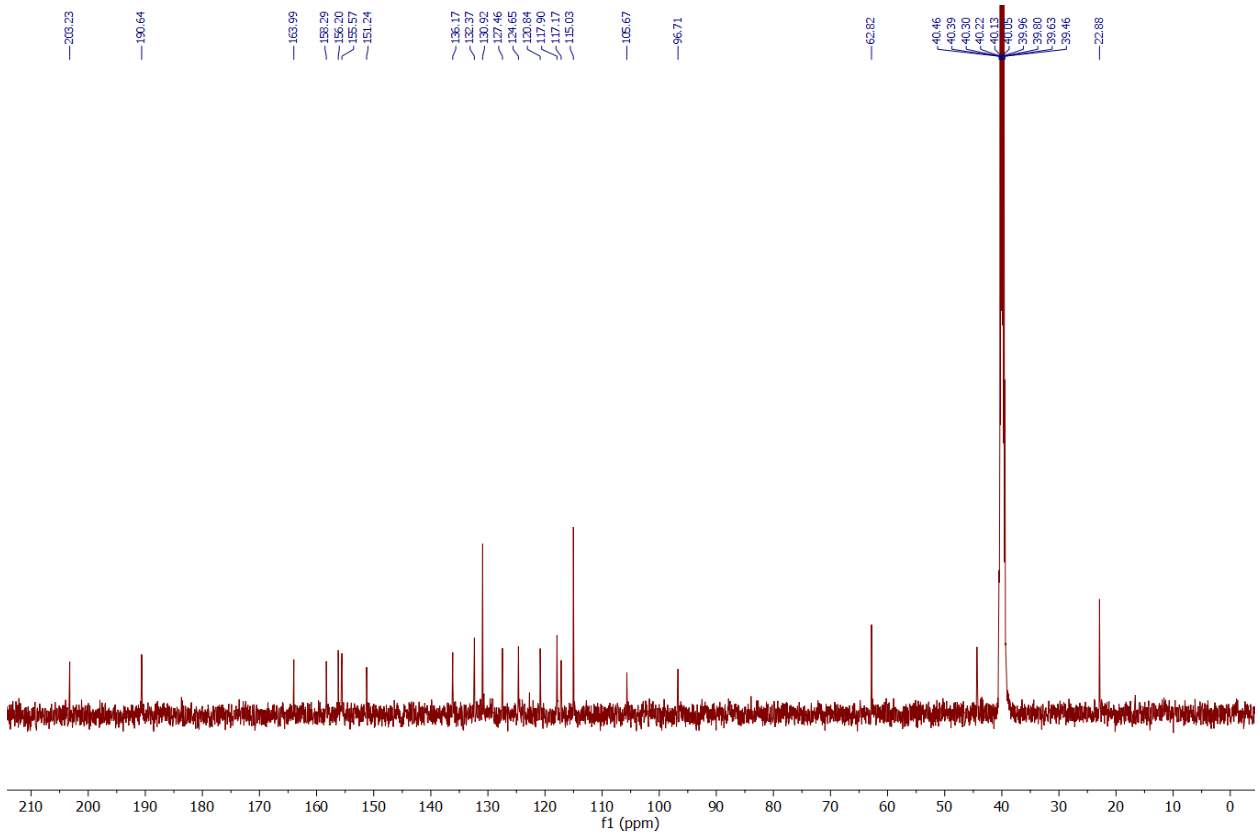

Supplementary Fig. 58. ^{13}**C NMR (126 MHz, DMSO-*d*$_6$) spectrum for 4b.**

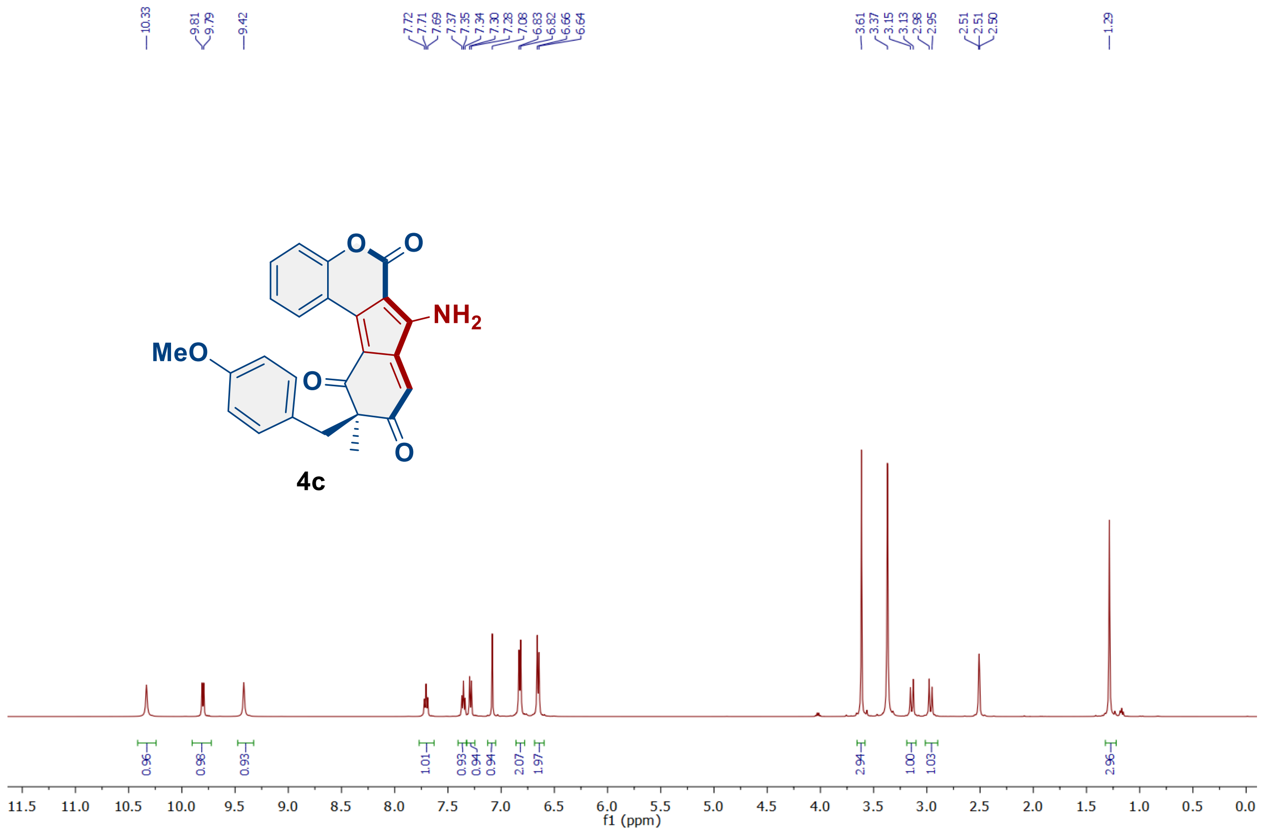

Supplementary Fig. 59. **^{1}H NMR (500 MHz, DMSO-d_6) spectrum for 4c.**

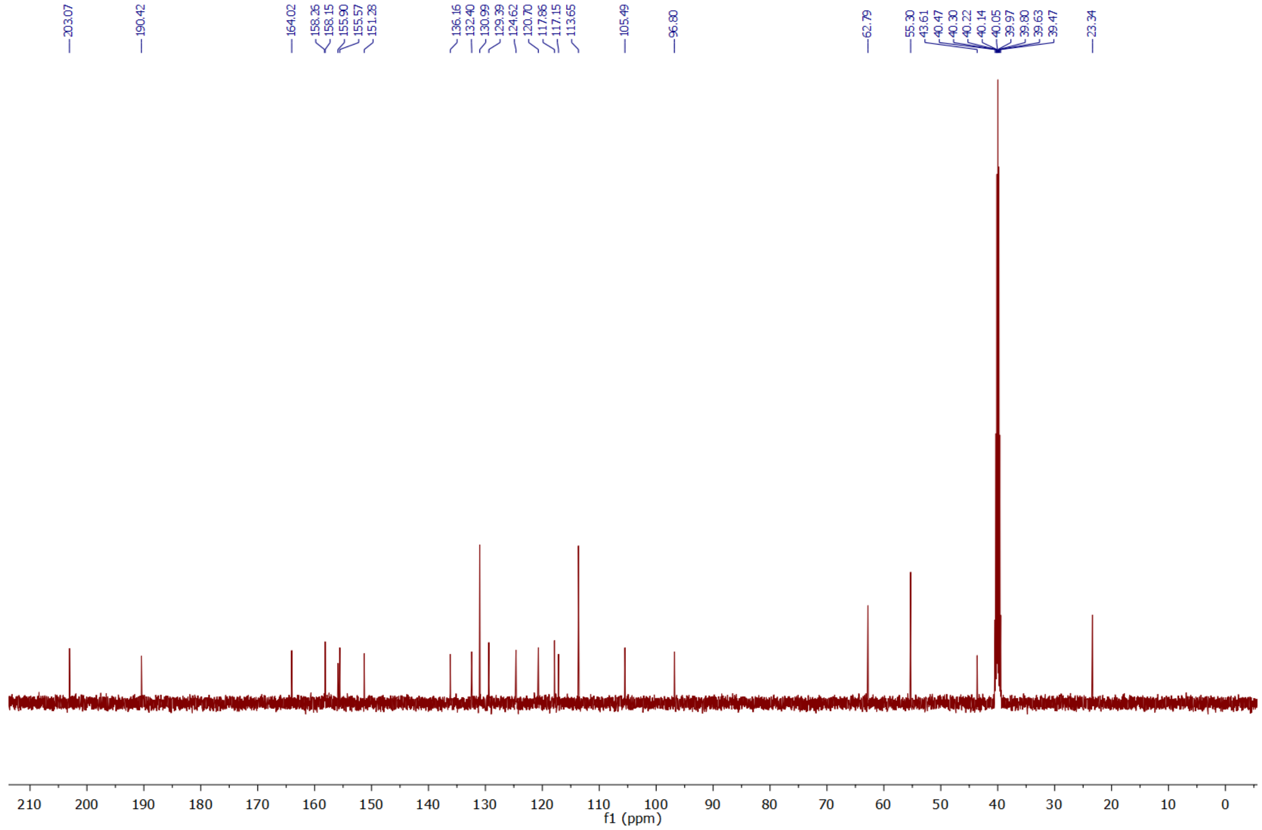

Supplementary Fig. 60. **^{13}C NMR (126 MHz, DMSO-d_6) spectrum for 4c.**

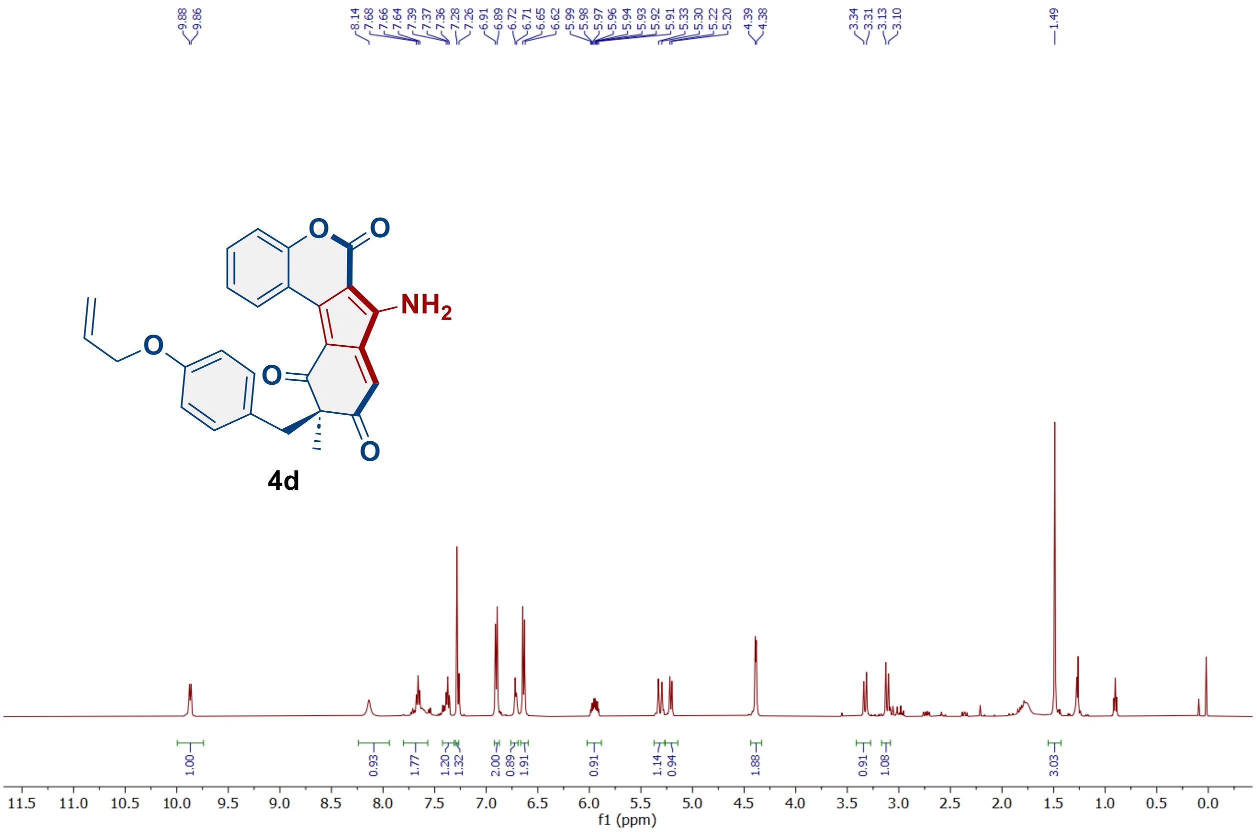

Supplementary Fig. 61. ^{1}H NMR (500 MHz, DMSO-d_6) spectrum for 4d.

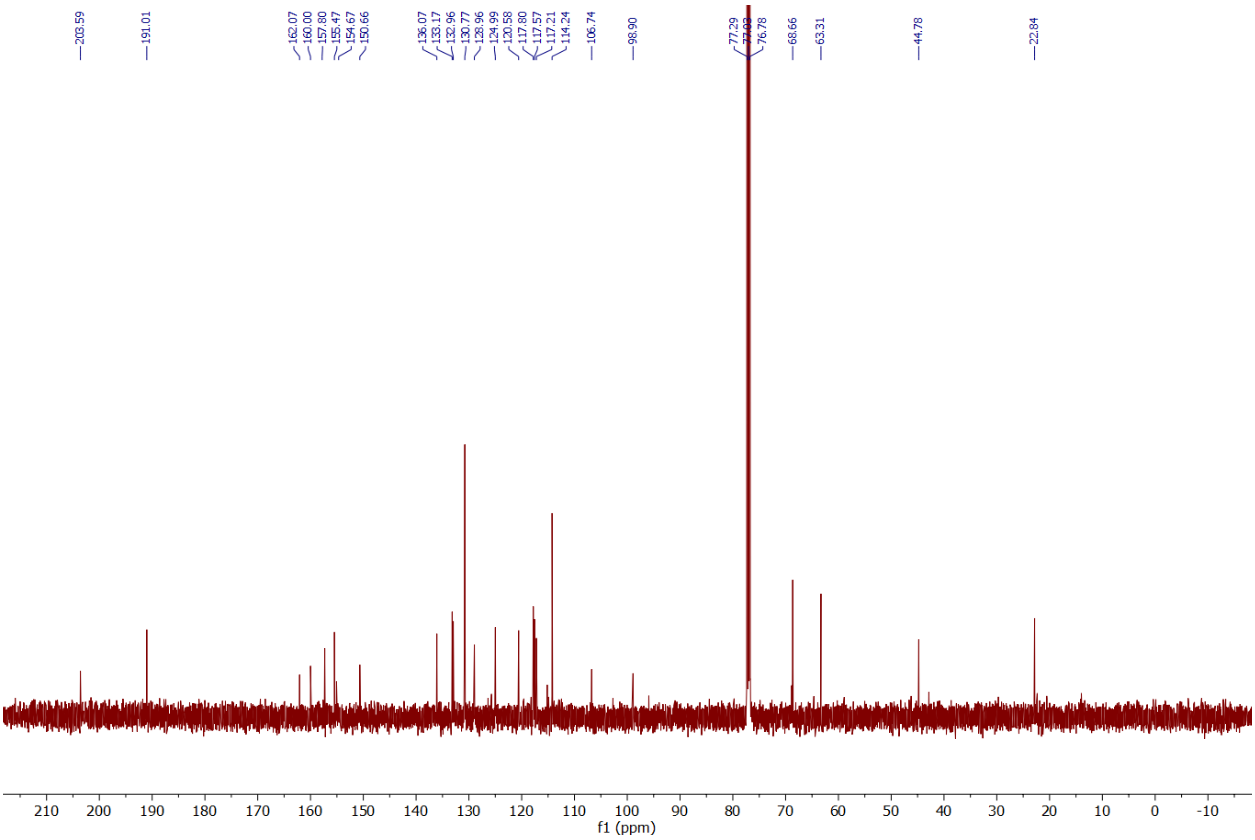

Supplementary Fig. 62. ^{13}C NMR (126 MHz, DMSO-d_6) spectrum for 4d.

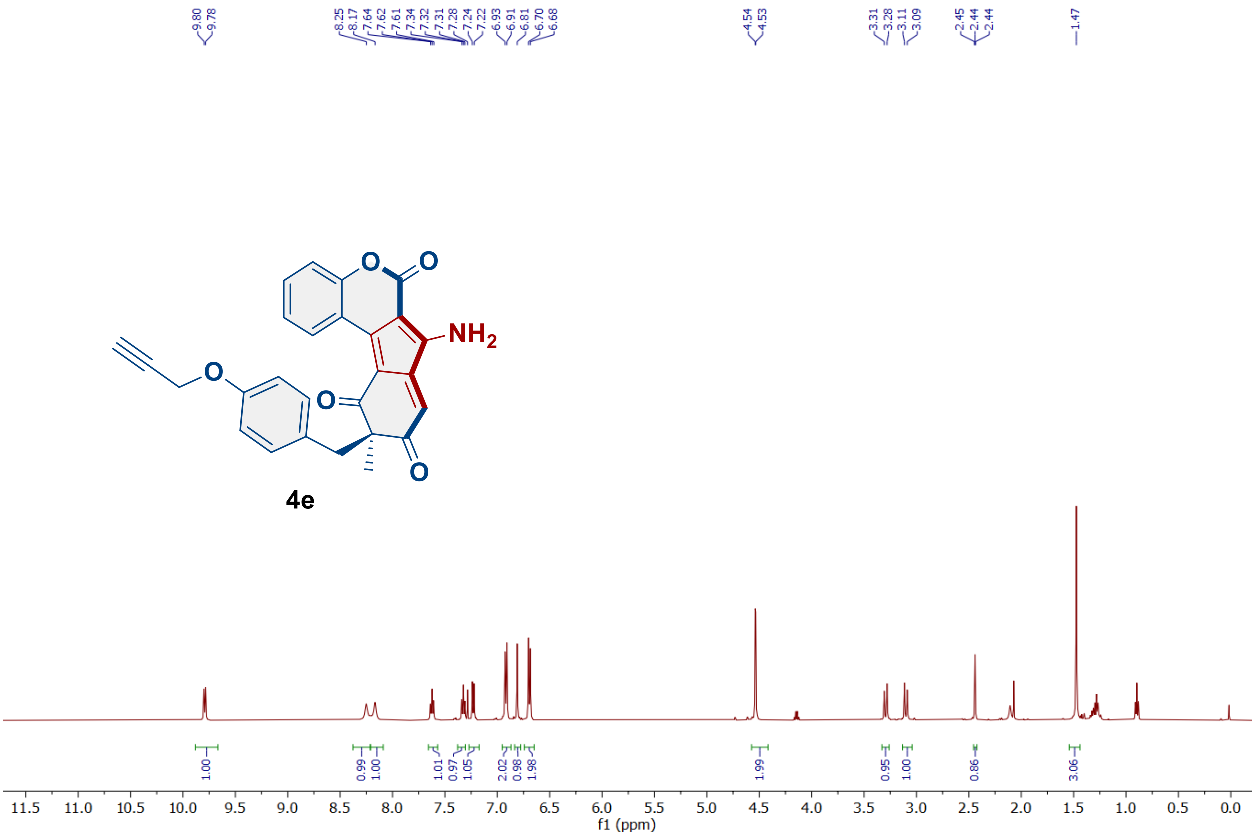

Supplementary Fig. 63. ^{1}H NMR (500 MHz, DMSO-d_6) spectrum for 4e.

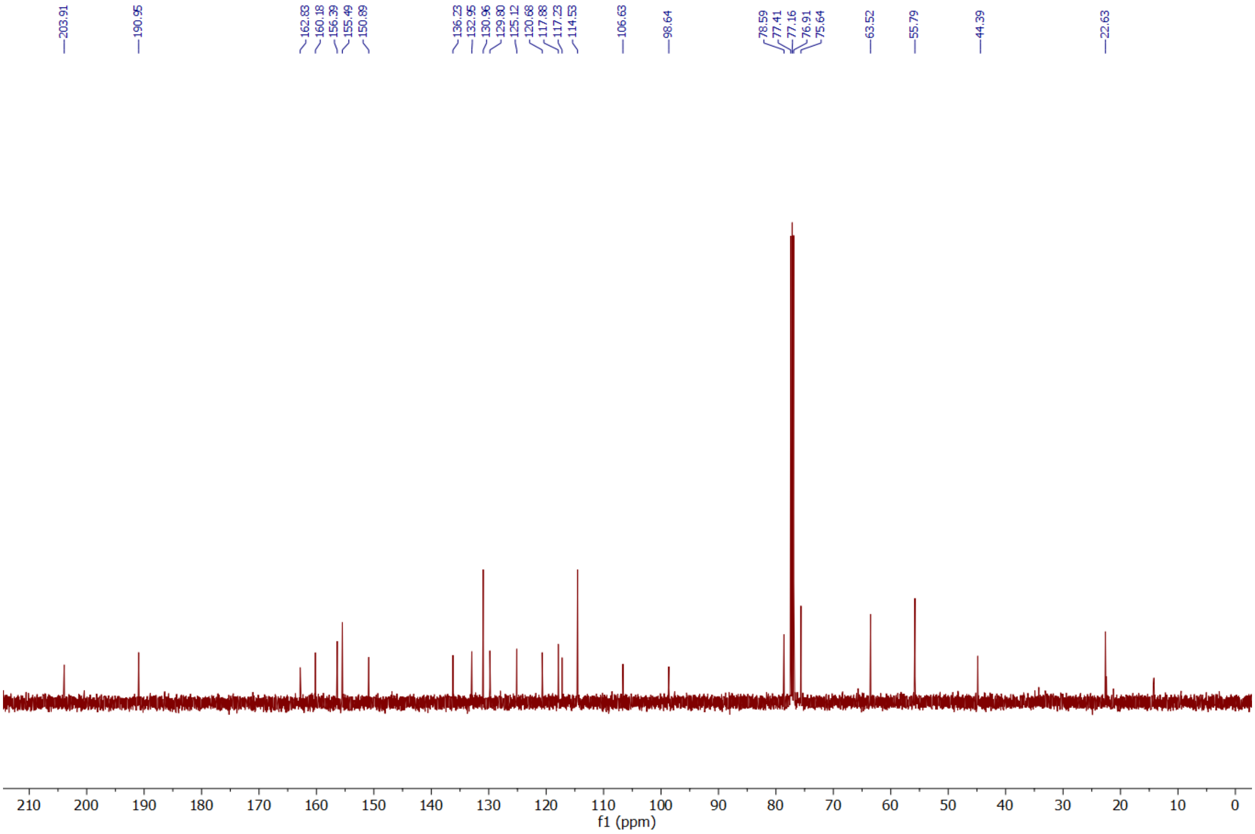

Supplementary Fig. 64. ^{13}C NMR (126 MHz, DMSO-d_6) spectrum for 4e.

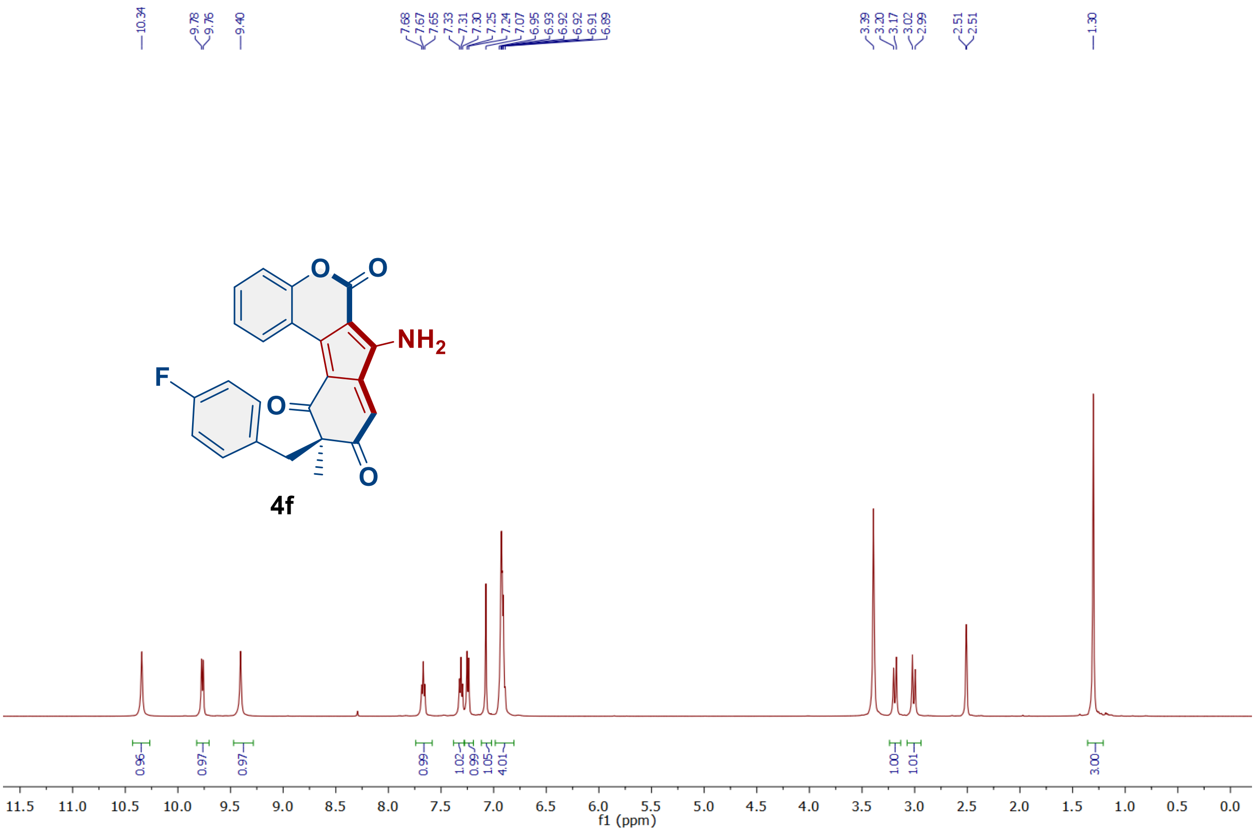

Supplementary Fig. 65. ^{1}H NMR (500 MHz, DMSO-d_6) spectrum for 4f.

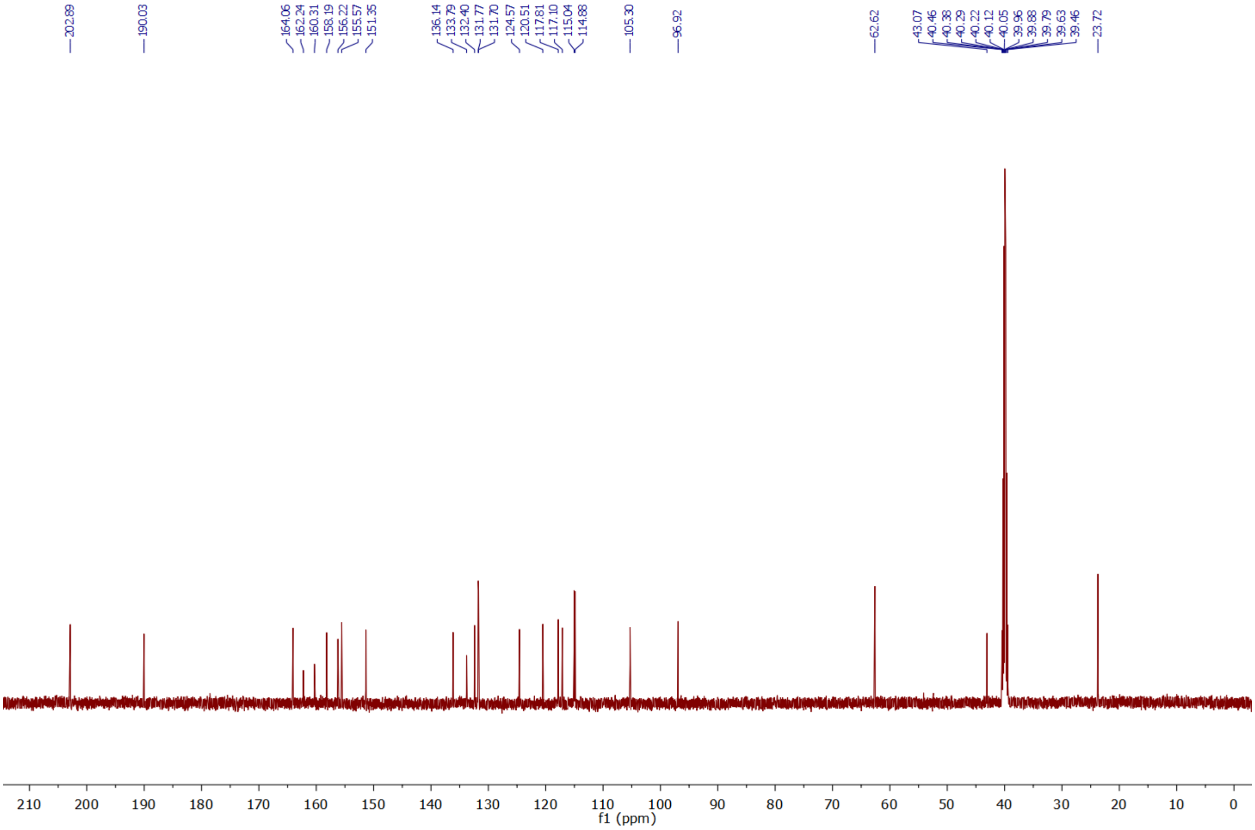

Supplementary Fig. 66. ^{13}C NMR (126 MHz, DMSO-d_6) spectrum for 4f.

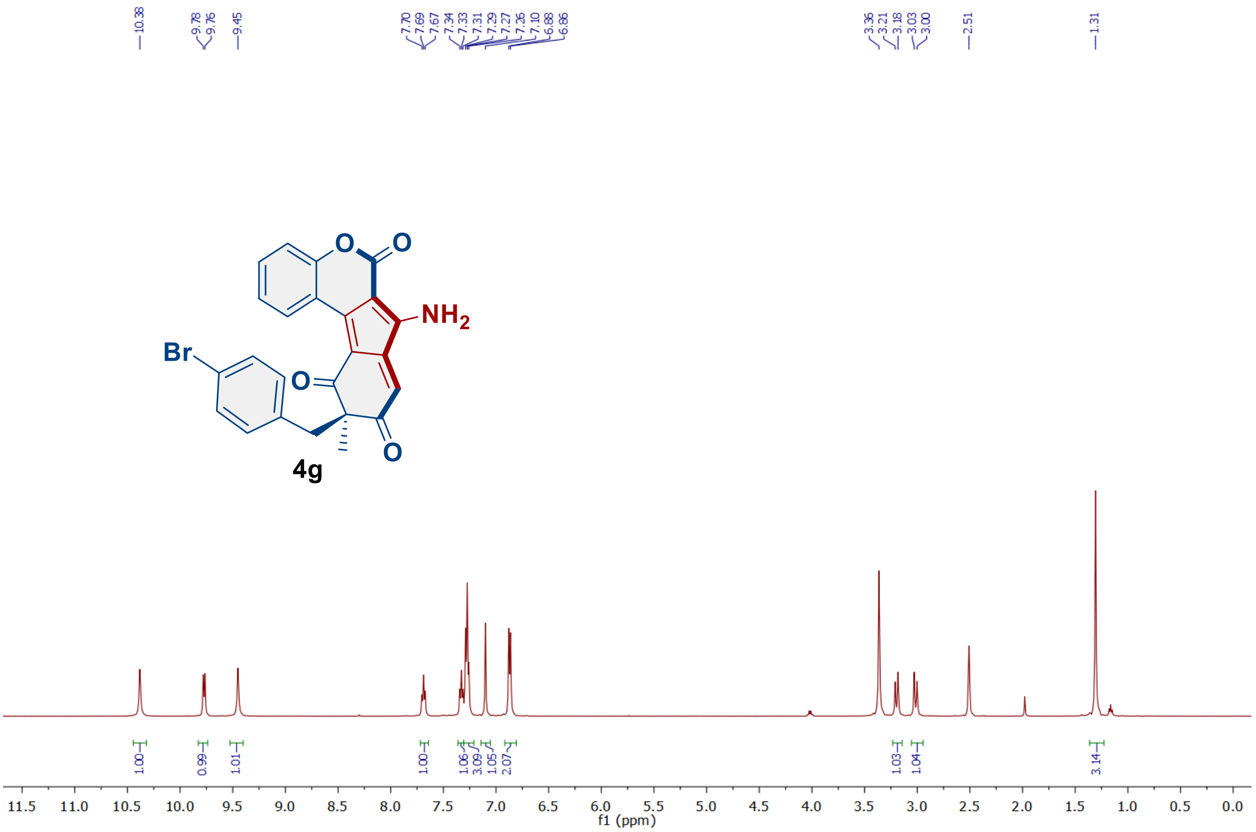

Supplementary Fig. 67. ^{1}H NMR (500 MHz, DMSO-d_6) spectrum for 4g.

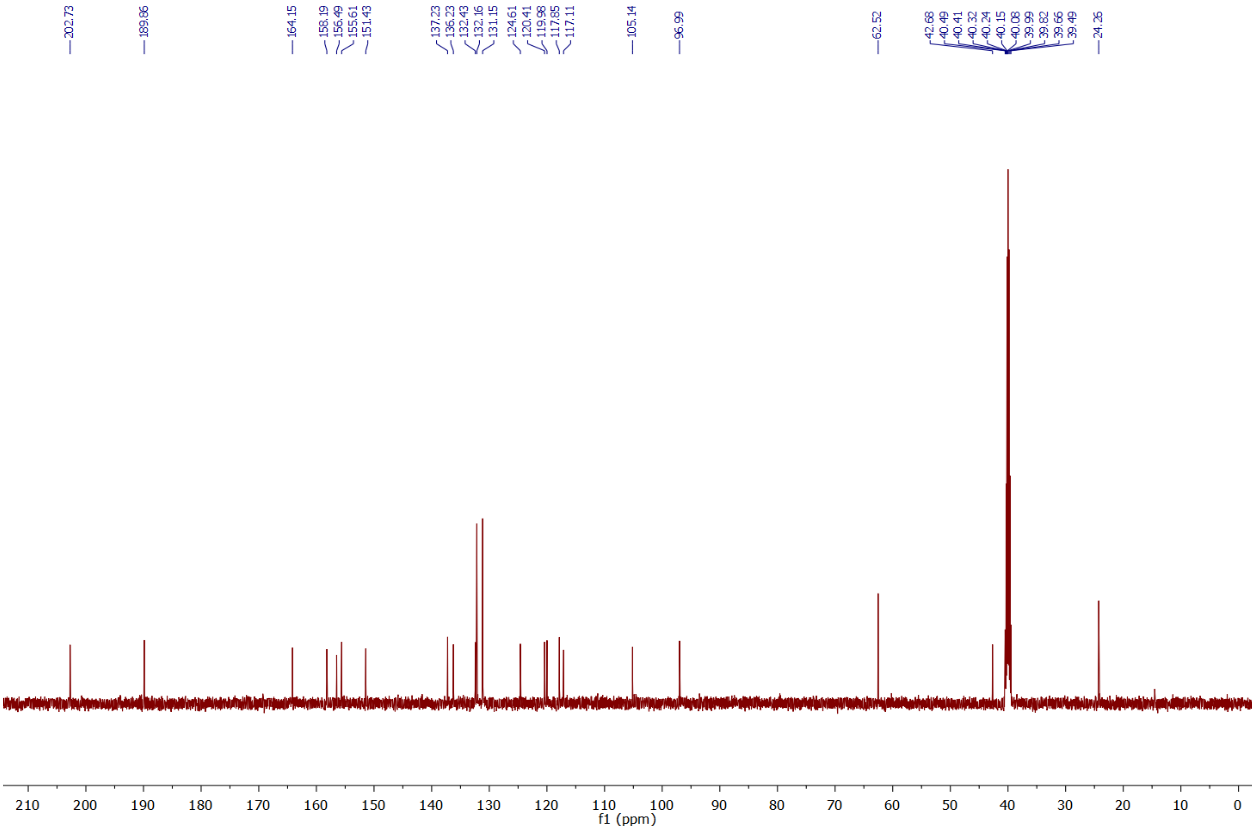

Supplementary Fig. 68. ^{13}C NMR (126 MHz, DMSO-d_6) spectrum for 4g.

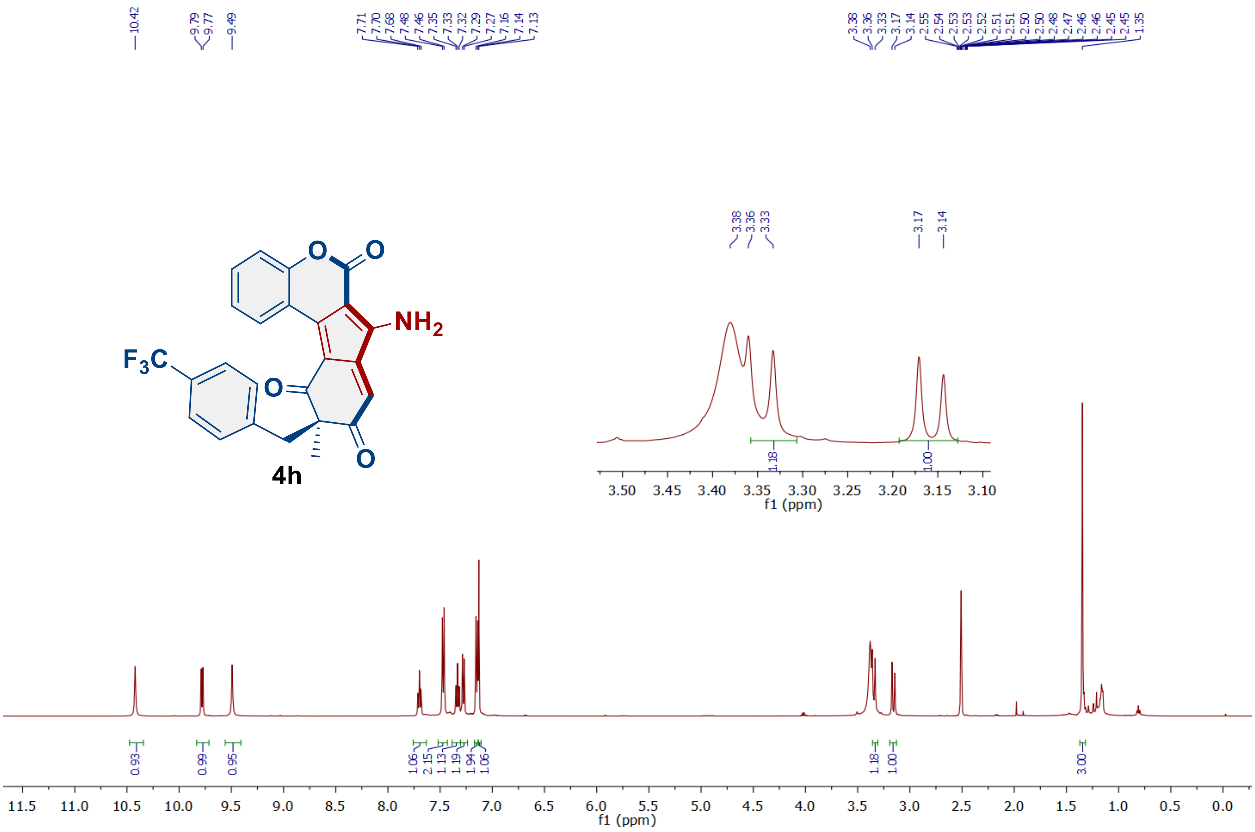

Supplementary Fig. 69. ^{1}**H NMR (500 MHz, DMSO-*d*$_6$) spectrum for 4h.**

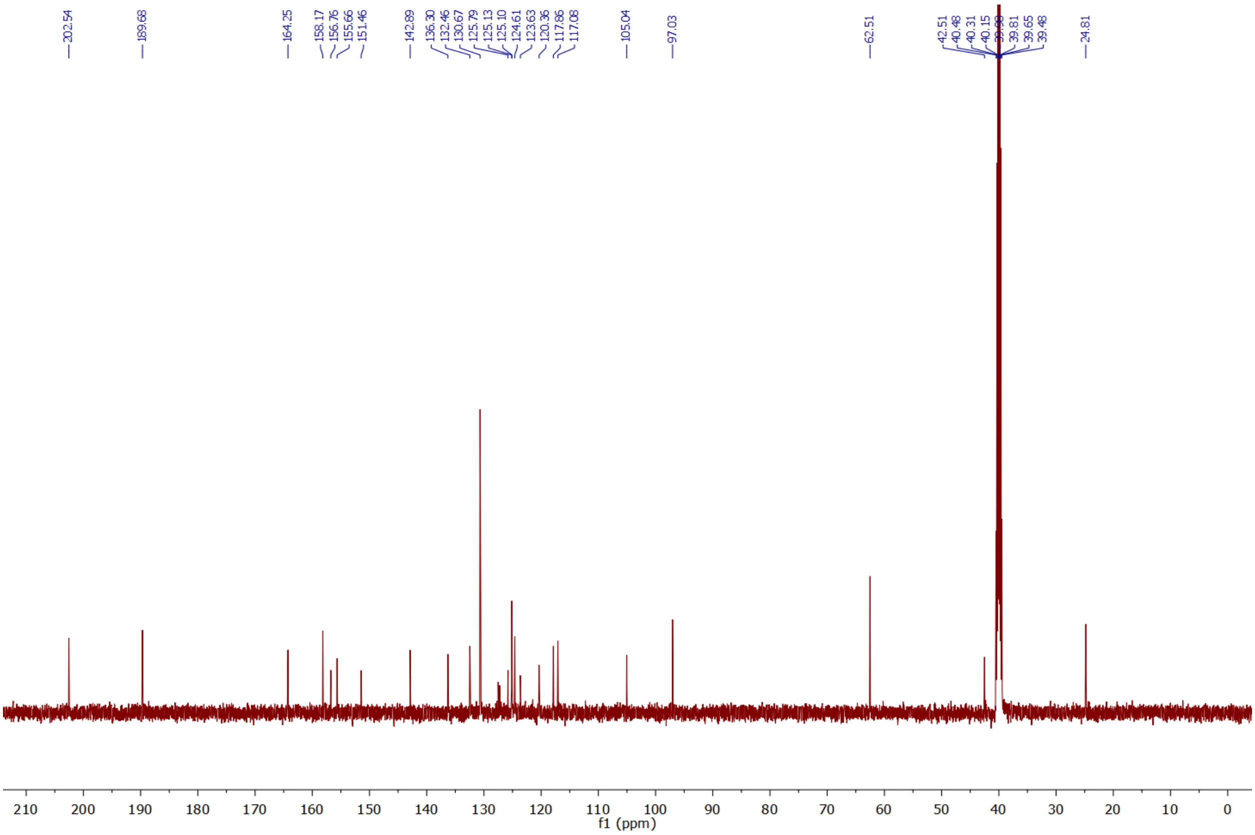

Supplementary Fig. 70. ^{13}**C NMR (126 MHz, DMSO-*d*$_6$) spectrum for 4h.**

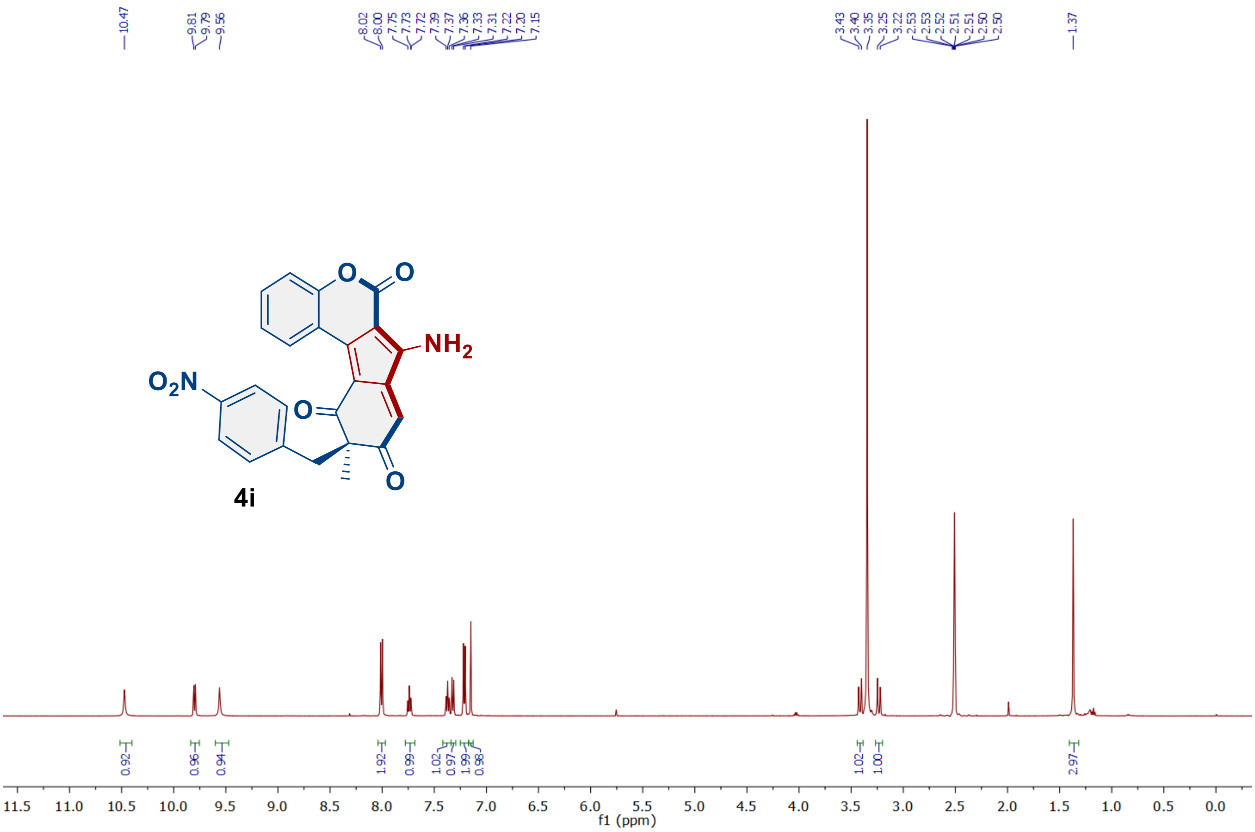

Supplementary Fig. 71. ^{1}**H NMR (500 MHz, DMSO-*d*$_{6}$) spectrum for 4i.**

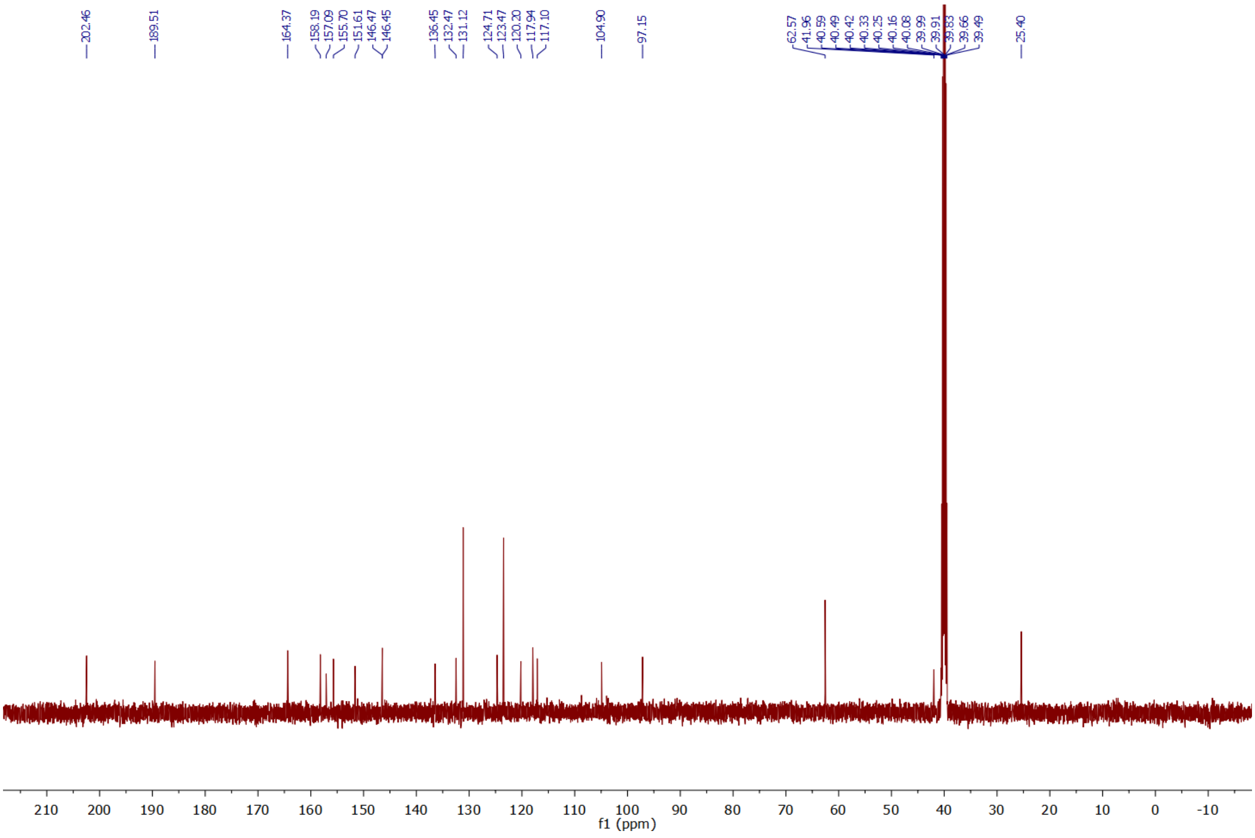

Supplementary Fig. 72. ^{13}**C NMR (126 MHz, DMSO-*d*$_{6}$) spectrum for 4i.**

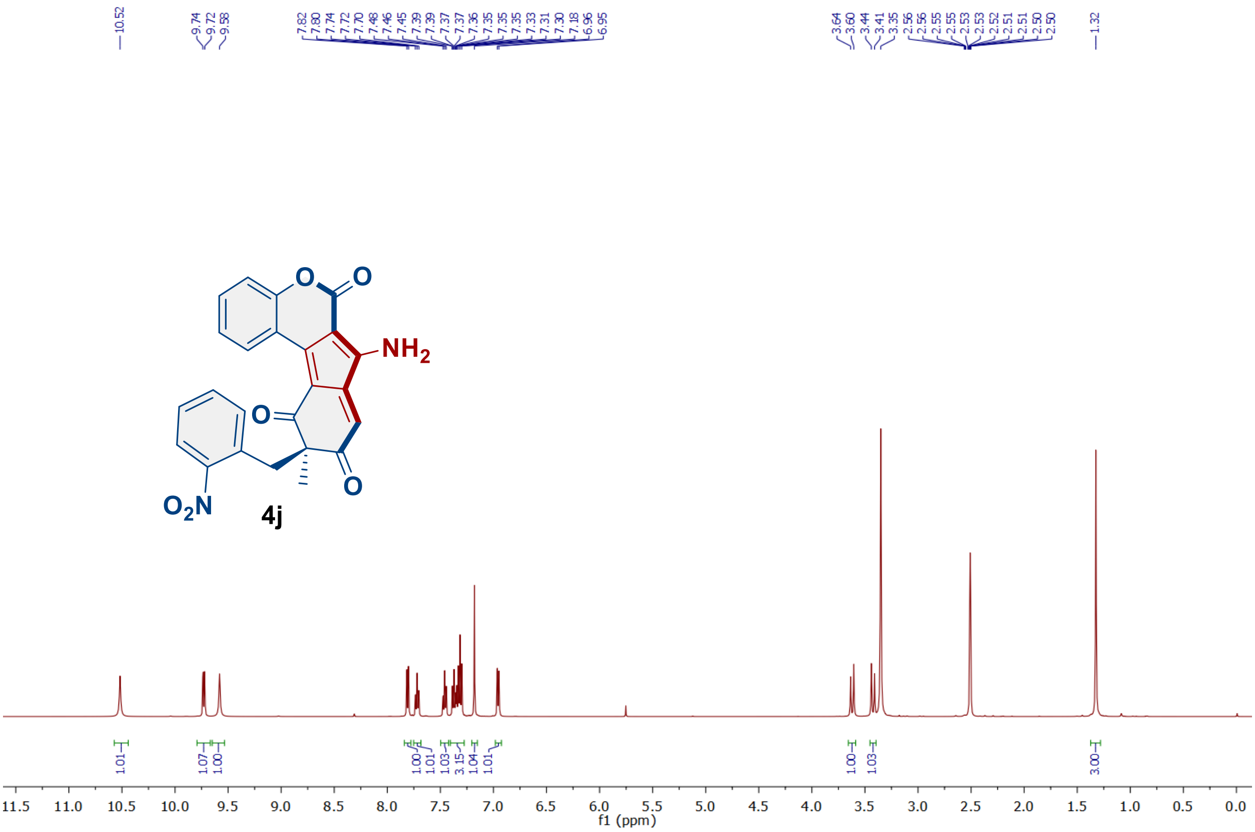

Supplementary Fig. 73. ^{1}H NMR (500 MHz, DMSO-d_6) spectrum for 4j.

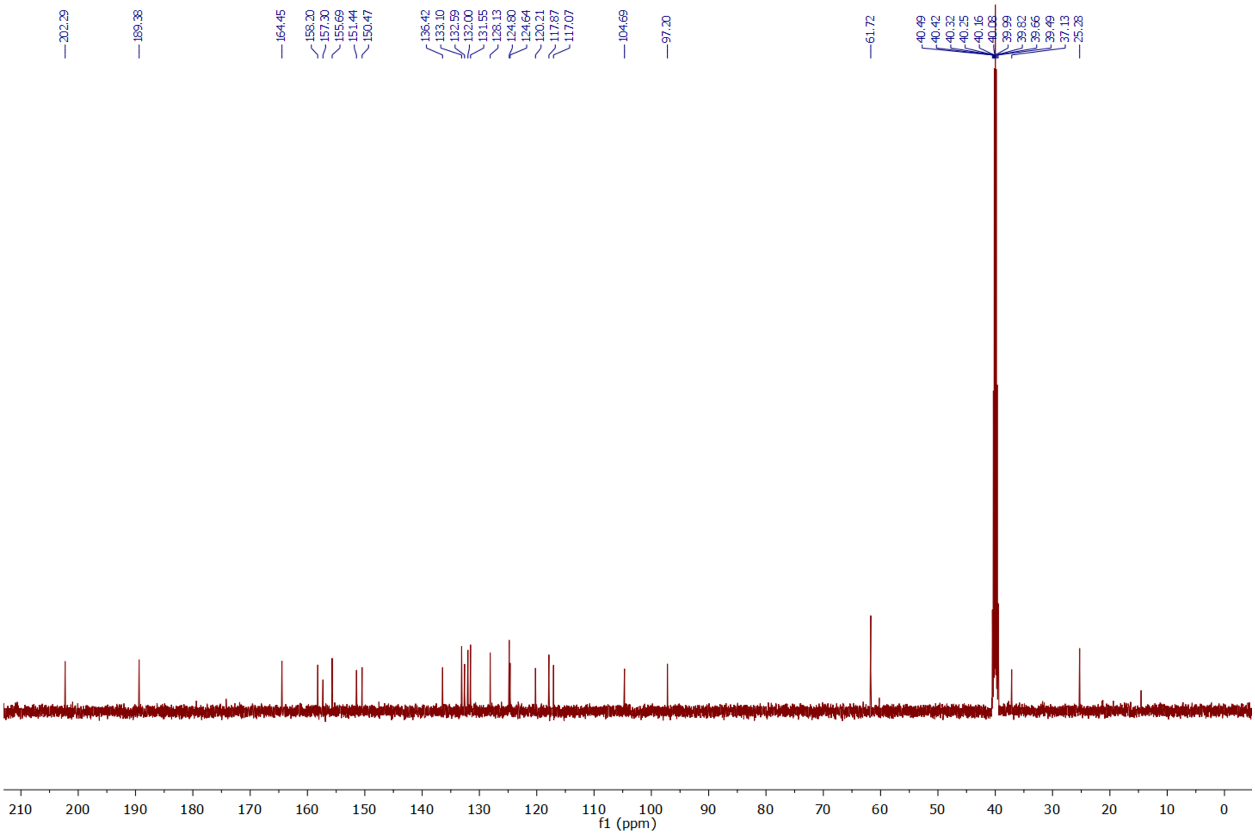

Supplementary Fig. 74. ^{13}C NMR (126 MHz, DMSO-d_6) spectrum for 4j.

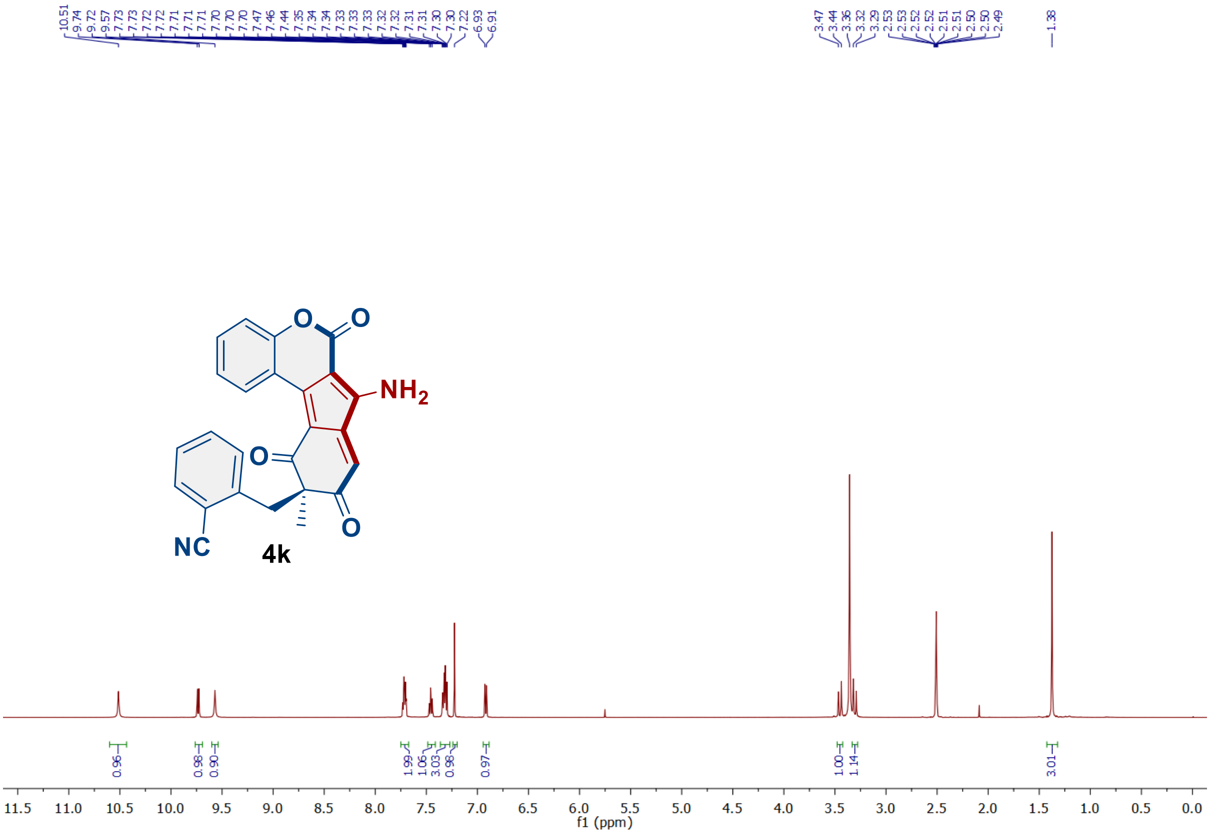

Supplementary Fig. 75. **^{1}H NMR (500 MHz, DMSO-d_6) spectrum for 4k.**

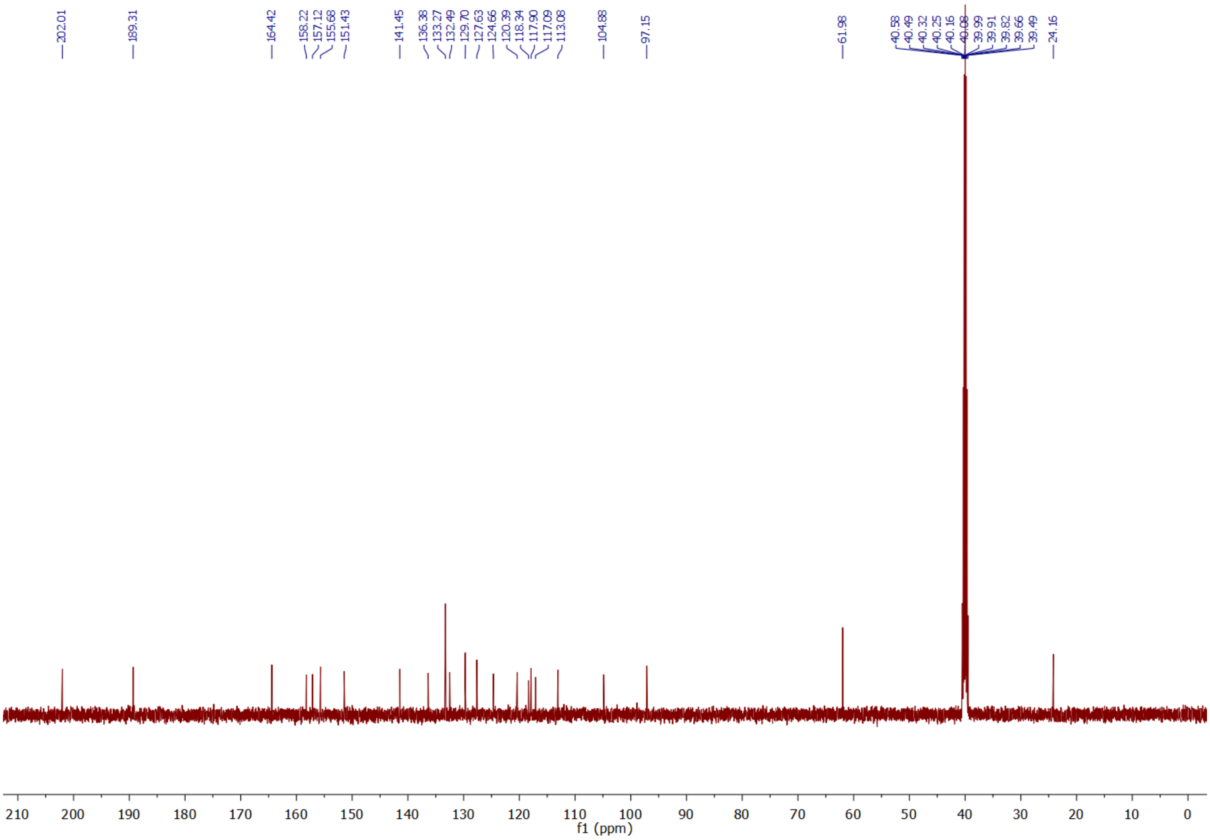

Supplementary Fig. 76. **^{13}C NMR (126 MHz, DMSO-d_6) spectrum for 4k.**

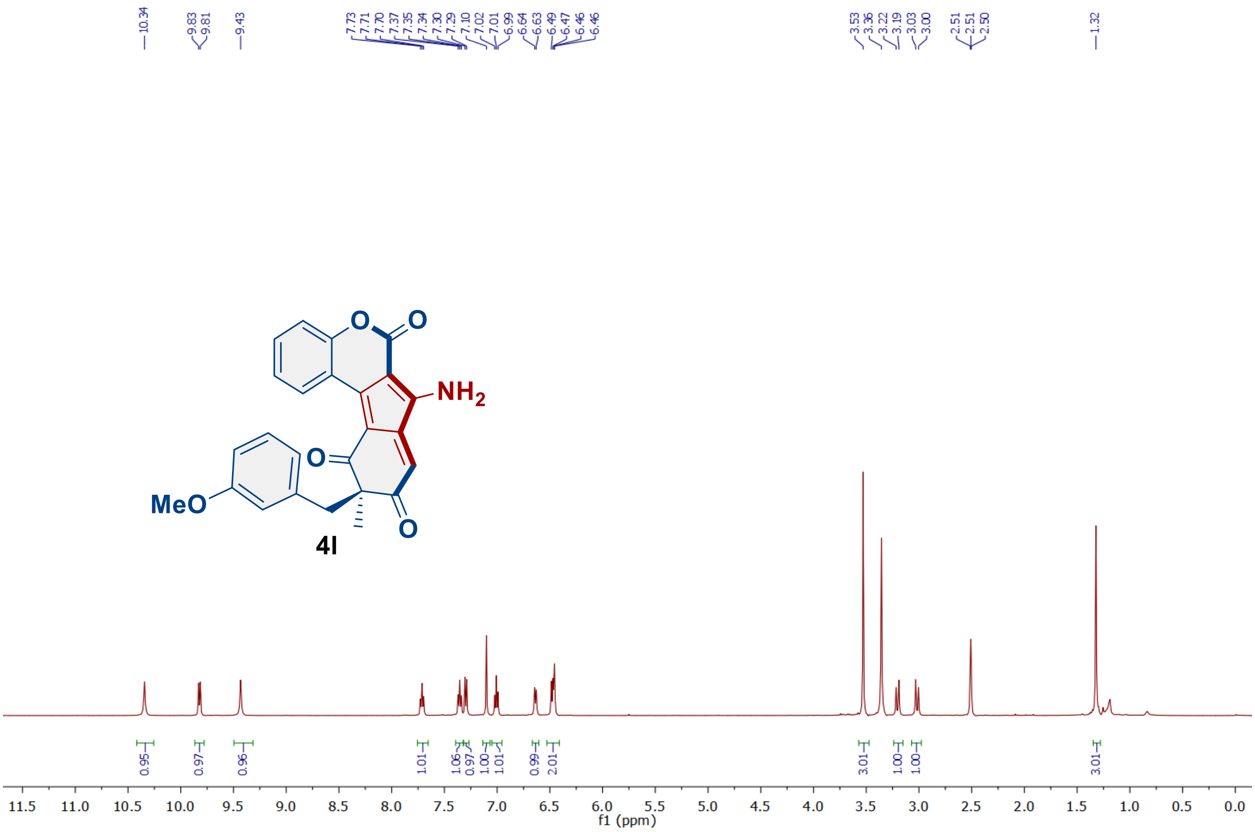

Supplementary Fig. 77. ^{1}**H NMR (500 MHz, DMSO-**d_6**) spectrum for 4l.**

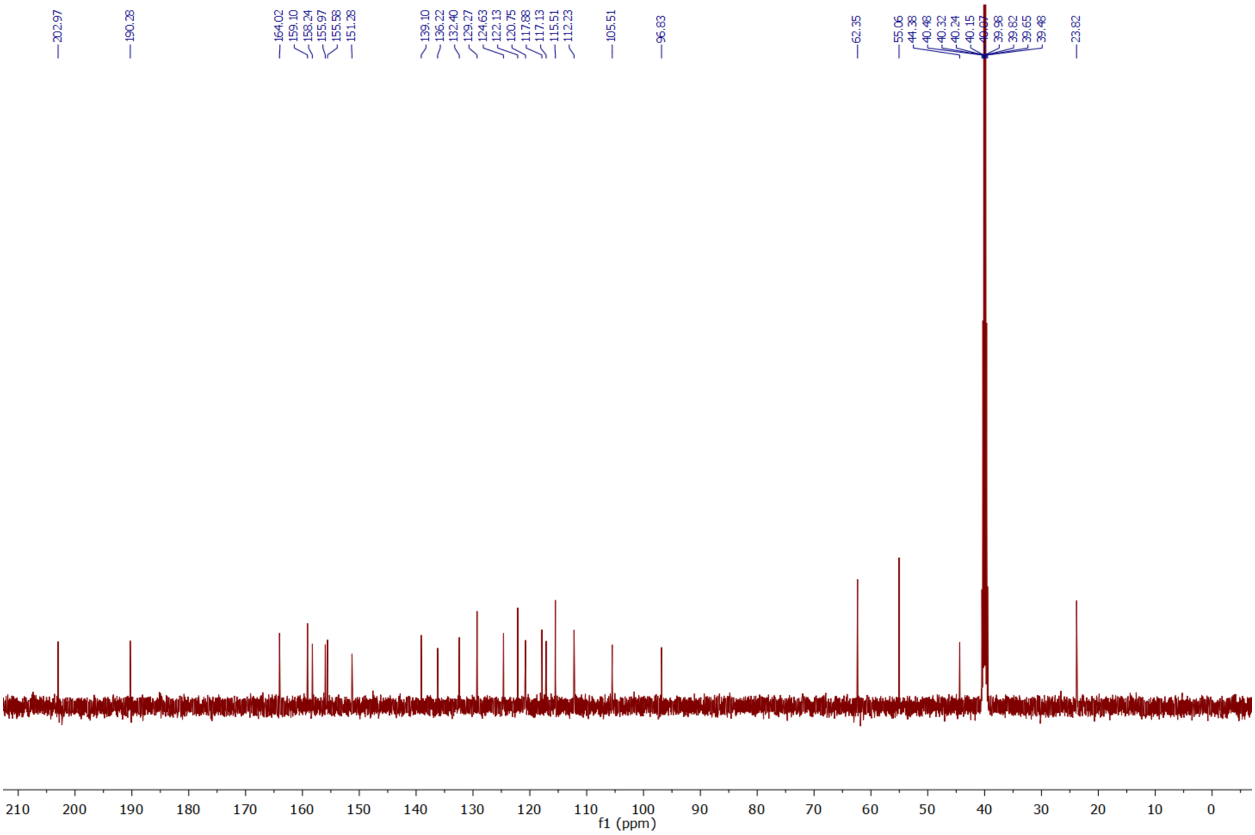

Supplementary Fig. 78. ^{13}**C NMR (126 MHz, DMSO-**d_6**) spectrum for 4l.**

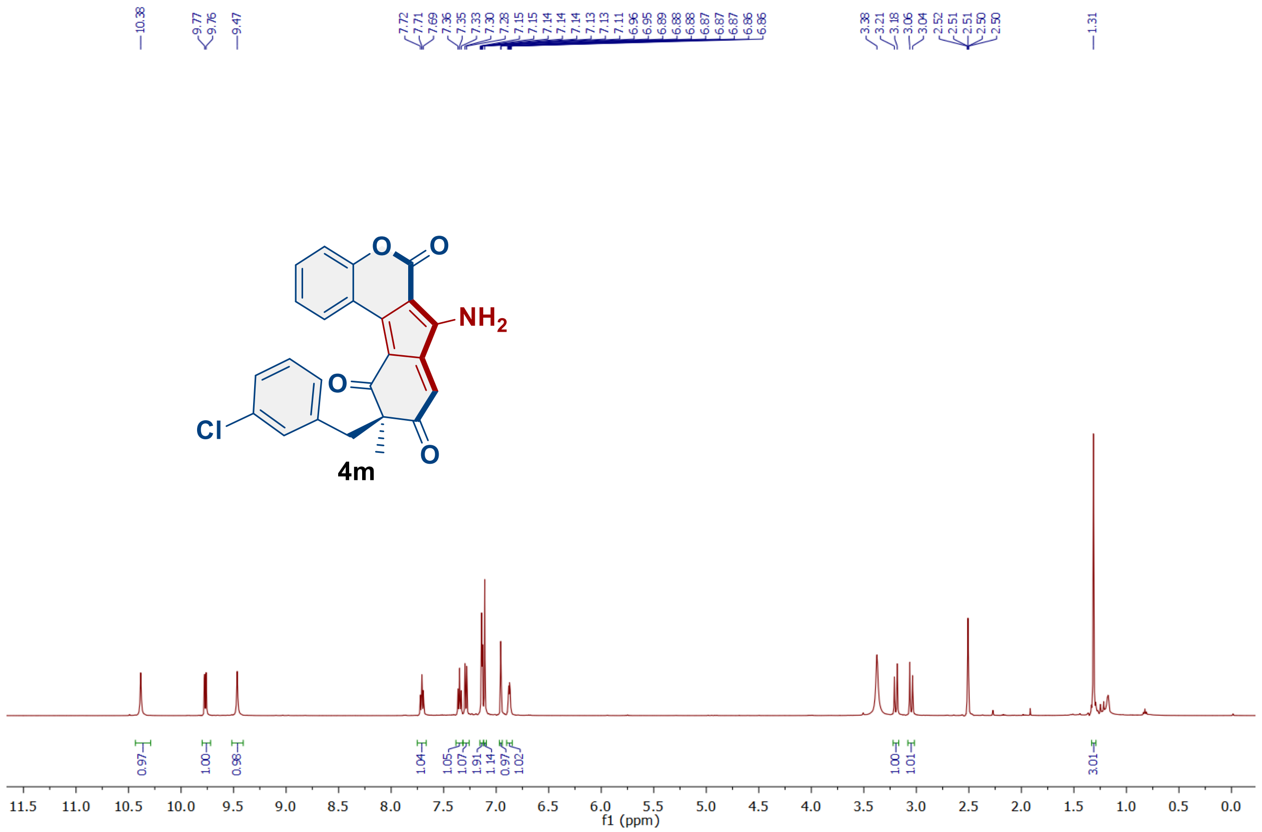

Supplementary Fig. 79. ^{1}**H NMR (500 MHz, DMSO-**d_6**) spectrum for 4m.**

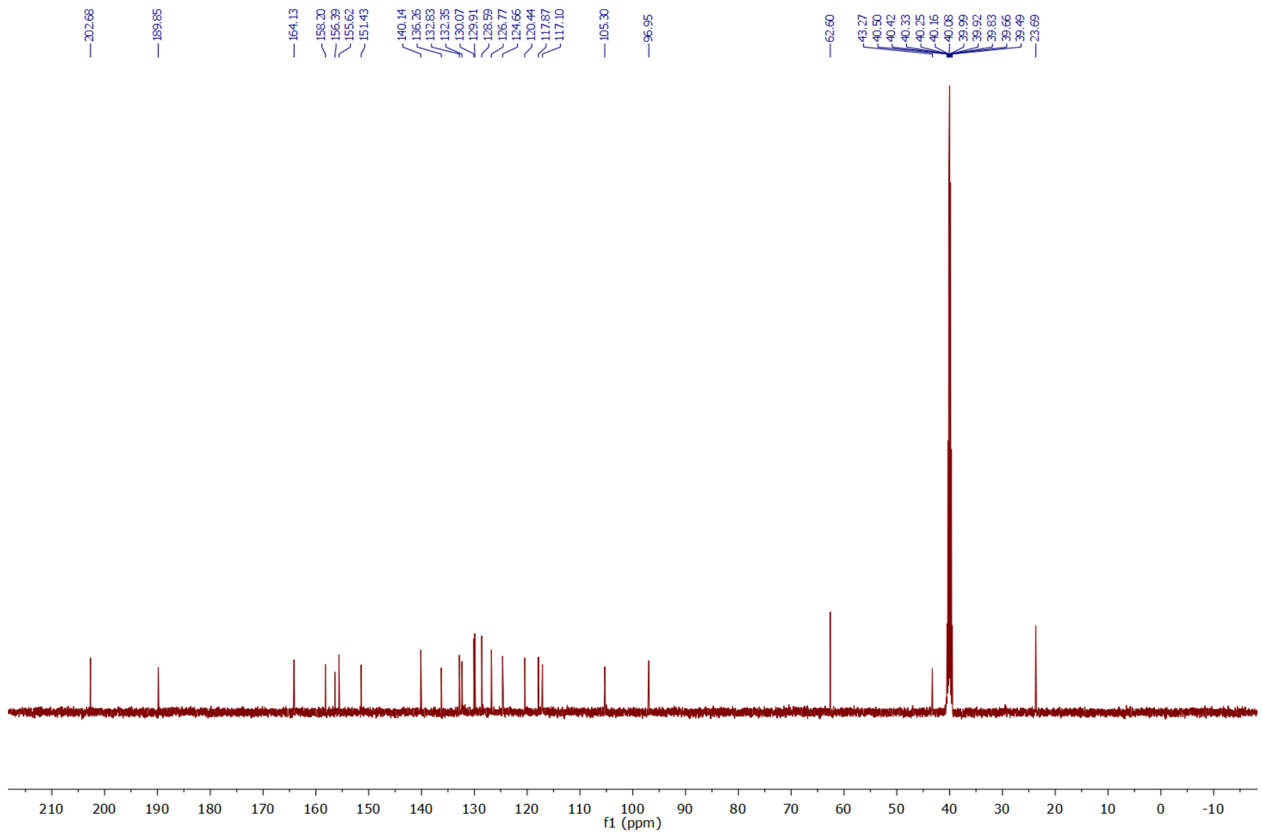

Supplementary Fig. 80. ^{13}**C NMR (126 MHz, DMSO-**d_6**) spectrum for 4m.**

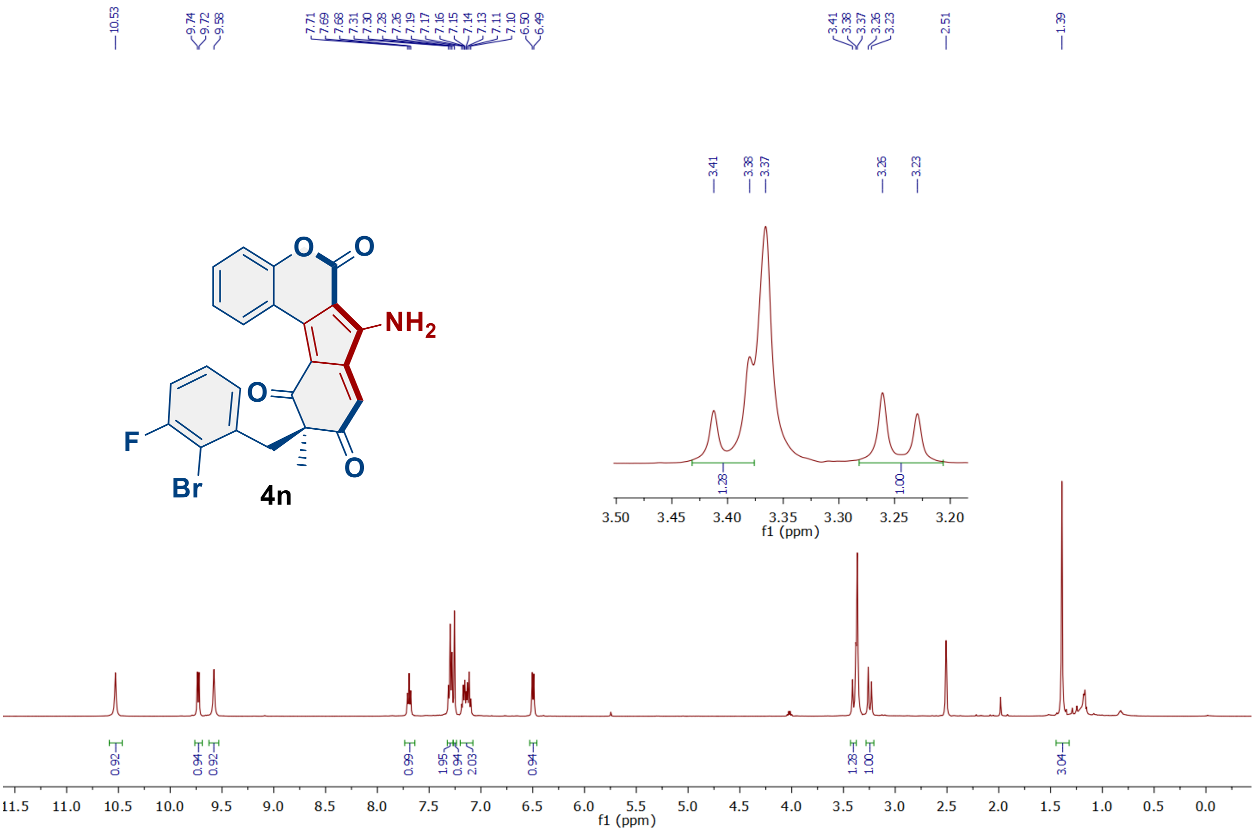

Supplementary Fig. 81. **^{1}H NMR (500 MHz, DMSO-d_6) spectrum for 4n.**

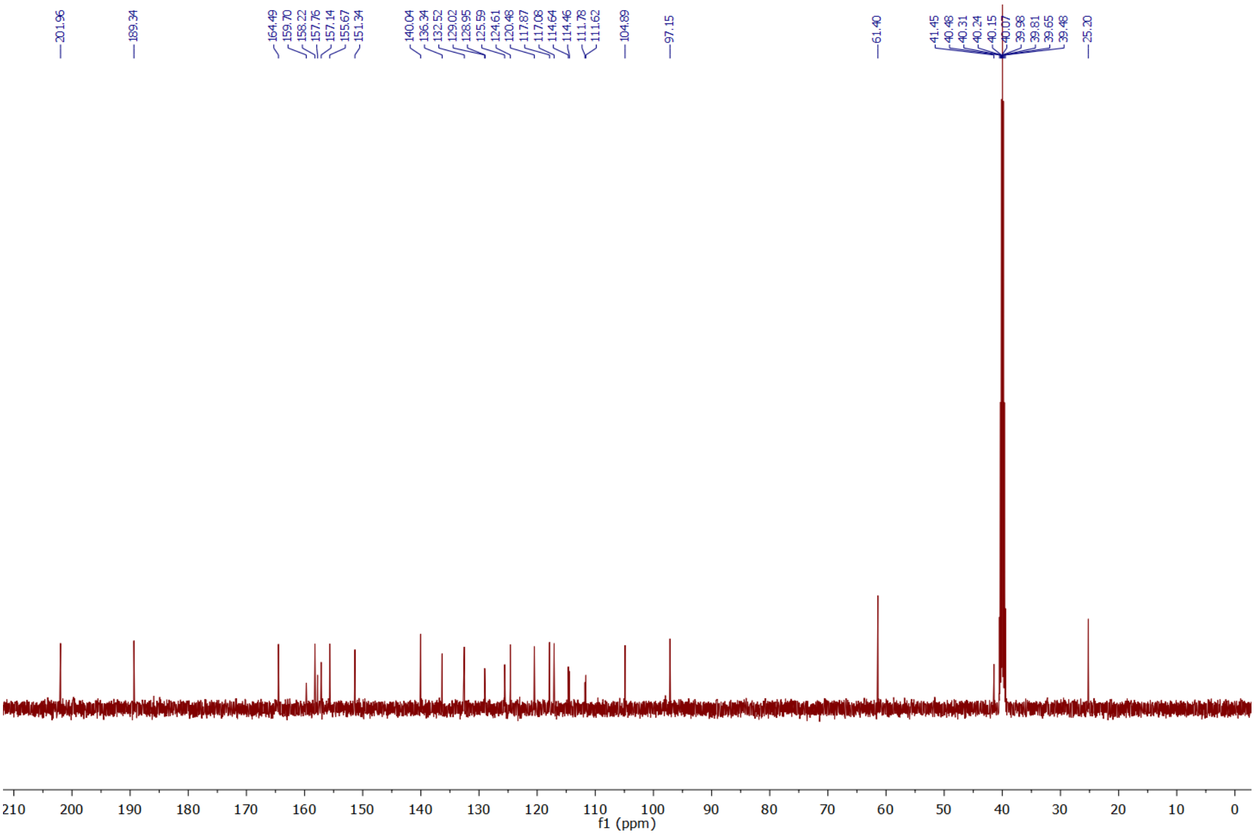

Supplementary Fig. 82. **^{13}C NMR (126 MHz, DMSO-d_6) spectrum for 4n.**

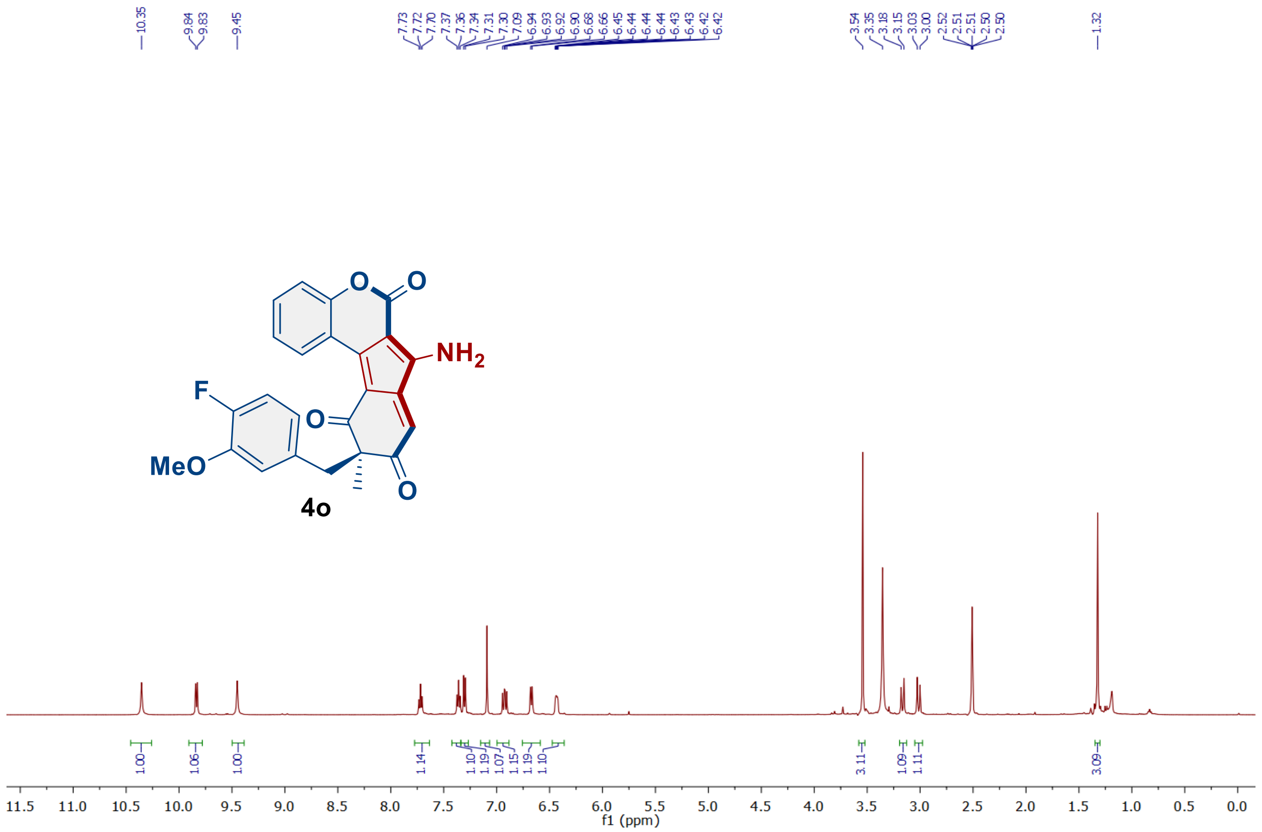

Supplementary Fig. 83. ^{1}H NMR (500 MHz, DMSO-d_6) spectrum for 4o.

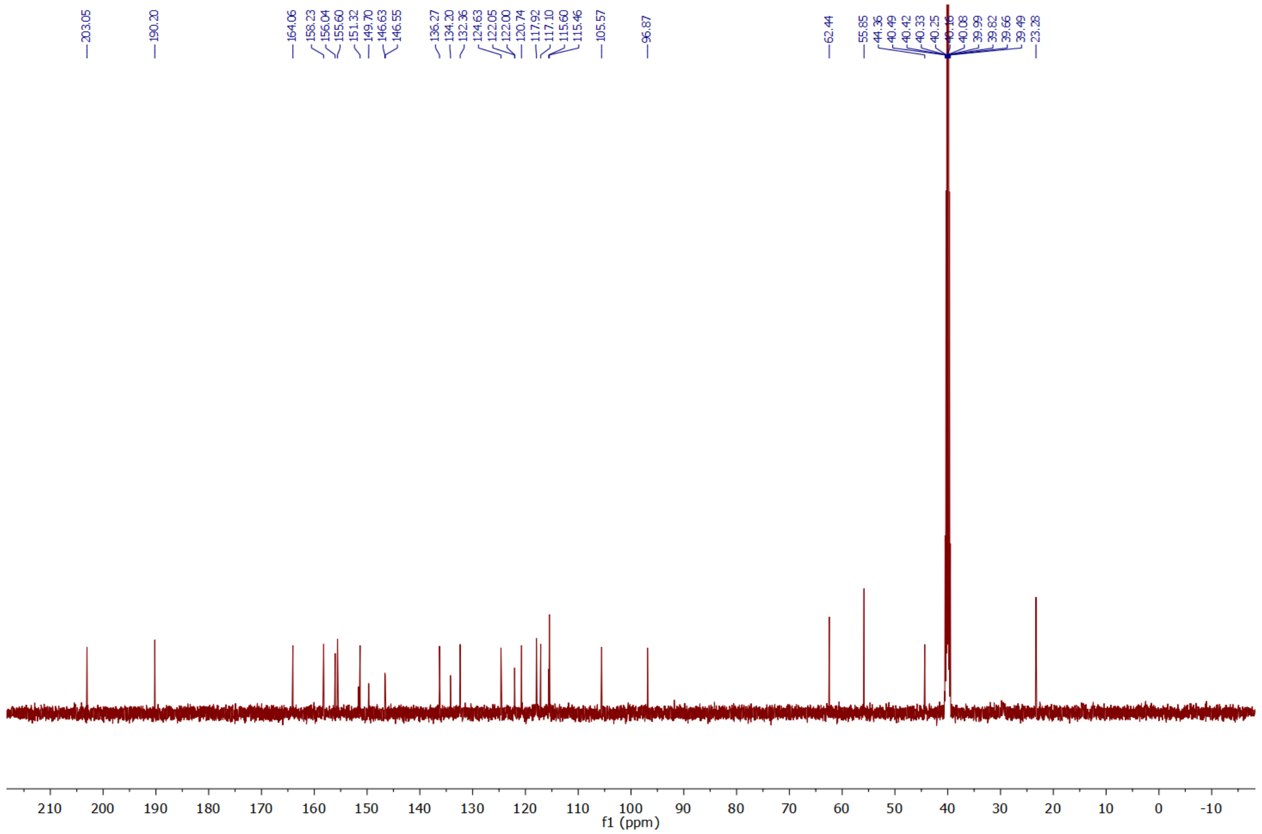

Supplementary Fig. 84. ^{13}C NMR (126 MHz, DMSO-d_6) spectrum for 4o.

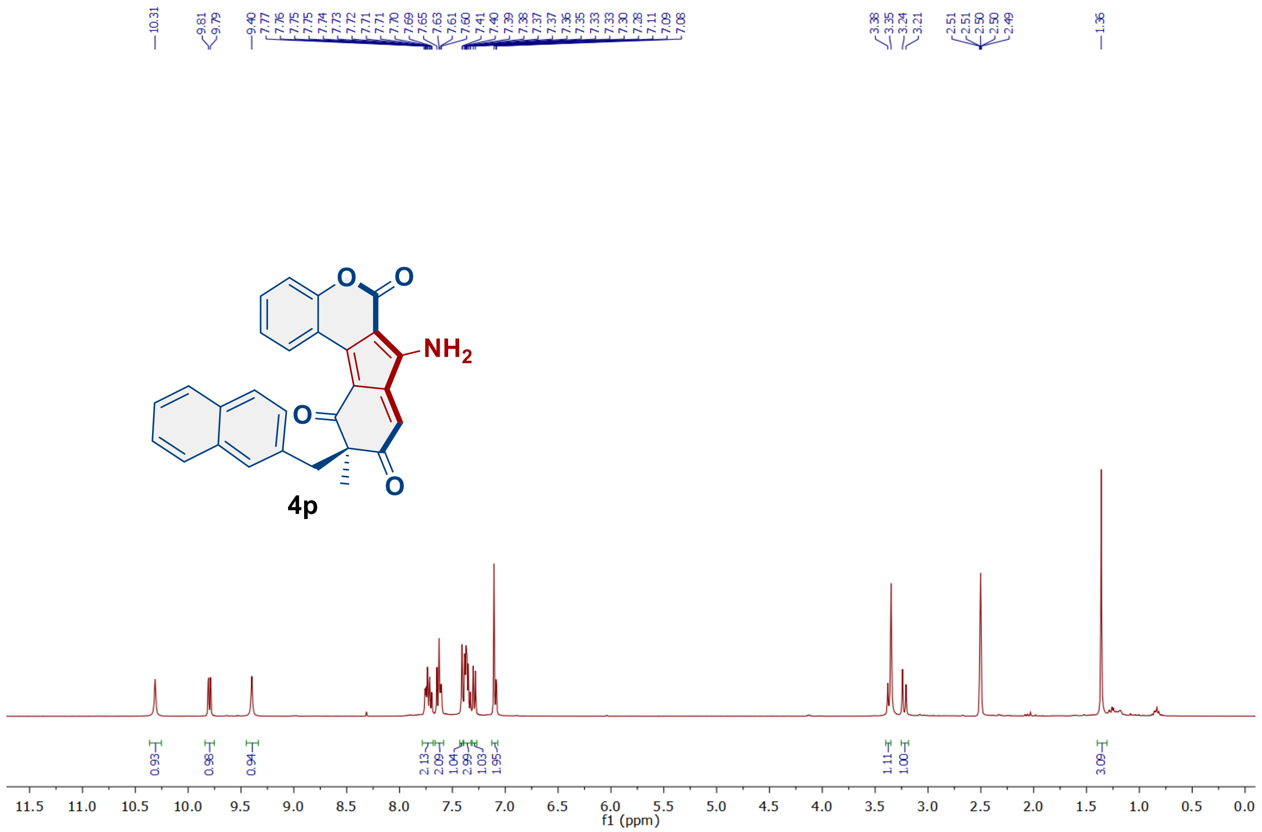

Supplementary Fig. 85. ^{1}**H NMR (500 MHz, DMSO-*d*$_6$) spectrum for 4p.**

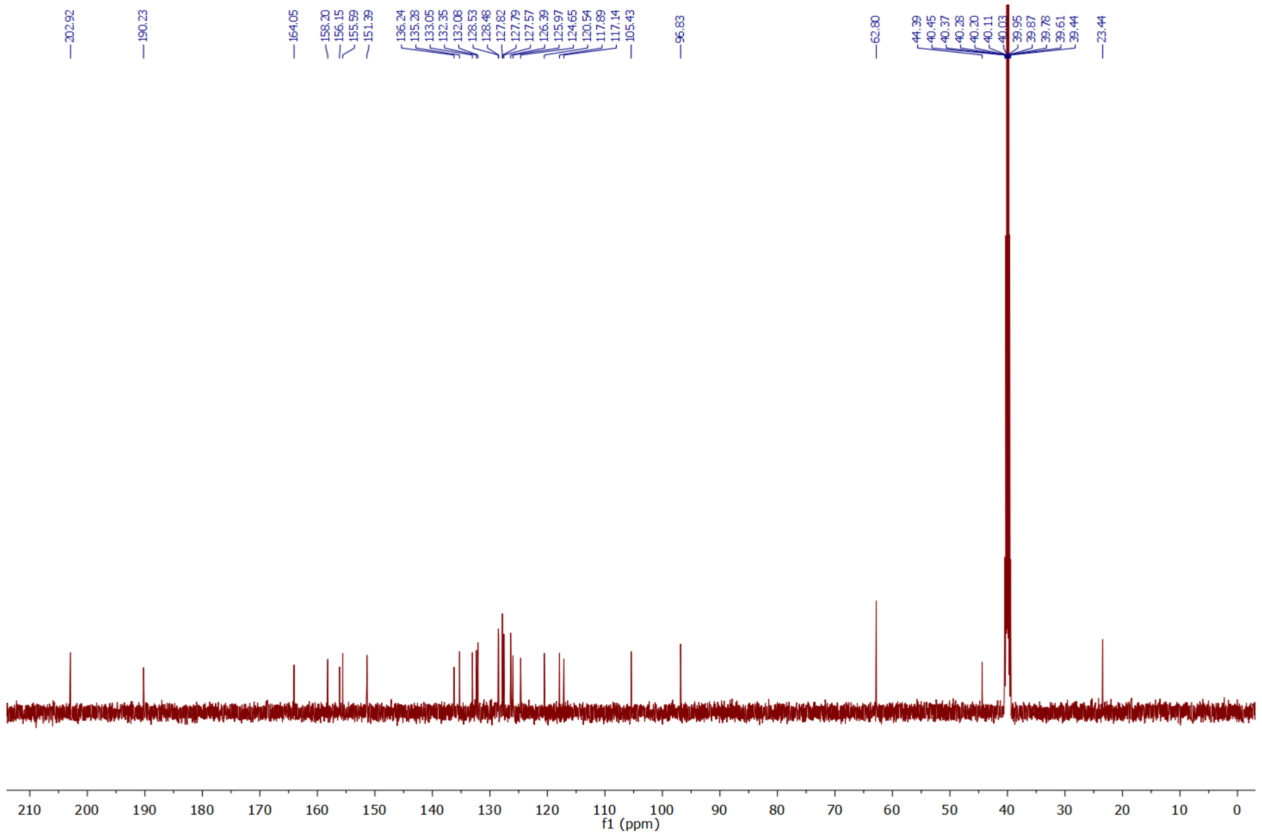

Supplementary Fig. 86. ^{13}**C NMR (126 MHz, DMSO-*d*$_6$) spectrum for 4p.**

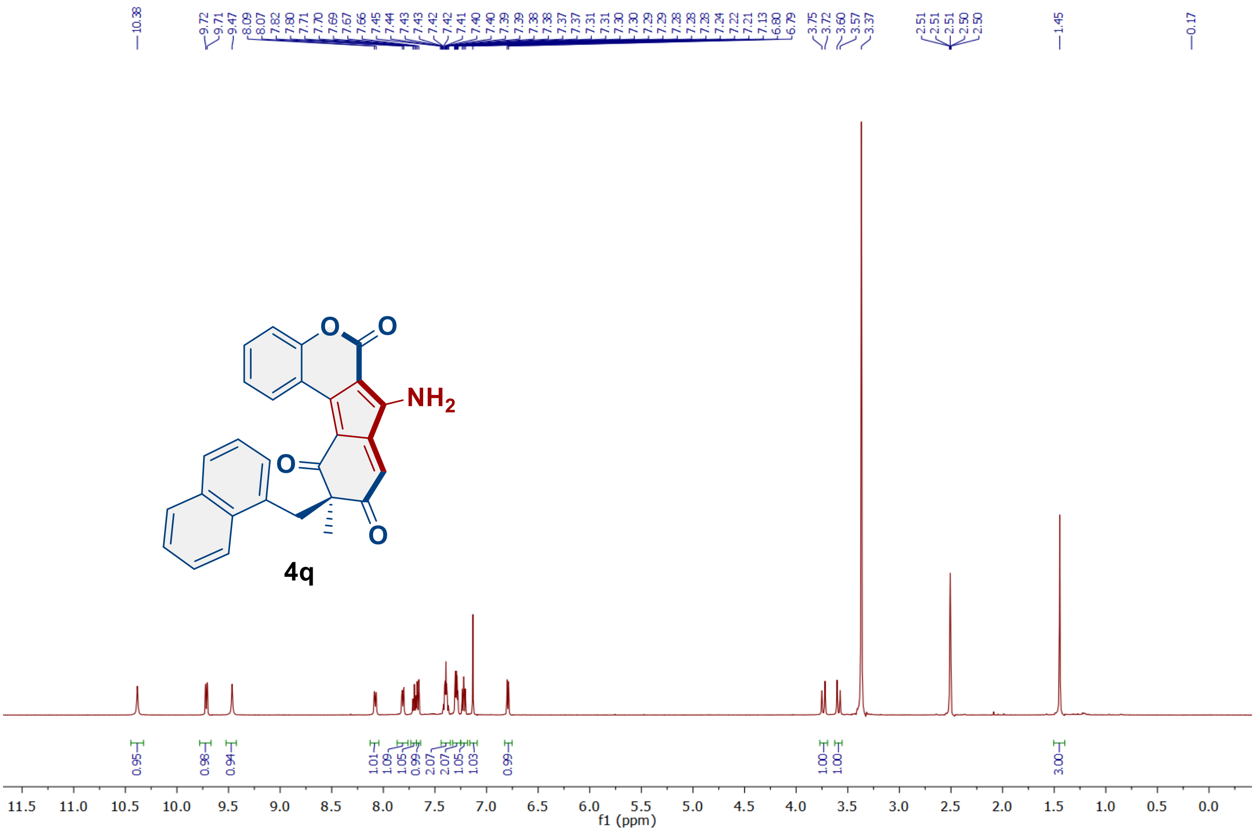

Supplementary Fig. 87. ^{1}H **(500 MHz, DMSO-*d*$_6$) spectrum for 4q.**

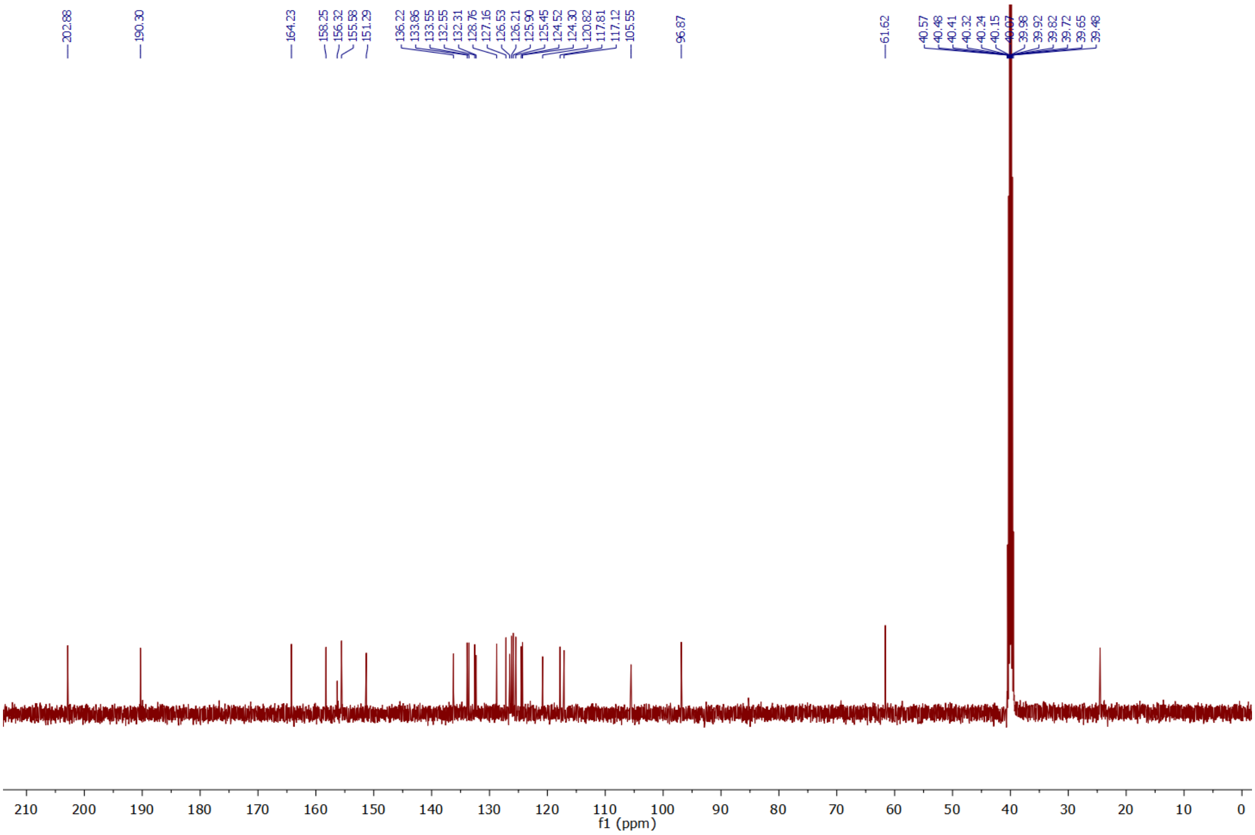

Supplementary Fig. 88. ^{13}C **NMR (126 MHz, DMSO-*d*$_6$) spectrum for 4q.**

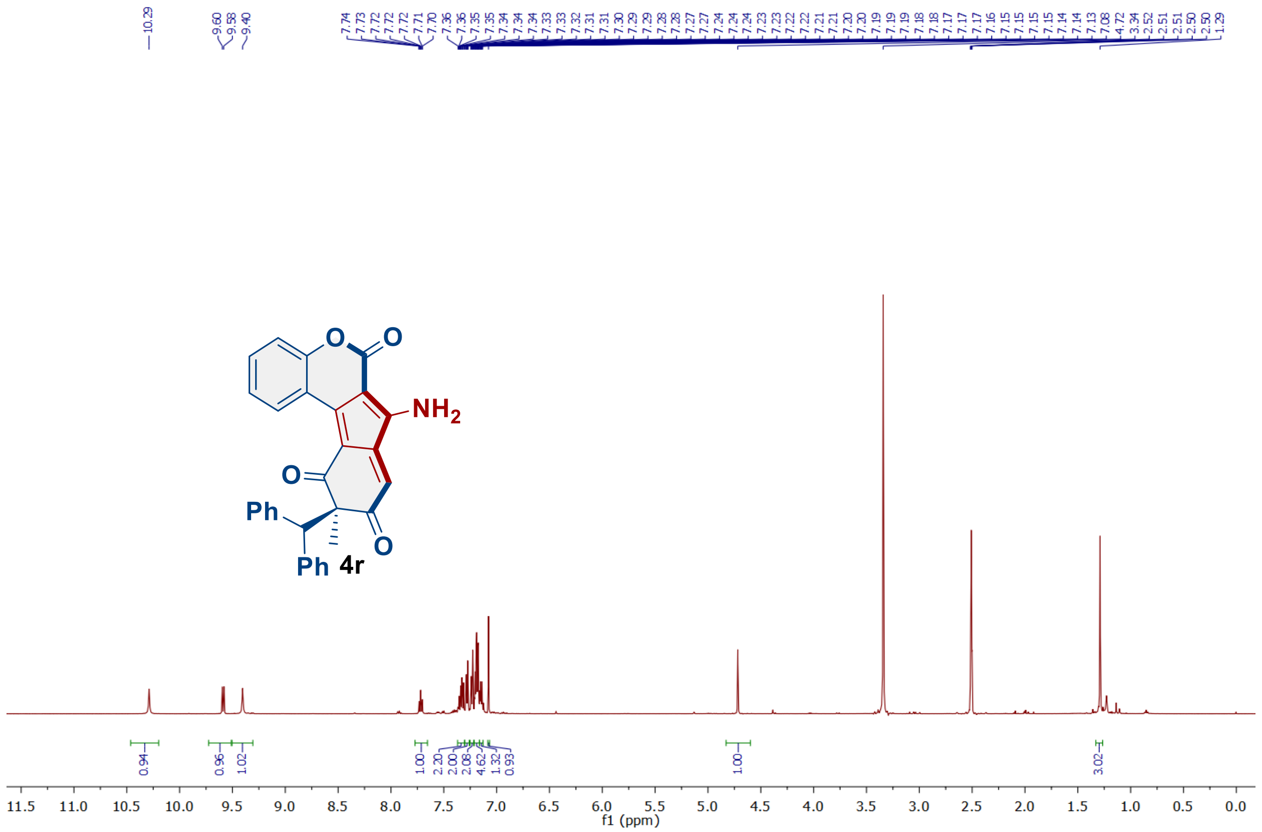

Supplementary Fig. 89. ^{1}**H NMR (500 MHz, DMSO-*d*$_6$) spectrum for 4r.**

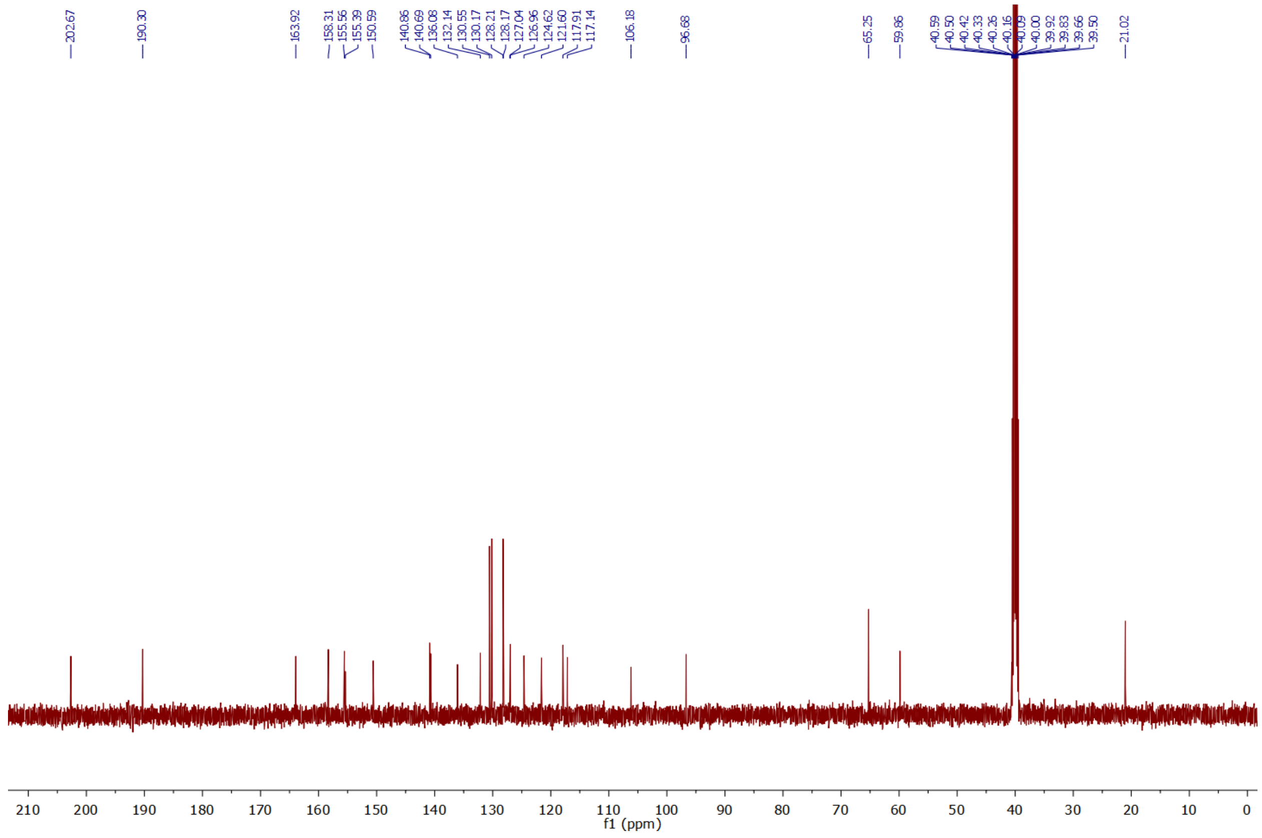

Supplementary Fig. 90. ^{13}**C NMR (126 MHz, DMSO-*d*$_6$) spectrum for 4r.**

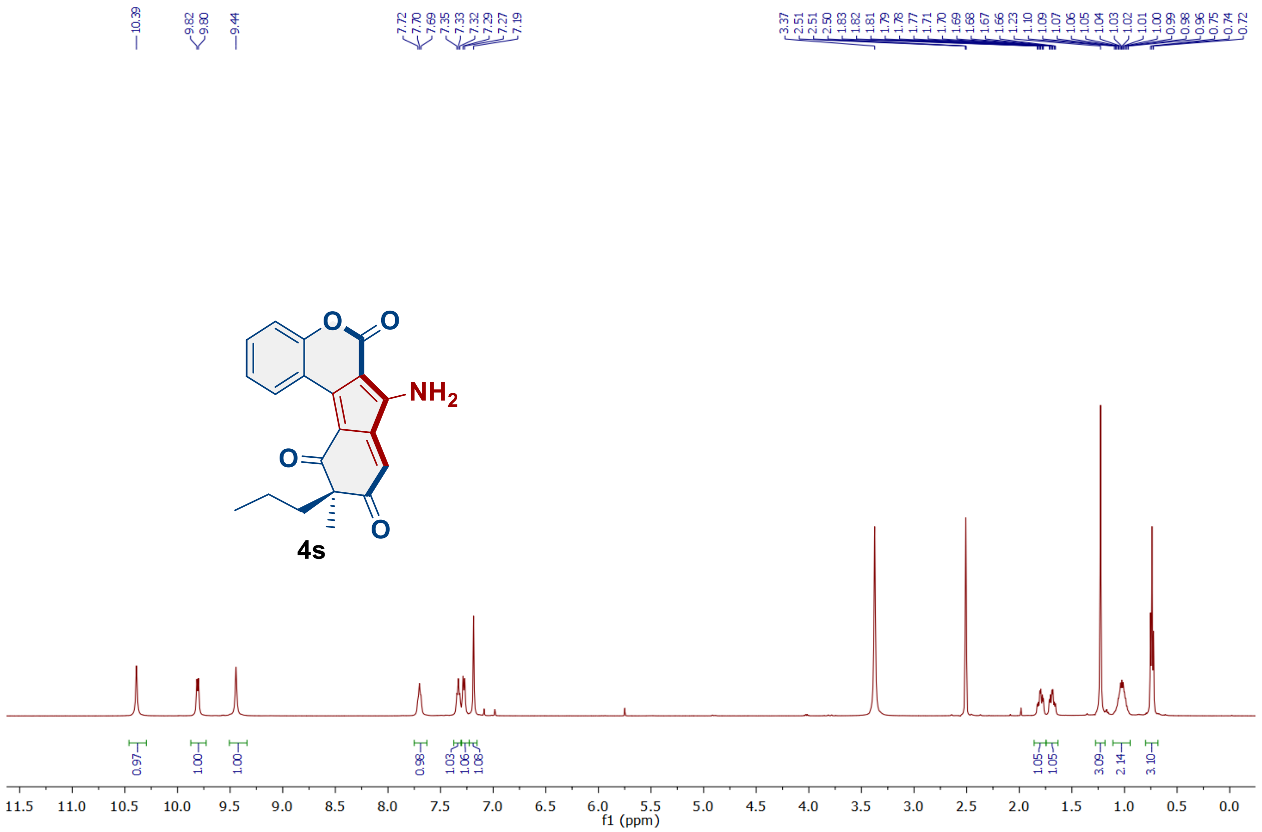

Supplementary Fig. 91. ^{1}H **NMR (500 MHz, DMSO-d_6) spectrum for 4s.**

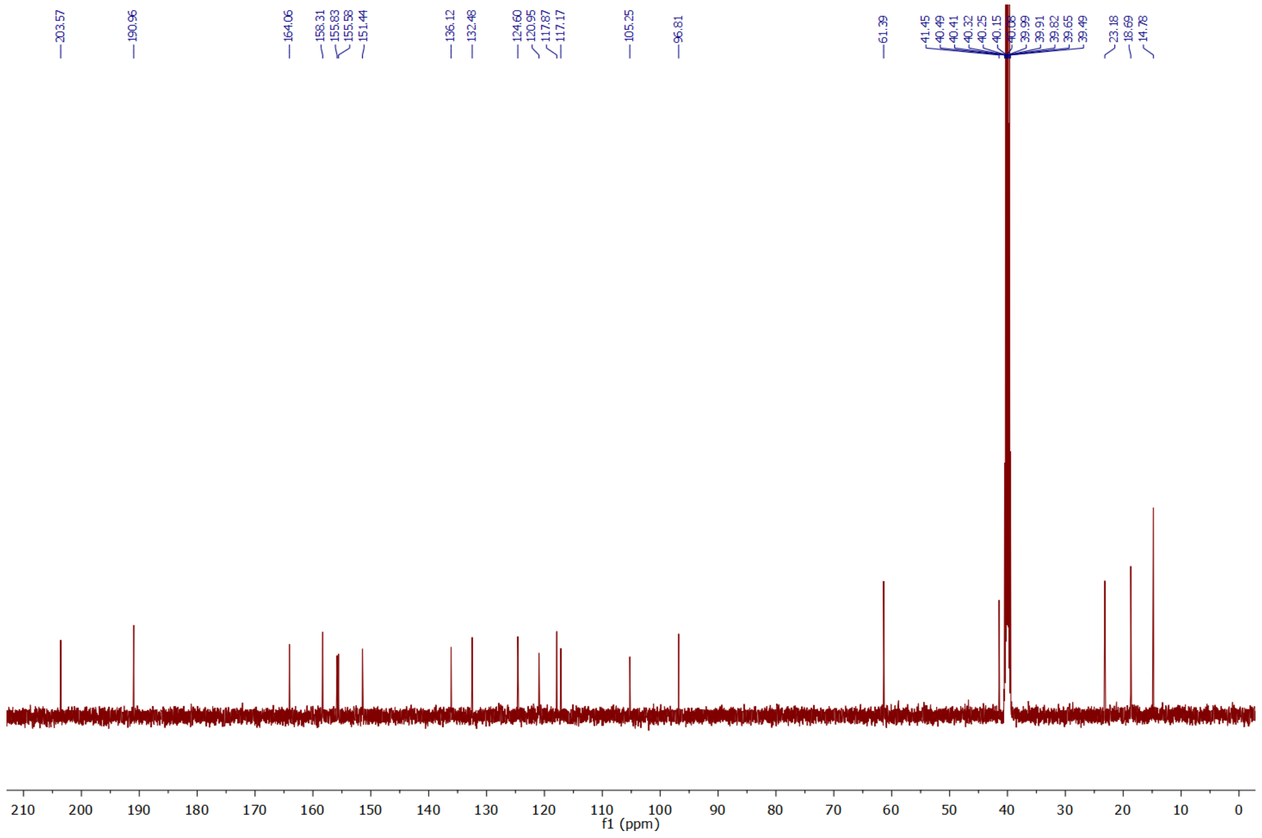

Supplementary Fig. 92. ^{13}C **NMR (126 MHz, DMSO-d_6) spectrum for 4s.**

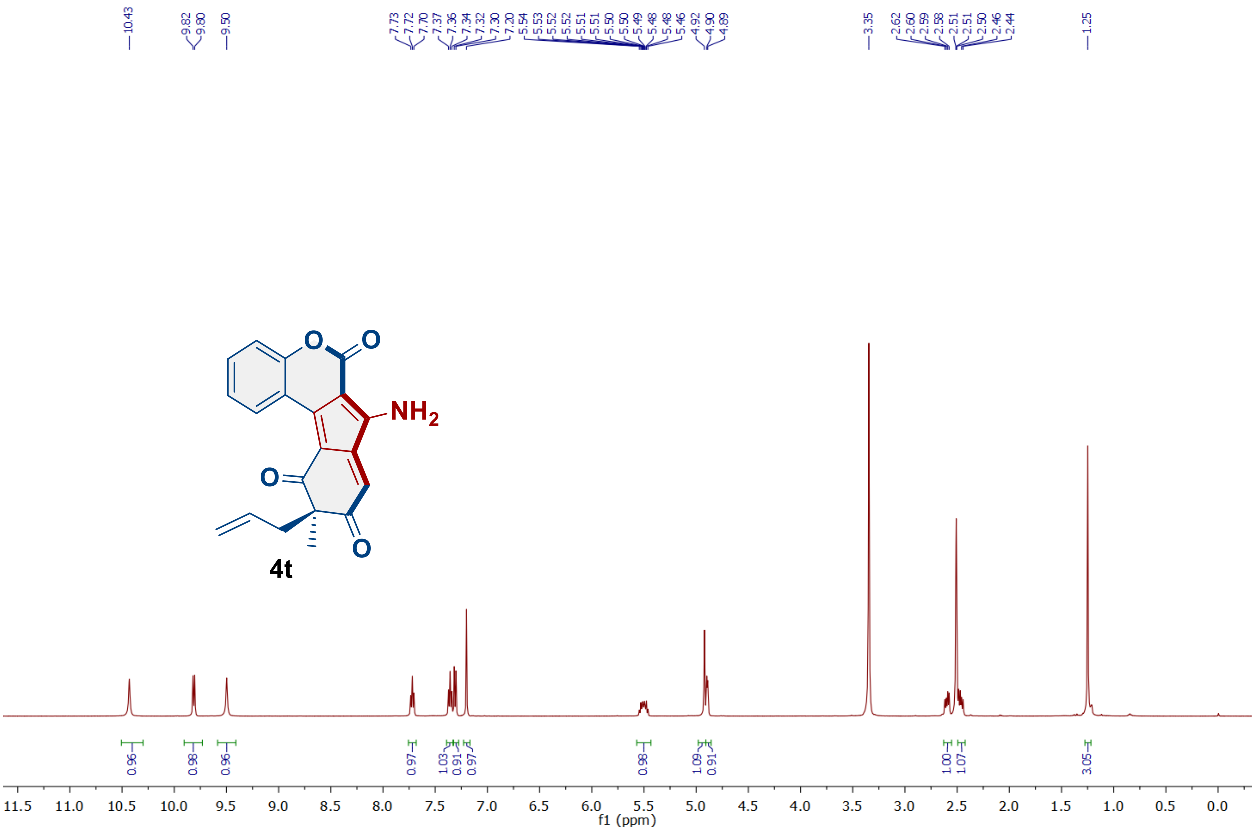

Supplementary Fig. 93. ^{1}**H NMR (500 MHz, DMSO-*d*$_6$) spectrum for 4t.**

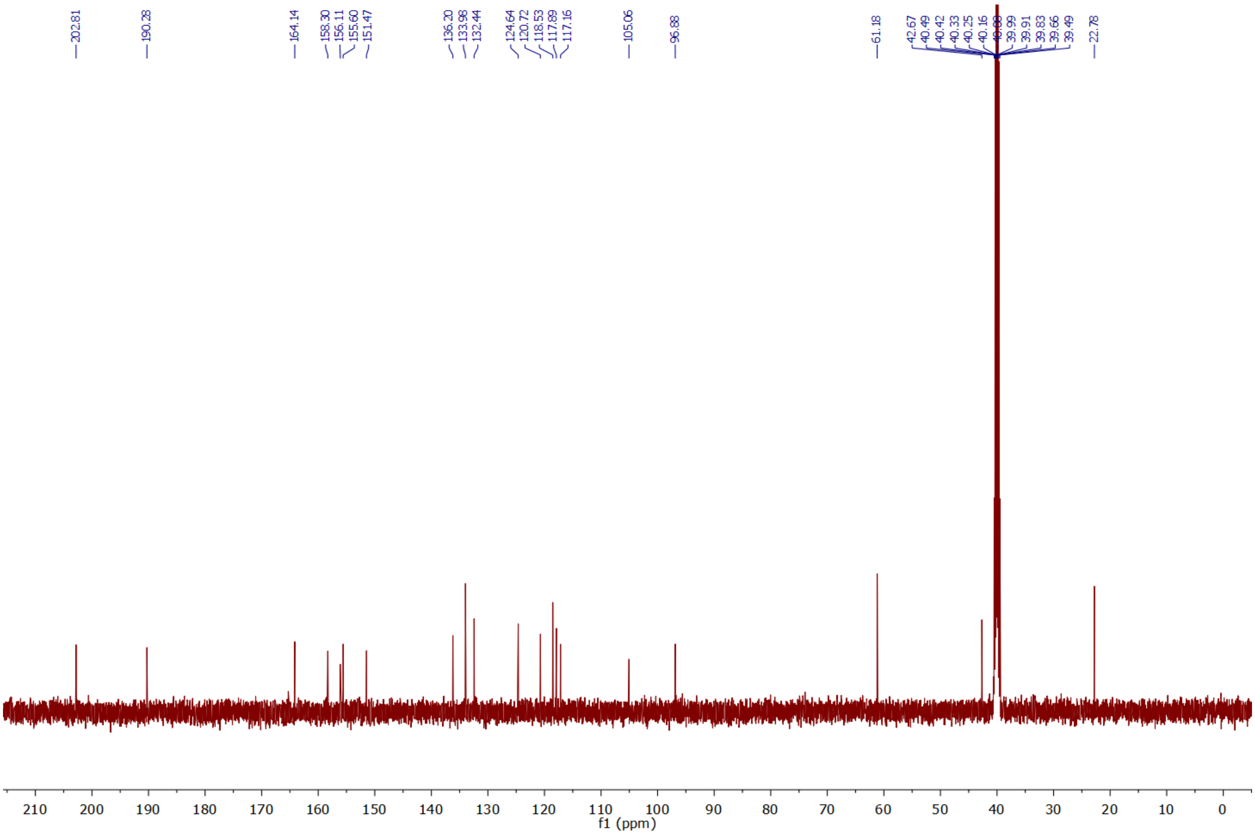

Supplementary Fig. 94. ^{13}**C NMR (126 MHz, DMSO-*d*$_6$) spectrum for 4t.**

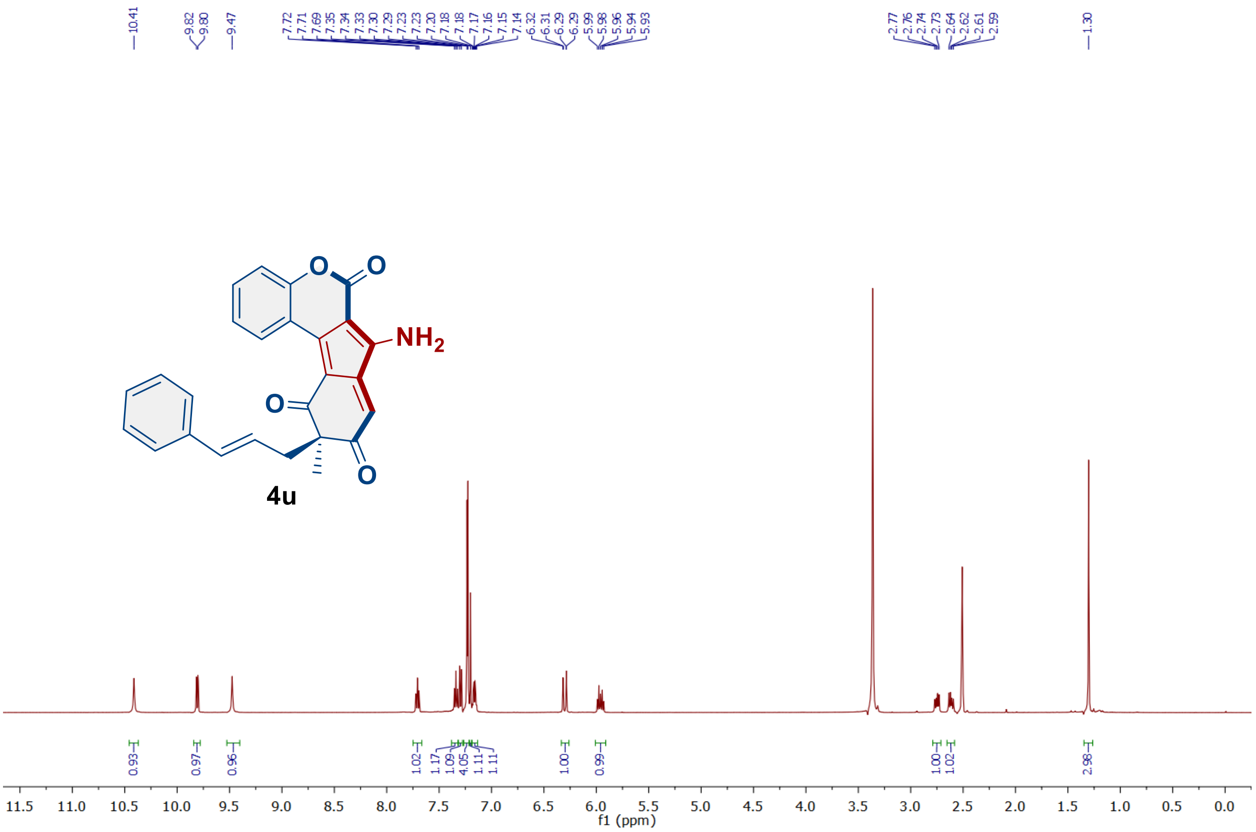

Supplementary Fig. 95. ^{1}**H NMR (500 MHz, DMSO-**d_6**) spectrum for 4u.**

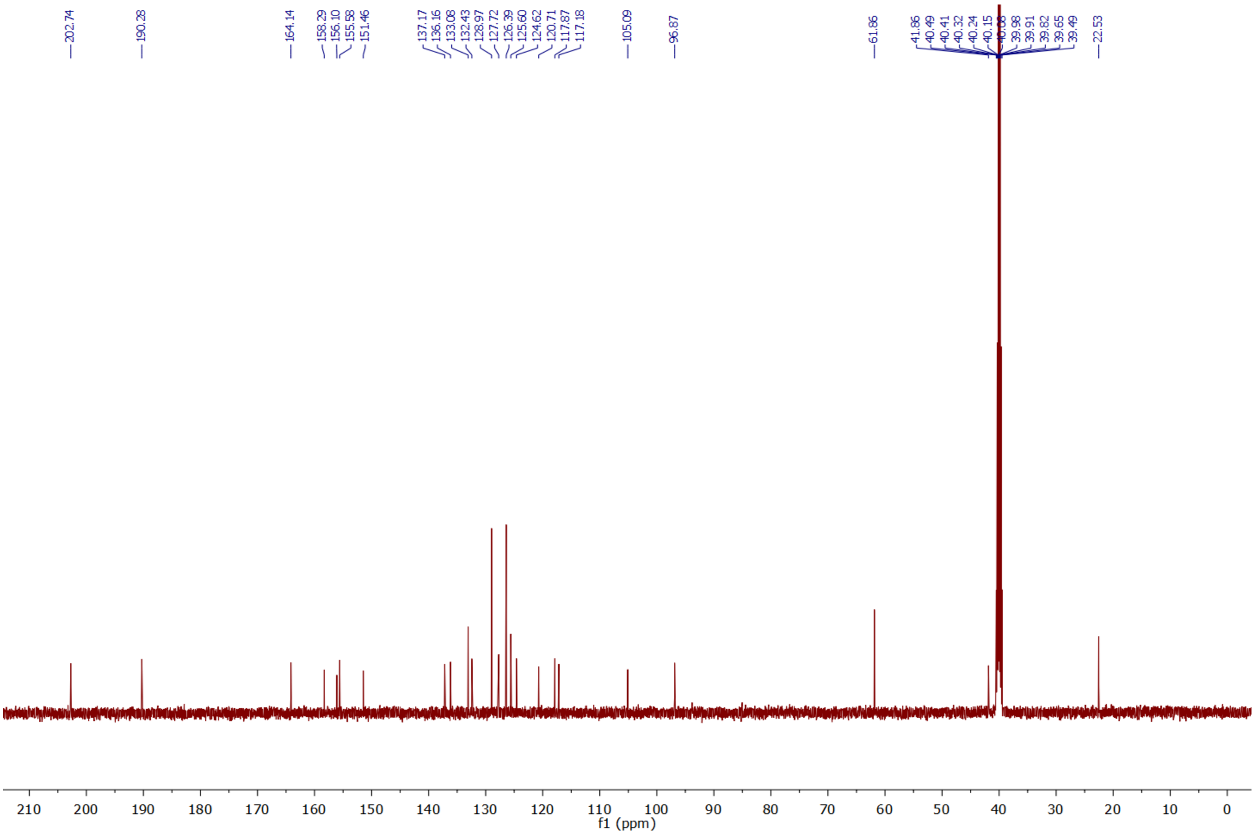

Supplementary Fig. 96. ^{13}**C NMR (126 MHz, DMSO-**d_6**) spectrum for 4u.**

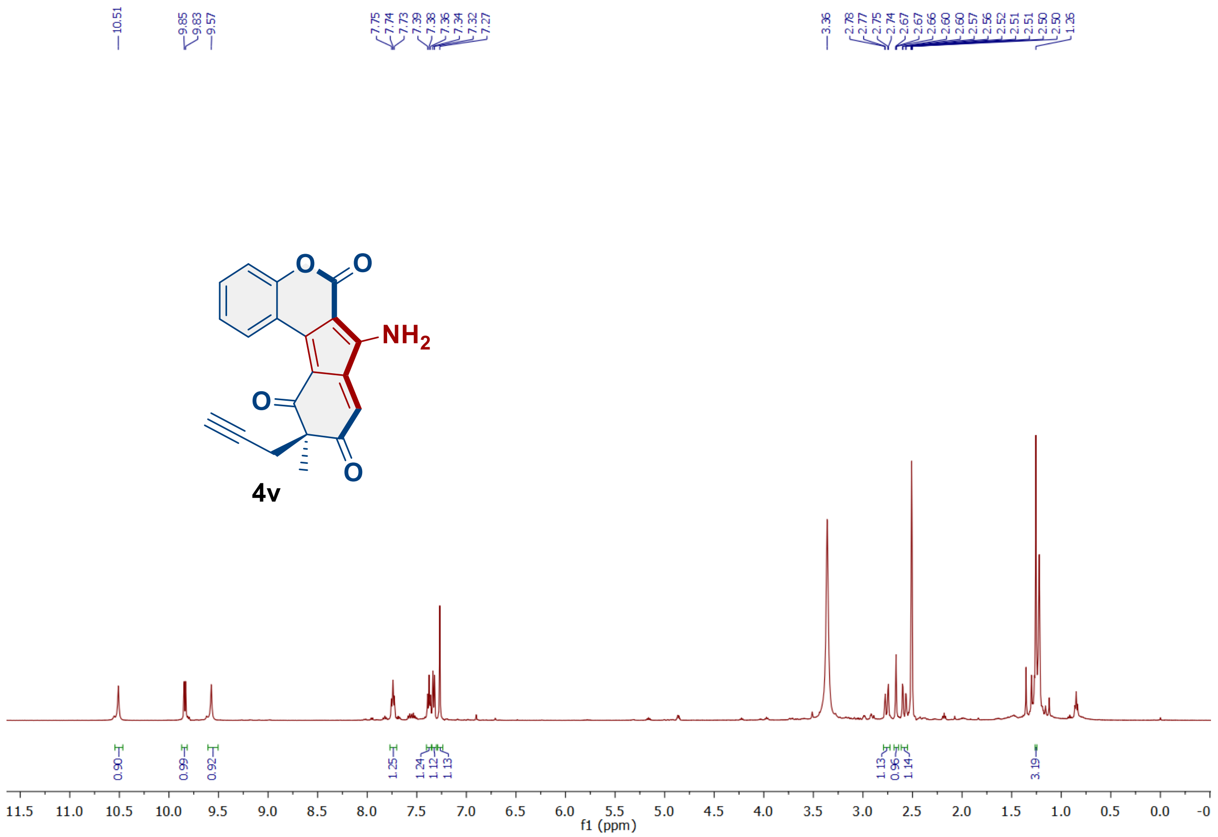

Supplementary Fig. 97. ^{1}**H NMR (500 MHz, DMSO-*d*$_6$) spectrum for 4v.**

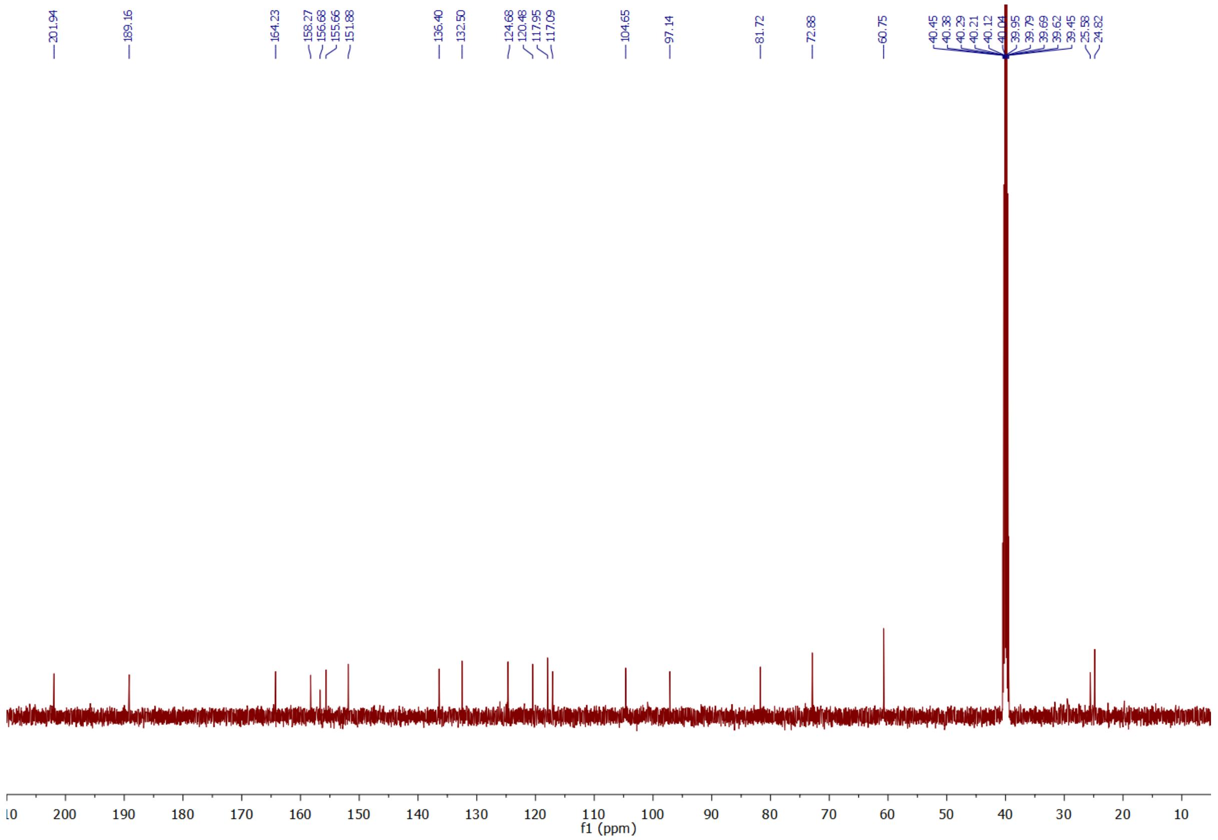

Supplementary Fig. 98. ^{13}**C NMR (126 MHz, DMSO-*d*$_6$) spectrum for 4v.**

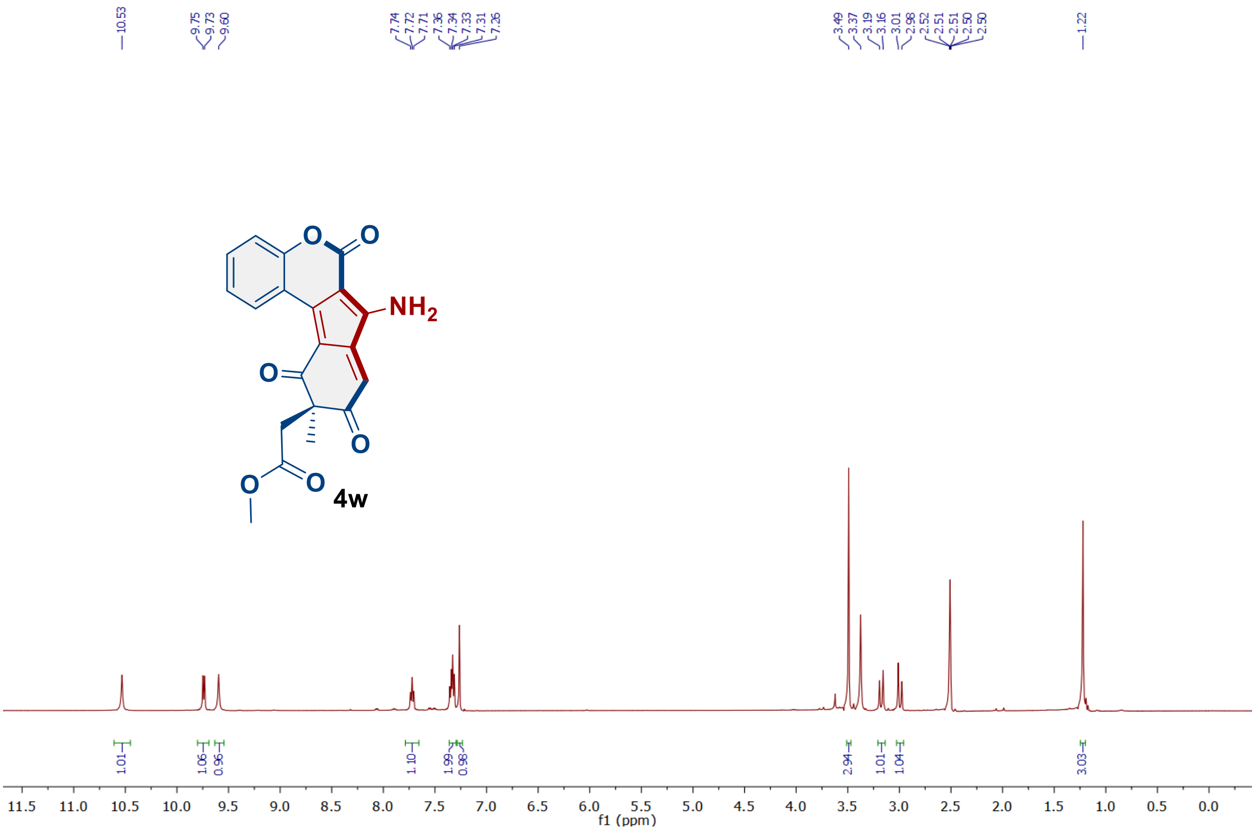

Supplementary Fig. 99. ^{1}H NMR (500 MHz, DMSO-d_6) spectrum for 4w.

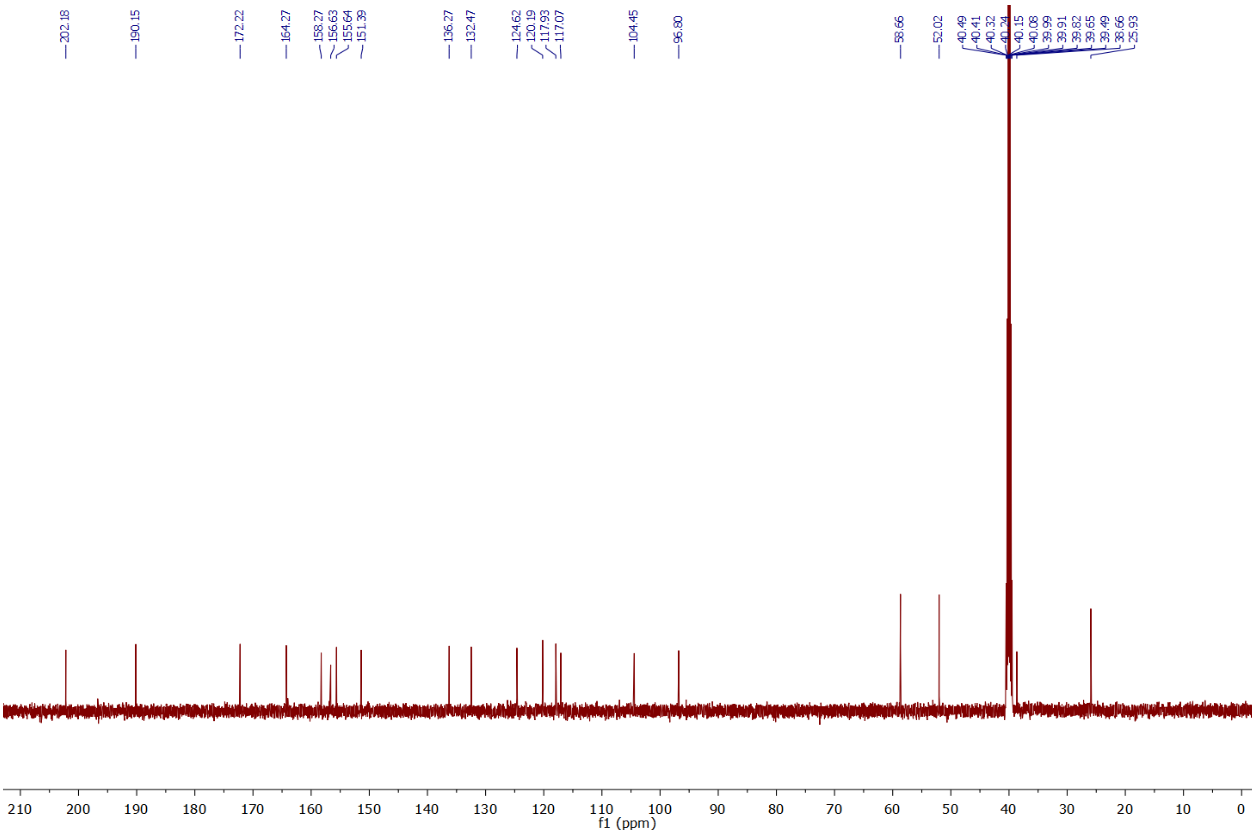

Supplementary Fig. 100. ^{13}C NMR (126 MHz, DMSO-d_6) spectrum for 4w.

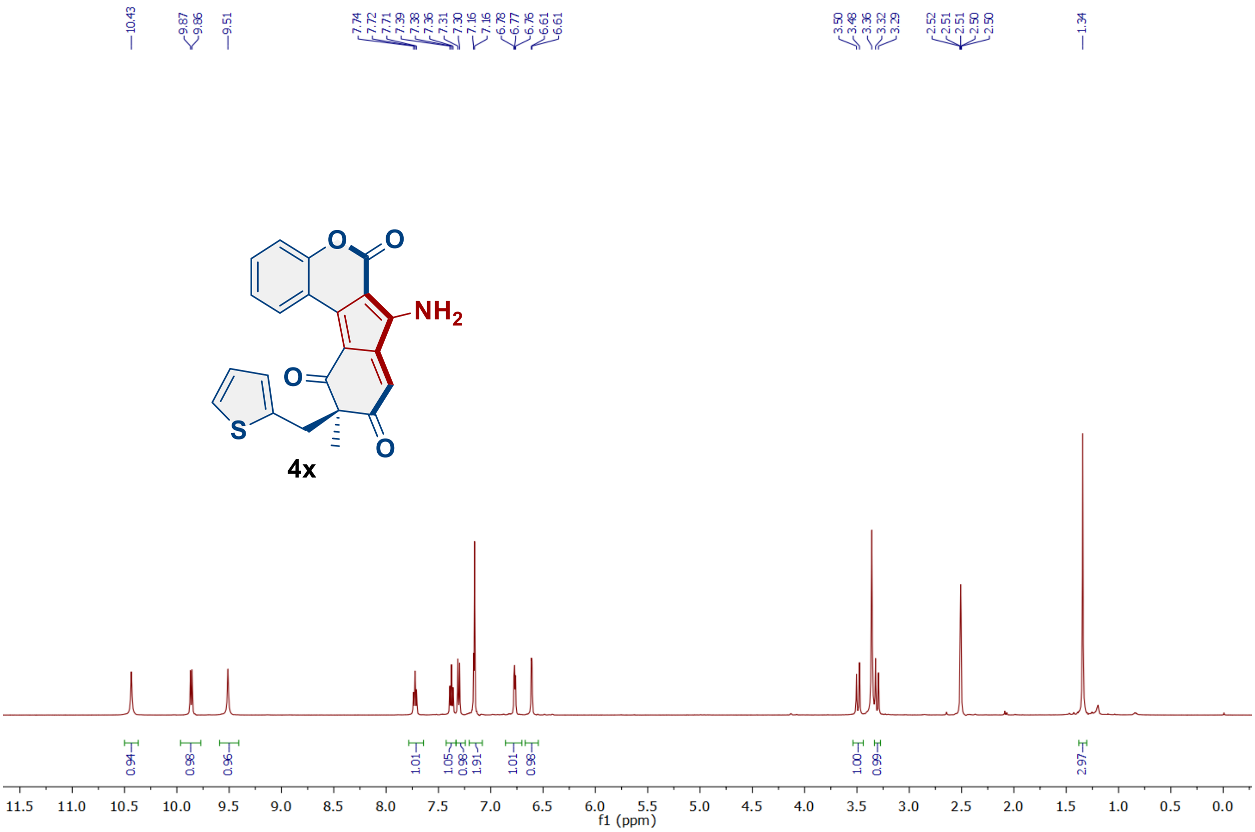

Supplementary Fig. 101. ^{1}H NMR (500 MHz, DMSO-d_6) spectrum for 4x.

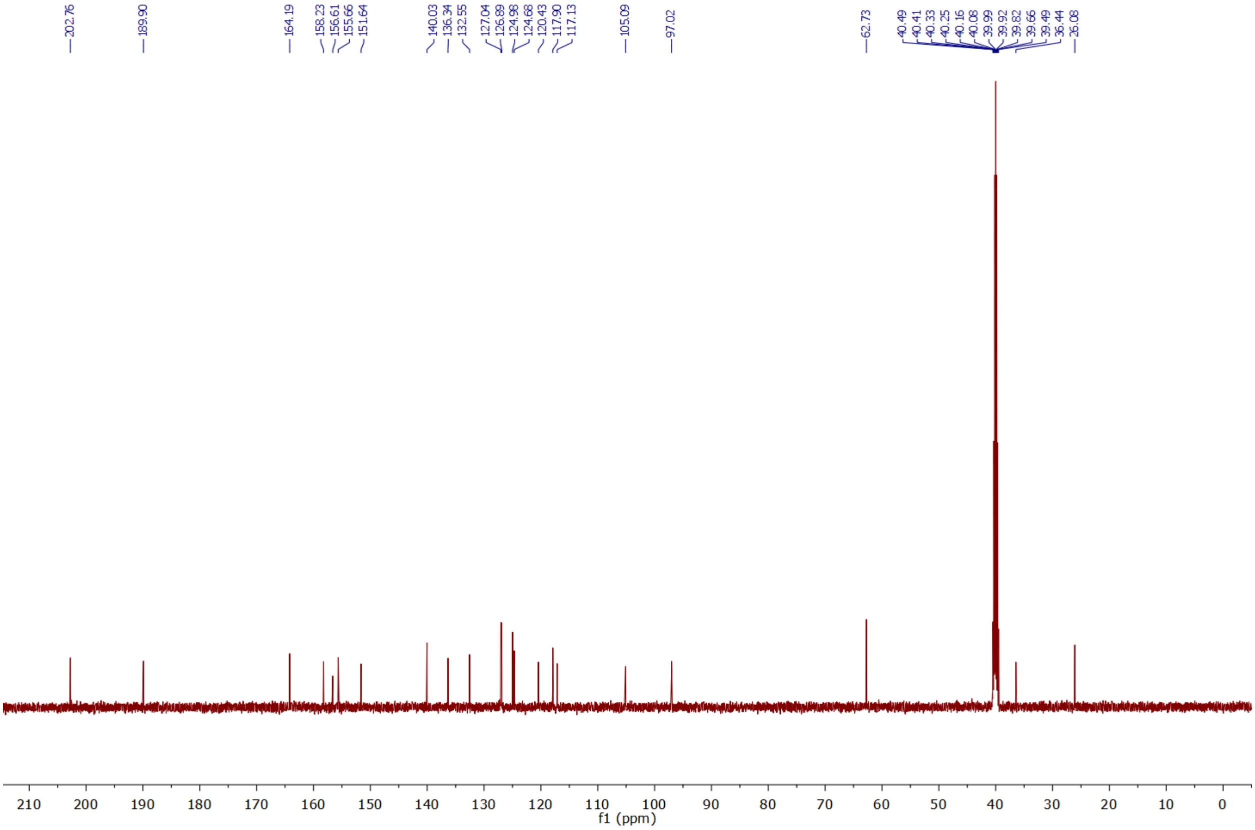

Supplementary Fig. 102. ^{13}C NMR (126 MHz, DMSO-d_6) spectrum for 4x.

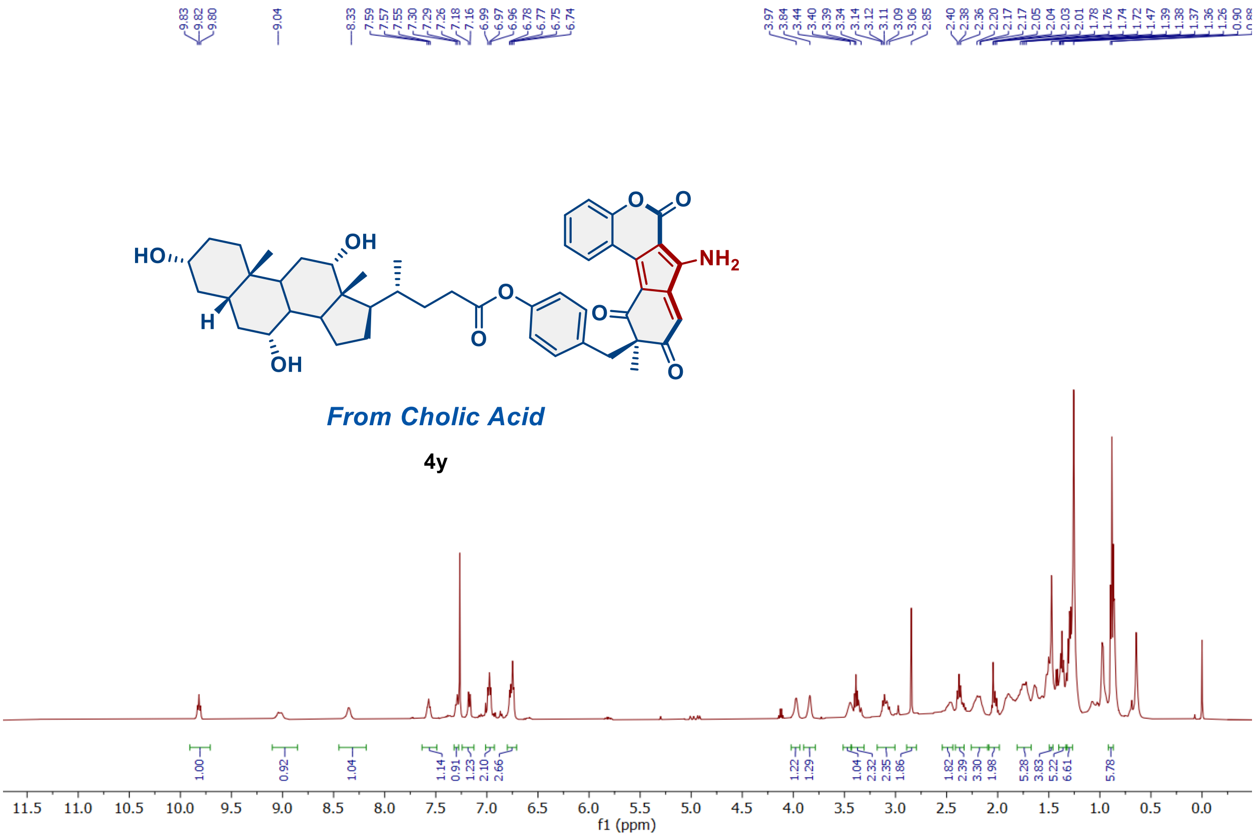

Supplementary Fig. 103. ^{1}H NMR (500 MHz, $CDCl_3$) spectrum for 4y.

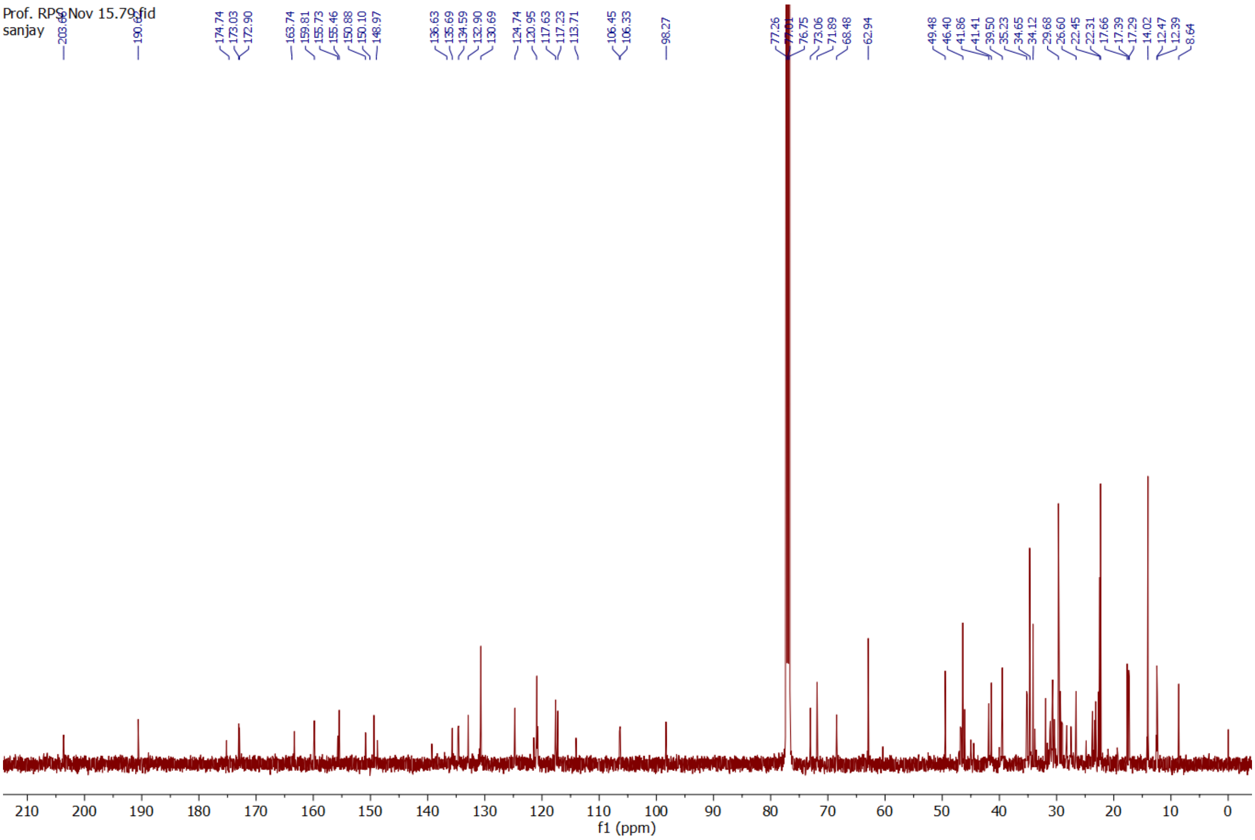

Supplementary Fig. 104. ^{13}C NMR (126 MHz, $CDCl_3$) spectrum for 4y.

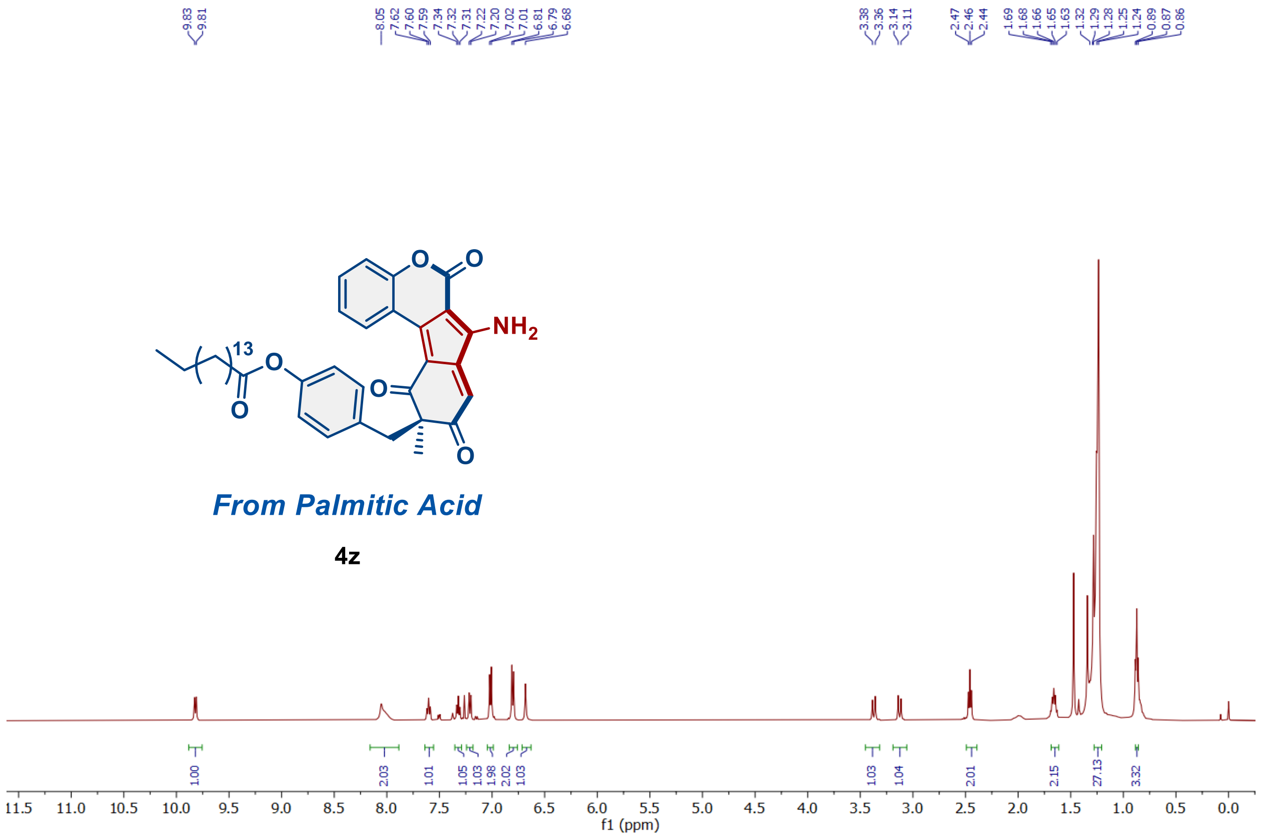

Supplementary Fig. 105. ^{1}H NMR (500 MHz, $CDCl_3$) spectrum for 4z.

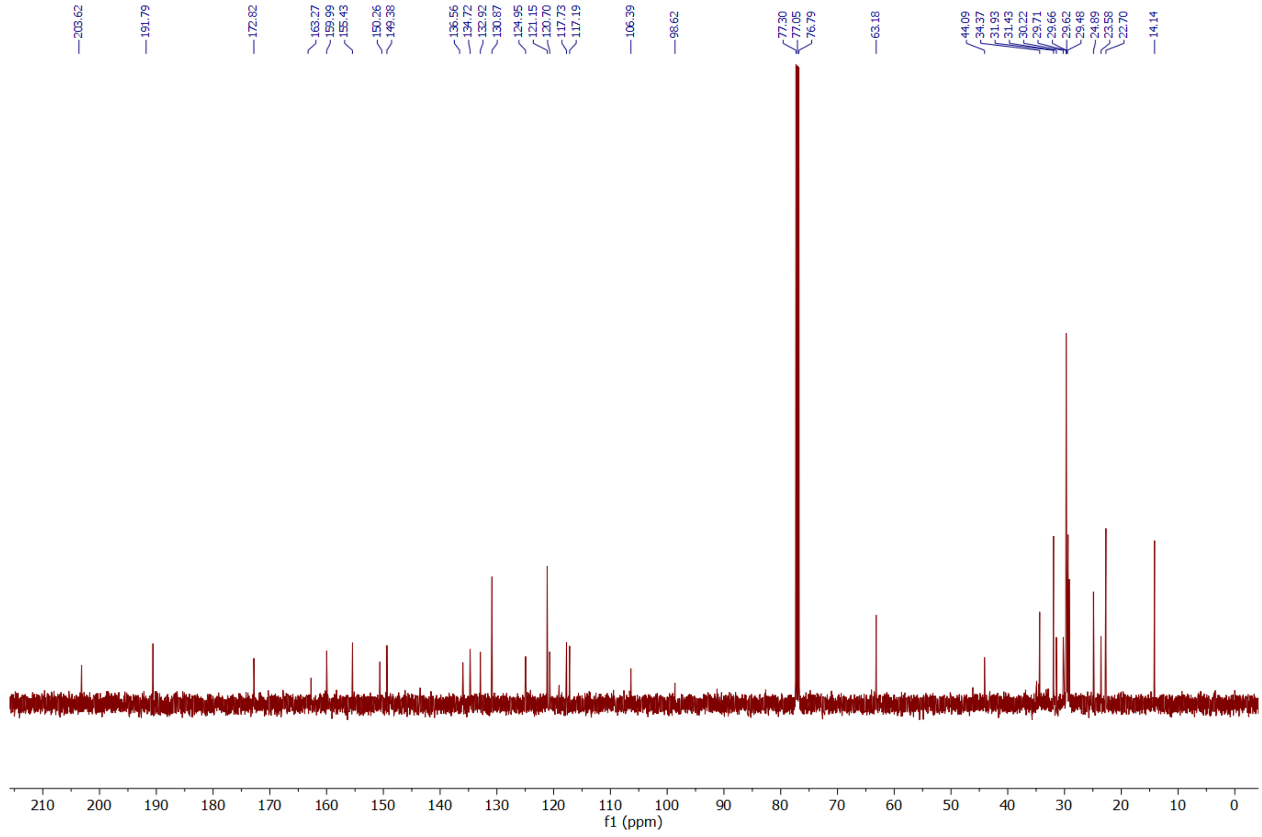

Supplementary Fig. 106. ^{13}C NMR (126 MHz, $CDCl_3$) spectrum for 4z.

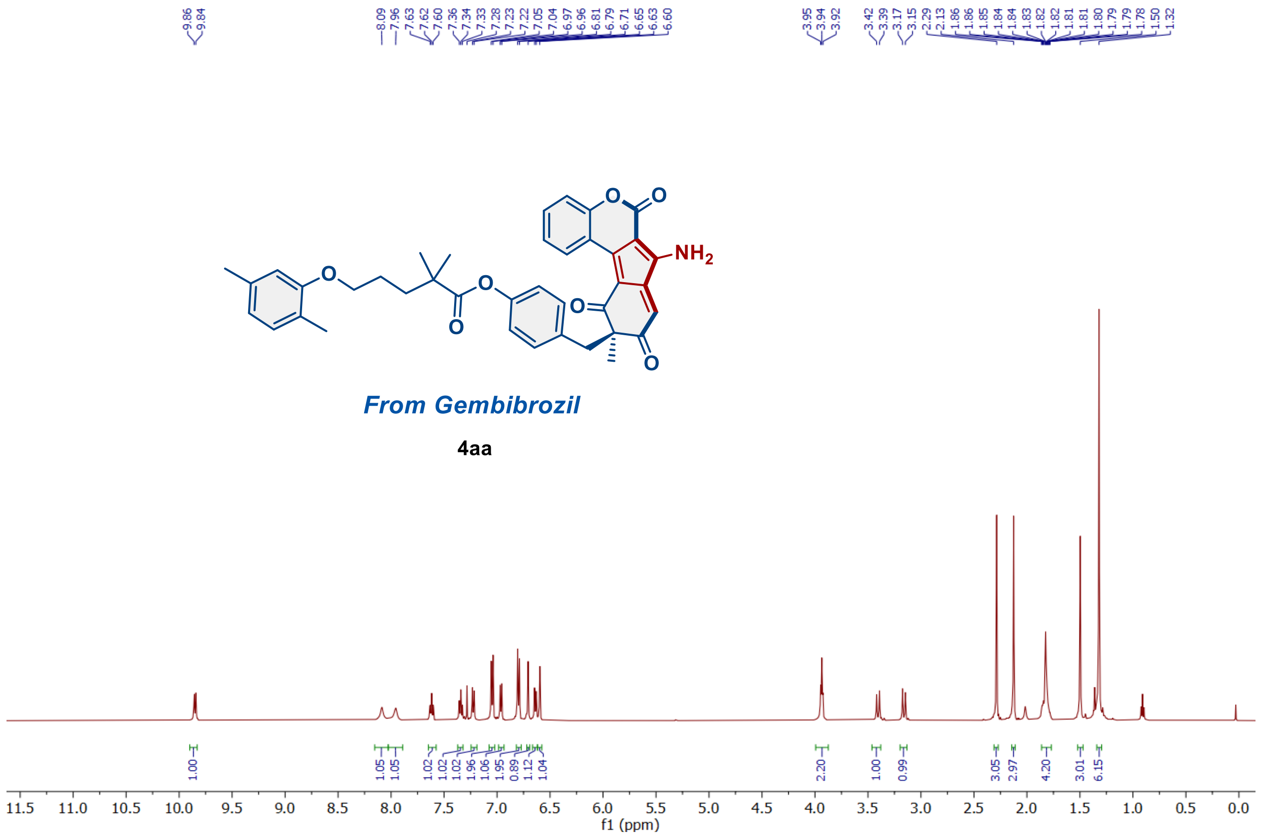

Supplementary Fig. 107. ^{1}H NMR (500 MHz, $CDCl_3$) spectrum for 4aa.

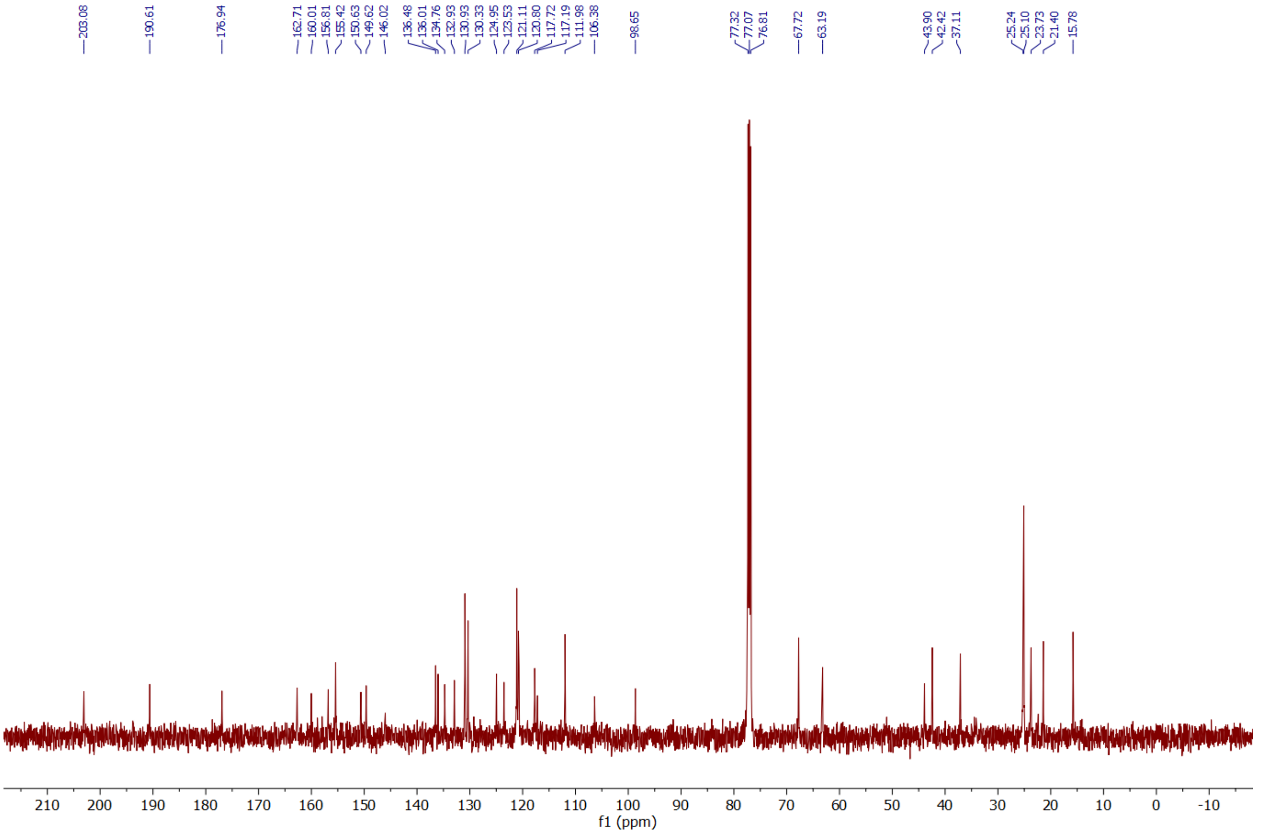

Supplementary Fig. 108. ^{13}C NMR (126 MHz, $CDCl_3$) spectrum for 4aa.

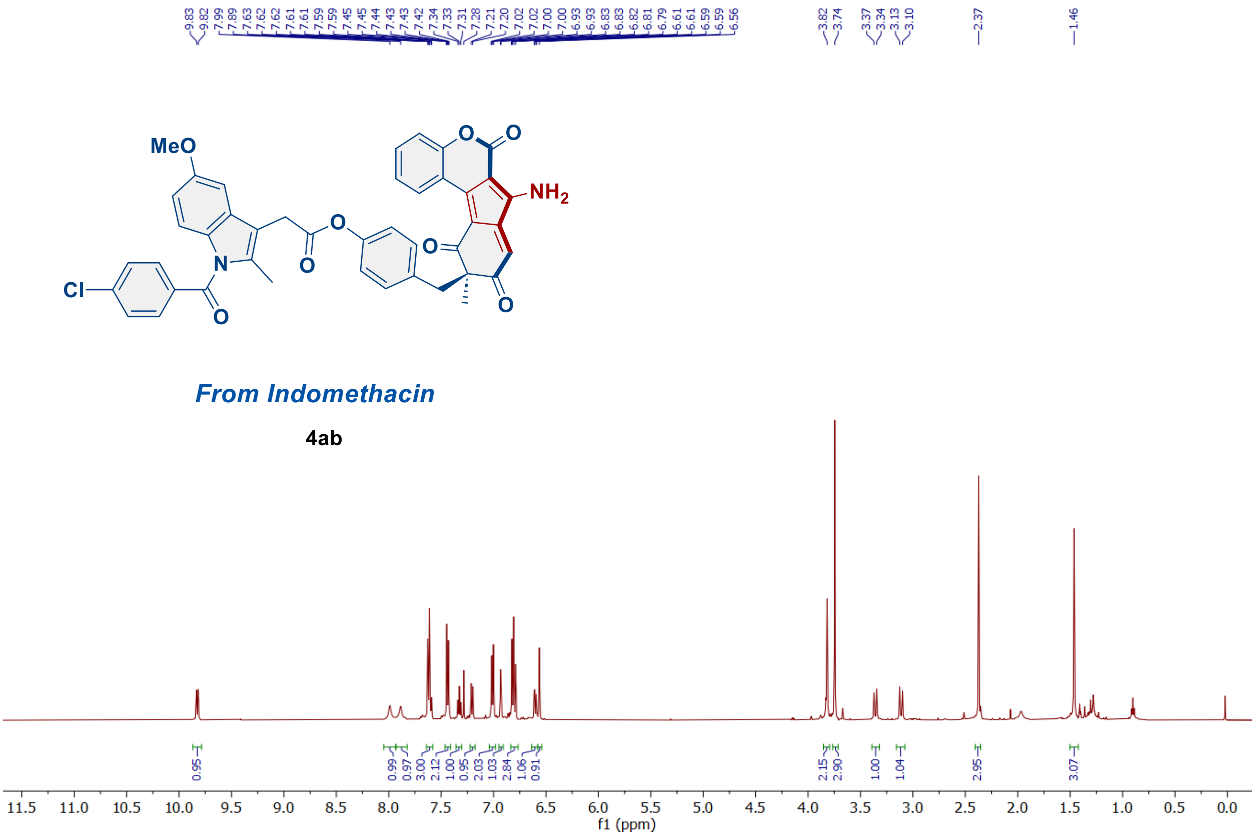

Supplementary Fig. 109. ^{1}H NMR (500 MHz, $CDCl_3$) spectrum for 4ab.

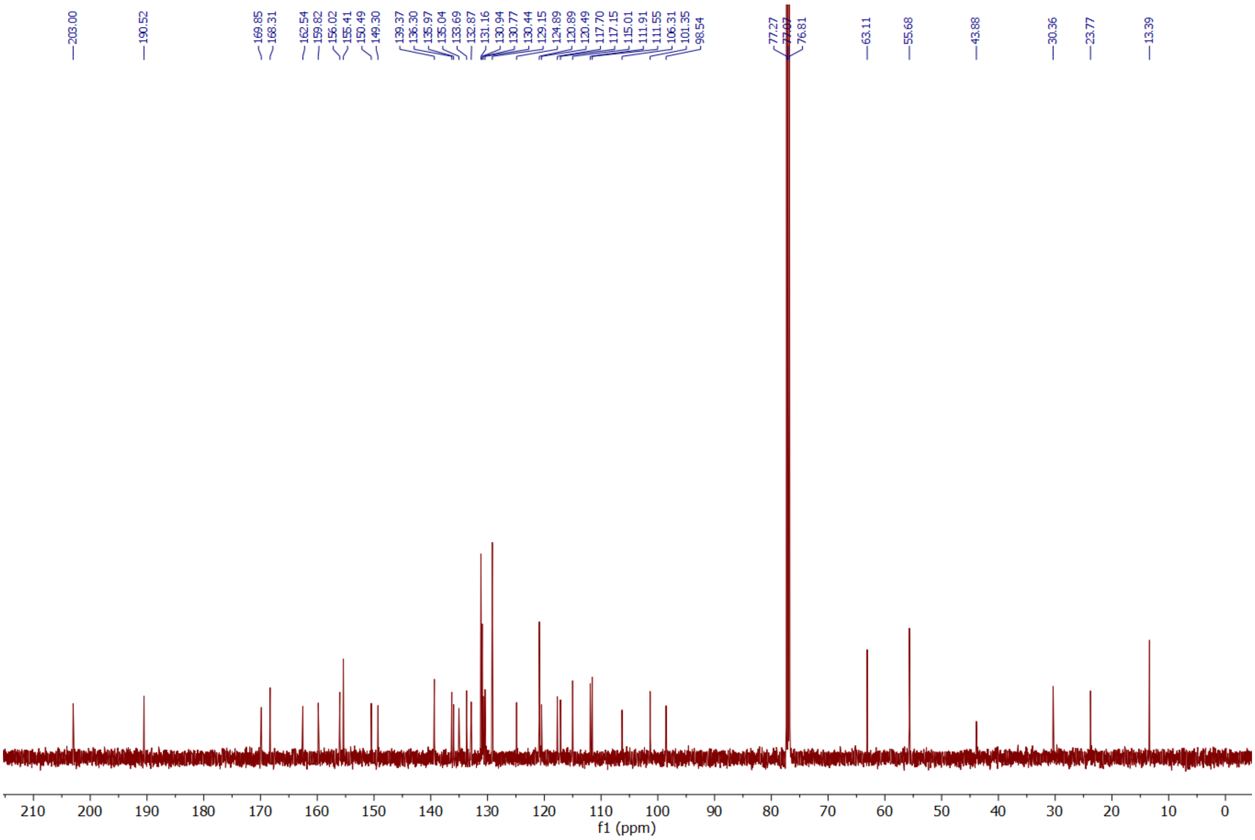

Supplementary Fig. 110. ^{13}C NMR (126 MHz, $CDCl_3$) spectrum for 4ab.

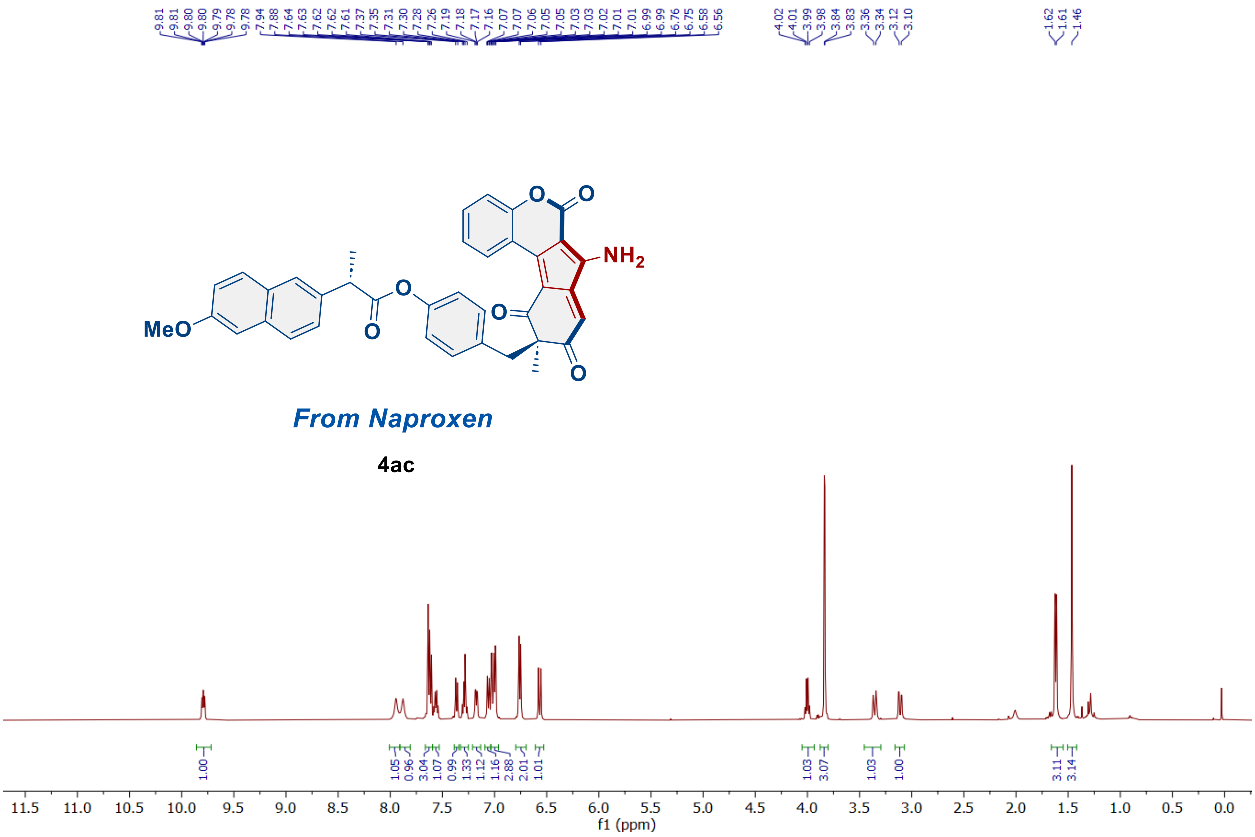

Supplementary Fig. 111. ^{1}H NMR (500 MHz, $CDCl_3$) spectrum for 4ac.

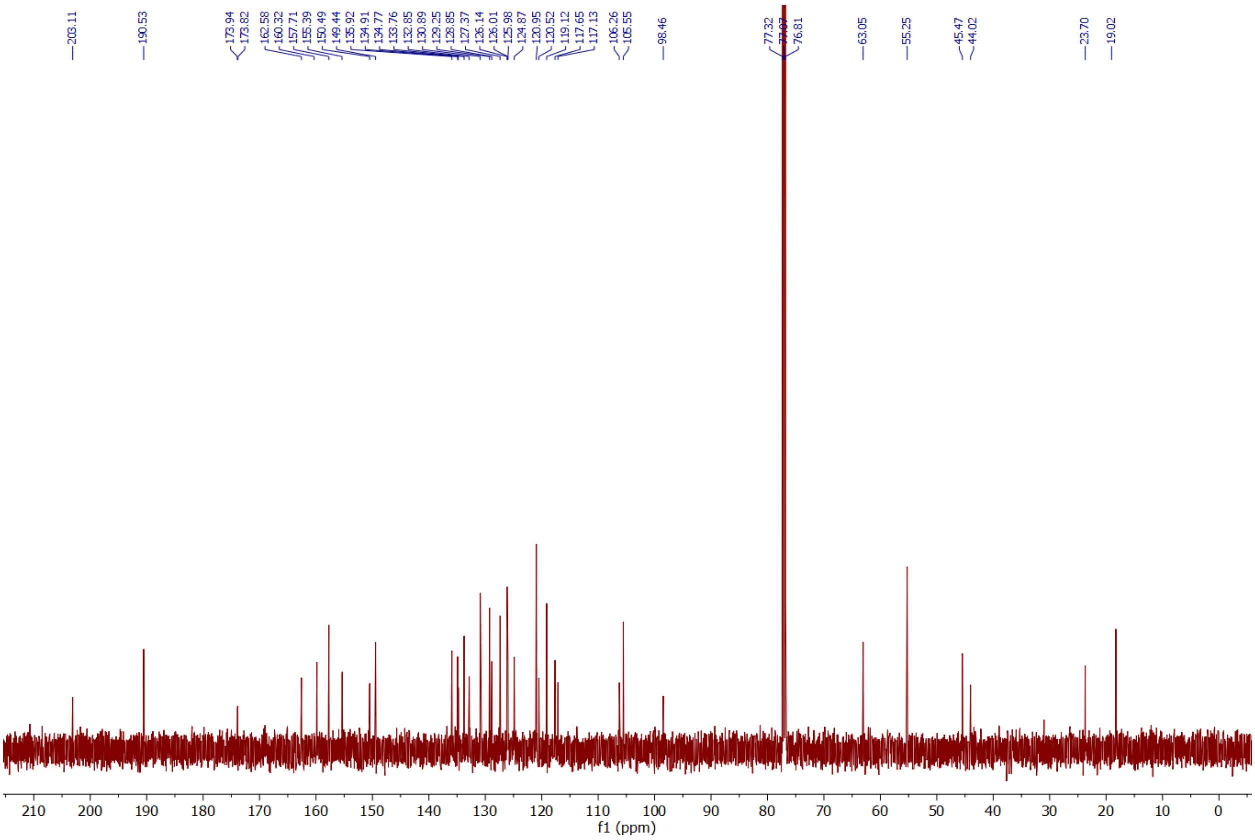

Supplementary Fig. 112. ^{13}C NMR (126 MHz, $CDCl_3$) spectrum for 4ac.

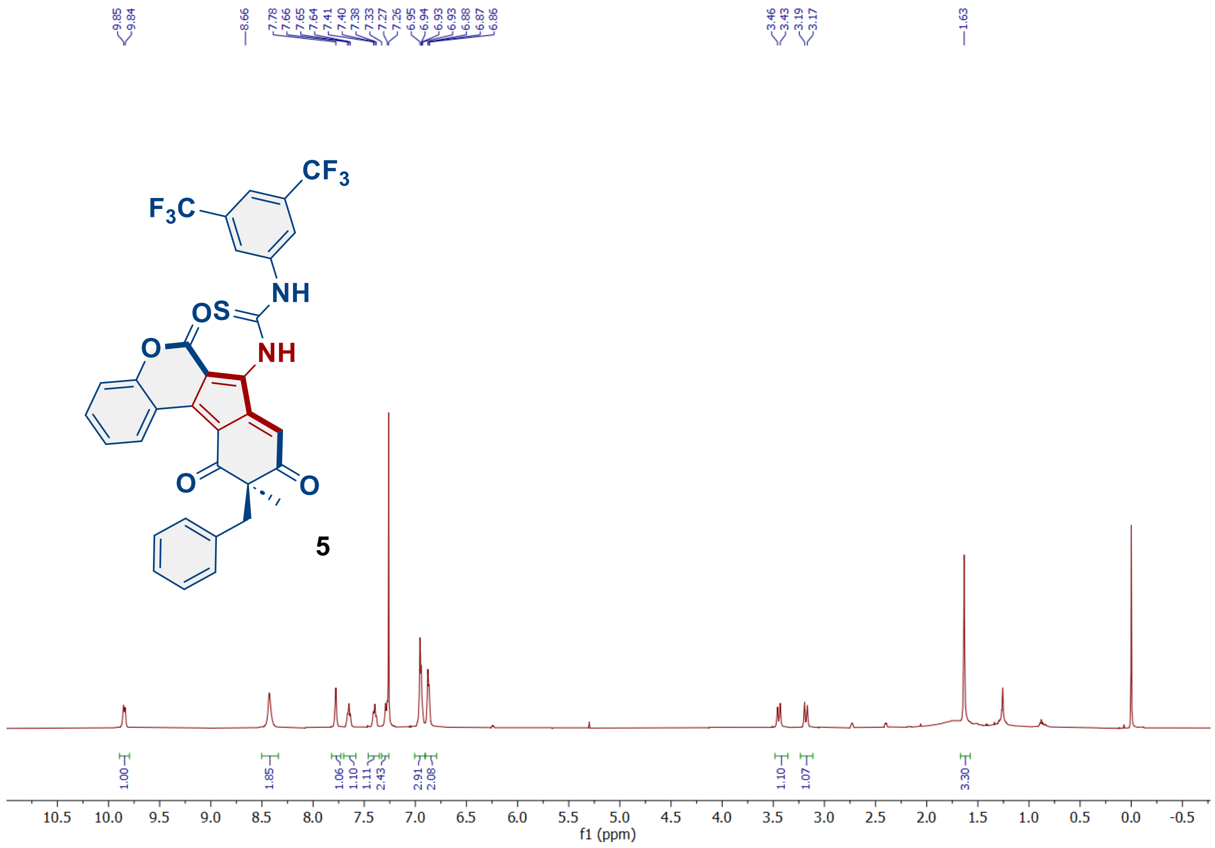

Supplementary Fig. 113. ^{1}**H NMR (500 MHz, CDCl$_3$) spectrum for 5.**

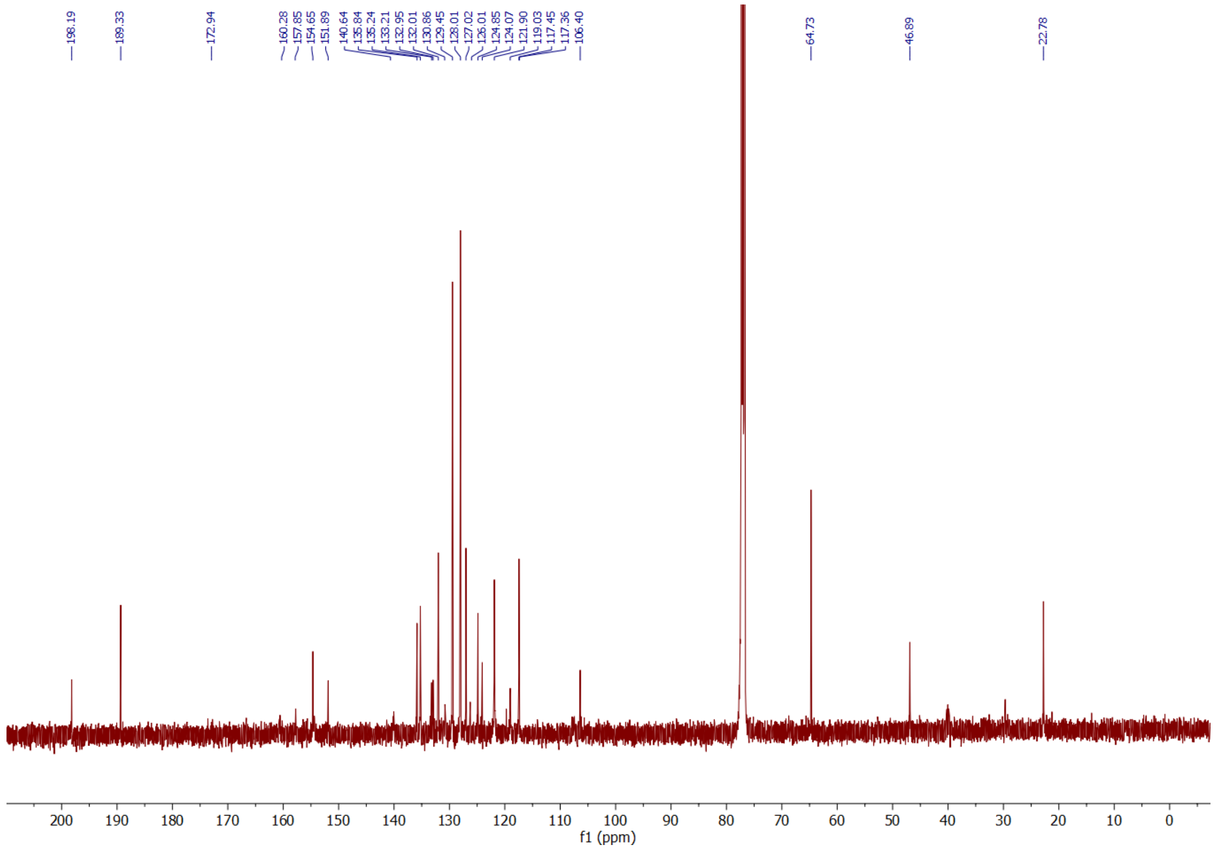

Supplementary Fig. 114. ^{13}**C NMR (126 MHz, CDCl$_3$) spectrum for 5.**

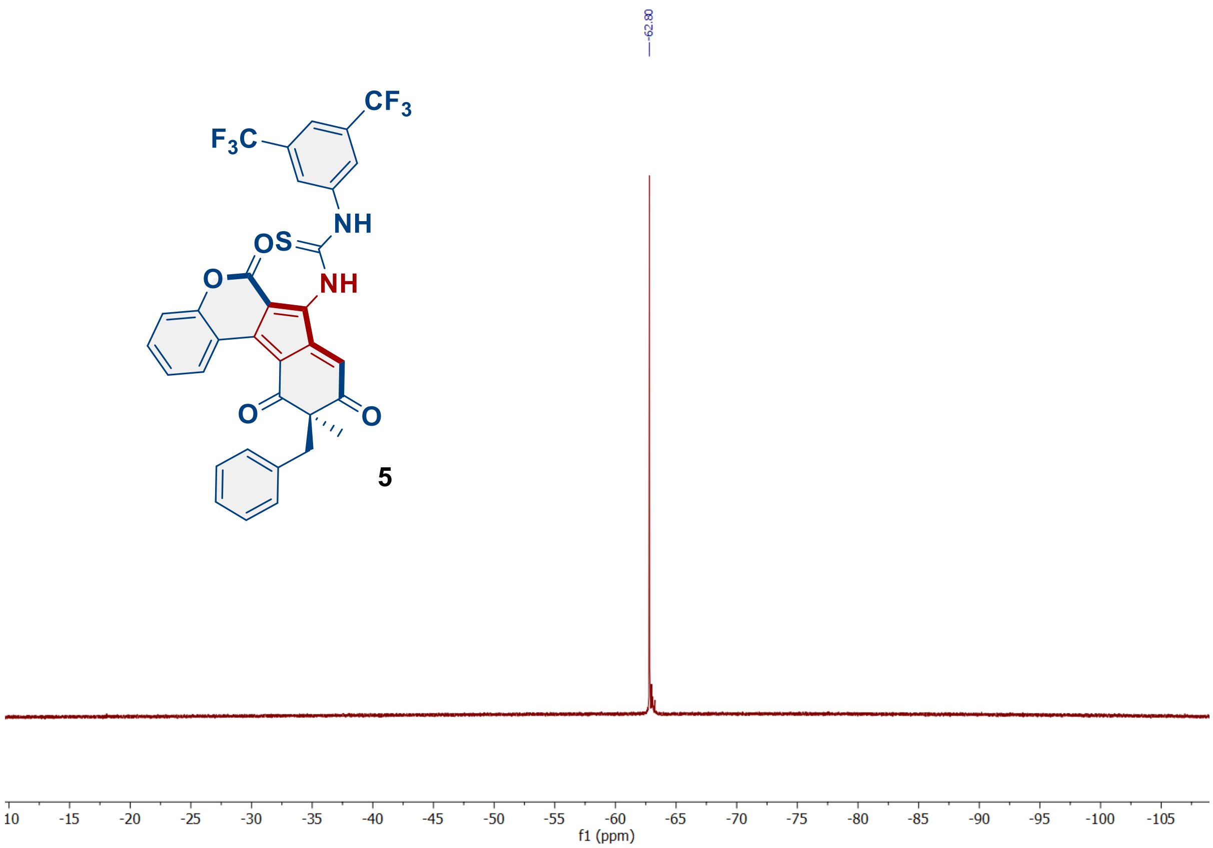

Supplementary Fig. 115. ^{19}**F NMR (471 MHz, CDCl$_3$) spectrum for 5.**

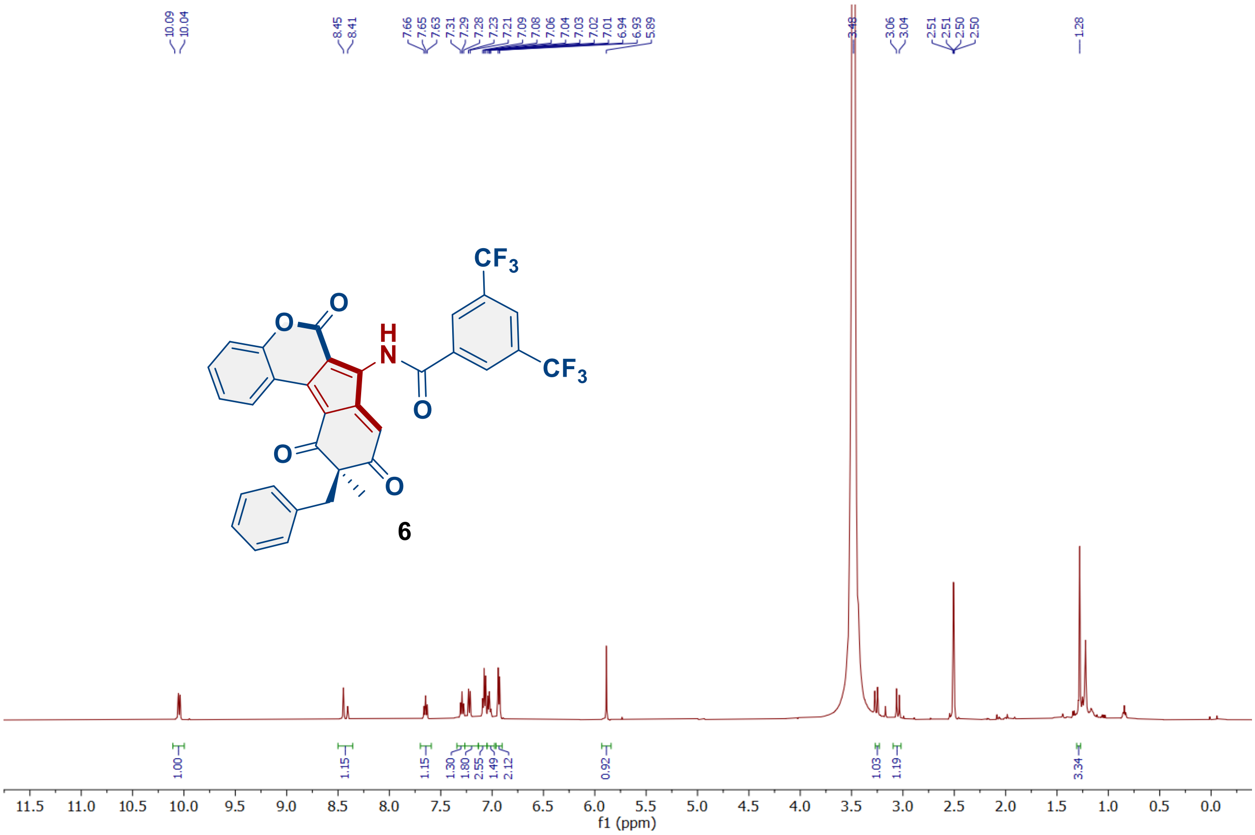

Supplementary Fig. 116. **^{1}H NMR (500 MHz, DMSO-*d*$_6$) spectrum for 6.**

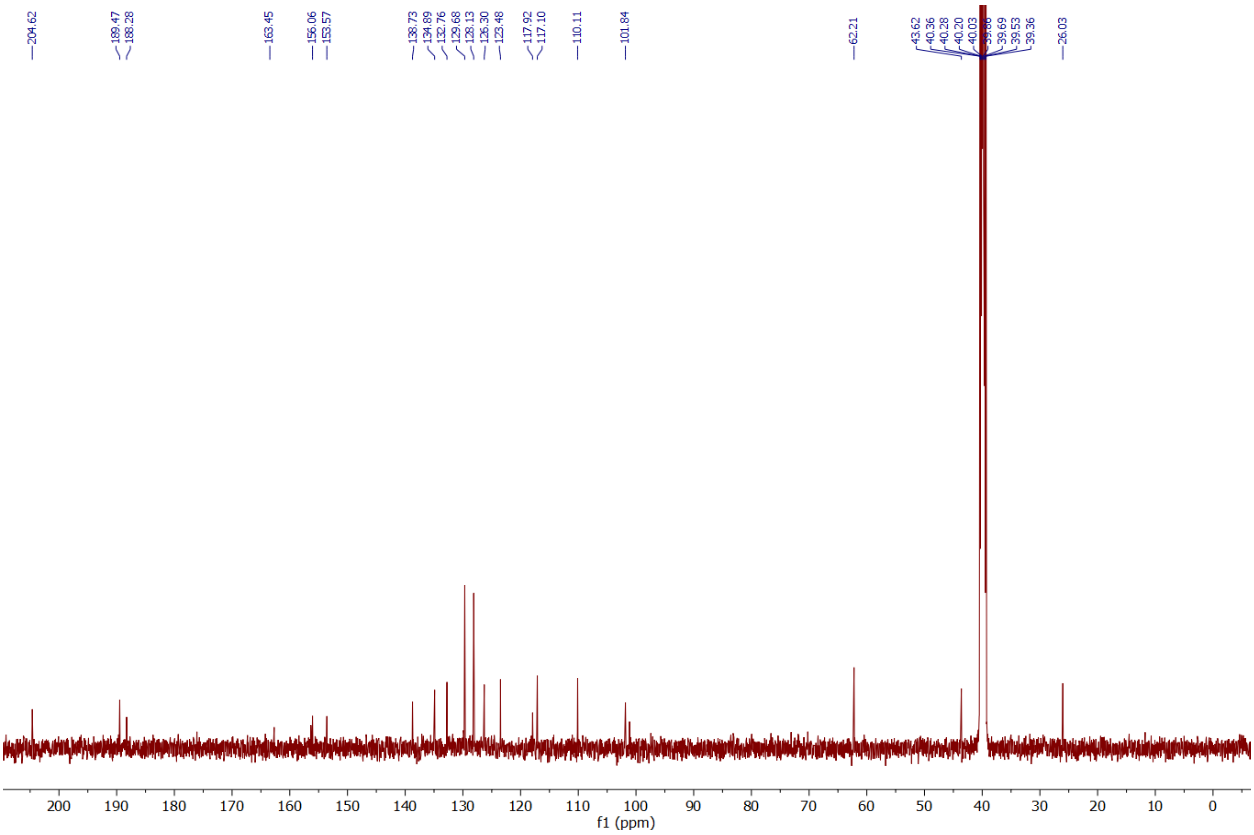

Supplementary Fig. 117. **^{13}C NMR (126 MHz, DMSO-*d*$_6$) spectrum for 6.**

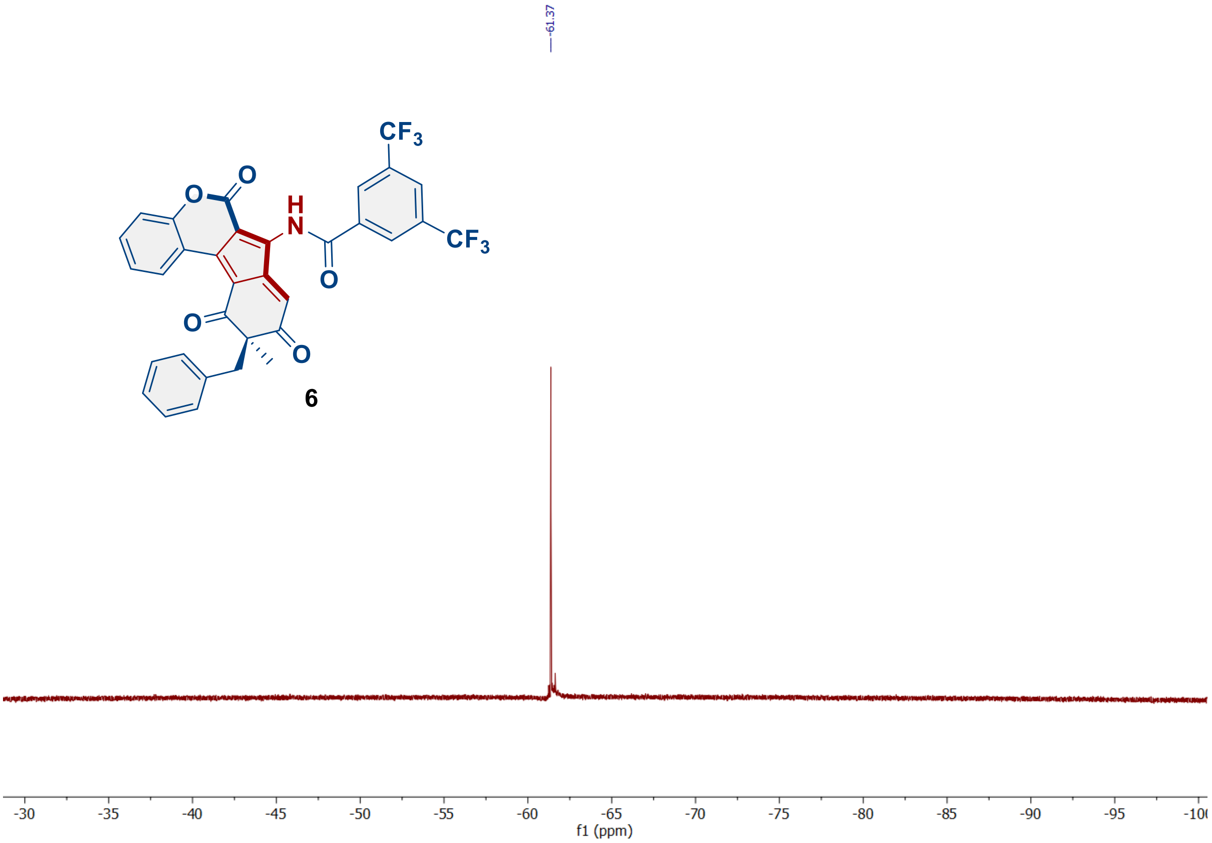

Supplementary Fig. 118. ^{13}C NMR (471 MHz, DMSO-d_6) spectrum for 6.

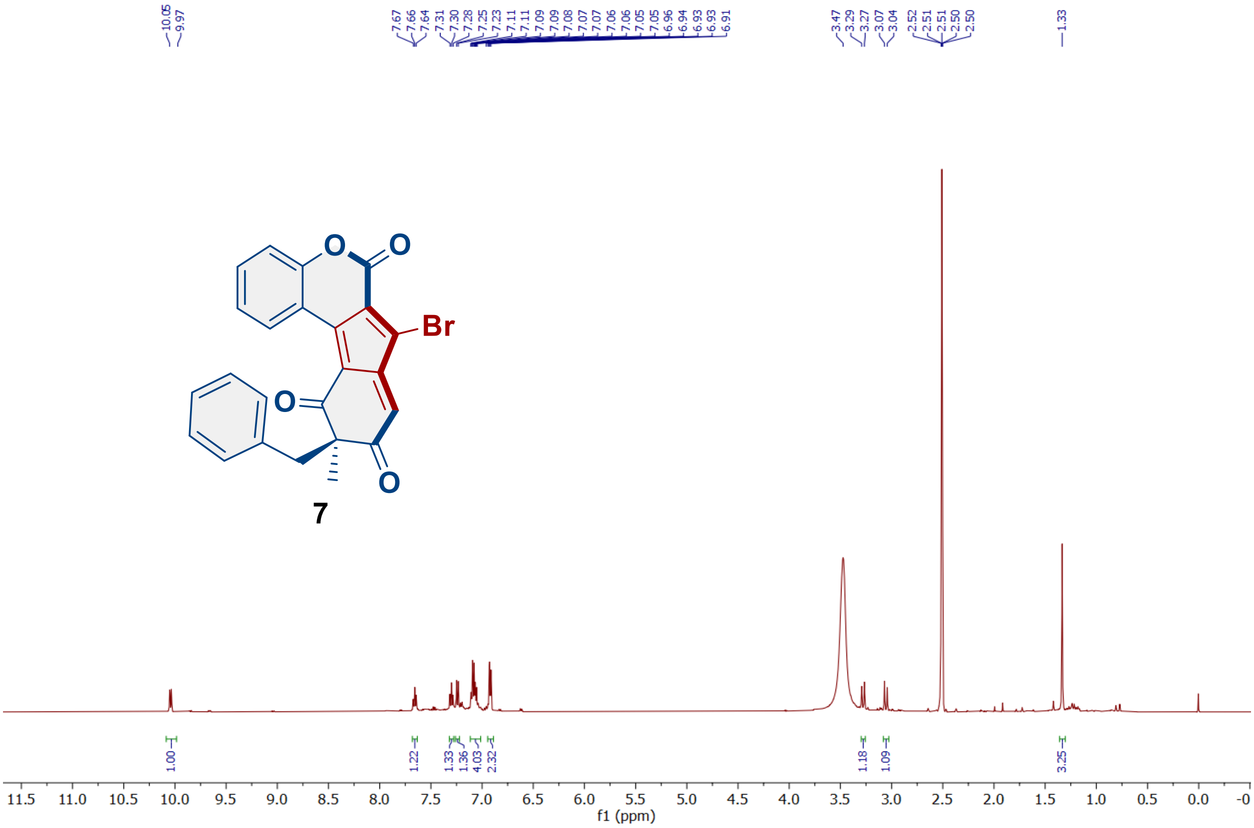

Supplementary Fig. 119. ^{1}**H NMR (500 MHz, DMSO-d_6) spectrum for 7.**

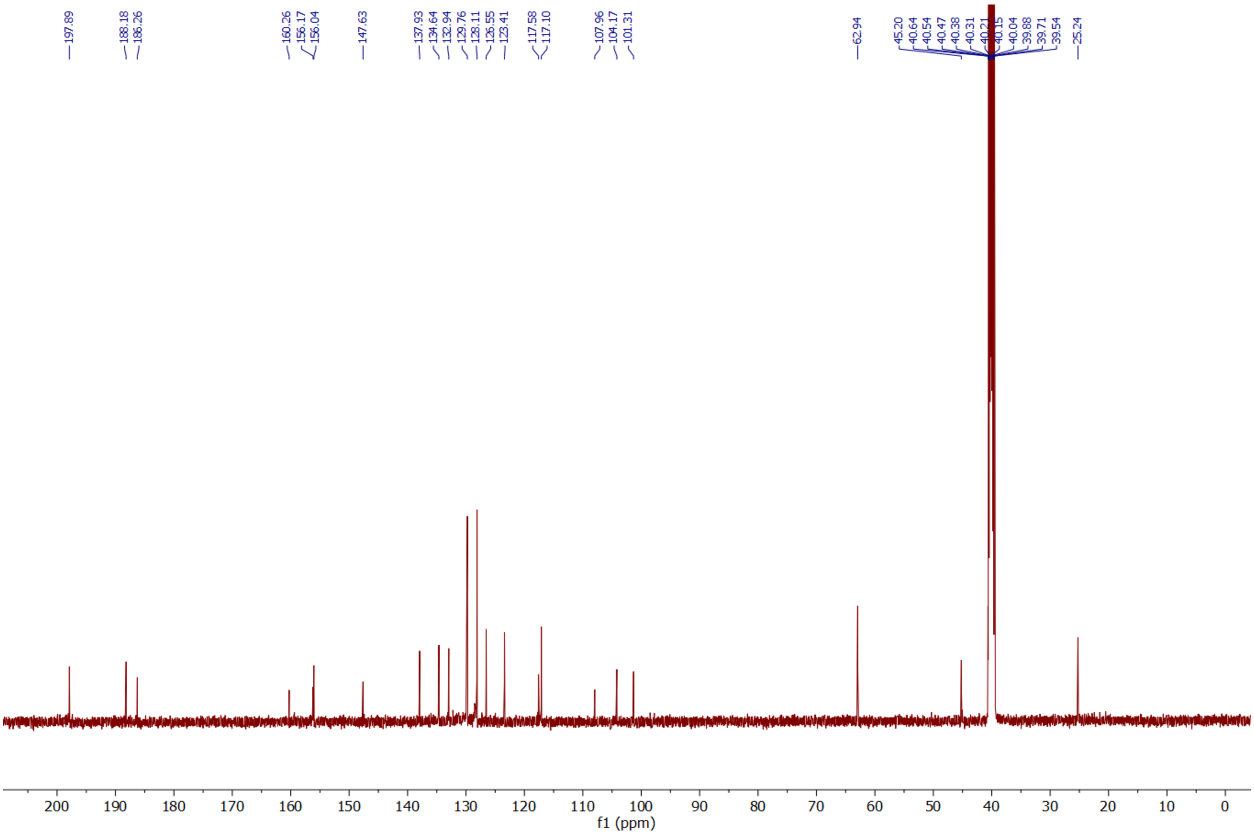

Supplementary Fig. 120. ^{13}**C NMR (126 MHz, DMSO-d_6) spectrum for 7.**

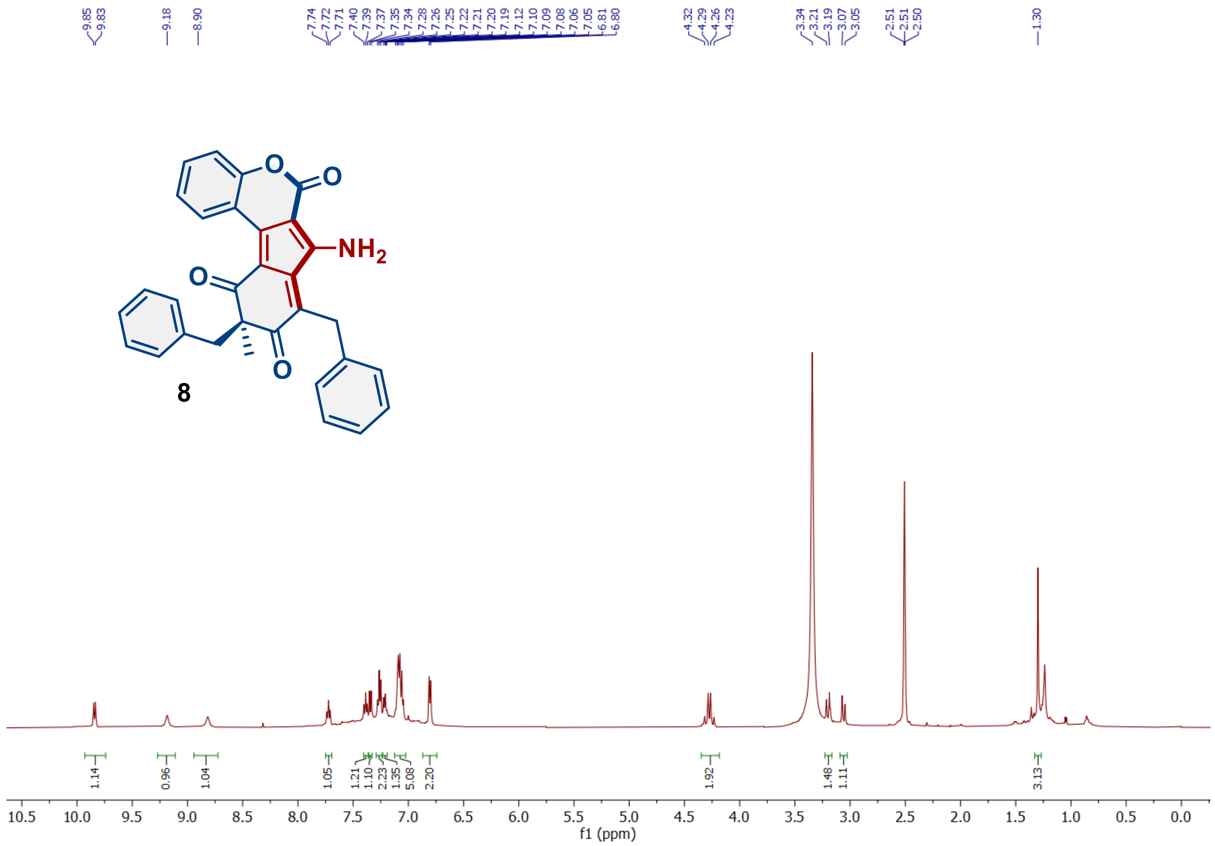

Supplementary Fig. 121. ^{1}**H NMR (500 MHz, DMSO-*d*$_6$) spectrum for 8.**

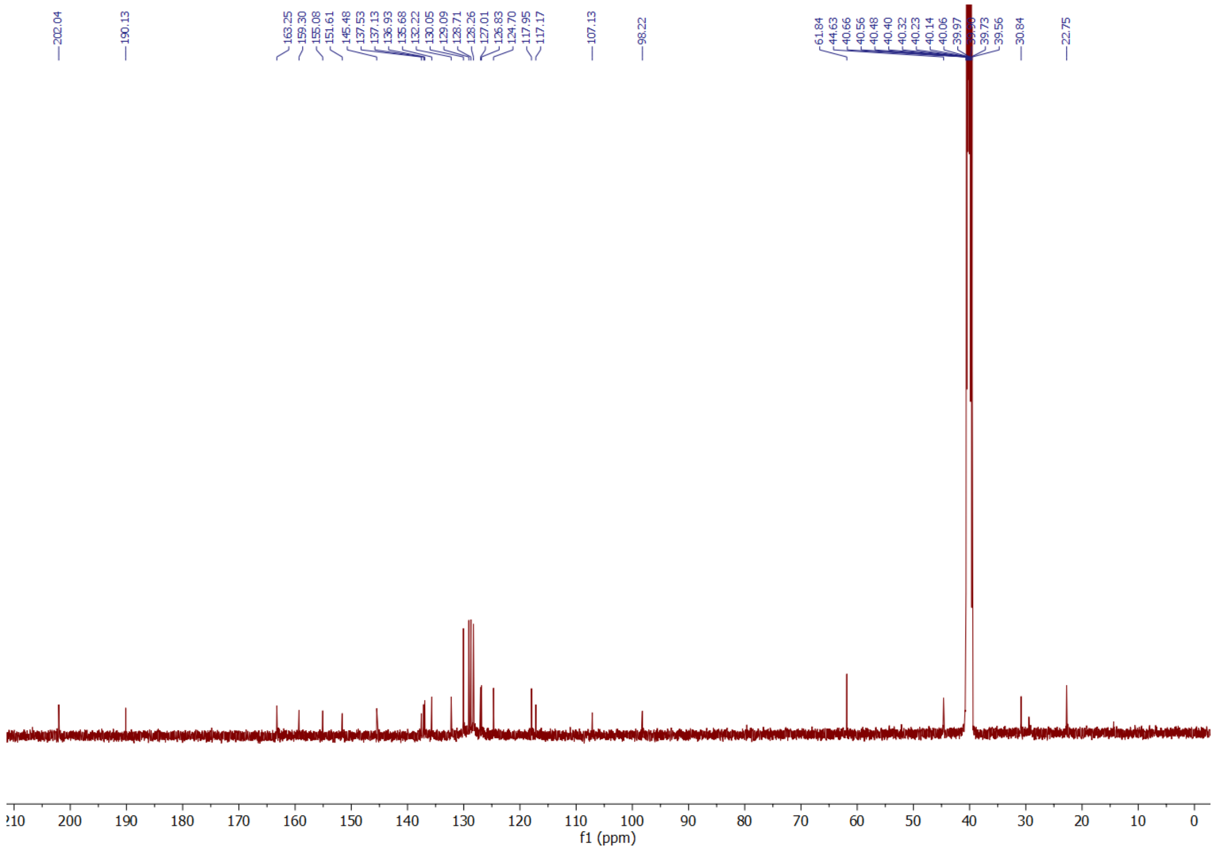

Supplementary Fig. 122. ^{13}**C NMR (126 MHz, DMSO-*d*$_6$) spectrum for 8.**

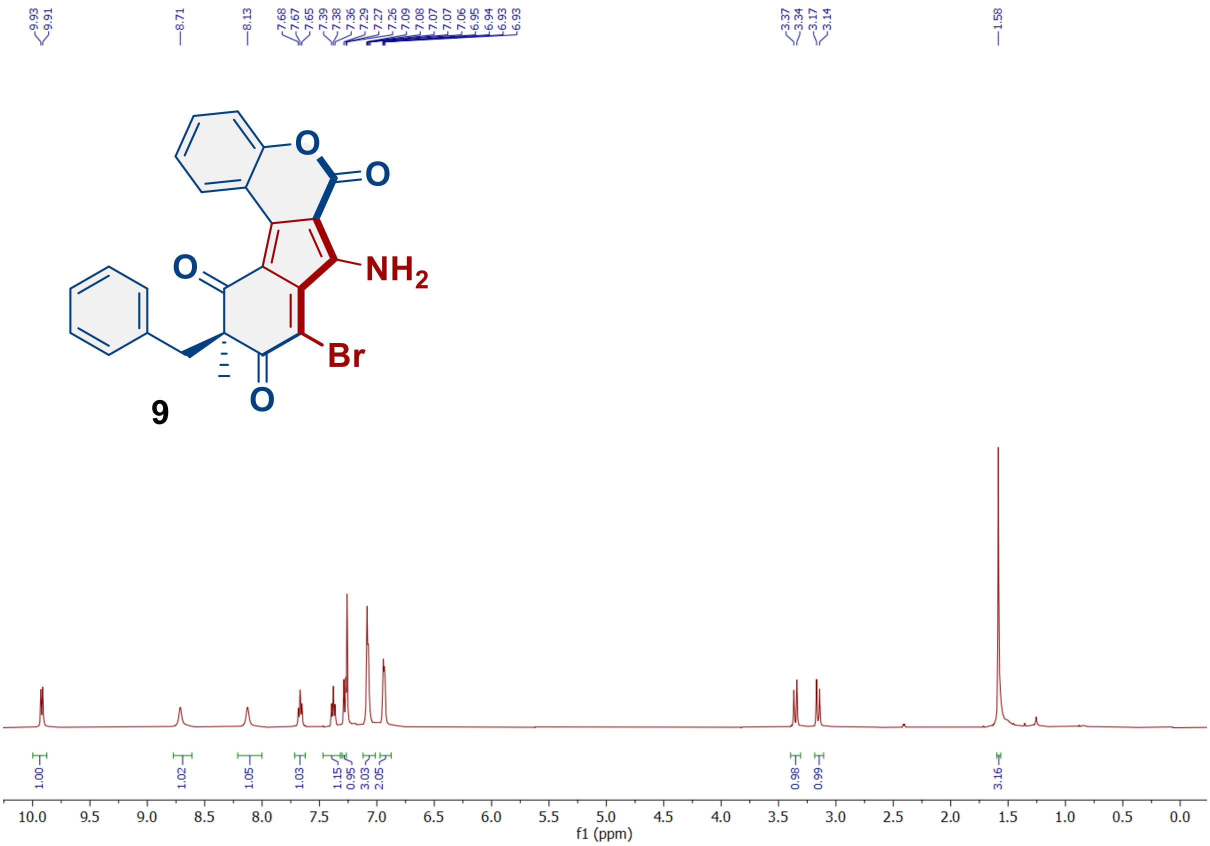

Supplementary Fig. 123. **^{1}H NMR (500 MHz, $CDCl_3$) spectrum for 9.**

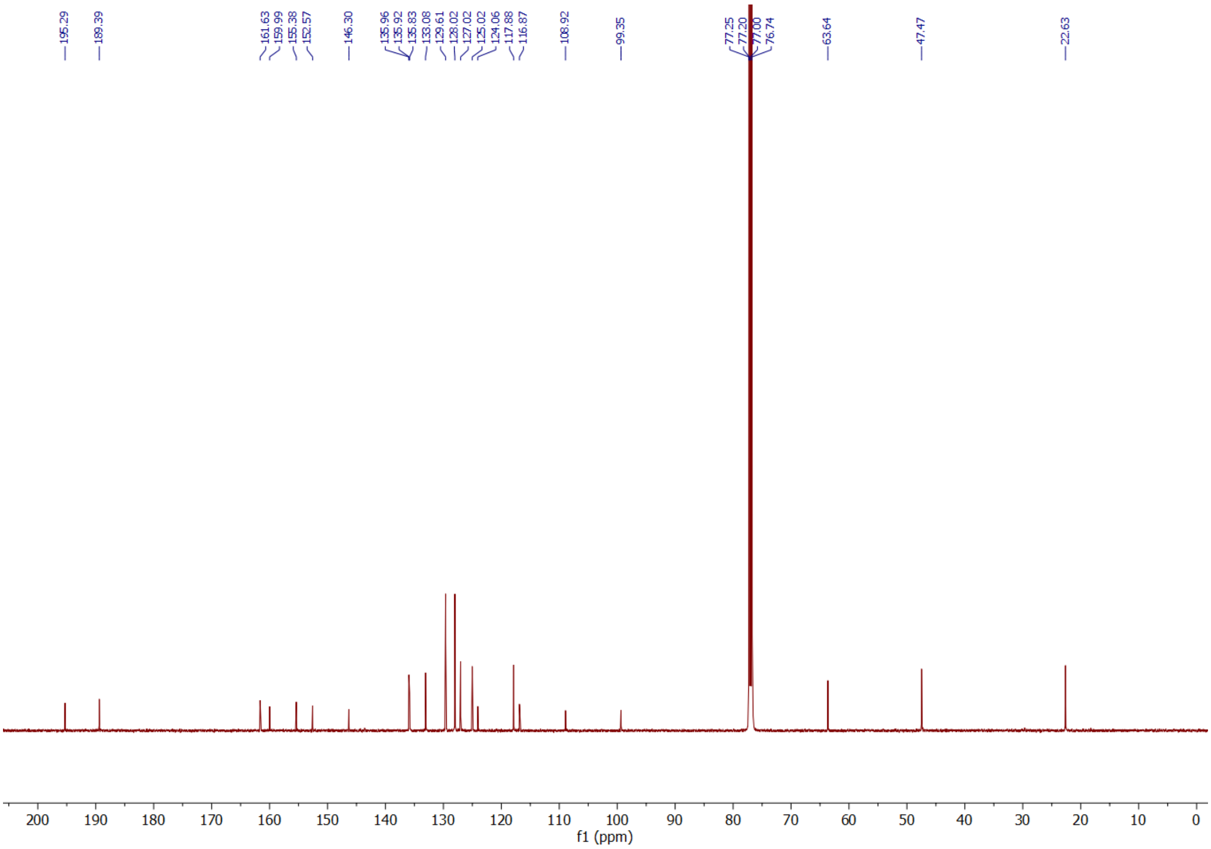

Supplementary Fig. 124. **^{13}C NMR (126 MHz, DMSO-d_6) spectrum for 9.**

8. HPLC Chromatograms

Supplementary Fig. 125. HPLC traces of rac-**3a** (top) and chiral-**3a** (bottom).

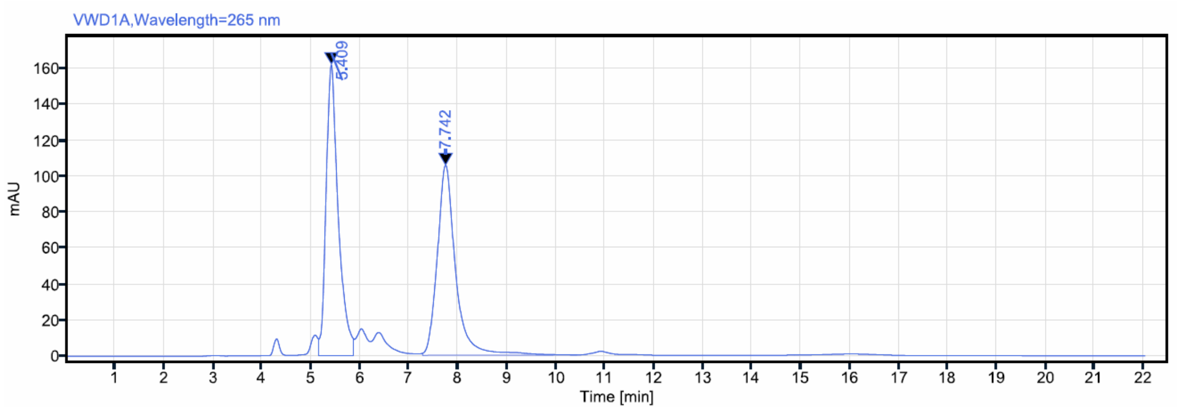

Signal: VWD1A,Wavelength=265 nm

RT [min]	Width [min]	Area	Height	Area%
5.409	0.70	2784.10	161.77	50.37
7.742	3.15	2743.60	105.58	49.63
	Sum	5527.70		

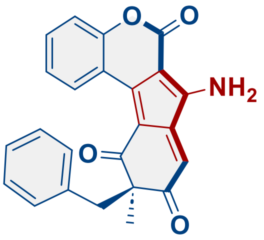

3a

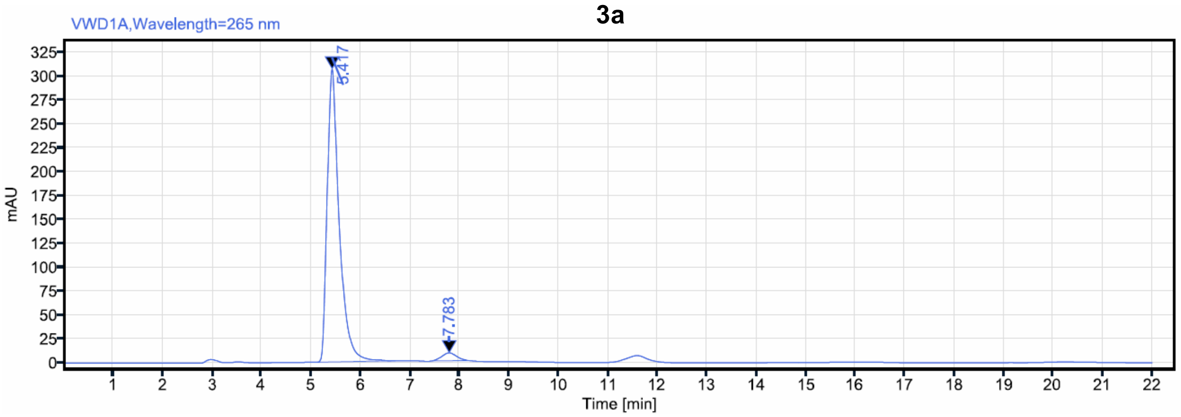

Signal: VWD1A,Wavelength=265 nm

RT [min]	Width [min]	Area	Height	Area%
5.417	1.57	5181.89	305.71	96.76
7.783	0.75	173.42	8.29	3.24
	Sum	5355.30		

Supplementary Fig. 126. HPLC traces of rac-**3b** (top) and chiral-**3b** (bottom).

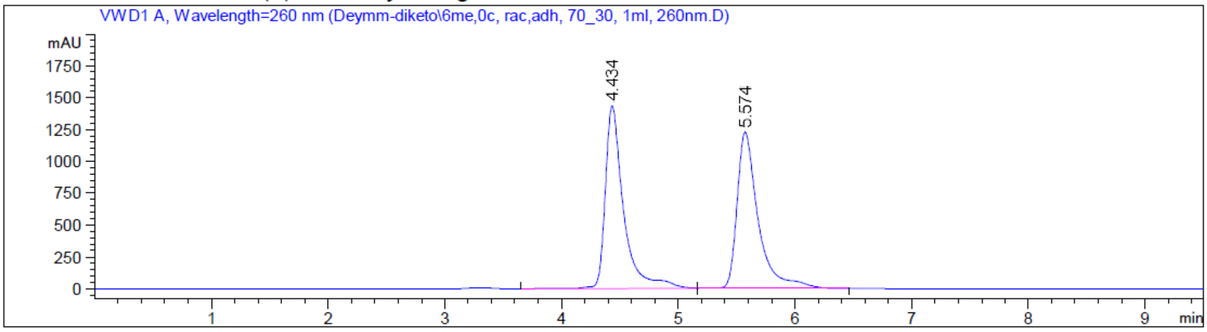

Signal 1: VWD1 A, Wavelength=260 nm

Peak #	RetTime [min]	Type	Width [min]	Area [mAU*s]	Height [mAU]	Area %
1	4.434	VV R	0.1582	1.55482e4	1432.84290	50.2732
2	5.574	VB R	0.1844	1.53793e4	1223.82959	49.7268

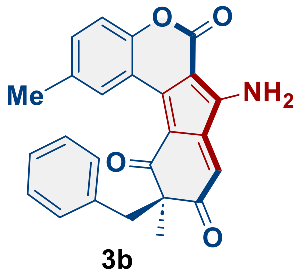

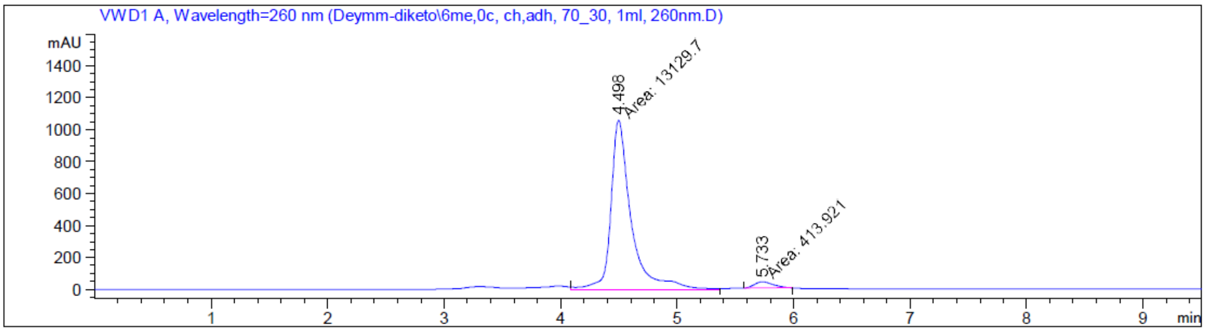

Signal 1: VWD1 A, Wavelength=260 nm

Peak #	RetTime [min]	Type	Width [min]	Area [mAU*s]	Height [mAU]	Area %
1	4.498	FM	0.2060	1.31297e4	1062.11890	96.9438
2	5.733	MM	0.1823	413.92114	37.84959	3.0562

Supplementary Fig. 127. HPLC traces of rac-**3c** (top) and chiral-**3c** (bottom)**.**

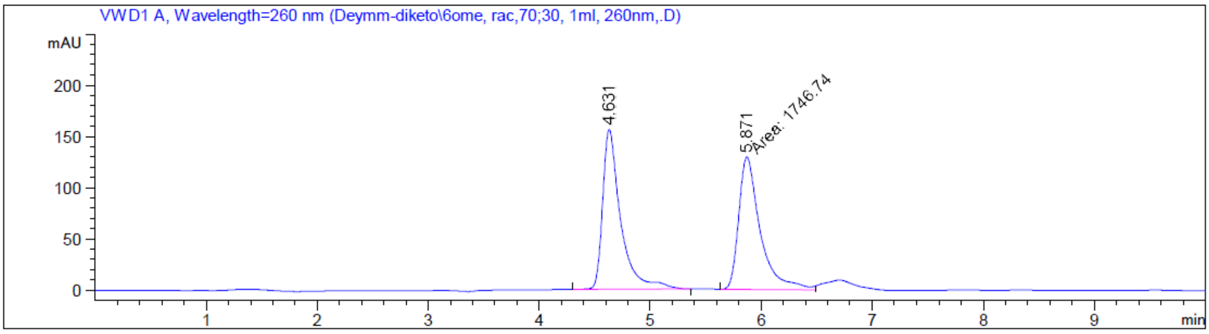

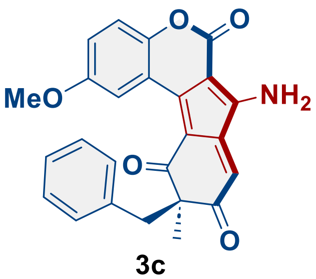

Signal 1: VWD1 A, Wavelength=260 nm

Peak #	RetTime [min]	Type	Width [min]	Area [mAU*s]	Height [mAU]	Area %
1	4.631	BV R	0.1628	1766.96826	156.01549	50.2879
2	5.871	MF	0.2253	1746.73608	129.24249	49.7121

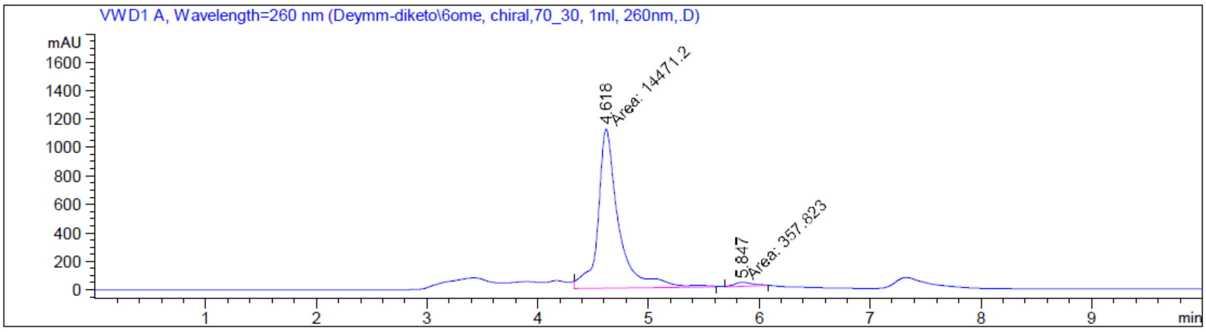

Signal 1: VWD1 A, Wavelength=260 nm

Peak #	RetTime [min]	Type	Width [min]	Area [mAU*s]	Height [mAU]	Area %
1	4.618	FM	0.2161	1.44712e4	1116.04175	97.5870
2	5.847	MM	0.2044	357.82281	29.17650	2.4130

Supplementary Fig. 128. HPLC traces of rac-**3d** (top) and chiral-**3d** (bottom).

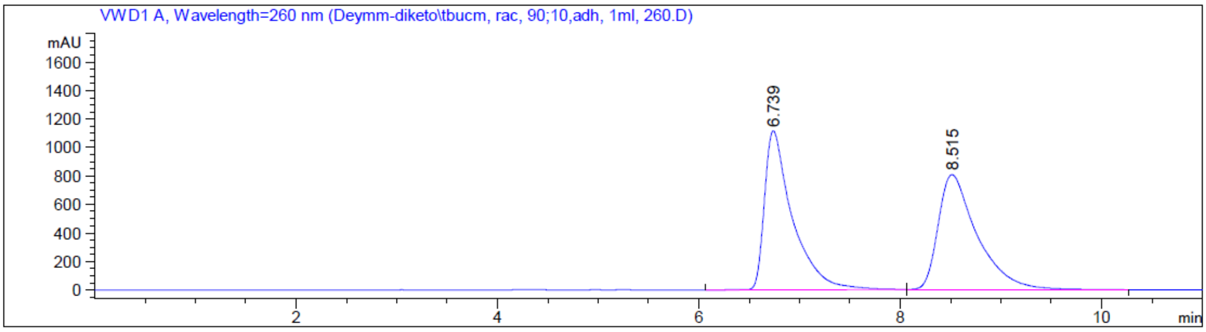

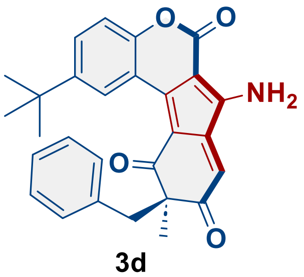

Signal 1: VWD1 A, Wavelength=260 nm

Peak #	RetTime [min]	Type	Width [min]	Area [mAU*s]	Height [mAU]	Area %
1	6.739	VV R	0.2775	2.16607e4	1115.55017	49.9919
2	8.515	VB	0.3929	2.16677e4	807.86853	50.0081

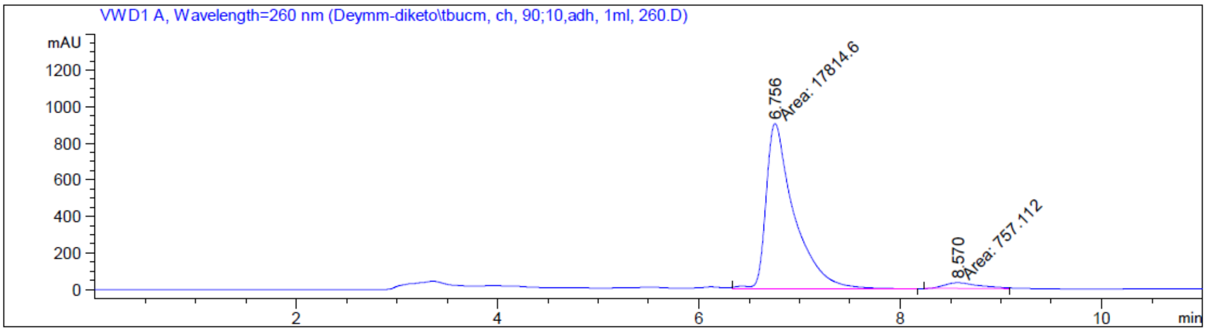

Signal 1: VWD1 A, Wavelength=260 nm

Peak #	RetTime [min]	Type	Width [min]	Area [mAU*s]	Height [mAU]	Area %
1	6.756	FM	0.3278	1.78146e4	905.86346	95.9233
2	8.570	MM	0.4095	757.11169	30.81491	4.0767

Supplementary Fig. 129. HPLC traces of rac-**3e** (top) and chiral-**3e** (bottom)**.**

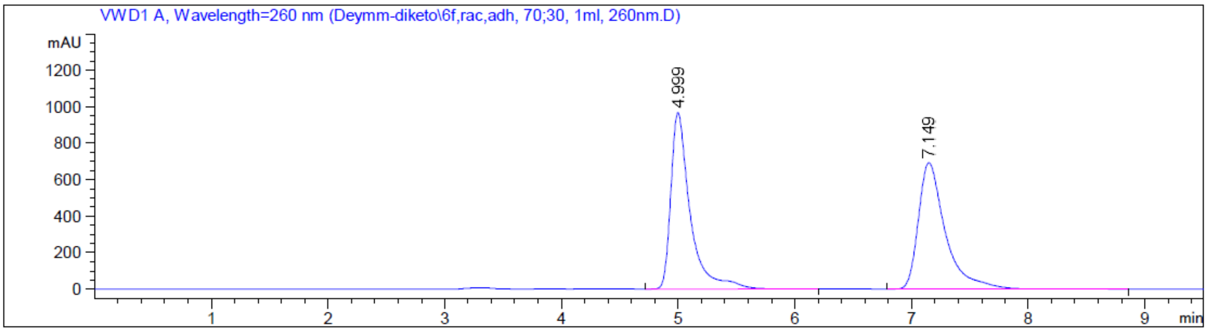

Signal 1: VWD1 A, Wavelength=260 nm

Peak #	RetTime [min]	Type	Width [min]	Area [mAU*s]	Height [mAU]	Area %
1	4.999	BB	0.1678	1.11717e4	967.07483	50.1072
2	7.149	BV R	0.2384	1.11239e4	692.37201	49.8928

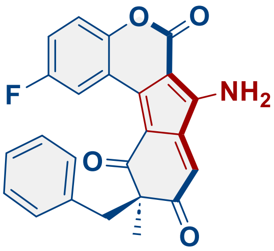

3e

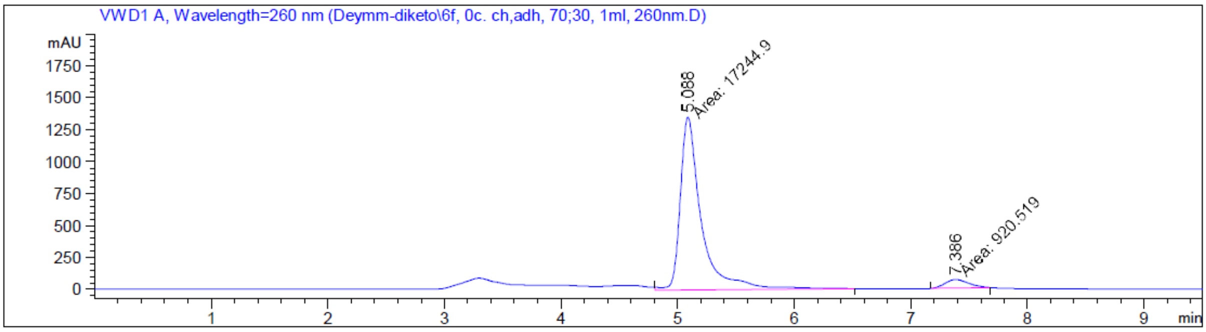

Signal 1: VWD1 A, Wavelength=260 nm

Peak #	RetTime [min]	Type	Width [min]	Area [mAU*s]	Height [mAU]	Area %
1	5.088	MM	0.2124	1.72449e4	1353.19177	94.9326
2	7.386	MM	0.2315	920.51862	66.26250	5.0674

Supplementary Fig. 130. HPLC traces of rac-**3f** (top) and chiral-**3f** (bottom).

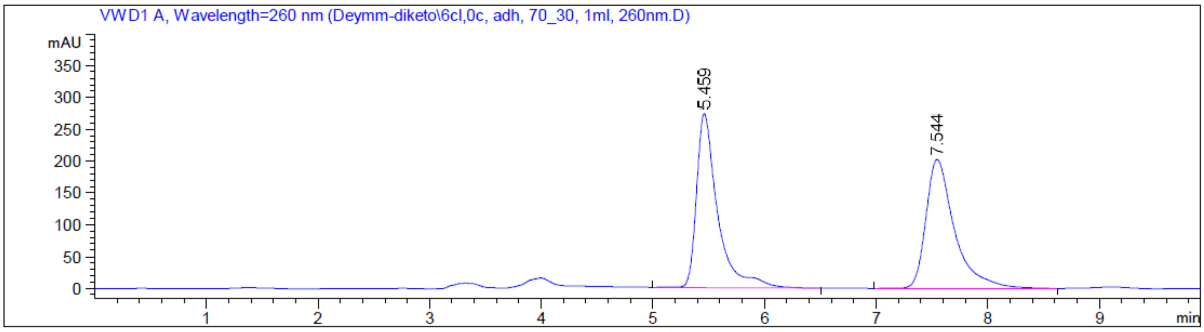

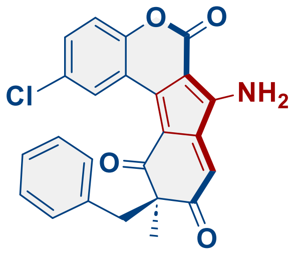

3f

Signal 1: VWD1 A, Wavelength=260 nm

Peak #	RetTime [min]	Type	Width [min]	Area [mAU*s]	Height [mAU]	Area %
1	5.459	BB	0.1918	3596.82666	272.89688	50.0071
2	7.544	BB	0.2631	3595.81177	202.60892	49.9929

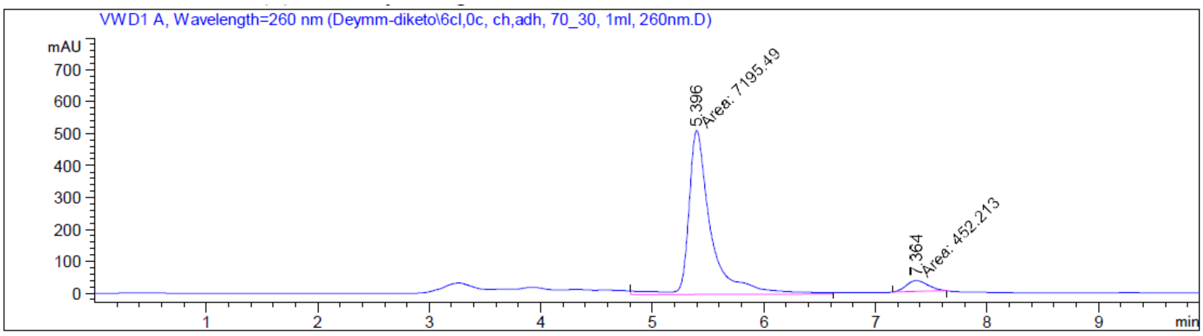

Signal 1: VWD1 A, Wavelength=260 nm

Peak #	RetTime [min]	Type	Width [min]	Area [mAU*s]	Height [mAU]	Area %
1	5.396	FM	0.2339	7195.48535	512.79413	94.0869
2	7.364	MM	0.2215	452.21280	34.03064	5.9131

Supplementary Fig. 131. HPLC traces of rac-**3g** (top) and chiral-**3g** (bottom)**.**

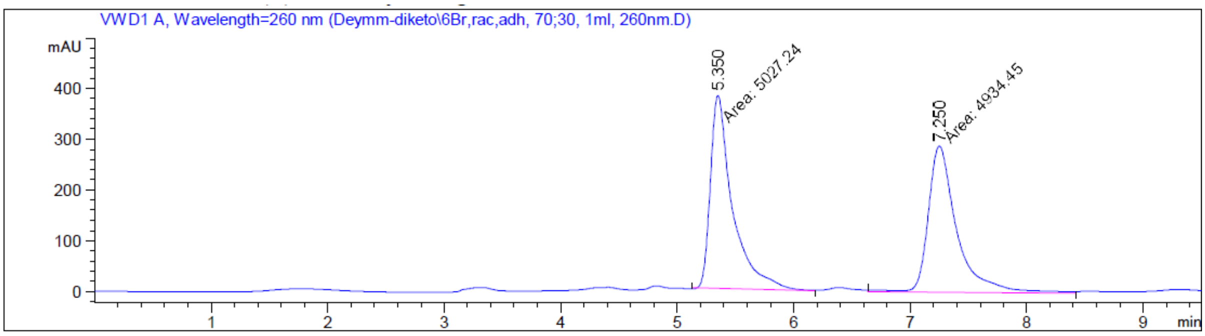

Signal 1: VWD1 A, Wavelength=260 nm

Peak #	RetTime [min]	Type	Width [min]	Area [mAU*s]	Height [mAU]	Area %
1	5.350	MM	0.2199	5027.24072	381.05576	50.4657
2	7.250	FM	0.2853	4934.45020	288.28906	49.5343

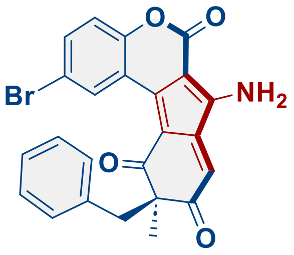

3g

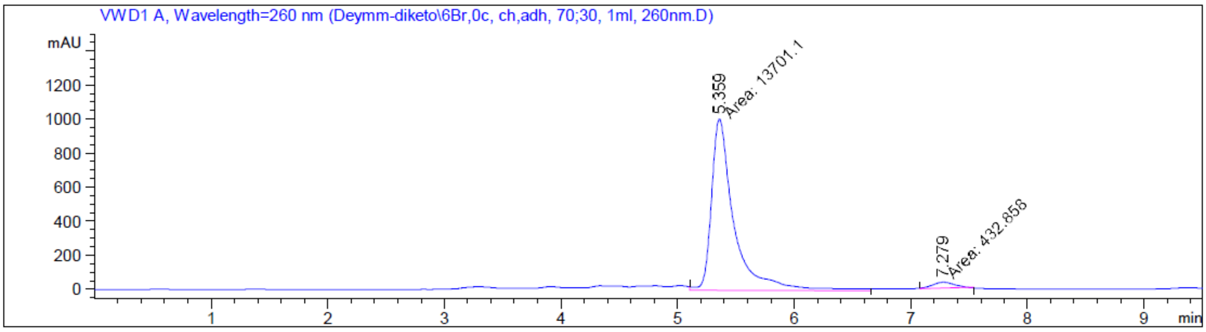

Signal 1: VWD1 A, Wavelength=260 nm

Peak #	RetTime [min]	Type	Width [min]	Area [mAU*s]	Height [mAU]	Area %
1	5.359	MF	0.2271	1.37011e4	1005.59503	96.9375
2	7.279	MF	0.2156	432.85825	33.46710	3.0625

Supplementary Fig. 132. HPLC tracec of rac-**3h** (top) and chiral-**3f** (bottom)**.**

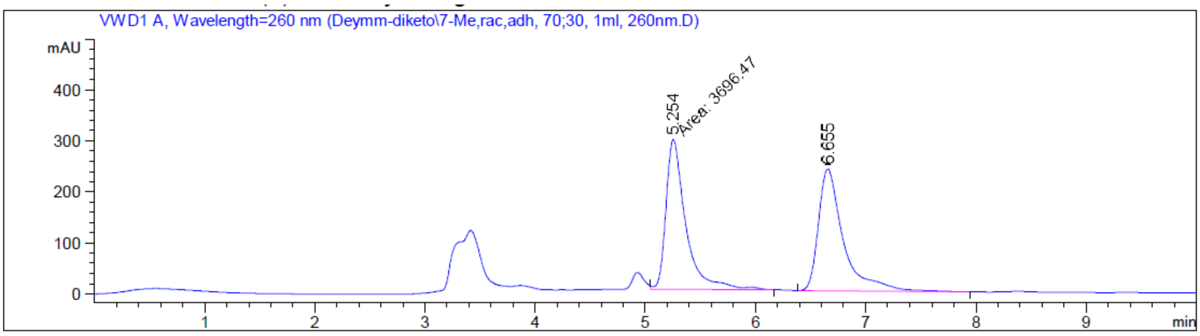

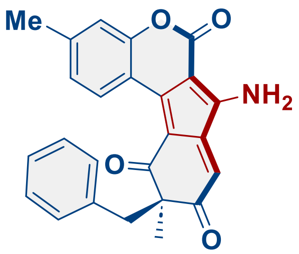

3h

Signal 1: VWD1 A, Wavelength=260 nm

Peak #	RetTime [min]	Type	Width [min]	Area [mAU*s]	Height [mAU]	Area %
1	5.254	FM	0.2096	3696.46655	293.87787	49.5122
2	6.655	BB	0.2311	3769.30640	238.84647	50.4878

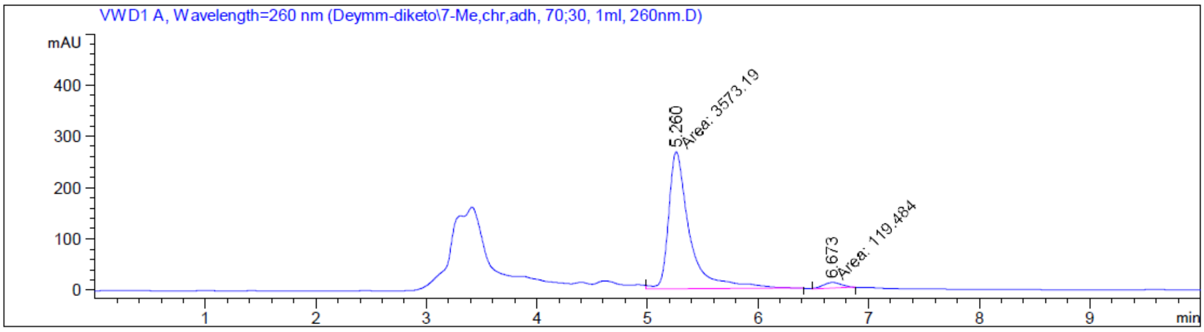

Signal 1: VWD1 A, Wavelength=260 nm

Peak #	RetTime [min]	Type	Width [min]	Area [mAU*s]	Height [mAU]	Area %
1	5.260	FM	0.2230	3573.18872	267.08124	96.7643
2	6.673	MM	0.1884	119.48445	10.57099	3.2357

Supplementary Fig. 133. HPLC tracec of rac-**3i** (top) and chiral-**3i** (bottom)**.**

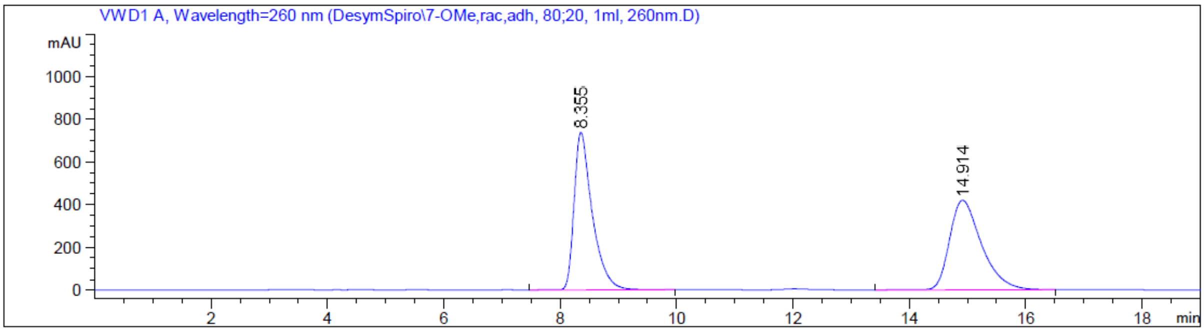

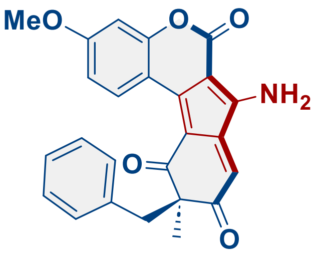

3i

Signal 1: VWD1 A, Wavelength=260 nm

Peak #	RetTime [min]	Type	Width [min]	Area [mAU*s]	Height [mAU]	Area %
1	8.355	VB R	0.3188	1.59859e4	737.73932	49.8857
2	14.914	VB R	0.5791	1.60592e4	419.30255	50.1143

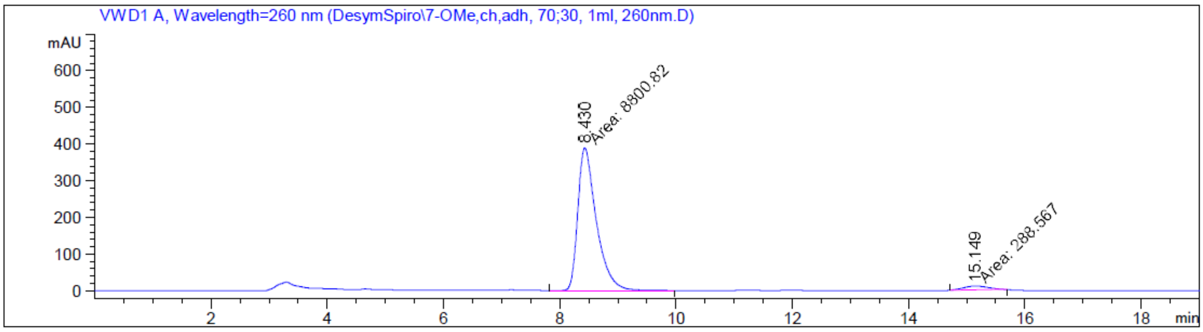

Signal 1: VWD1 A, Wavelength=260 nm

Peak #	RetTime [min]	Type	Width [min]	Area [mAU*s]	Height [mAU]	Area %
1	8.430	MM	0.3759	8800.82324	390.25433	96.8252
2	15.149	MM	0.4839	288.56738	9.93938	3.1748

Supplementary Fig. 134. HPLC tracec of rac-**3j** (top) and chiral-**3j** (bottom).

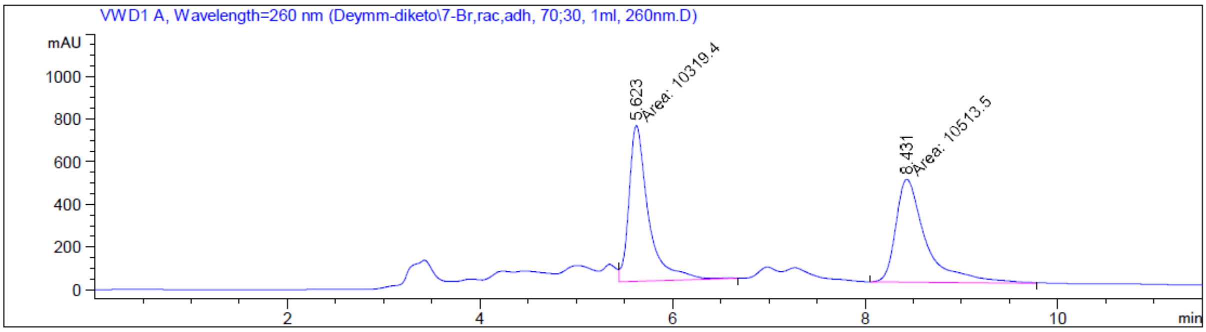

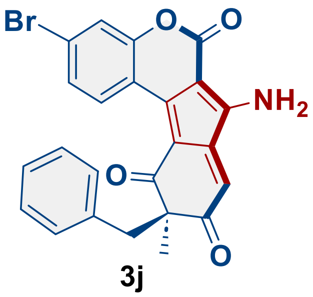

3j

Signal 1: VWD1 A, Wavelength=260 nm

Peak #	RetTime [min]	Type	Width [min]	Area [mAU*s]	Height [mAU]	Area %
1	5.623	FM	0.2352	1.03194e4	731.12000	49.5343
2	8.431	MF	0.3638	1.05135e4	481.69040	50.4657

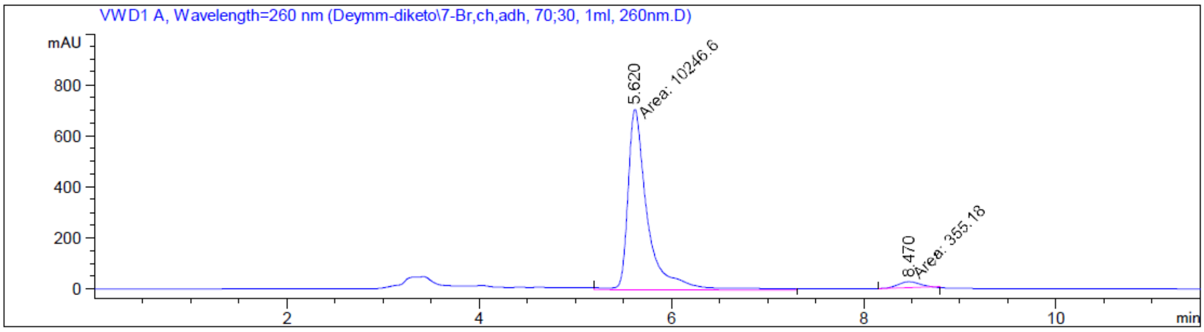

Signal 1: VWD1 A, Wavelength=260 nm

Peak #	RetTime [min]	Type	Width [min]	Area [mAU*s]	Height [mAU]	Area %
1	5.620	FM	0.2416	1.02466e4	706.89813	96.6498
2	8.470	MM	0.2568	355.18030	23.04754	3.3502

Supplementary Fig. 135. HPLC traces of rac-**3k** (top) and chiral-**3k** (bottom)**.**

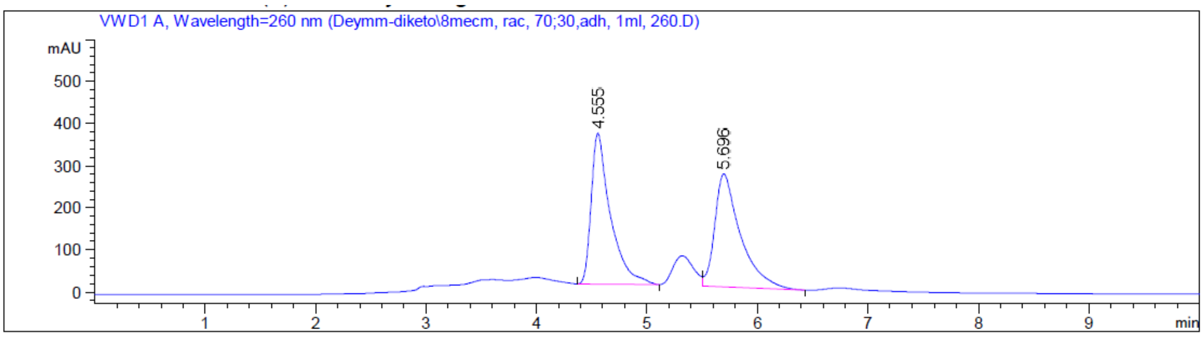

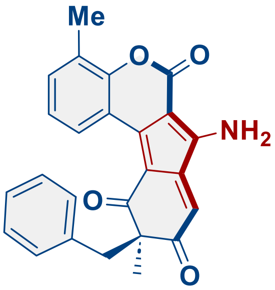

3k

Signal 1: VWD1 A, Wavelength=260 nm

Peak #	RetTime [min]	Type	Width [min]	Area [mAU*s]	Height [mAU]	Area %
1	4.555	BB	0.1732	4333.00195	358.06393	49.9104
2	5.696	VB	0.2329	4348.56250	268.61252	50.0896

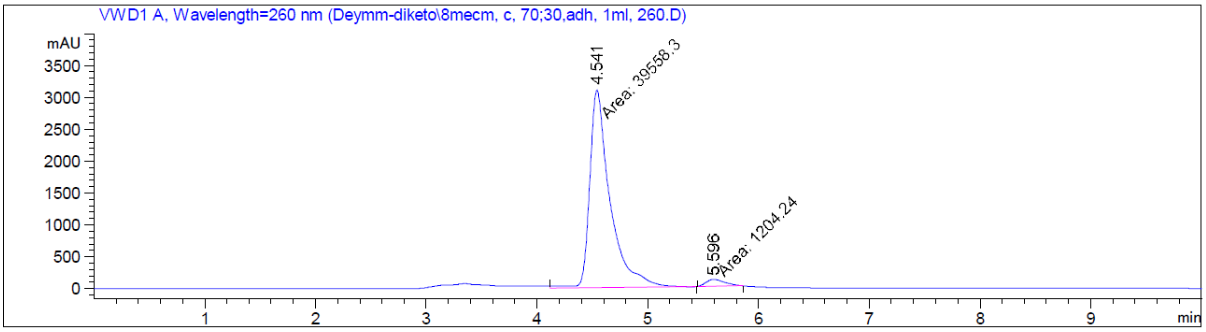

Signal 1: VWD1 A, Wavelength=260 nm

Peak #	RetTime [min]	Type	Width [min]	Area [mAU*s]	Height [mAU]	Area %
1	4.541	FM	0.2125	3.95583e4	3102.00000	97.0457
2	5.596	MM	0.1900	1204.23730	105.61787	2.9543

Supplementary Fig. 136. HPLC traces of rac-**3l** (top) and chiral-**3l** (bottom).

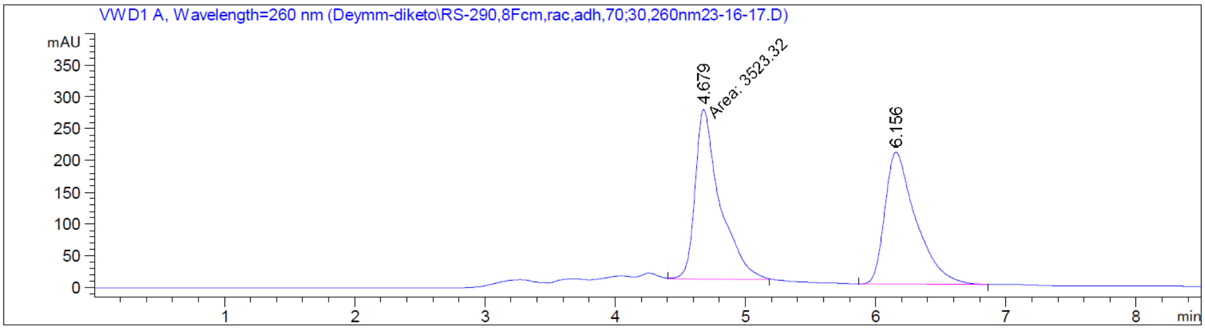

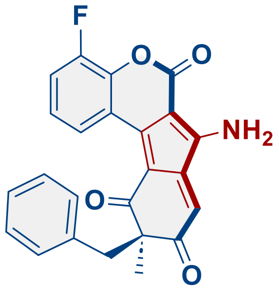

3l

Signal 1: VWD1 A, Wavelength=260 nm

Peak #	RetTime [min]	Type	Width [min]	Area [mAU*s]	Height [mAU]	Area %
1	4.679	MM	0.2204	3523.32031	266.41397	50.6406
2	6.156	BB	0.2393	3434.17578	207.31235	49.3594

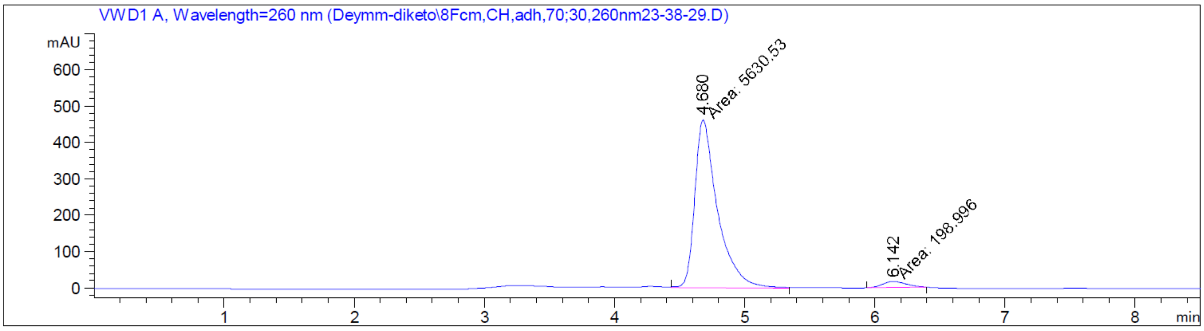

Signal 1: VWD1 A, Wavelength=260 nm

Peak #	RetTime [min]	Type	Width [min]	Area [mAU*s]	Height [mAU]	Area %
1	4.680	MM	0.2034	5630.53369	461.43768	96.5864
2	6.142	MM	0.2021	198.99634	16.41245	3.4136

Supplementary Fig. 137. HPLC traces of rac-**3m** (top) and chiral-**3m** (bottom).

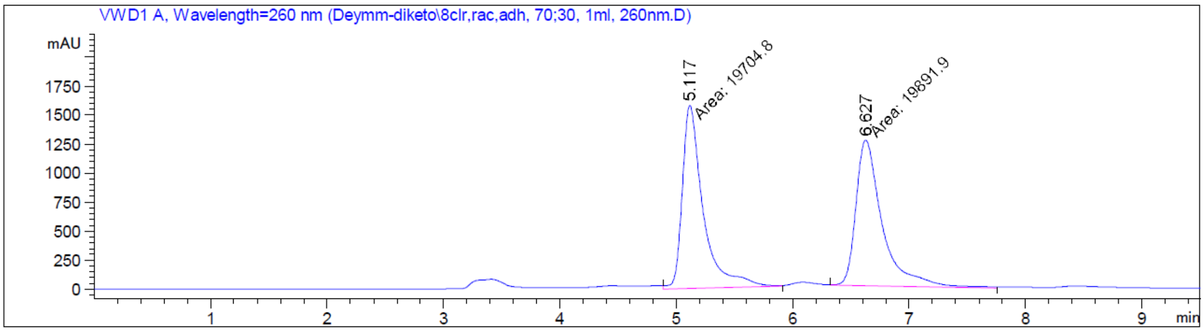

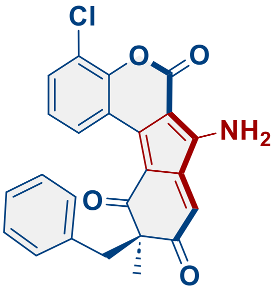

3m

Signal 1: VWD1 A, Wavelength=260 nm

Peak #	RetTime [min]	Type	Width [min]	Area [mAU*s]	Height [mAU]	Area %
1	5.117	MM	0.2081	1.97048e4	1578.00916	49.7638
2	6.627	MM	0.2636	1.98919e4	1257.50415	50.2362

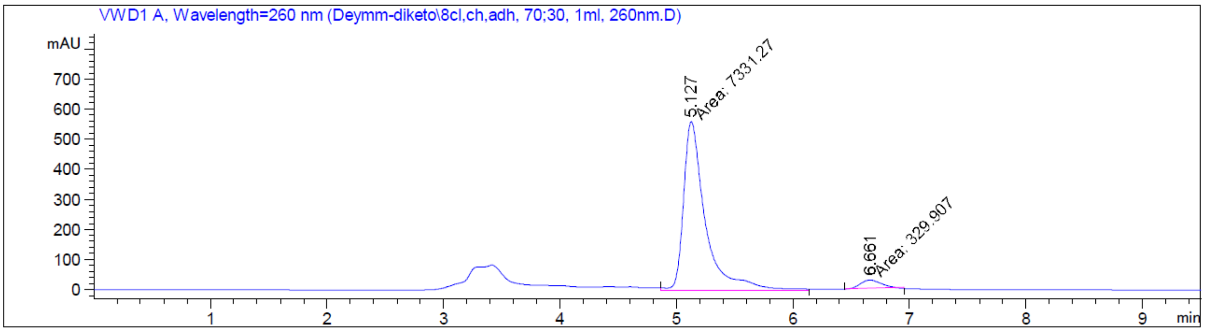

Signal 1: VWD1 A, Wavelength=260 nm

Peak #	RetTime [min]	Type	Width [min]	Area [mAU*s]	Height [mAU]	Area %
1	5.127	MM	0.2180	7331.27051	560.48816	95.6938
2	6.661	MM	0.2010	329.90659	27.34939	4.3062

Supplementary Fig. 138. HPLC traces of rac-**3n** (top) and chiral-**3n** (bottom).

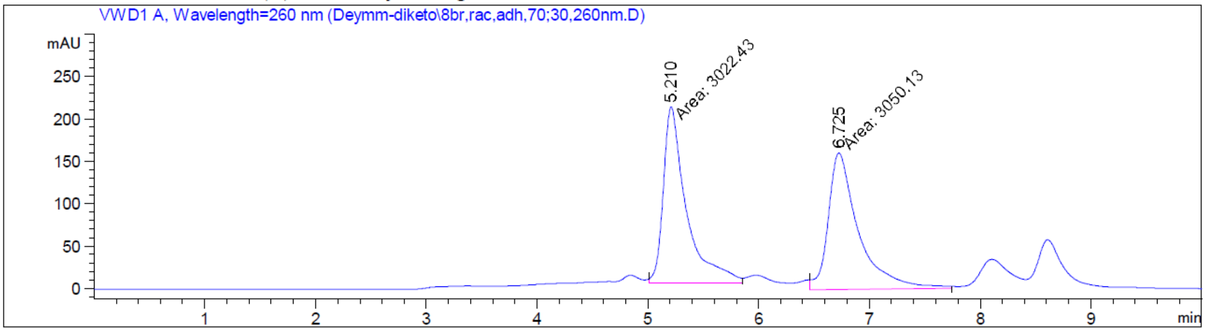

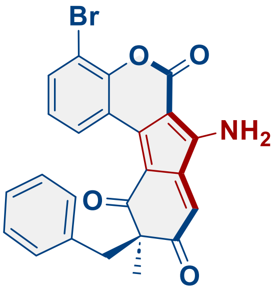

3n

Signal 1: VWD1 A, Wavelength=260 nm

Peak #	RetTime [min]	Type	Width [min]	Area [mAU*s]	Height [mAU]	Area %
1	5.210	MF	0.2426	3022.43140	207.68054	49.7720
2	6.725	MM	0.3166	3050.12817	160.56541	50.2280

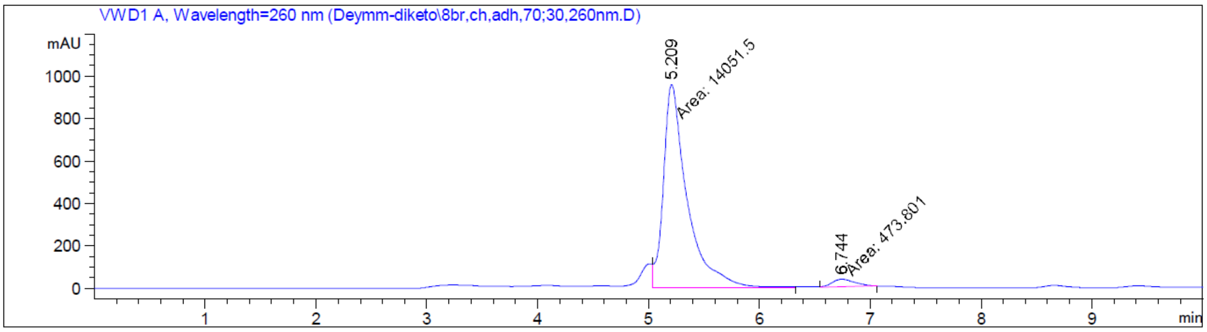

Signal 1: VWD1 A, Wavelength=260 nm

Peak #	RetTime [min]	Type	Width [min]	Area [mAU*s]	Height [mAU]	Area %
1	5.209	FM	0.2452	1.40515e4	955.11847	96.7381
2	6.744	MM	0.2283	473.80142	34.59096	3.2619

Supplementary Fig. 139. HPLC traces of rac-**3o** (top) and chiral-**3o** (bottom).

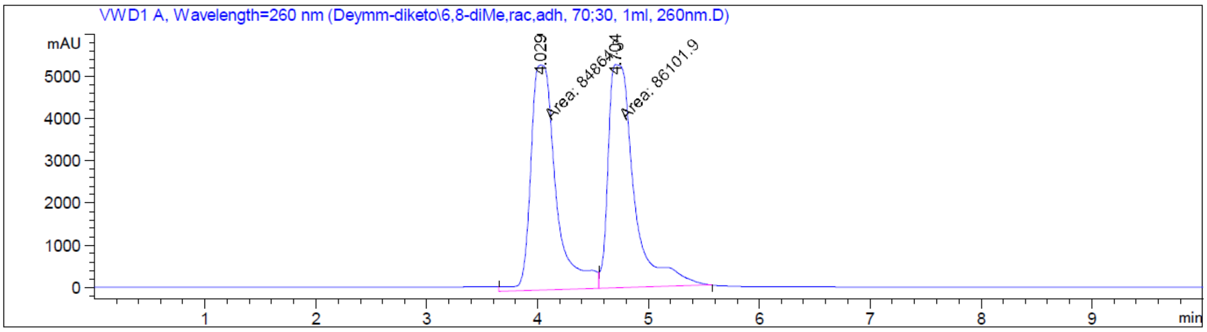

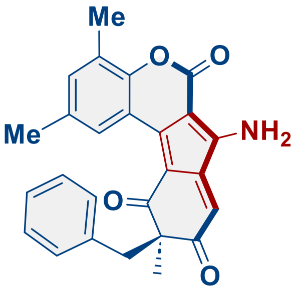

3o

Signal 1: VWD1 A, Wavelength=260 nm

Peak #	RetTime [min]	Type	Width [min]	Area [mAU*s]	Height [mAU]	Area %
1	4.029	MF	0.2658	8.48643e4	5320.76367	49.6381
2	4.704	FM	0.2717	8.61019e4	5282.07764	50.3619

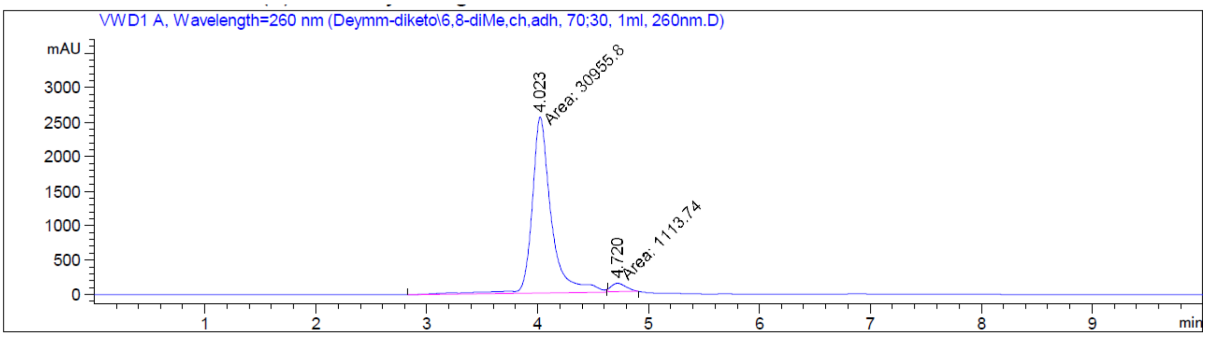

Signal 1: VWD1 A, Wavelength=260 nm

Peak #	RetTime [min]	Type	Width [min]	Area [mAU*s]	Height [mAU]	Area %
1	4.023	MM	0.2024	3.09558e4	2549.56226	96.5271
2	4.720	FM	0.1510	1113.74023	122.91880	3.4729

Supplementary Fig. 140. HPLC traces of rac-**3p** (top) and chiral-**3p** (bottom).

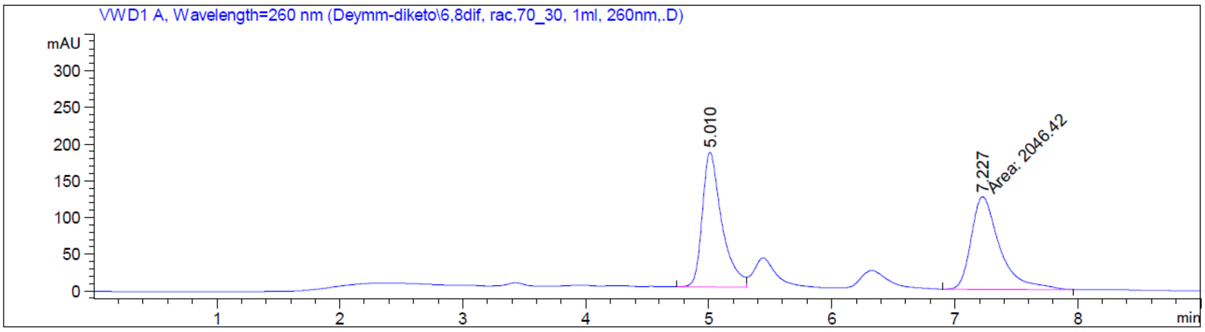

Signal 1: VWD1 A, Wavelength=260 nm

Peak #	RetTime [min]	Type	Width [min]	Area [mAU*s]	Height [mAU]	Area %
1	5.010	BV	0.1633	2033.34546	183.49777	49.8398
2	7.227	MF	0.2706	2046.41846	126.06322	50.1602

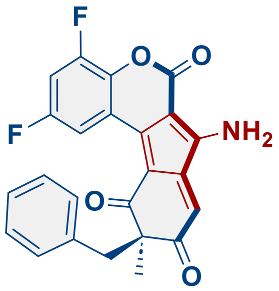

3p

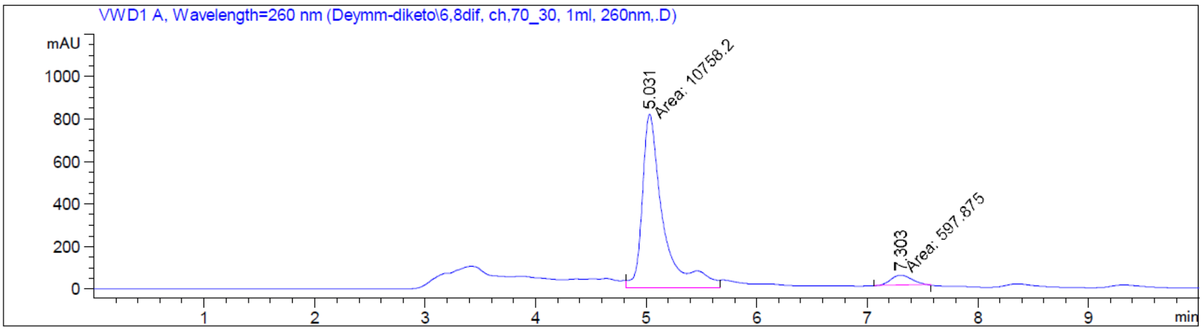

Signal 1: VWD1 A, Wavelength=260 nm

Peak #	RetTime [min]	Type	Width [min]	Area [mAU*s]	Height [mAU]	Area %
1	5.031	FM	0.2194	1.07582e4	817.34760	94.7352
2	7.303	MM	0.2138	597.87482	46.60258	5.2648

Supplementary Fig. 141. HPLC traces of rac-**3l** (top) and chiral-**3l** (bottom).

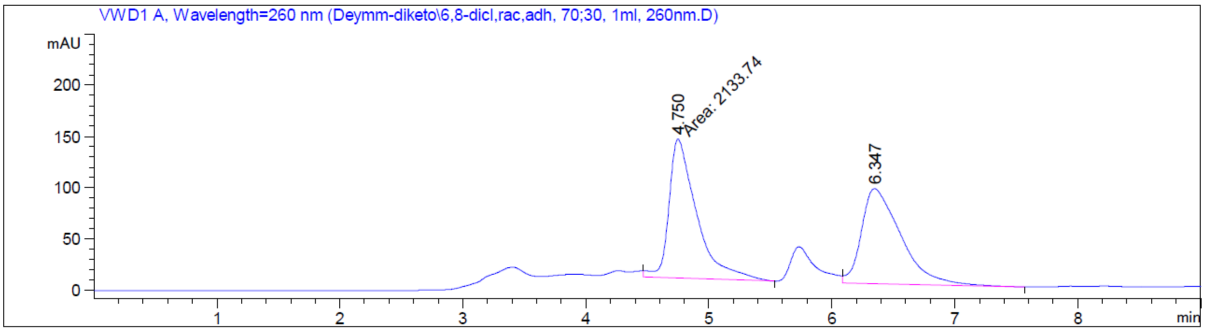

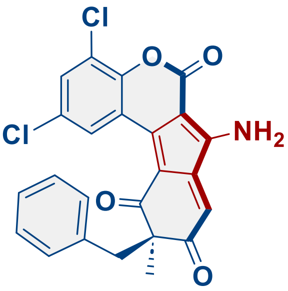

3q

Signal 1: VWD1 A, Wavelength=260 nm

Peak #	RetTime [min]	Type	Width [min]	Area [mAU*s]	Height [mAU]	Area %
1	4.750	FM	0.2623	2133.73999	135.58611	50.8104
2	6.347	VB	0.3544	2065.67432	92.56064	49.1896

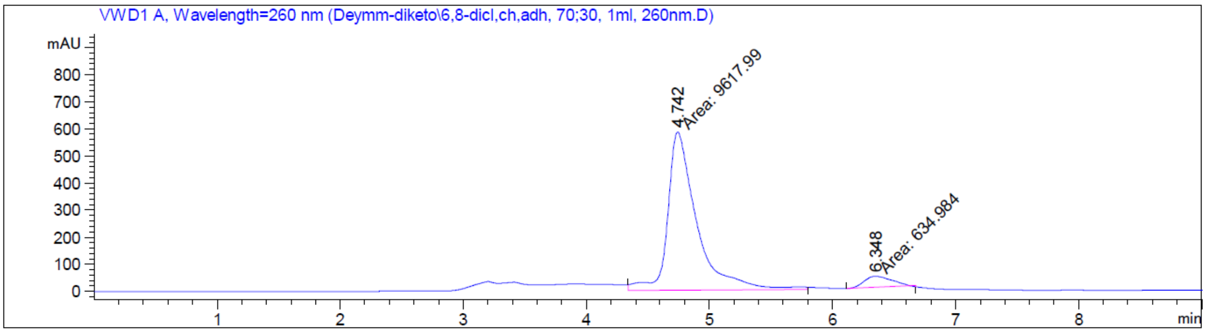

Signal 1: VWD1 A, Wavelength=260 nm

Peak #	RetTime [min]	Type	Width [min]	Area [mAU*s]	Height [mAU]	Area %
1	4.742	FM	0.2742	9617.99414	584.62036	93.8068
2	6.348	MM	0.2578	634.98438	41.04463	6.1932

Supplementary Fig. 142. HPLC traces of rac-**3r** (top) and chiral-**3r** (bottom).

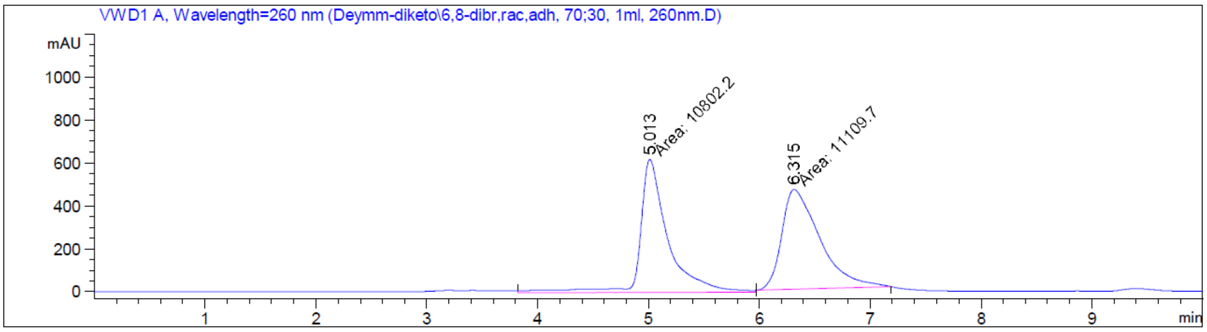

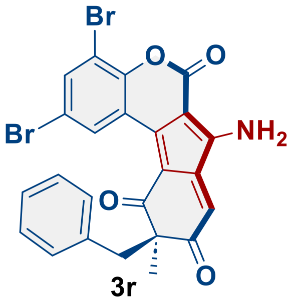

3r

Signal 1: VWD1 A, Wavelength=260 nm

Peak #	RetTime [min]	Type	Width [min]	Area [mAU*s]	Height [mAU]	Area %
1	5.013	MM	0.2909	1.08022e4	618.86444	49.2985
2	6.315	MM	0.3987	1.11097e4	464.38684	50.7015

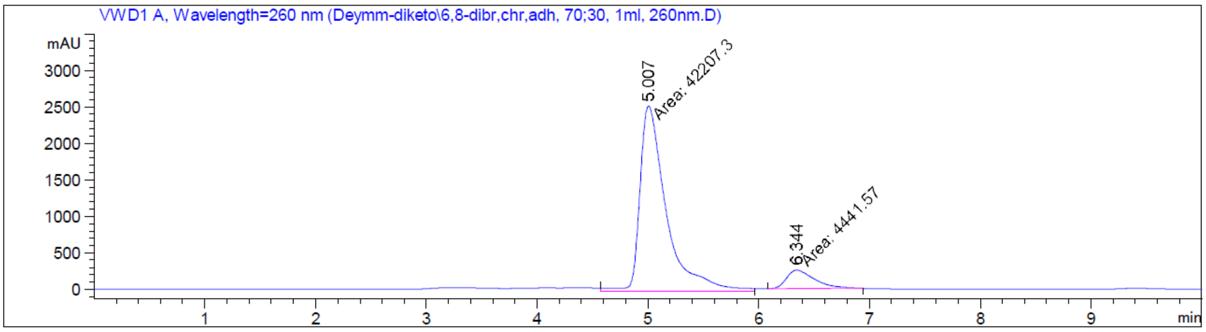

Signal 1: VWD1 A, Wavelength=260 nm

Peak #	RetTime [min]	Type	Width [min]	Area [mAU*s]	Height [mAU]	Area %
1	5.007	MM	0.2771	4.22073e4	2538.36646	90.4787
2	6.344	MM	0.2938	4441.57129	251.95392	9.5213

Supplementary Fig. 143. HPLC traces of rac-**3s** (top) and chiral-**3s** (bottom).

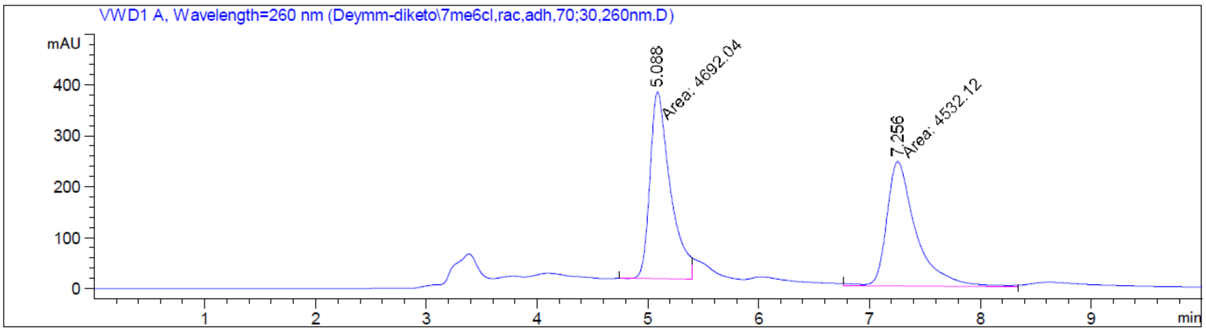

Signal 1: VWD1 A, Wavelength=260 nm

Peak #	RetTime [min]	Type	Width [min]	Area [mAU*s]	Height [mAU]	Area %
1	5.088	MF	0.2130	4692.03516	367.11713	50.8668
2	7.256	MM	0.3090	4532.12451	244.48357	49.1332

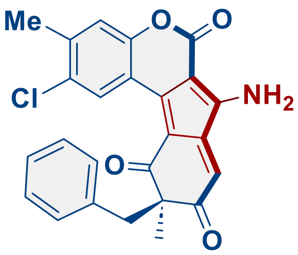

3s

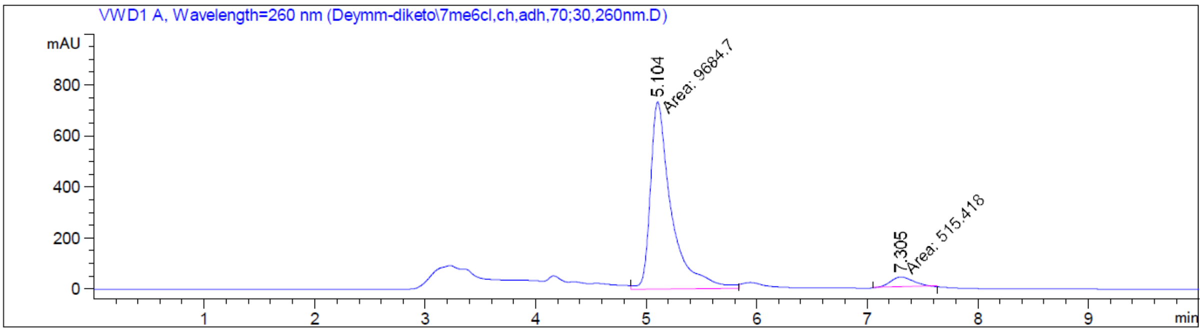

Signal 1: VWD1 A, Wavelength=260 nm

Peak #	RetTime [min]	Type	Width [min]	Area [mAU*s]	Height [mAU]	Area %
1	5.104	MF	0.2200	9684.69824	733.52527	94.9469
2	7.305	MM	0.2286	515.41827	37.58277	5.0531

Supplementary Fig. 144. HPLC traces of rac-**3t** (top) and chiral-**3t** (bottom).

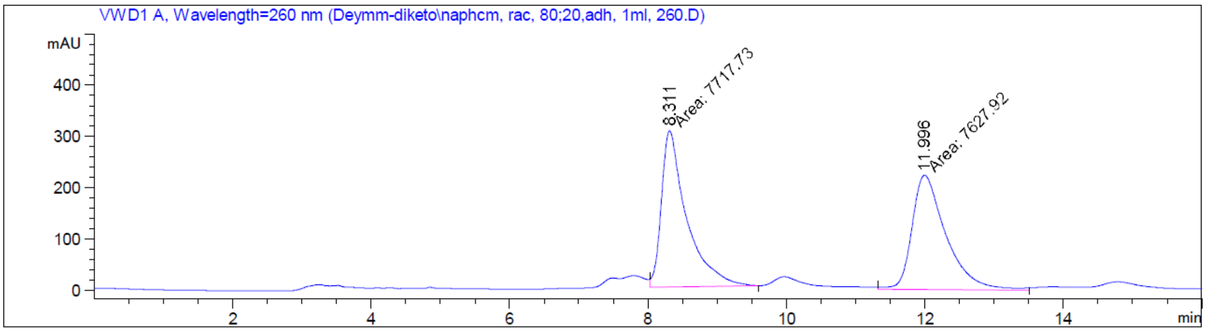

Signal 1: VWD1 A, Wavelength=260 nm

Peak #	RetTime [min]	Type	Width [min]	Area [mAU*s]	Height [mAU]	Area %
1	8.311	FM	0.4220	7717.73486	304.79486	50.2927
2	11.996	MM	0.5702	7627.91504	222.95874	49.7073

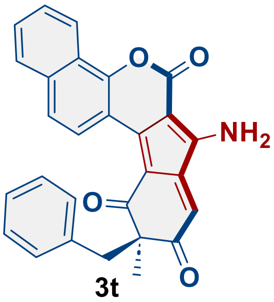

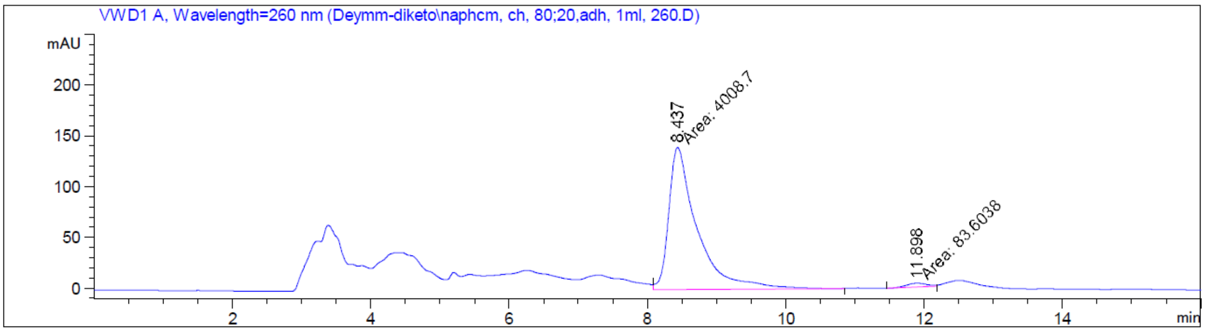

Signal 1: VWD1 A, Wavelength=260 nm

Peak #	RetTime [min]	Type	Width [min]	Area [mAU*s]	Height [mAU]	Area %
1	8.437	FM	0.4785	4008.70288	139.61781	97.9570
2	11.898	MM	0.3604	83.60380	3.86674	2.0430

Supplementary Fig. 145. HPLC traces of rac-**4a** (top) and chiral-**4a** (bottom).

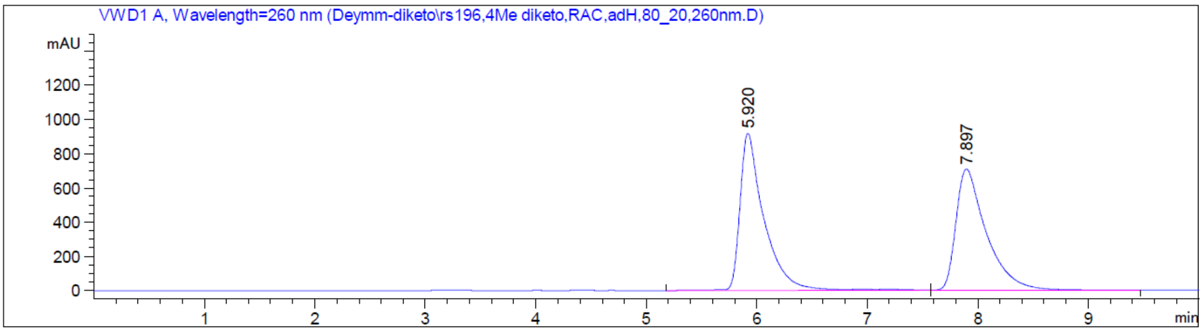

Signal 1: VWD1 A, Wavelength=260 nm

Peak #	RetTime [min]	Type	Width [min]	Area [mAU*s]	Height [mAU]	Area %
1	5.920	VV R	0.2120	1.38626e4	917.54224	50.6440
2	7.897	BB	0.2776	1.35100e4	707.99585	49.3560

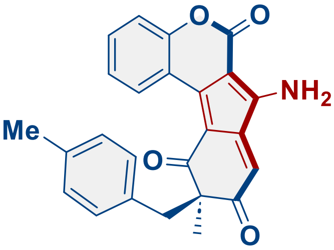

4a

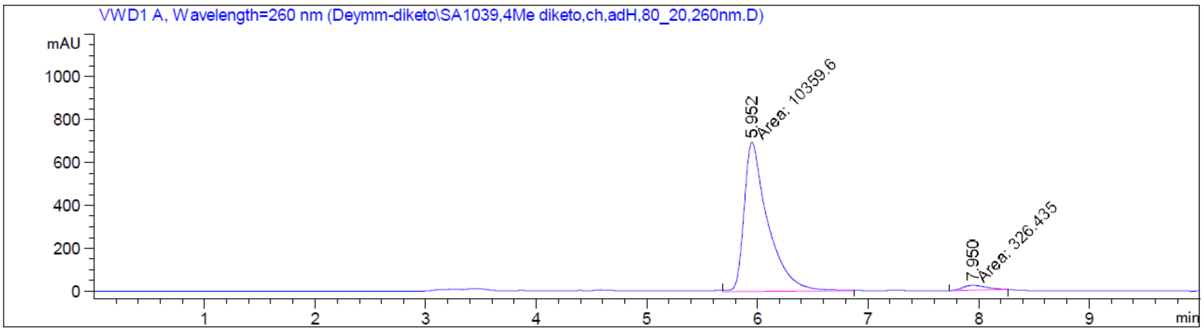

Signal 1: VWD1 A, Wavelength=260 nm

Peak #	RetTime [min]	Type	Width [min]	Area [mAU*s]	Height [mAU]	Area %
1	5.952	MM	0.2478	1.03596e4	696.70612	96.9452
2	7.950	MM	0.2410	326.43460	22.57807	3.0548

Supplementary Fig. 146. HPLC traces of rac-**4b** (top) and chiral-**4b** (bottom).

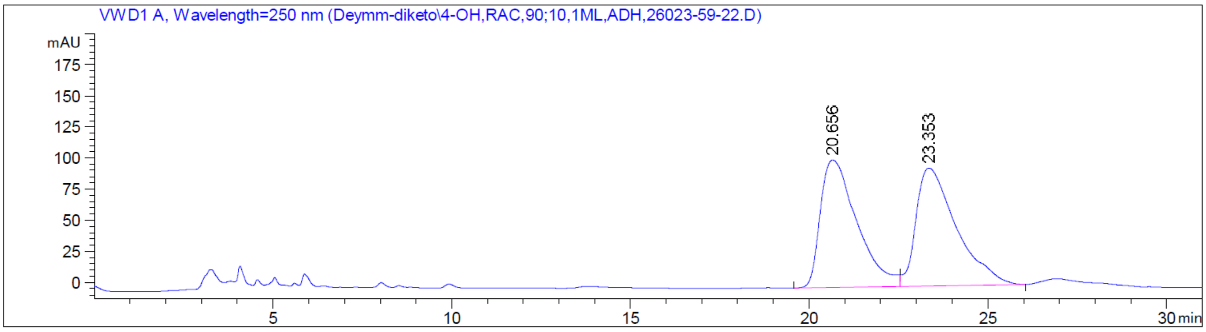

Signal 1: VWD1 A, Wavelength=250 nm

Peak #	RetTime [min]	Type	Width [min]	Area [mAU*s]	Height [mAU]	Area %
1	20.656	BV	1.0609	7296.18994	102.56881	49.4917
2	23.353	VB	1.1497	7446.06348	94.76421	50.5083

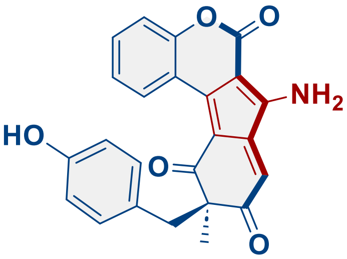

4b

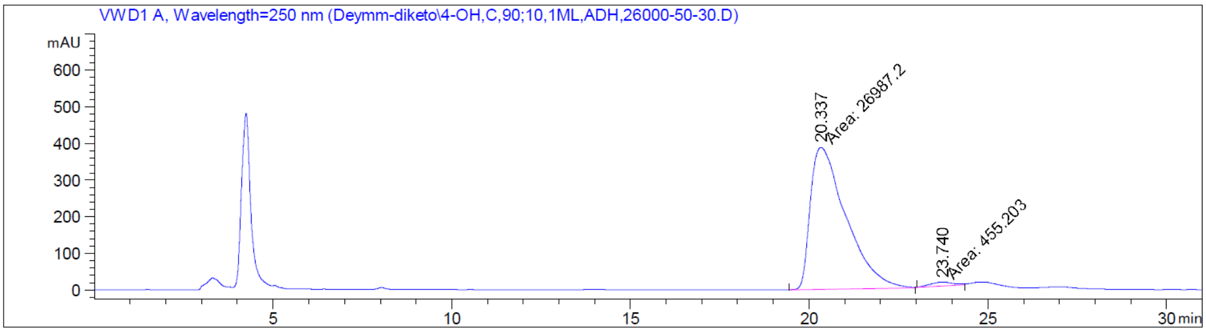

Signal 1: VWD1 A, Wavelength=250 nm

Peak #	RetTime [min]	Type	Width [min]	Area [mAU*s]	Height [mAU]	Area %
1	20.337	MM	1.1595	2.69872e4	387.90546	98.3412
2	23.740	MM	0.7751	455.20258	9.78835	1.6588

Supplementary Fig. 147. HPLC traces of rac-**4c** (top) and chiral-**4c** (bottom).

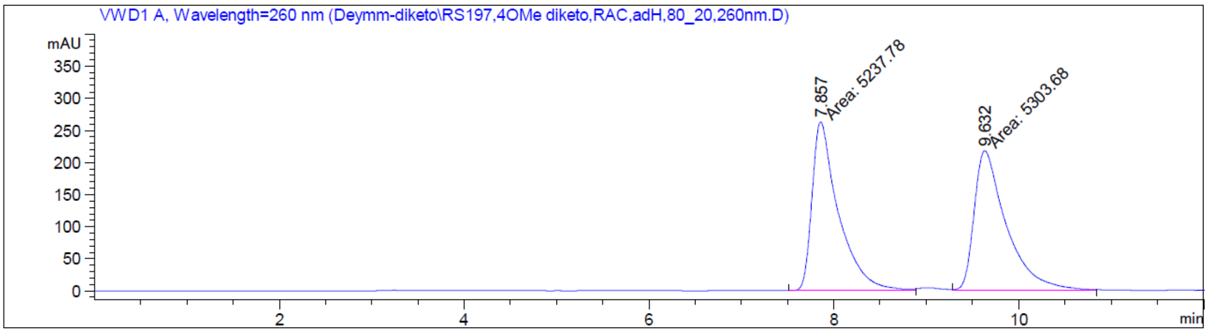

Signal 1: VWD1 A, Wavelength=260 nm

Peak #	RetTime [min]	Type	Width [min]	Area [mAU*s]	Height [mAU]	Area %
1	7.857	MF	0.3319	5237.78027	263.03485	49.6874
2	9.632	MF	0.4063	5303.67725	217.53783	50.3126

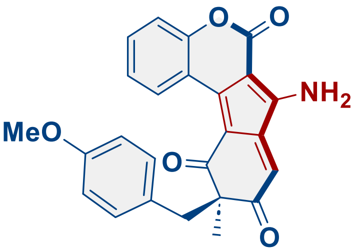

4c

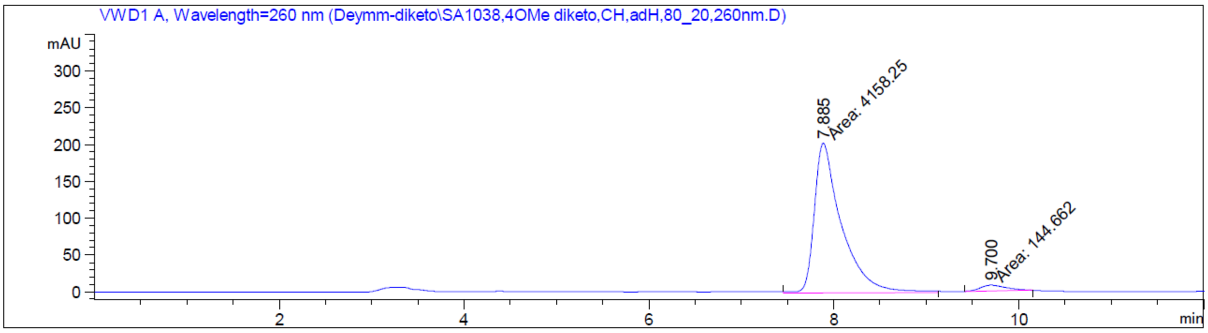

Signal 1: VWD1 A, Wavelength=260 nm

Peak #	RetTime [min]	Type	Width [min]	Area [mAU*s]	Height [mAU]	Area %
1	7.885	MF	0.3414	4158.25146	202.99203	96.6380
2	9.700	MM	0.3099	144.66231	7.78000	3.3620

Supplementary Fig. 148. HPLC traces of rac-**4d** (top) and chiral-**4d** (bottom).

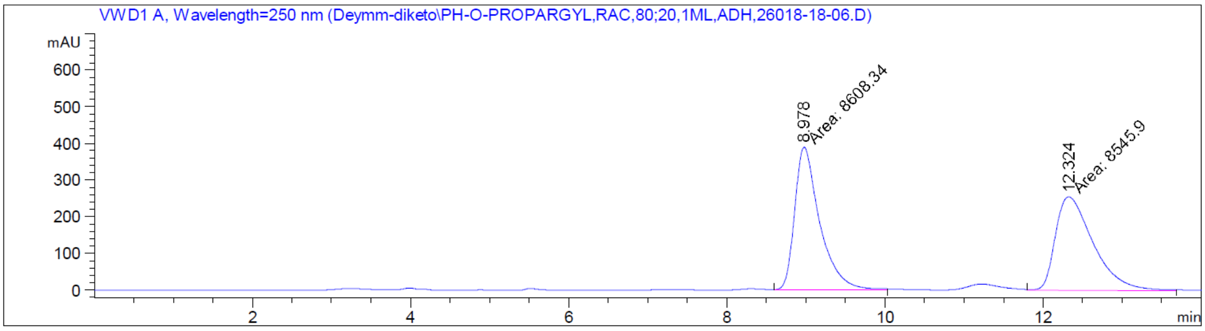

Signal 1: VWD1 A, Wavelength=250 nm

Peak #	RetTime [min]	Type	Width [min]	Area [mAU*s]	Height [mAU]	Area %
1	8.978	MF	0.3682	8608.33984	389.68414	50.1820
2	12.324	FM	0.5579	8545.89551	255.30988	49.8180

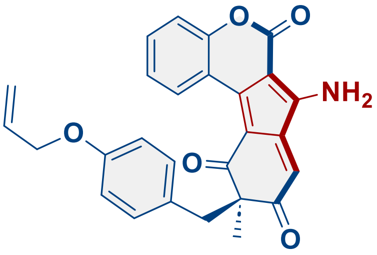

4d

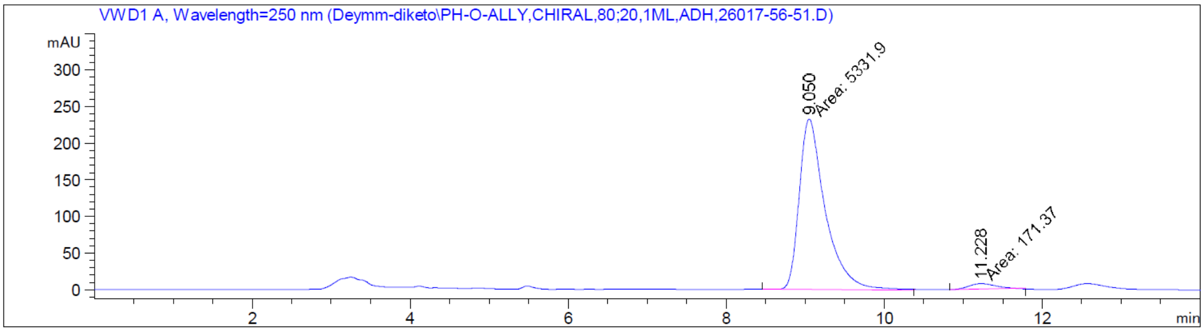

Signal 1: VWD1 A, Wavelength=250 nm

Peak #	RetTime [min]	Type	Width [min]	Area [mAU*s]	Height [mAU]	Area %
1	9.050	MF	0.3821	5331.90039	232.59875	96.8860
2	11.228	MM	0.3843	171.36978	7.43116	3.1140

Supplementary Fig. 149. HPLC traces of rac-**4e** (top) and chiral-**4e** (bottom).

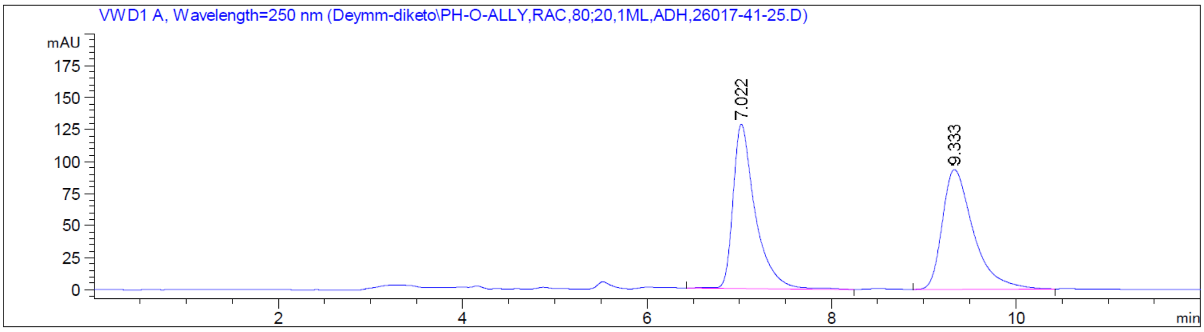

Signal 1: VWD1 A, Wavelength=250 nm

Peak #	RetTime [min]	Type	Width [min]	Area [mAU*s]	Height [mAU]	Area %
1	7.022	VB R	0.2452	2150.40210	128.51517	49.6078
2	9.333	BB	0.3517	2184.40112	93.44664	50.3922

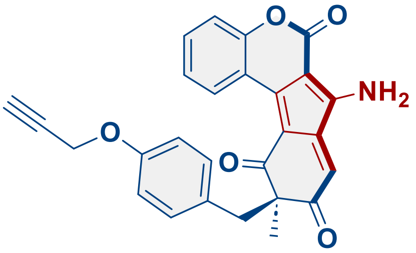

4e

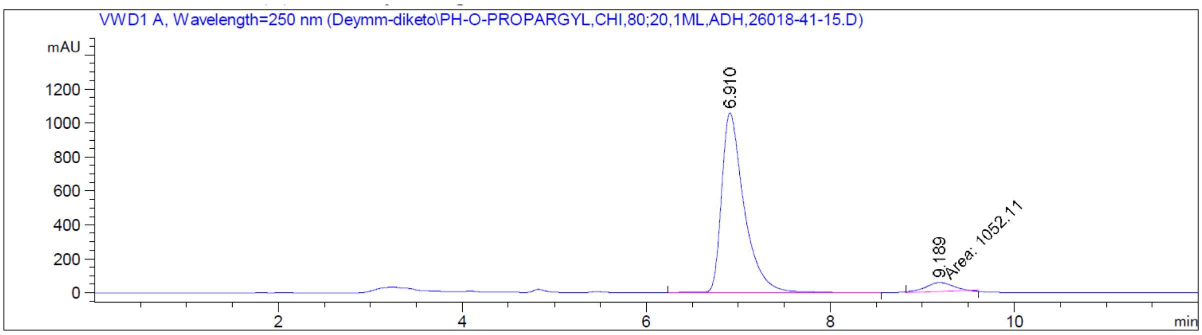

Signal 1: VWD1 A, Wavelength=250 nm

Peak #	RetTime [min]	Type	Width [min]	Area [mAU*s]	Height [mAU]	Area %
1	6.910	VB R	0.2514	1.80175e4	1057.41675	94.4828
2	9.189	MM	0.3399	1052.11157	51.58502	5.5172

Supplementary Fig. 150. HPLC traces of rac-**4f** (top) and chiral-**4f** (bottom).

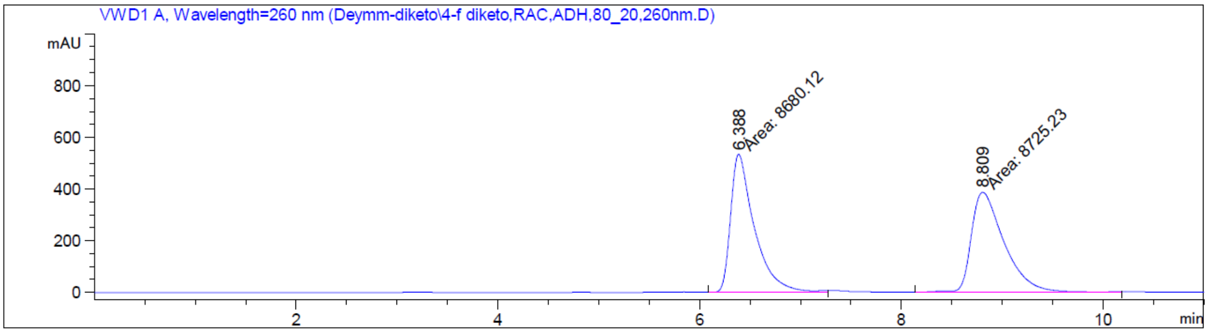

Signal 1: VWD1 A, Wavelength=260 nm

Peak #	RetTime [min]	Type	Width [min]	Area [mAU*s]	Height [mAU]	Area %
1	6.388	MM	0.2714	8680.11523	533.02069	49.8704
2	8.809	MF	0.3769	8725.23242	385.82623	50.1296

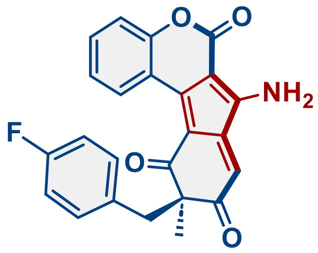

4f

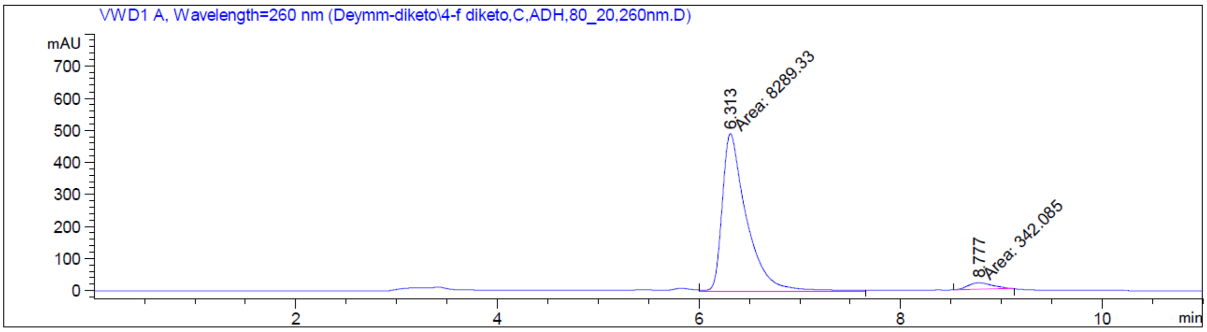

Signal 1: VWD1 A, Wavelength=260 nm

Peak #	RetTime [min]	Type	Width [min]	Area [mAU*s]	Height [mAU]	Area %
1	6.313	MF	0.2810	8289.32520	491.73123	96.0367
2	8.777	MM	0.2817	342.08459	20.24142	3.9633

Supplementary Fig. 151. HPLC traces of rac-**4g** (top) and chiral-**4g** (bottom).

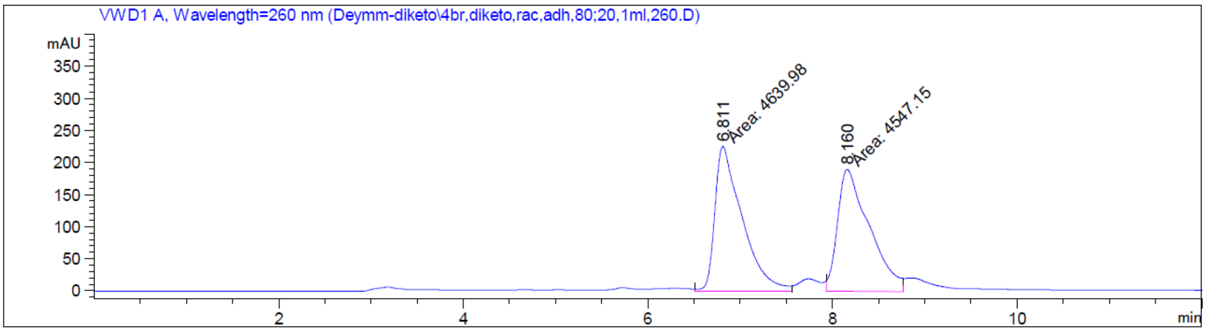

Signal 1: VWD1 A, Wavelength=260 nm

Peak #	RetTime [min]	Type	Width [min]	Area [mAU*s]	Height [mAU]	Area %
1	6.811	MF	0.3434	4639.98291	225.19049	50.5052
2	8.160	MF	0.3994	4547.15137	189.73978	49.4948

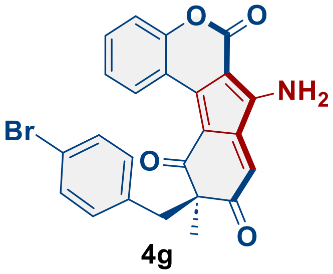

4g

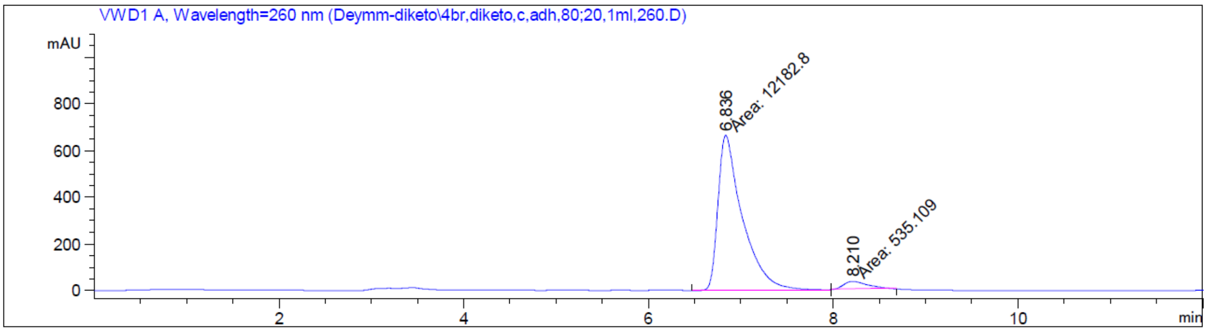

Signal 1: VWD1 A, Wavelength=260 nm

Peak #	RetTime [min]	Type	Width [min]	Area [mAU*s]	Height [mAU]	Area %
1	6.836	MM	0.3060	1.21828e4	663.50488	95.7925
2	8.210	MM	0.2777	535.10913	32.11537	4.2075

Supplementary Fig. 152. HPLC traces of rac-**4h** (top) and chiral-**4h** (bottom).

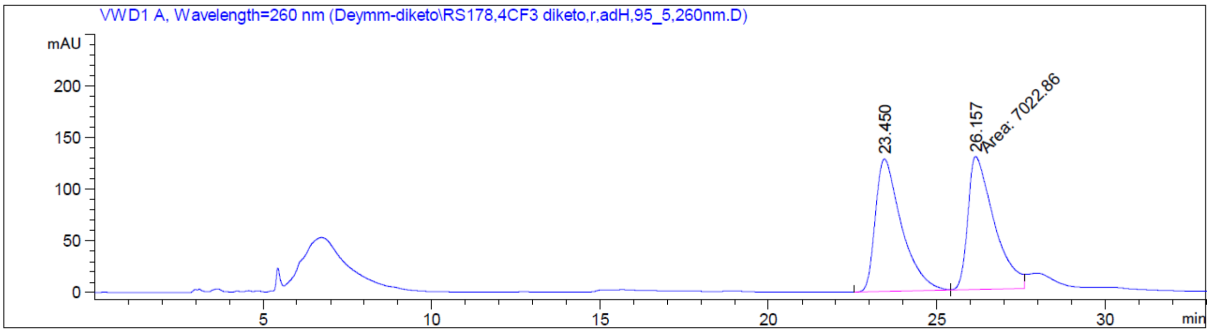


```
Signal 1: VWD1 A, Wavelength=260 nm

Peak RetTime Type  Width     Area      Height     Area
  #   [min]        [min]   [mAU*s]     [mAU]        %
----|-------|----|-------|----------|----------|--------|
   1  23.450 BV    0.7871 6934.36865  128.18233  49.6830
   2  26.157 MF    0.9080 7022.85986  128.90636  50.3170
```

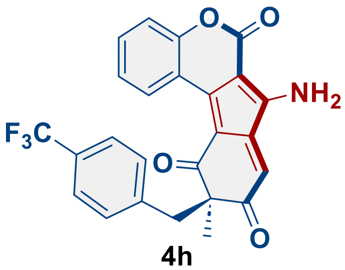

4h

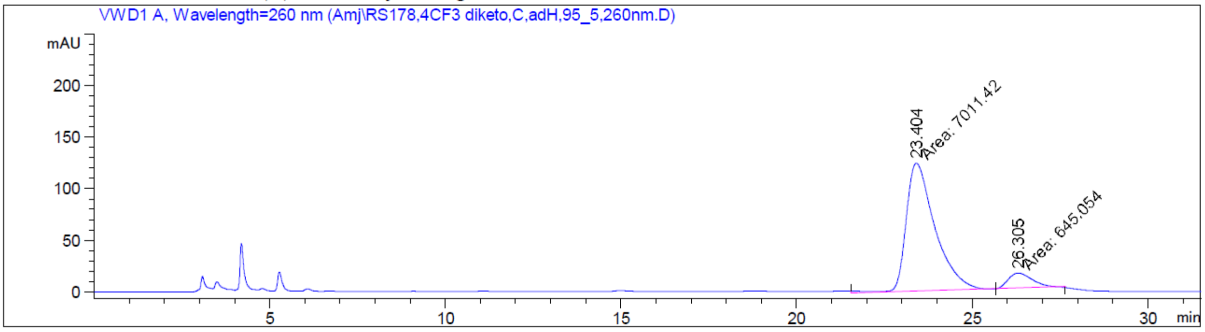


```
Signal 1: VWD1 A, Wavelength=260 nm

Peak RetTime Type  Width     Area      Height     Area
  #   [min]        [min]   [mAU*s]     [mAU]        %
----|-------|----|-------|----------|----------|--------|
   1  23.404 MF    0.9444 7011.41846  123.73085  91.5751
   2  26.305 FM    0.7625  645.05371   14.09920   8.4249
```

Supplementary Fig. 153. HPLC traces of rac-**4i** (top) and chiral-**4i** (bottom).

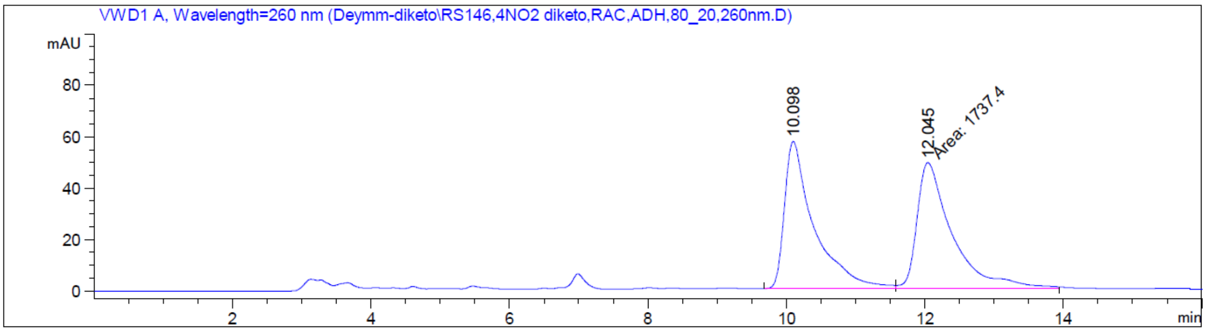

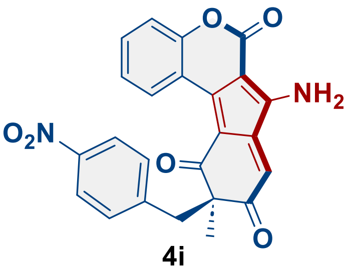

4i

Signal 1: VWD1 A, Wavelength=260 nm

Peak #	RetTime [min]	Type	Width [min]	Area [mAU*s]	Height [mAU]	Area %
1	10.098	BV	0.4149	1700.97253	57.20784	49.4703
2	12.045	MF	0.5906	1737.39856	49.02770	50.5297

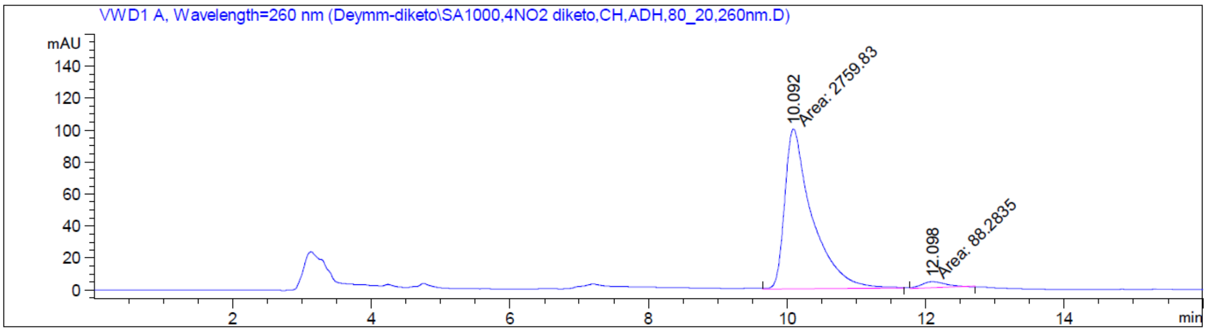

Signal 1: VWD1 A, Wavelength=260 nm

Peak #	RetTime [min]	Type	Width [min]	Area [mAU*s]	Height [mAU]	Area %
1	10.092	MM	0.4601	2759.83154	99.98194	96.9003
2	12.098	MM	0.3933	88.28349	3.74127	3.0997

Supplementary Fig. 154. HPLC traces of rac-**4j** (top) and chiral-**4j** (bottom).

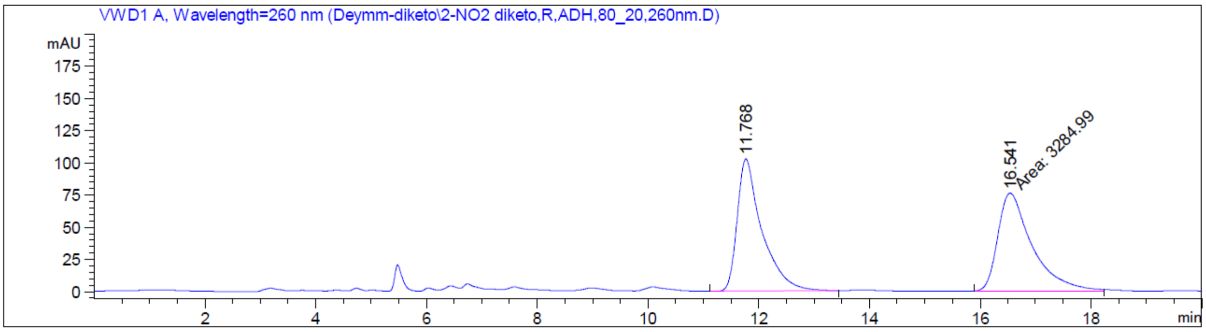

Signal 1: VWD1 A, Wavelength=260 nm

Peak #	RetTime [min]	Type	Width [min]	Area [mAU*s]	Height [mAU]	Area %
1	11.768	BB	0.4425	3173.49854	102.42148	49.1368
2	16.541	MF	0.7209	3284.99292	75.94944	50.8632

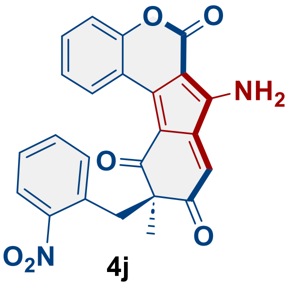

4j

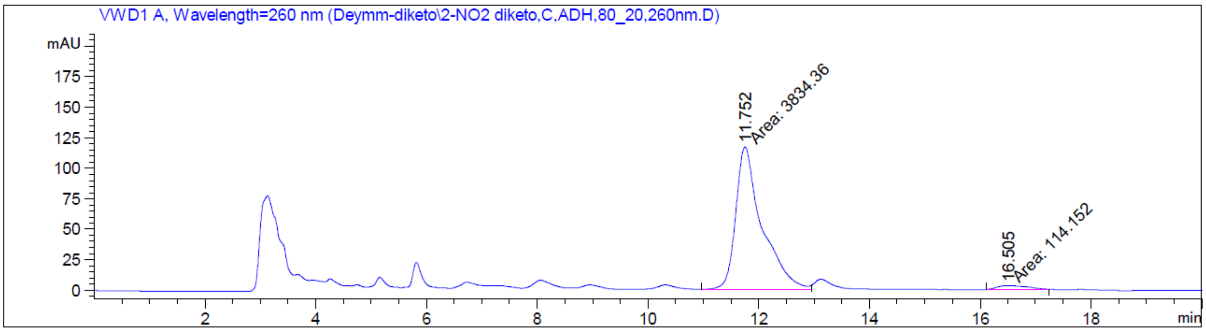

Signal 1: VWD1 A, Wavelength=260 nm

Peak #	RetTime [min]	Type	Width [min]	Area [mAU*s]	Height [mAU]	Area %
1	11.752	MF	0.5481	3834.36304	116.59351	97.1090
2	16.505	MM	0.6201	114.15225	3.06818	2.8910

Supplementary Fig. 155. HPLC traces of rac-**4k** (top) and chiral-**4k** (bottom).

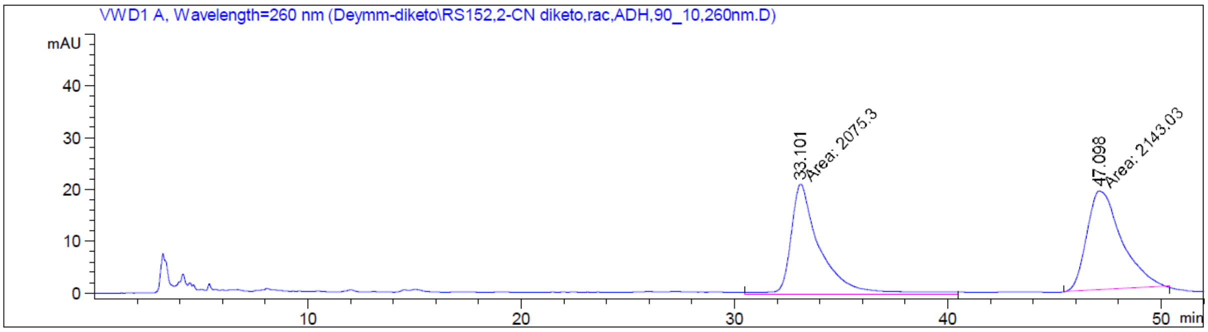

Signal 1: VWD1 A, Wavelength=260 nm

Peak #	RetTime [min]	Type	Width [min]	Area [mAU*s]	Height [mAU]	Area %
1	33.101	MM	1.6241	2075.29517	21.29716	49.1971
2	47.098	MM	1.8787	2143.03271	19.01126	50.8029

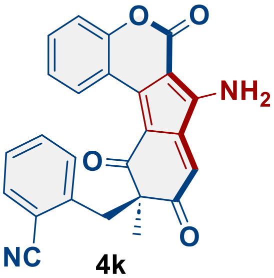

4k

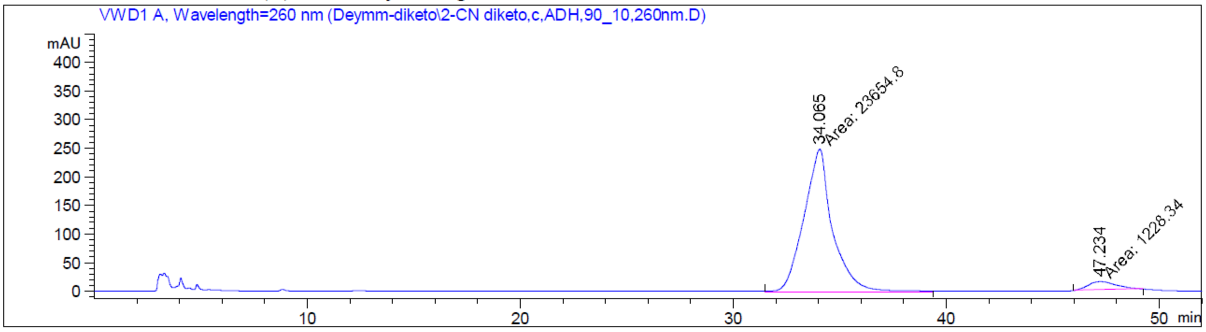

Signal 1: VWD1 A, Wavelength=260 nm

Peak #	RetTime [min]	Type	Width [min]	Area [mAU*s]	Height [mAU]	Area %
1	34.065	MM	1.5777	2.36548e4	249.89302	95.0636
2	47.234	MM	1.4721	1228.34216	13.90702	4.9364

Supplementary Fig. 156. HPLC traces of rac-**4l** (top) and chiral-**4l** (bottom).

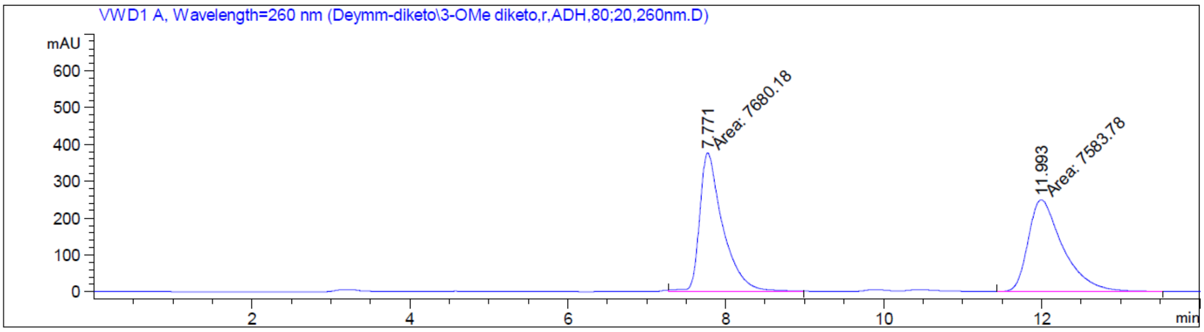

Signal 1: VWD1 A, Wavelength=260 nm

Peak #	RetTime [min]	Type	Width [min]	Area [mAU*s]	Height [mAU]	Area %
1	7.771	MF	0.3401	7680.17969	376.33246	50.3158
2	11.993	MF	0.5081	7583.77588	248.75452	49.6842

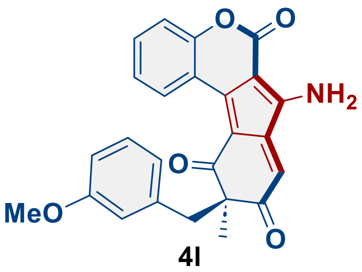

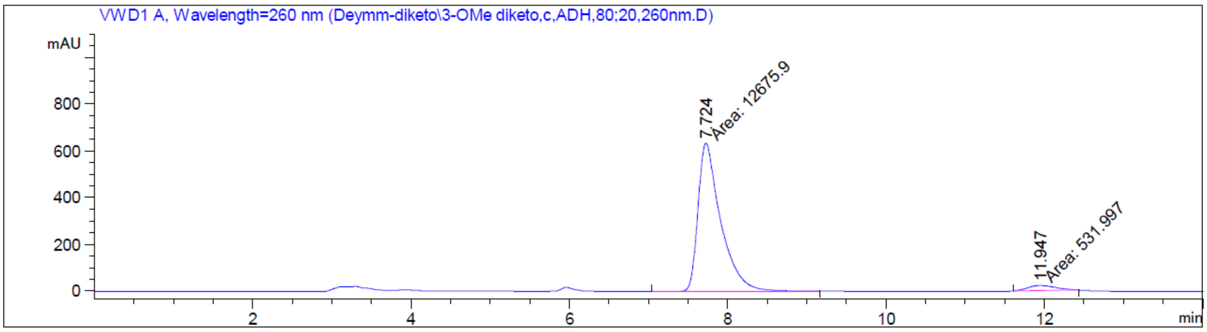

Signal 1: VWD1 A, Wavelength=260 nm

Peak #	RetTime [min]	Type	Width [min]	Area [mAU*s]	Height [mAU]	Area %
1	7.724	MF	0.3336	1.26759e4	633.34680	95.9721
2	11.947	MM	0.4061	531.99738	21.83619	4.0279

Supplementary Fig. 157. HPLC traces of rac-**4m** (top) and chiral-**4m** (bottom).

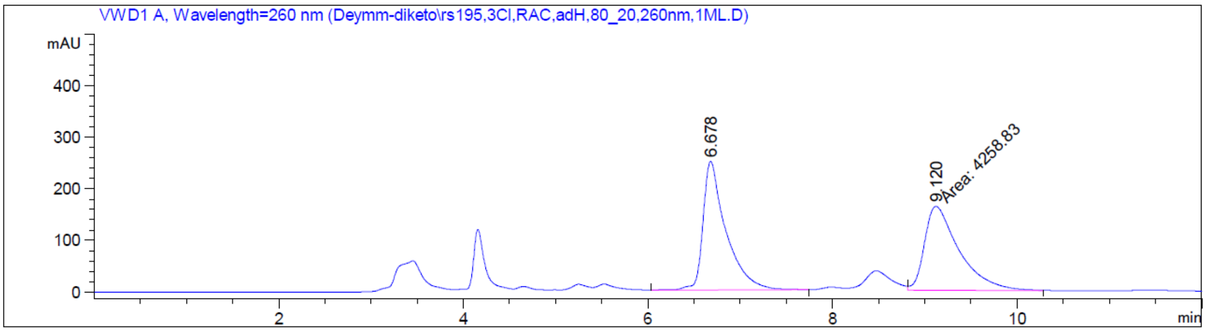

Signal 1: VWD1 A, Wavelength=260 nm

Peak #	RetTime [min]	Type	Width [min]	Area [mAU*s]	Height [mAU]	Area %
1	6.678	VB R	0.2445	4327.81250	249.29245	50.4017
2	9.120	MF	0.4361	4258.83057	162.77354	49.5983

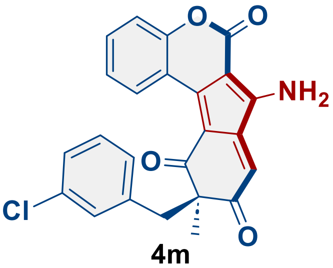

4m

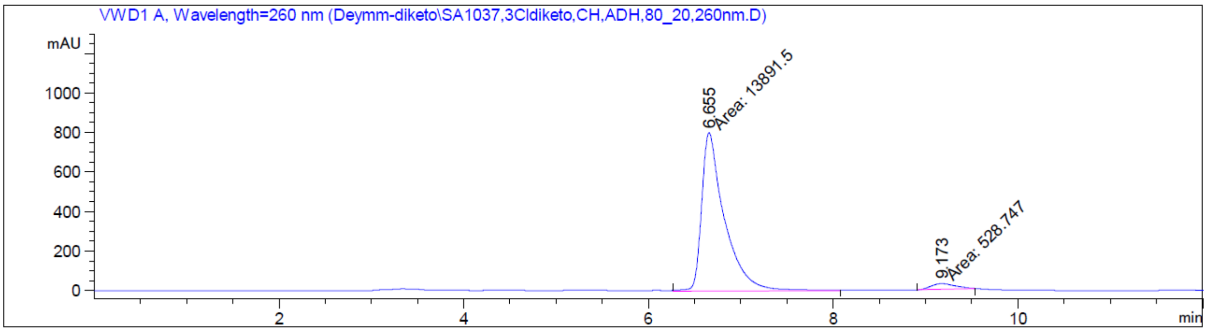

Signal 1: VWD1 A, Wavelength=260 nm

Peak #	RetTime [min]	Type	Width [min]	Area [mAU*s]	Height [mAU]	Area %
1	6.655	MM	0.2884	1.38915e4	802.74786	96.3333
2	9.173	MM	0.3089	528.74677	28.52872	3.6667

Supplementary Fig. 158. HPLC traces of rac-**4n** (top) and chiral-**4n** (bottom).

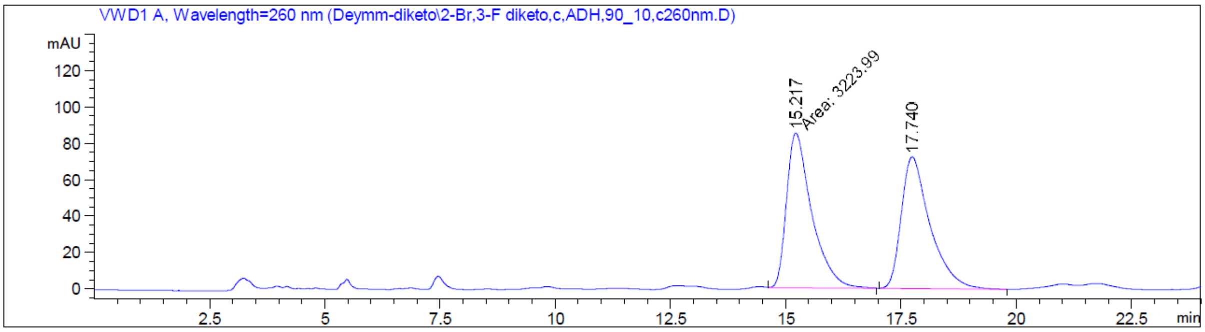

Signal 1: VWD1 A, Wavelength=260 nm

Peak #	RetTime [min]	Type	Width [min]	Area [mAU*s]	Height [mAU]	Area %
1	15.217	MM	0.6325	3223.98755	84.95377	50.7838
2	17.740	BB	0.6395	3124.46582	72.25005	49.2162

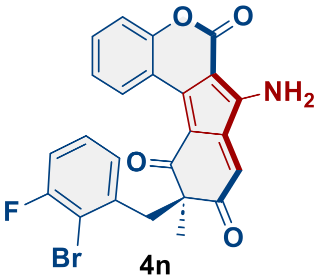

4n

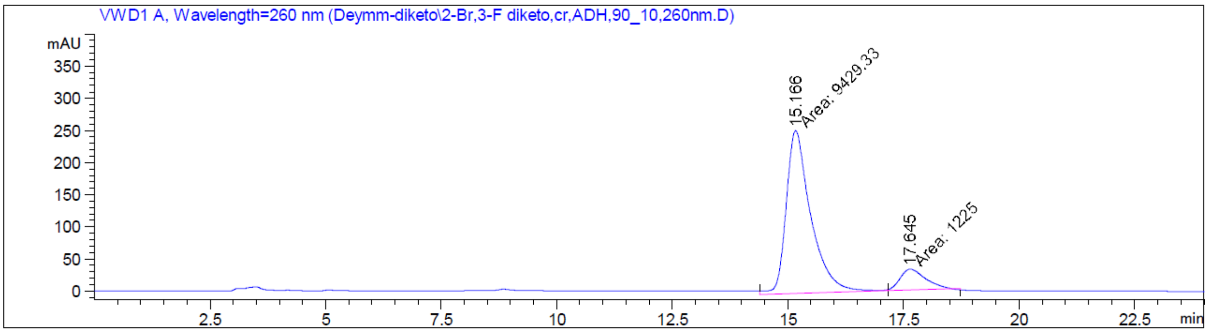

Signal 1: VWD1 A, Wavelength=260 nm

Peak #	RetTime [min]	Type	Width [min]	Area [mAU*s]	Height [mAU]	Area %
1	15.166	MF	0.6198	9429.33496	253.54562	88.5024
2	17.645	FM	0.6330	1224.99646	32.25348	11.4976

Supplementary Fig. 159. HPLC traces of rac-**4o** (top) and chiral-**4o** (bottom).

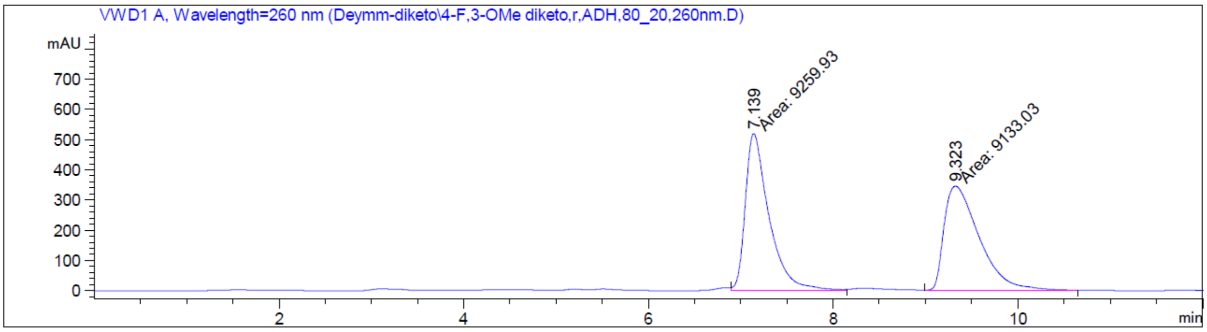

Signal 1: VWD1 A, Wavelength=260 nm

Peak #	RetTime [min]	Type	Width [min]	Area [mAU*s]	Height [mAU]	Area %
1	7.139	MF	0.2968	9259.93164	519.90973	50.3450
2	9.323	MF	0.4418	9133.03320	344.54977	49.6550

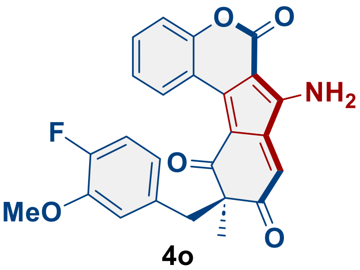

4o

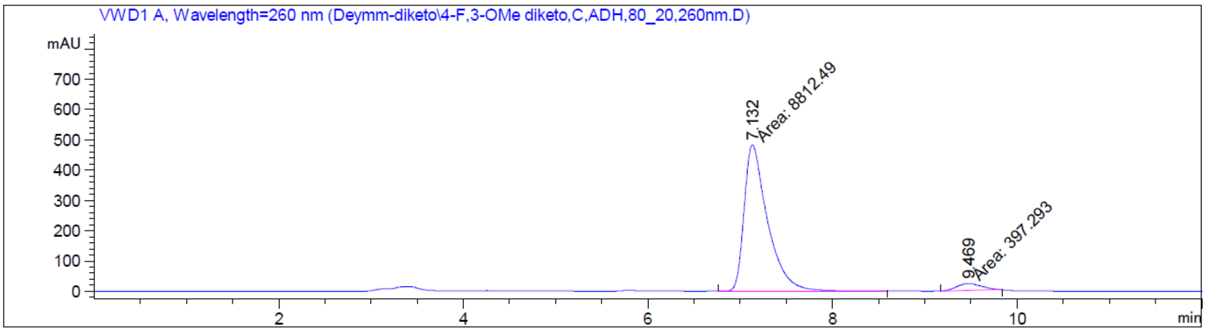

Signal 1: VWD1 A, Wavelength=260 nm

Peak #	RetTime [min]	Type	Width [min]	Area [mAU*s]	Height [mAU]	Area %
1	7.132	MM	0.3016	8812.48828	487.00226	95.6862
2	9.469	MM	0.3034	397.29254	21.82663	4.3138

Supplementary Fig. 160. HPLC traces of rac-**4p** (top) and chiral-**4p** (bottom).

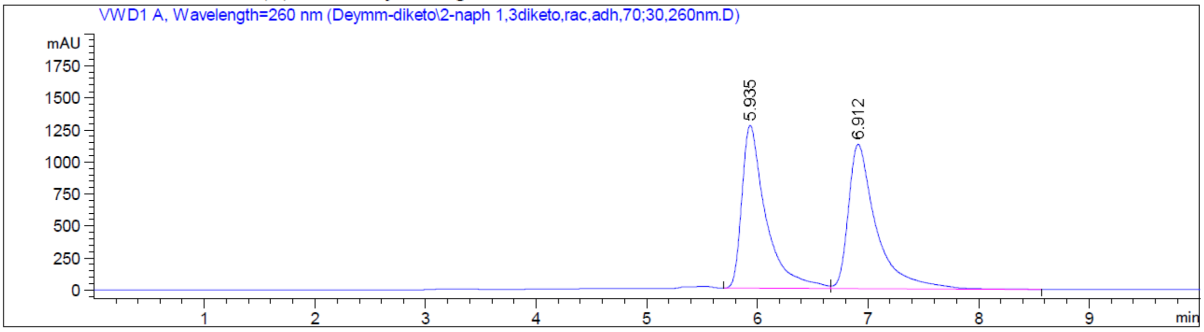

Signal 1: VWD1 A, Wavelength=260 nm

Peak #	RetTime [min]	Type	Width [min]	Area [mAU*s]	Height [mAU]	Area %
1	5.935	BV	0.2175	1.92441e4	1272.25464	49.3390
2	6.912	VB	0.2536	1.97597e4	1126.56543	50.6610

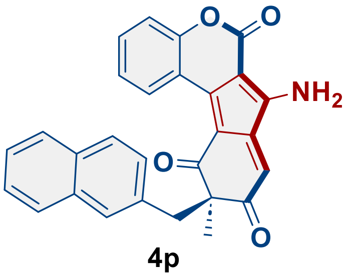

4p

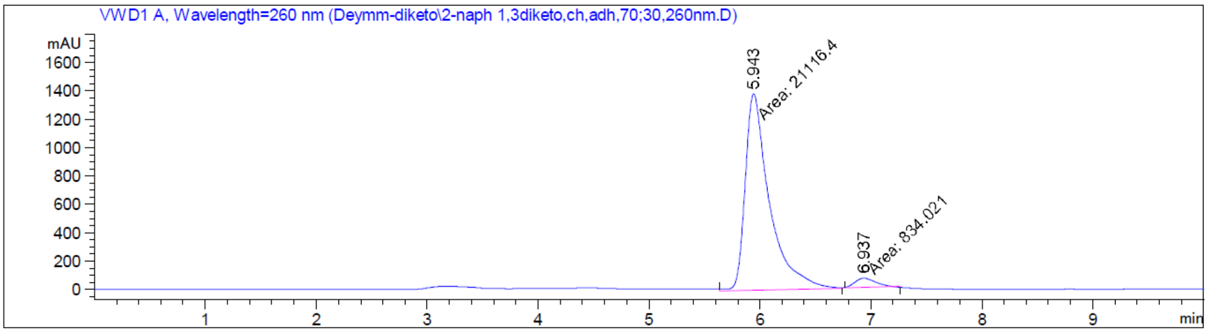

Signal 1: VWD1 A, Wavelength=260 nm

Peak #	RetTime [min]	Type	Width [min]	Area [mAU*s]	Height [mAU]	Area %
1	5.943	MM	0.2543	2.11164e4	1383.99829	96.2004
2	6.937	MM	0.2154	834.02148	64.54496	3.7996

Supplementary Fig. 161. HPLC traces of rac-**4q** (top) and chiral-**4q** (bottom).

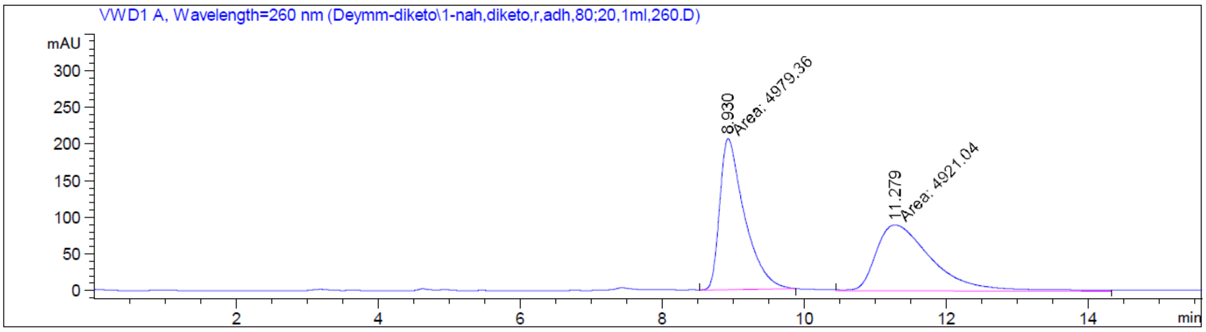

Signal 1: VWD1 A, Wavelength=260 nm

Peak #	RetTime [min]	Type	Width [min]	Area [mAU*s]	Height [mAU]	Area %
1	8.930	MF	0.4033	4979.35693	205.79277	50.2945
2	11.279	MM	0.9144	4921.03662	89.69706	49.7055

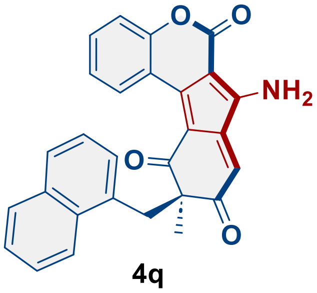

4q

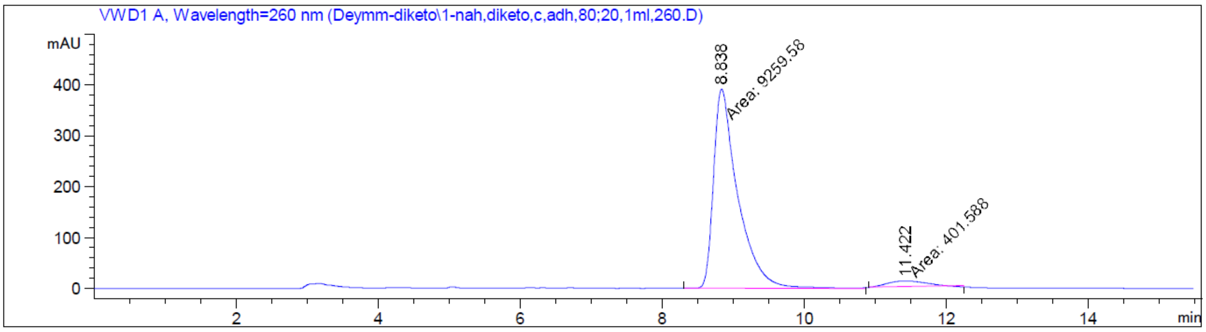

Signal 1: VWD1 A, Wavelength=260 nm

Peak #	RetTime [min]	Type	Width [min]	Area [mAU*s]	Height [mAU]	Area %
1	8.838	MM	0.3949	9259.58008	390.79849	95.8433
2	11.422	MM	0.5989	401.58774	11.17512	4.1567

Supplementary Fig. 162. HPLC traces of rac-**4r** (top) and chiral-**4r** (bottom).

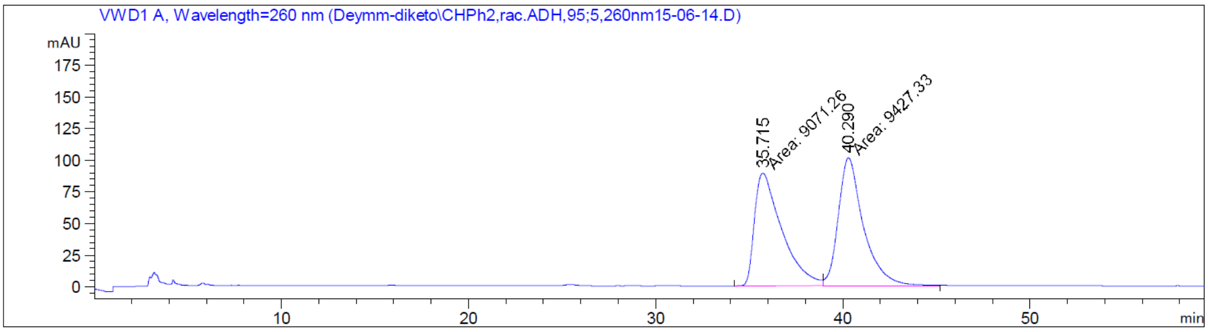

Signal 1: VWD1 A, Wavelength=260 nm

Peak #	RetTime [min]	Type	Width [min]	Area [mAU*s]	Height [mAU]	Area %
1	35.715	MF	1.6968	9071.25879	89.10297	49.0376
2	40.290	FM	1.5531	9427.32715	101.16959	50.9624

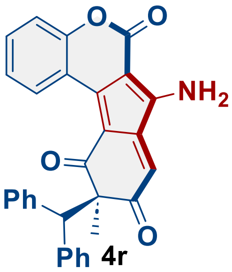

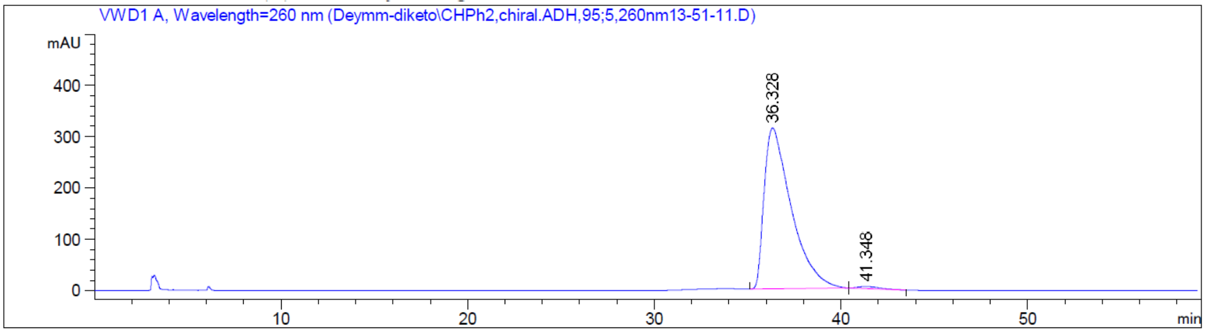

Signal 1: VWD1 A, Wavelength=260 nm

Peak #	RetTime [min]	Type	Width [min]	Area [mAU*s]	Height [mAU]	Area %
1	36.328	BB	1.4374	3.16519e4	313.37714	99.0043
2	41.348	BB	0.9399	318.33914	4.26450	0.9957

Supplementary Fig. 163. HPLC traces of rac-**4s** (top) and chiral-**4s** (bottom).

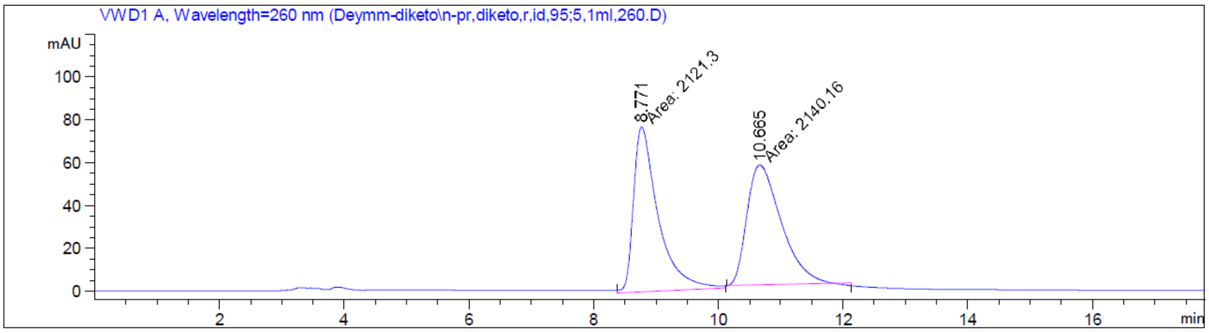

Signal 1: VWD1 A, Wavelength=260 nm

Peak #	RetTime [min]	Type	Width [min]	Area [mAU*s]	Height [mAU]	Area %
1	8.771	MM	0.4597	2121.30054	76.91224	49.7787
2	10.665	MM	0.6384	2140.15894	55.87708	50.2213

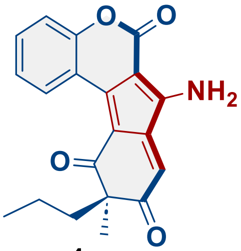

4s

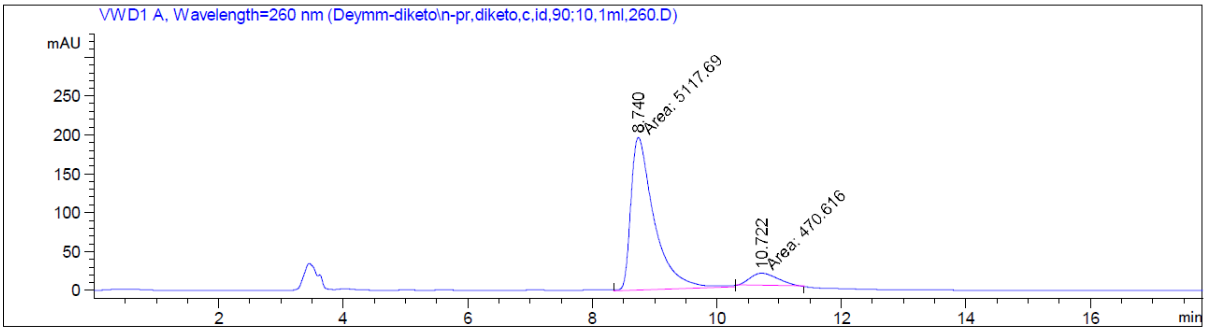

Signal 1: VWD1 A, Wavelength=260 nm

Peak #	RetTime [min]	Type	Width [min]	Area [mAU*s]	Height [mAU]	Area %
1	8.740	MM	0.4348	5117.69141	196.17313	91.5786
2	10.722	MM	0.5090	470.61603	15.40878	8.4214

Supplementary Fig. 164. HPLC traces of rac-**4t** (top) and chiral-**4t** (bottom).

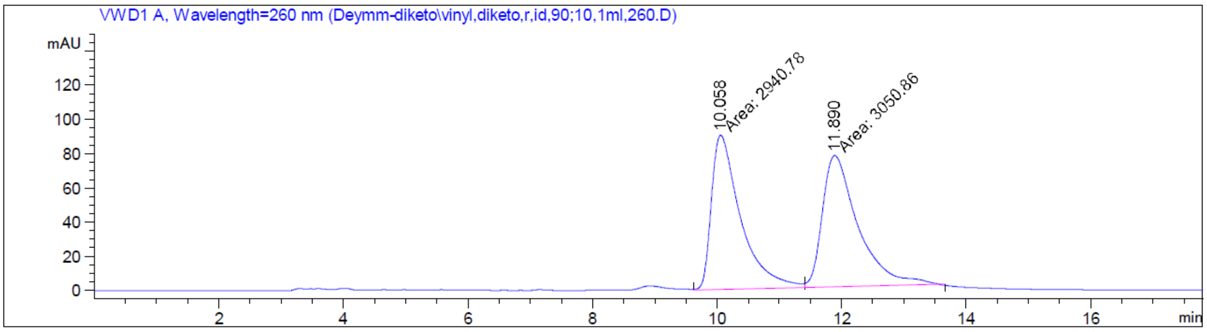

Signal 1: VWD1 A, Wavelength=260 nm

Peak #	RetTime [min]	Type	Width [min]	Area [mAU*s]	Height [mAU]	Area %
1	10.058	MF	0.5439	2940.77930	90.11033	49.0813
2	11.890	FM	0.6630	3050.86426	76.69544	50.9187

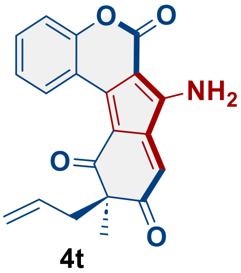

4t

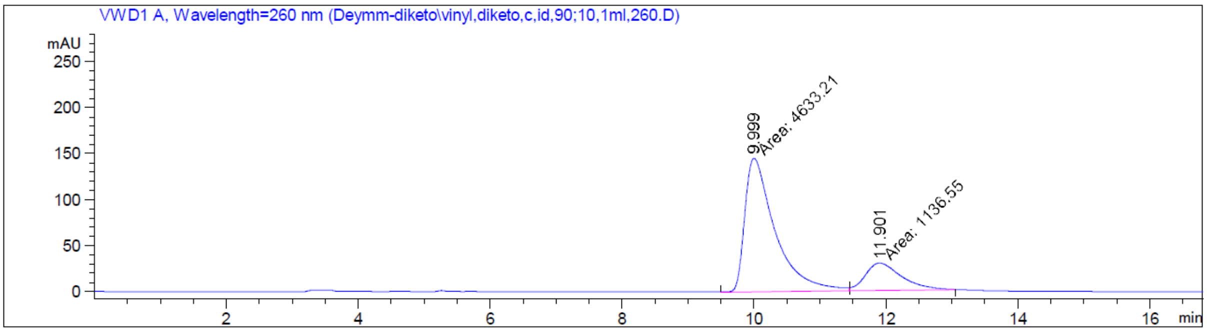

Signal 1: VWD1 A, Wavelength=260 nm

Peak #	RetTime [min]	Type	Width [min]	Area [mAU*s]	Height [mAU]	Area %
1	9.999	MF	0.5316	4633.20898	145.25284	80.3015
2	11.901	MF	0.6365	1136.55457	29.76272	19.6985

Supplementary Fig. 165. HPLC traces of rac-**4u** (top) and chiral-**4u** (bottom).

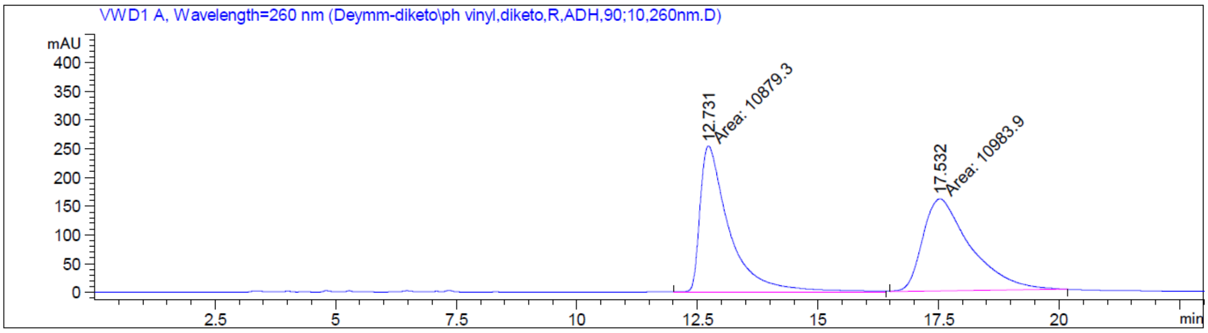

Signal 1: VWD1 A, Wavelength=260 nm

Peak #	RetTime [min]	Type	Width [min]	Area [mAU*s]	Height [mAU]	Area %
1	12.731	MM	0.7118	1.08793e4	254.73091	49.7606
2	17.532	MM	1.1429	1.09839e4	160.17987	50.2394

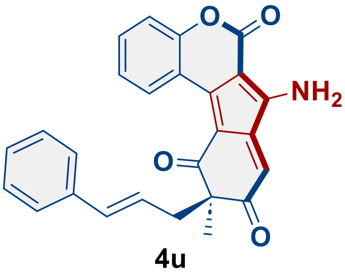

4u

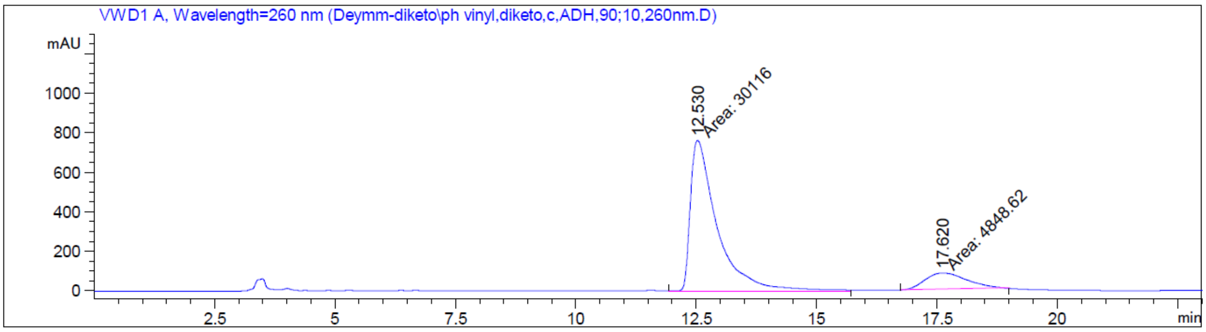

Signal 1: VWD1 A, Wavelength=260 nm

Peak #	RetTime [min]	Type	Width [min]	Area [mAU*s]	Height [mAU]	Area %
1	12.530	MM	0.6562	3.01160e4	764.93420	86.1328
2	17.620	MM	0.9947	4848.61963	81.23940	13.8672

Supplementary Fig. 166. HPLC traces of rac-**4v** (top) and chiral-**4v** (bottom).

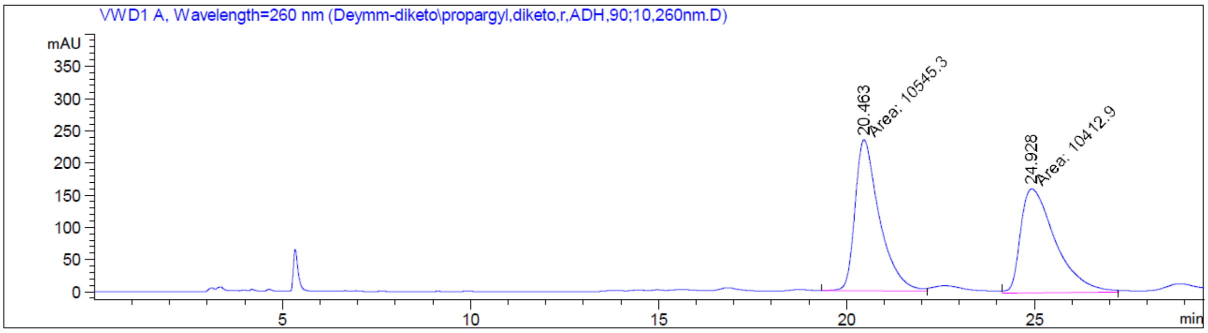

Signal 1: VWD1 A, Wavelength=260 nm

Peak #	RetTime [min]	Type	Width [min]	Area [mAU*s]	Height [mAU]	Area %
1	20.463	MF	0.7505	1.05453e4	234.17033	50.3161
2	24.928	MM	1.0752	1.04129e4	161.40524	49.6839

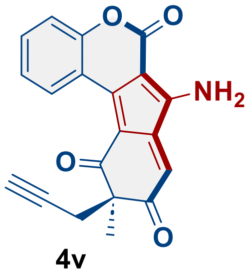

4v

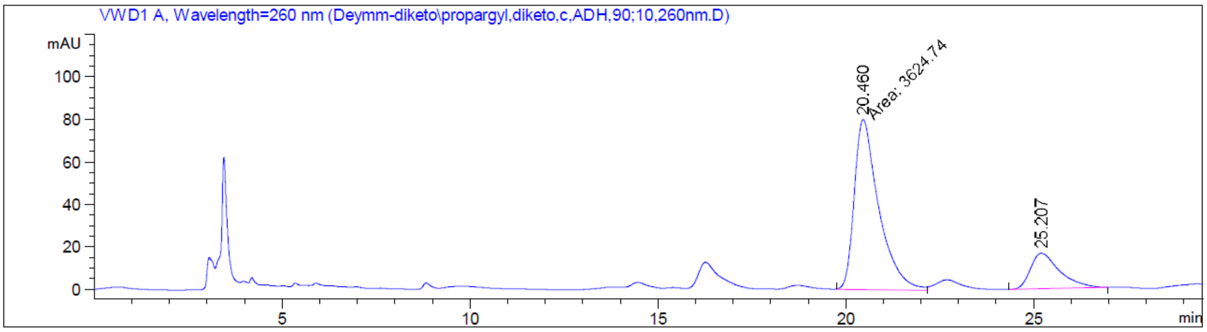

Signal 1: VWD1 A, Wavelength=260 nm

Peak #	RetTime [min]	Type	Width [min]	Area [mAU*s]	Height [mAU]	Area %
1	20.460	MF	0.7576	3624.74341	79.73808	80.0726
2	25.207	BB	0.8083	902.07977	16.72497	19.9274

Supplementary Fig. 167. HPLC traces of rac-**4w** (top) and chiral-**4w** (bottom).

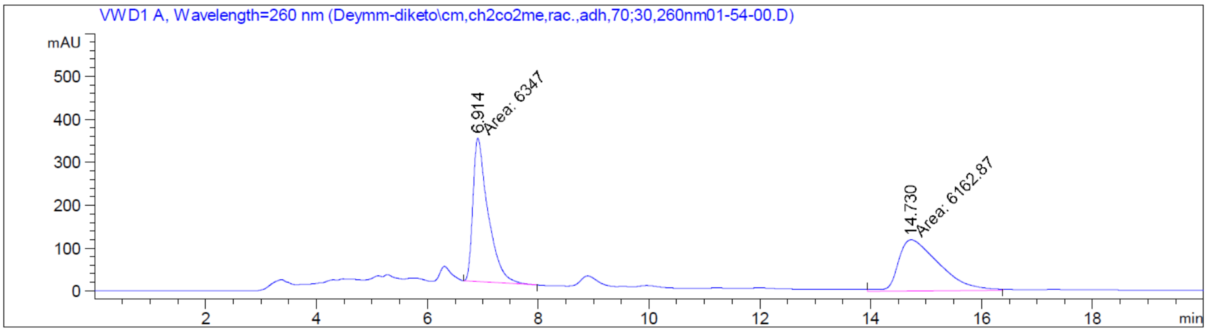

Signal 1: VWD1 A, Wavelength=260 nm

Peak #	RetTime [min]	Type	Width [min]	Area [mAU*s]	Height [mAU]	Area %
1	6.914	MF	0.3162	6347.00000	334.52164	50.7359
2	14.730	MF	0.8572	6162.87256	119.83178	49.2641

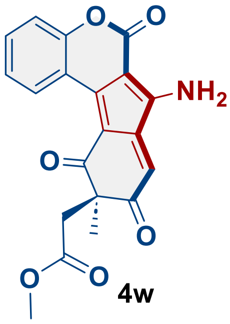

4w

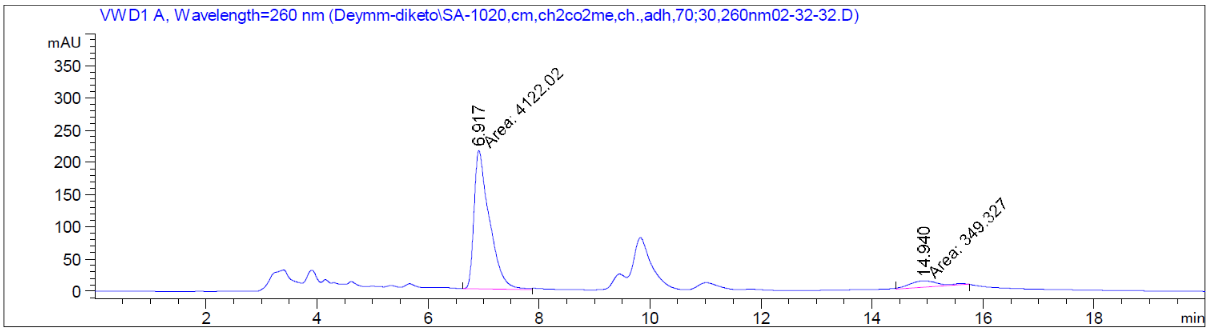

Signal 1: VWD1 A, Wavelength=260 nm

Peak #	RetTime [min]	Type	Width [min]	Area [mAU*s]	Height [mAU]	Area %
1	6.917	MF	0.3201	4122.02393	214.59117	92.1874
2	14.940	MM	0.6016	349.32697	9.67733	7.8126

Supplementary Fig. 168. HPLC traces of rac-**4x** (top) and chiral-**4x** (bottom).

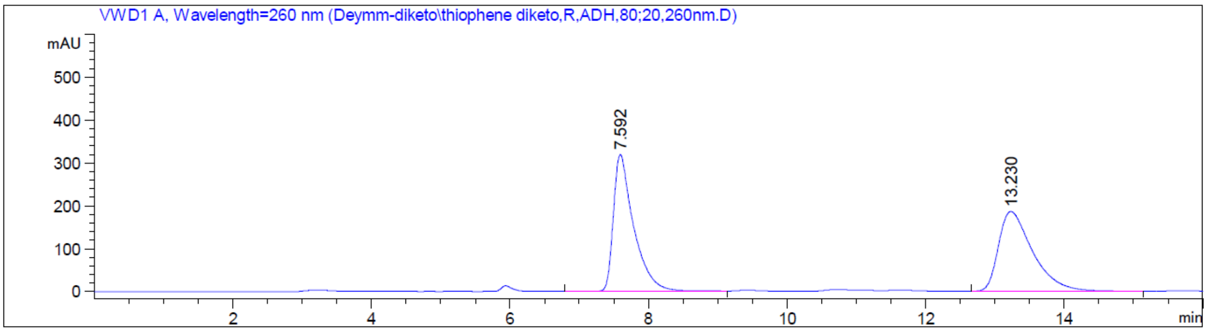

Signal 1: VWD1 A, Wavelength=260 nm

Peak #	RetTime [min]	Type	Width [min]	Area [mAU*s]	Height [mAU]	Area %
1	7.592	BV R	0.2929	6462.62158	318.25107	50.4990
2	13.230	BB	0.5138	6334.89111	185.30574	49.5010

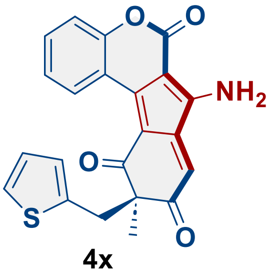

4x

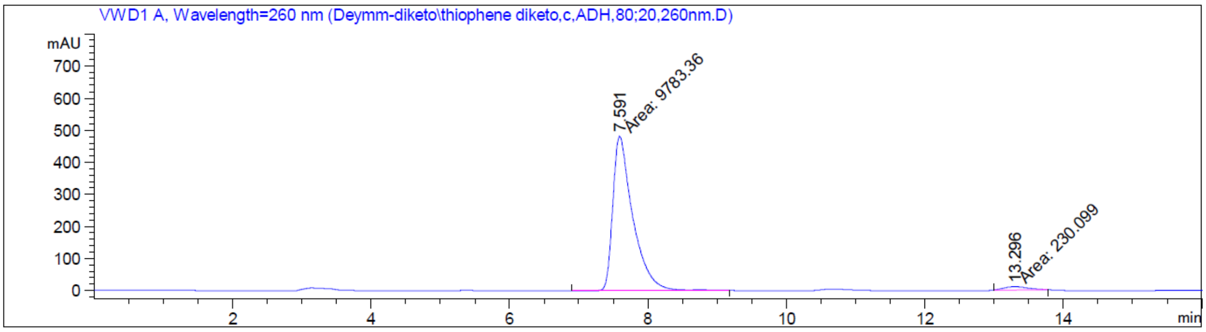

Signal 1: VWD1 A, Wavelength=260 nm

Peak #	RetTime [min]	Type	Width [min]	Area [mAU*s]	Height [mAU]	Area %
1	7.591	MM	0.3387	9783.36133	481.37302	97.7021
2	13.296	MM	0.3970	230.09947	9.66086	2.2979

Supplementary Fig. 169. HPLC traces of rac-**4z** (top) and chiral-**4z** (bottom).

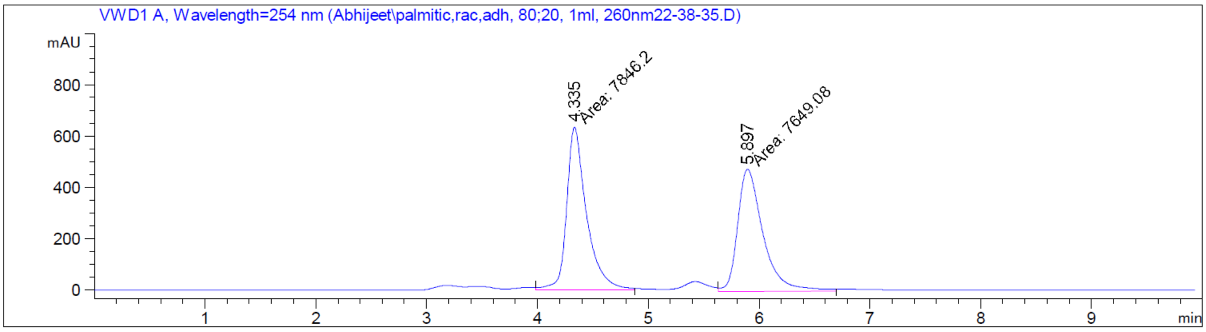

Signal 1: VWD1 A, Wavelength=254 nm

Peak #	RetTime [min]	Type	Width [min]	Area [mAU*s]	Height [mAU]	Area %
1	4.335	MF	0.2071	7846.20313	631.53131	50.6361
2	5.897	MF	0.2677	7649.07764	476.30072	49.3639

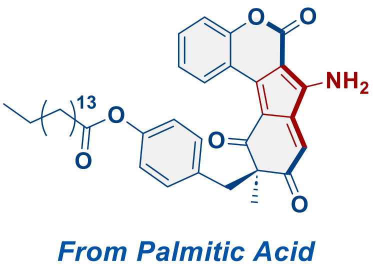

4z

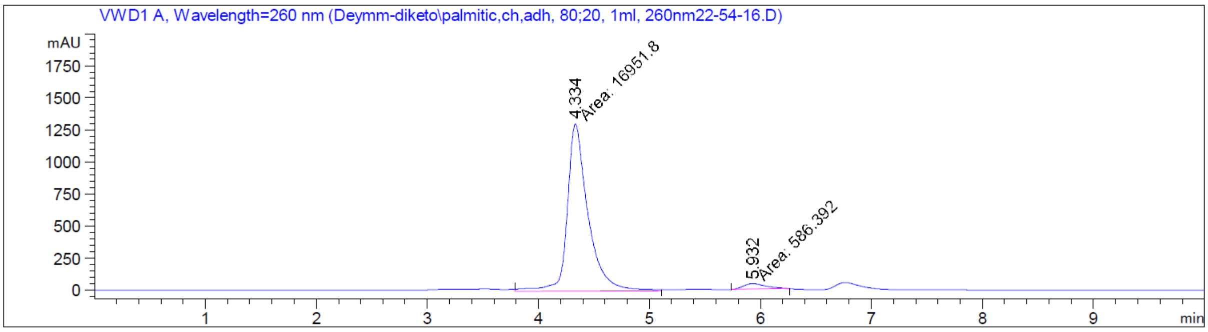

Signal 1: VWD1 A, Wavelength=260 nm

Peak #	RetTime [min]	Type	Width [min]	Area [mAU*s]	Height [mAU]	Area %
1	4.334	MM	0.2172	1.69518e4	1300.96375	96.6565
2	5.932	MM	0.2310	586.39197	42.30604	3.3435

Supplementary Fig. 170. HPLC traces of rac-**4aa** (top) and chiral-**4aa** (bottom).

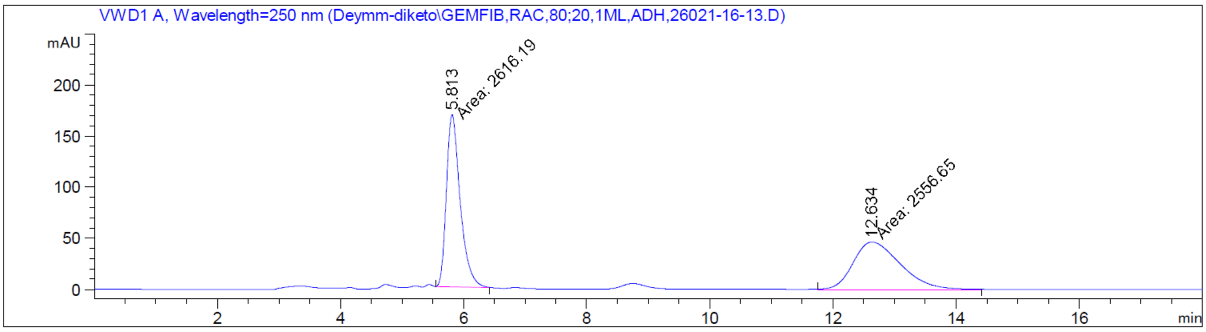

Signal 1: VWD1 A, Wavelength=250 nm

Peak #	RetTime [min]	Type	Width [min]	Area [mAU*s]	Height [mAU]	Area %
1	5.813	MM	0.2589	2616.19214	168.44670	50.5755
2	12.634	MM	0.9092	2556.64771	46.86639	49.4245

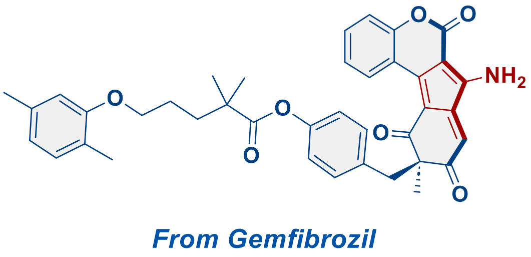

4aa

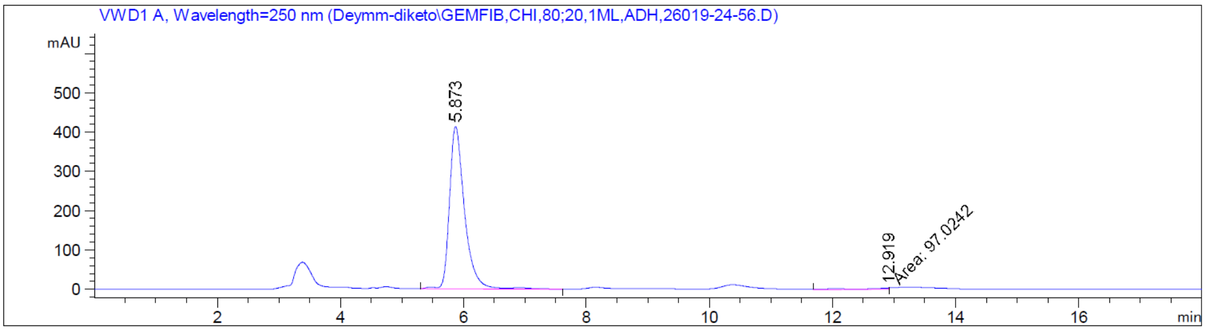

Signal 1: VWD1 A, Wavelength=250 nm

Peak #	RetTime [min]	Type	Width [min]	Area [mAU*s]	Height [mAU]	Area %
1	5.873	VV R	0.2509	7000.06641	412.74030	98.6329
2	12.919	MM	0.4856	97.02418	3.33026	1.3671

Supplementary Fig. 171. HPLC traces of rac-**4ab** (top) and chiral-**4ab** (bottom)**.**

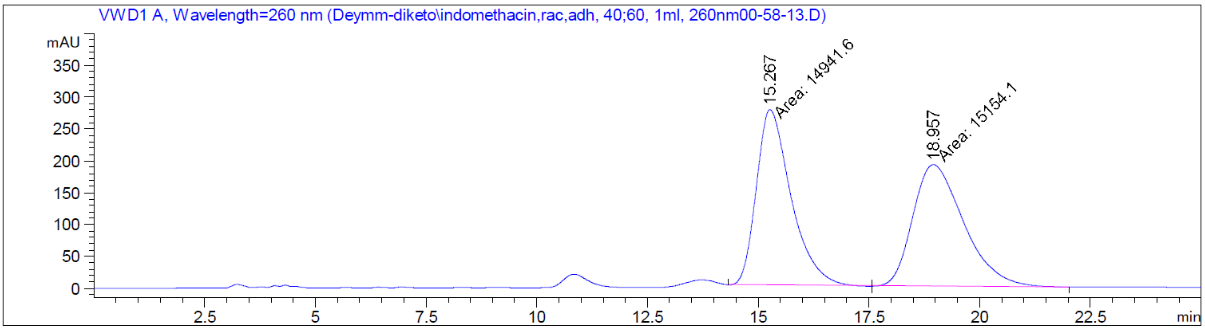

Signal 1: VWD1 A, Wavelength=260 nm

Peak #	RetTime [min]	Type	Width [min]	Area [mAU*s]	Height [mAU]	Area %
1	15.267	MF	0.9046	1.49416e4	275.28452	49.6470
2	18.957	FM	1.3271	1.51541e4	190.30971	50.3530

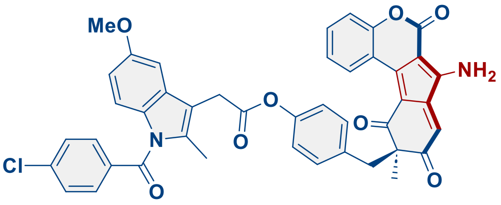

From Indomethacin

4ab

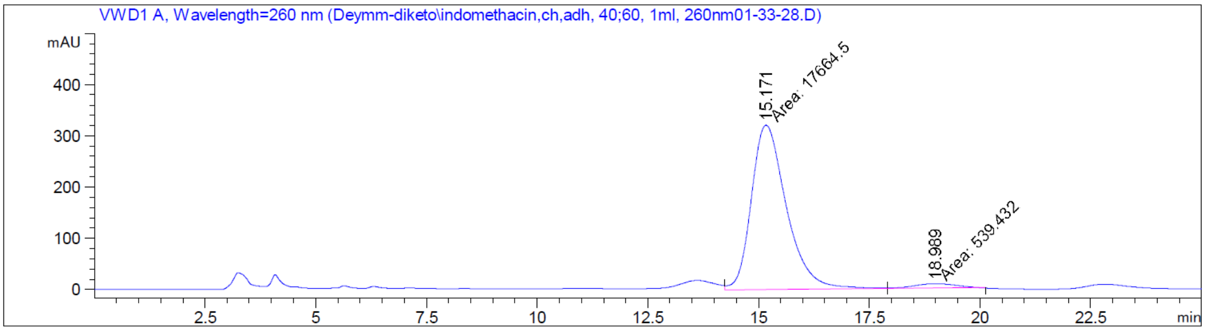

Signal 1: VWD1 A, Wavelength=260 nm

Peak #	RetTime [min]	Type	Width [min]	Area [mAU*s]	Height [mAU]	Area %
1	15.171	MF	0.9182	1.76645e4	320.62735	97.0367
2	18.989	FM	1.0794	539.43170	8.32934	2.9633

Supplementary Fig. 172. HPLC traces of rac-**4ac** (top) and chiral-**4ac** (bottom).

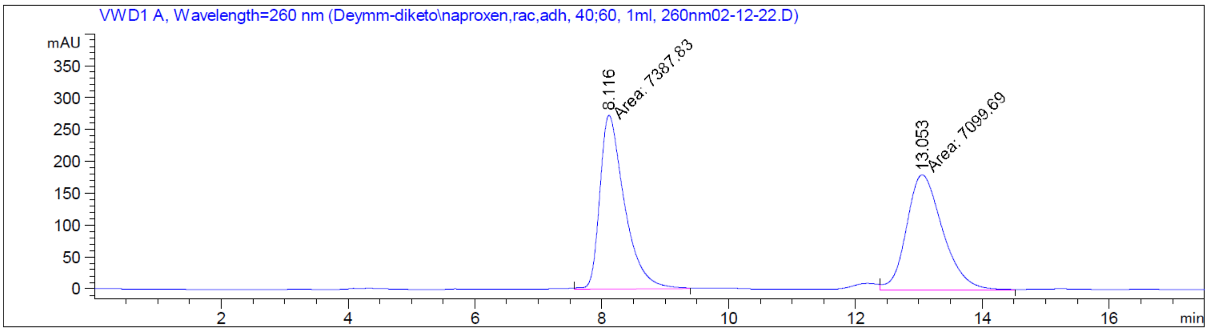

Signal 1: VWD1 A, Wavelength=260 nm

Peak #	RetTime [min]	Type	Width [min]	Area [mAU*s]	Height [mAU]	Area %
1	8.116	FM	0.4521	7387.83252	272.35989	50.9944
2	13.053	MF	0.6576	7099.69336	179.92627	49.0056

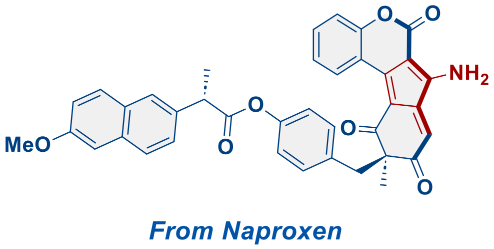

4ac

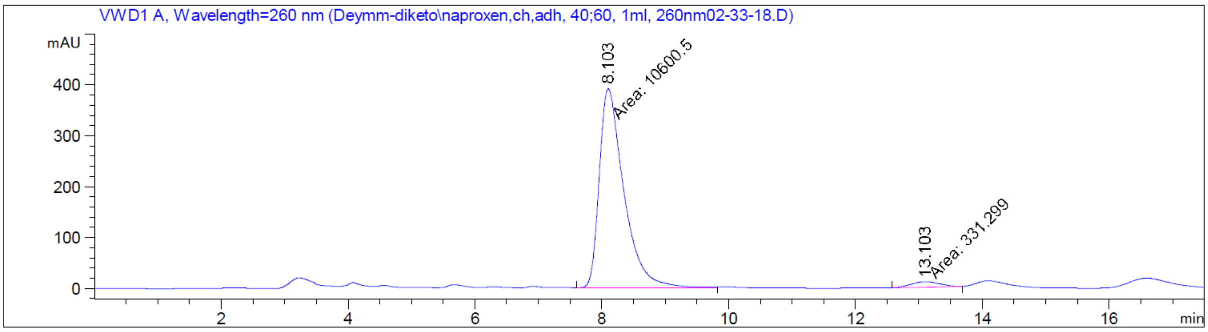

Signal 1: VWD1 A, Wavelength=260 nm

Peak #	RetTime [min]	Type	Width [min]	Area [mAU*s]	Height [mAU]	Area %
1	8.103	MF	0.4516	1.06005e4	391.22693	96.9694
2	13.103	MM	0.5242	331.29886	10.53351	3.0306

Supplementary Fig. 173. HPLC traces of rac-**5** (top) and chiral-**5** (bottom).

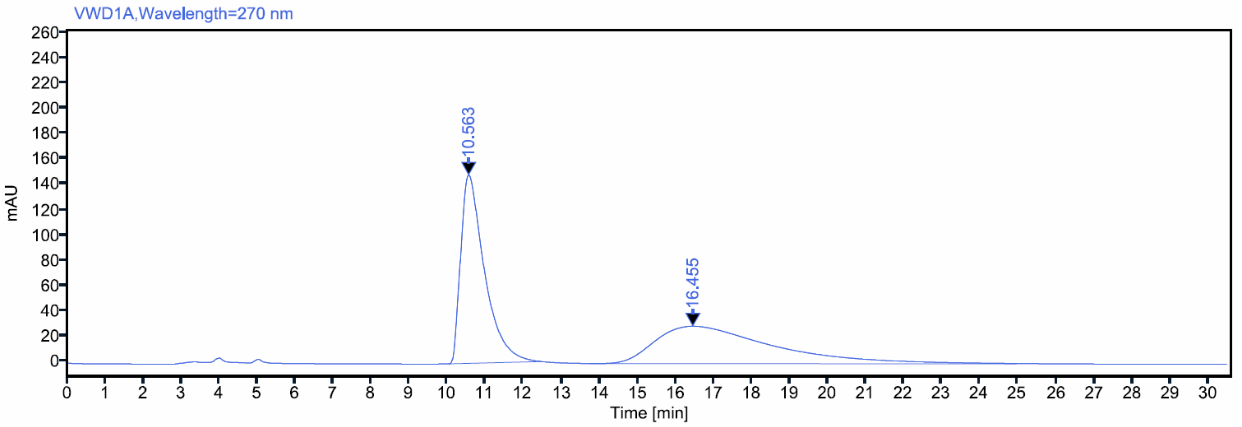

Signal: VWD1A,Wavelength=270 nm

RT [min]	Width [min]	Area	Height	Area%
10.563	2.43	6490.42	149.52	50.38
16.455	11.21	6392.39	29.69	49.62
	Sum	12882.80		

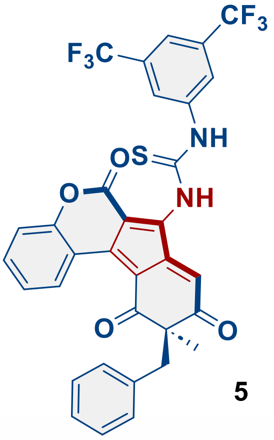

5

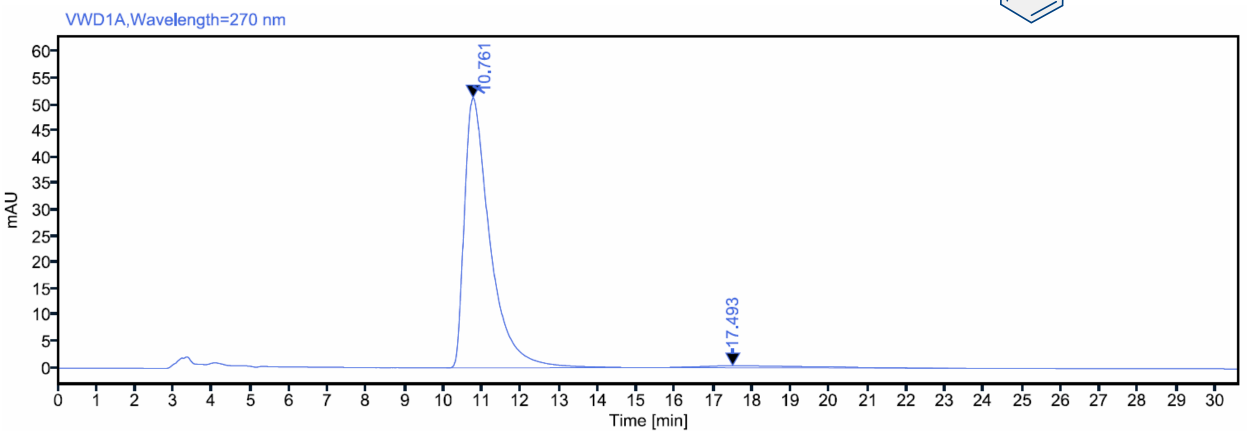

Signal: VWD1A,Wavelength=270 nm

RT [min]	Width [min]	Area	Height	Area%
10.761	5.11	2369.91	51.23	96.69
17.493	7.59	81.15	0.37	3.31
	Sum	2451.06		

Supplementary Fig. 174. HPLC traces of rac-**6** (top) and chiral-**6** (bottom).

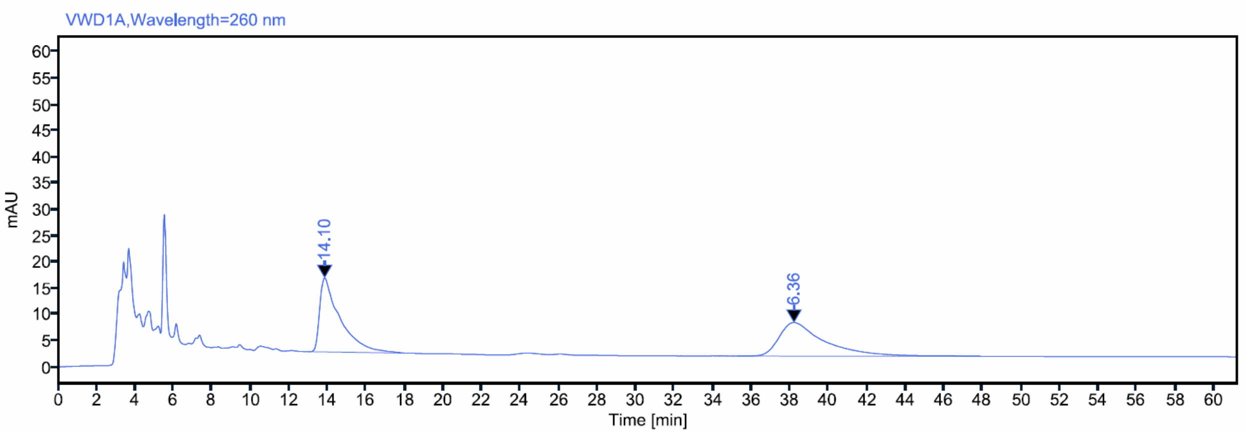

Signal: VWD1A,Wavelength=260 nm

RT [min]	Width [min]	Area	Height	Area%
13.826	5.13	1037.51	14.10	49.93
38.197	13.42	1040.58	6.36	50.07
	Sum	2078.10		

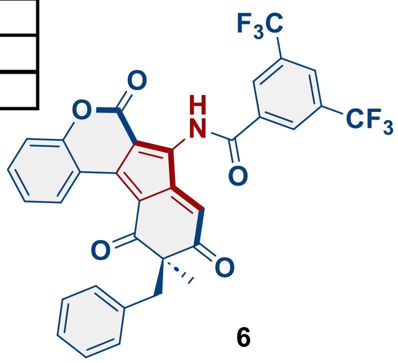

6

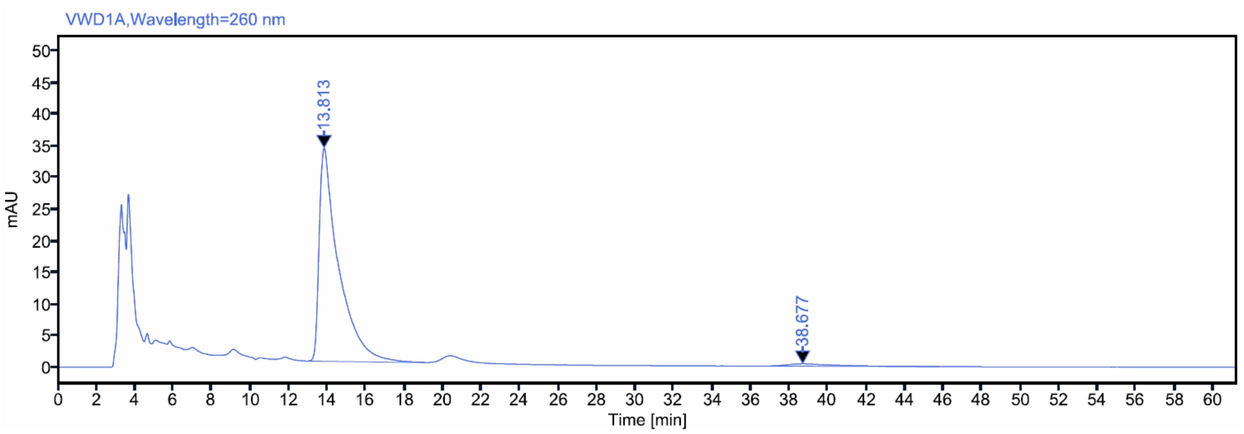

Signal: VWD1A,Wavelength=260 nm

RT [min]	Width [min]	Area	Height	Area%
13.813	6.12	2359.56	33.76	97.27
38.677	9.59	66.26	0.37	2.73
	Sum	2425.82		

Supplementary Fig. 175. HPLC traces of rac-**7** (top) and chiral-**7** (bottom).

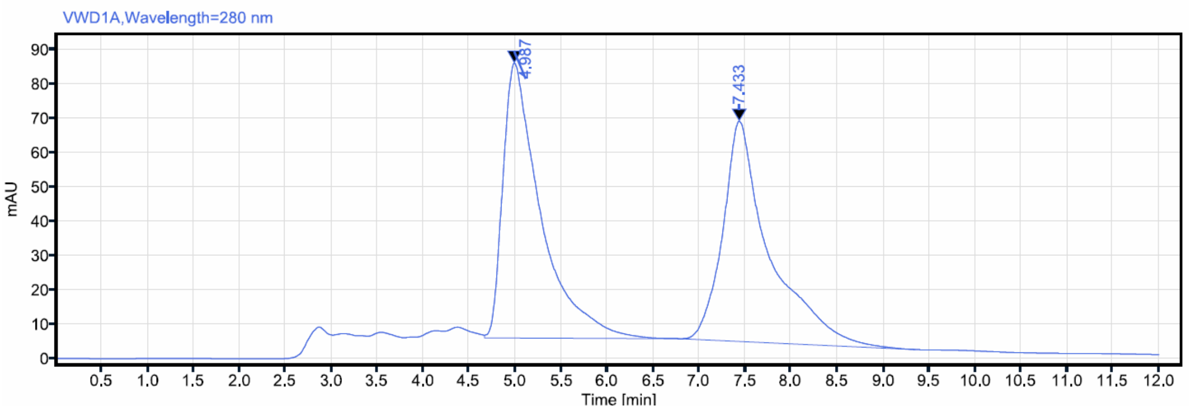

Signal: VWD1A,Wavelength=280 nm

RT [min]	Width [min]	Area	Height	Area%
4.987	2.13	2298.04	80.36	50.37
7.433	2.54	2263.95	64.37	49.63
	Sum	4561.99		

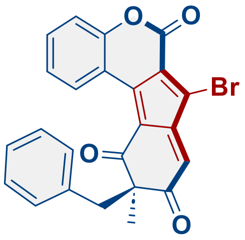

7

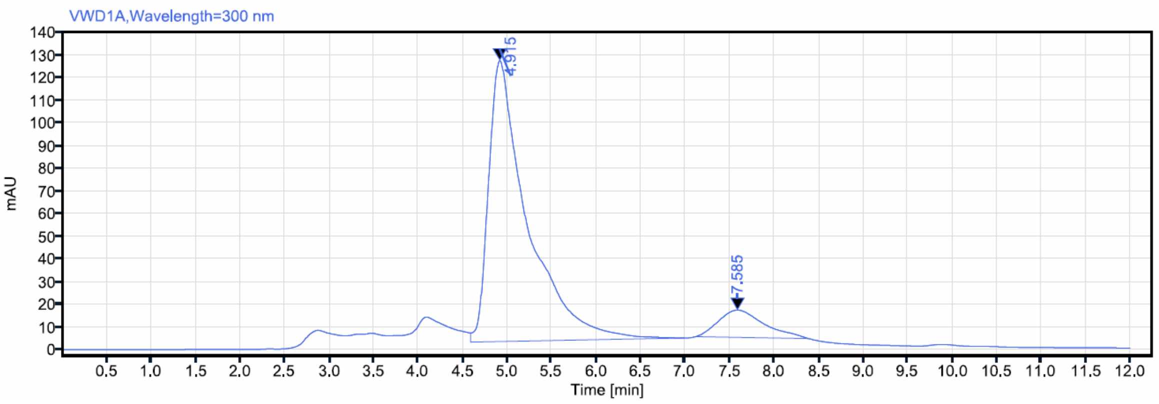

Signal: VWD1A,Wavelength=300 nm

RT [min]	Width [min]	Area	Height	Area%
4.915	2.40	3767.31	124.12	89.81
7.585	1.26	427.41	12.01	10.19
	Sum	4194.72		

Supplementary Fig. 176. HPLC traces of rac-**8**(top) and chiral-**8** (bottom).

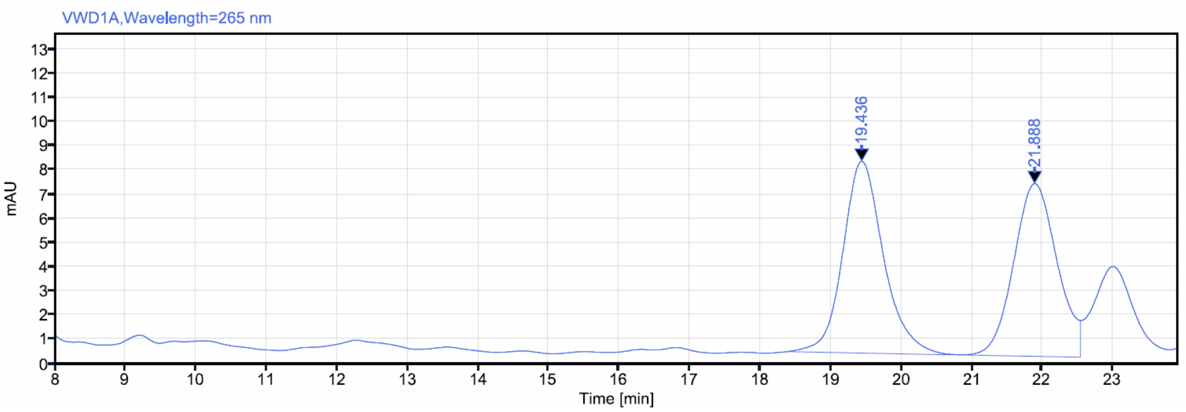

Signal: VWD1A,Wavelength=265 nm

RT [min]	Width [min]	Area	Height	Area%
19.436	2.23	317.39	7.93	50.99
21.888	1.93	305.09	7.14	49.01
	Sum	622.47		

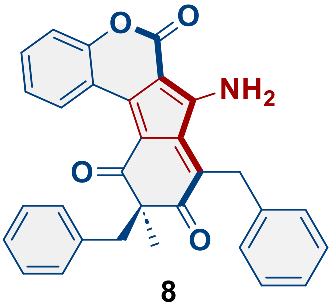

8

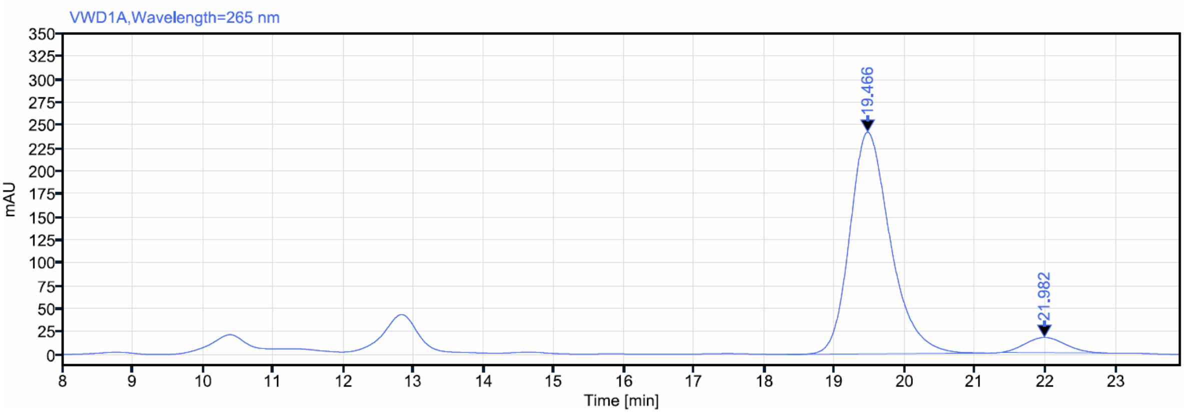

Signal: VWD1A,Wavelength=265 nm

RT [min]	Width [min]	Area	Height	Area%
19.466	2.90	9681.58	241.59	93.63
21.982	1.50	658.32	16.59	6.37
	Sum	10339.90		

Supplementary Fig. 177. HPLC traces of rac-**4aa** (top) and chiral-**4aa** (bottom)**.**

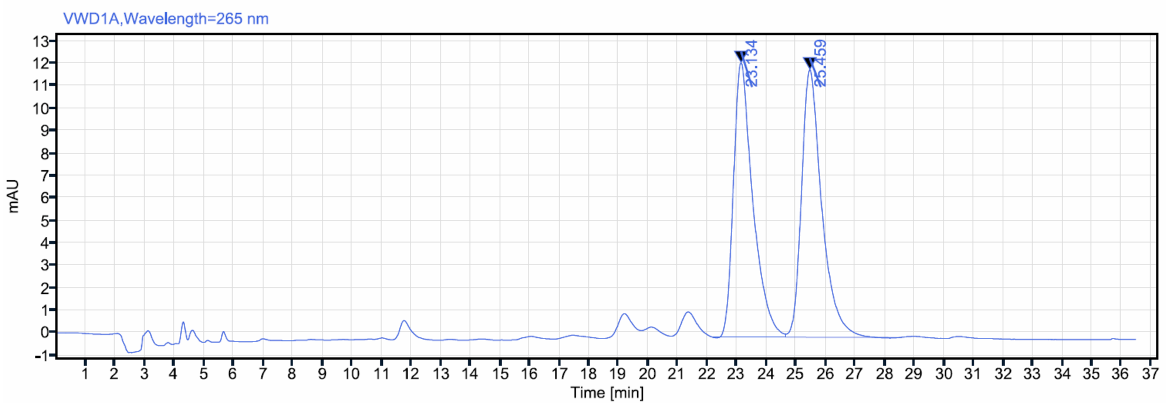

Signal: VWD1A,Wavelength=265 nm

RT [min]	Width [min]	Area	Height	Area%
23.134	2.46	557.14	12.21	49.60
25.459	3.92	566.08	11.92	50.40
	Sum	1123.23		

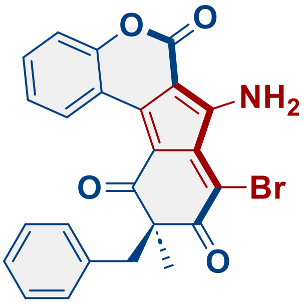

9

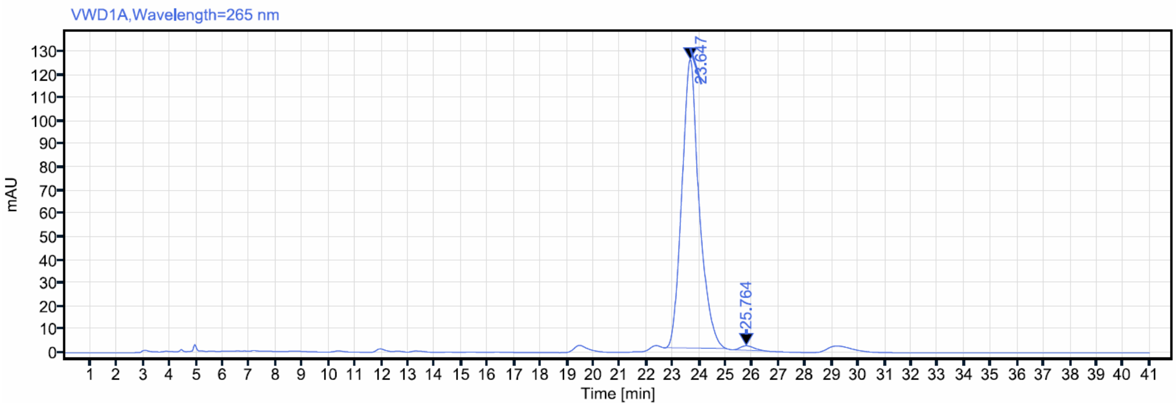

Signal: VWD1A,Wavelength=265 nm

RT [min]	Width [min]	Area	Height	Area%
23.647	2.37	5667.57	124.39	98.82
25.764	1.19	67.47	1.91	1.18
	Sum	5735.04		

8. HPLC Chromatograms of selected compounds obtained at 10 mol% catalyst loading

Supplementary Fig. 178. HPLC traces of rac-**3j** (top) and chiral-**3j** (bottom)**.**

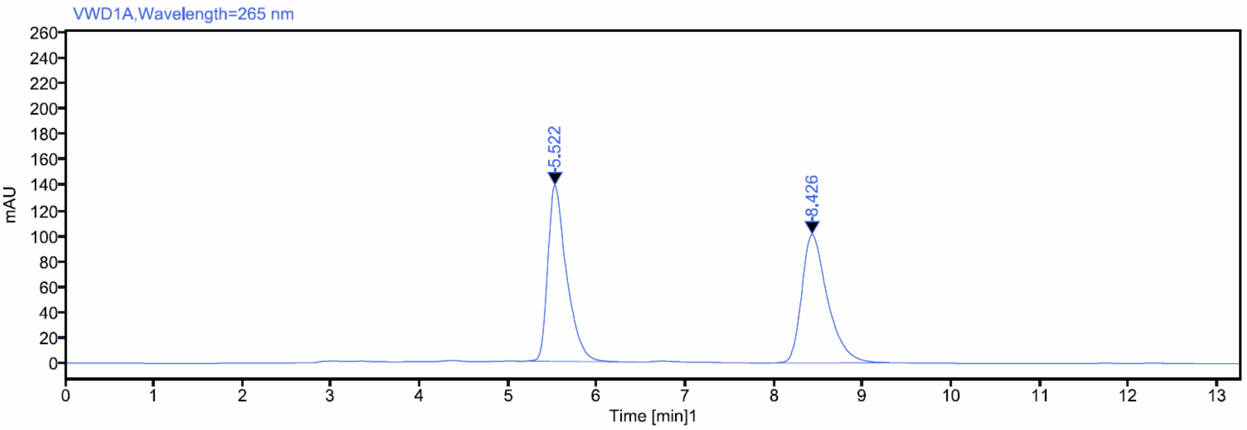

Signal: VWD1A,Wavelength=265 nm

RT [min]	Width [min]	Area	Height	Area%
5.522	1.39	2047.78	138.93	49.83
8.426	1.58	2061.46	101.47	50.17
	Sum	4109.25		

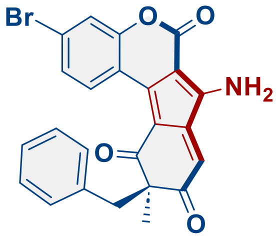

3j

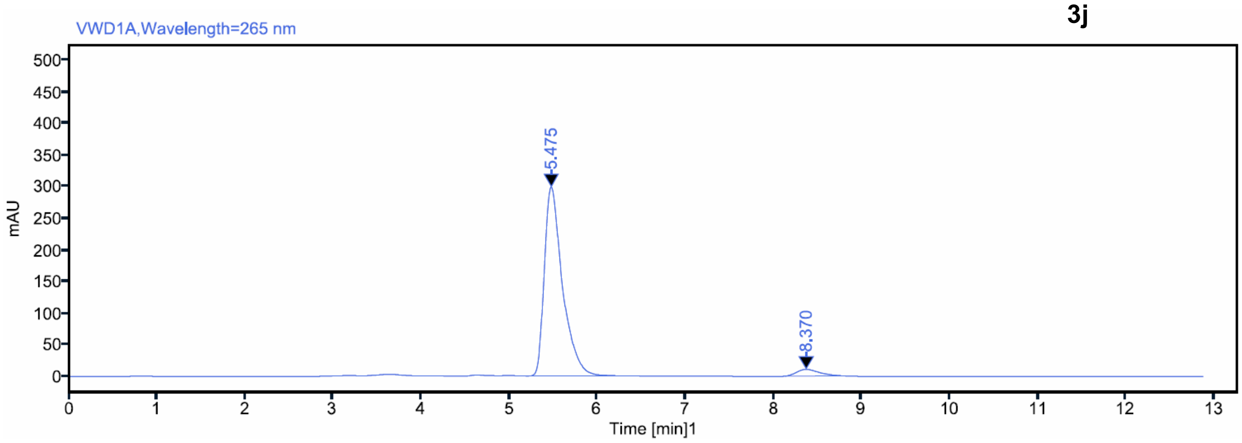

Signal: VWD1A,Wavelength=265 nm

RT [min]	Width [min]	Area	Height	Area%
5.475	1.12	4263.45	299.36	95.77
8.370	0.66	188.26	10.63	4.23
	Sum	4451.71		

Supplementary Fig. 179. HPLC traces of rac-**4a** (top) and chiral-**4a** (bottom).

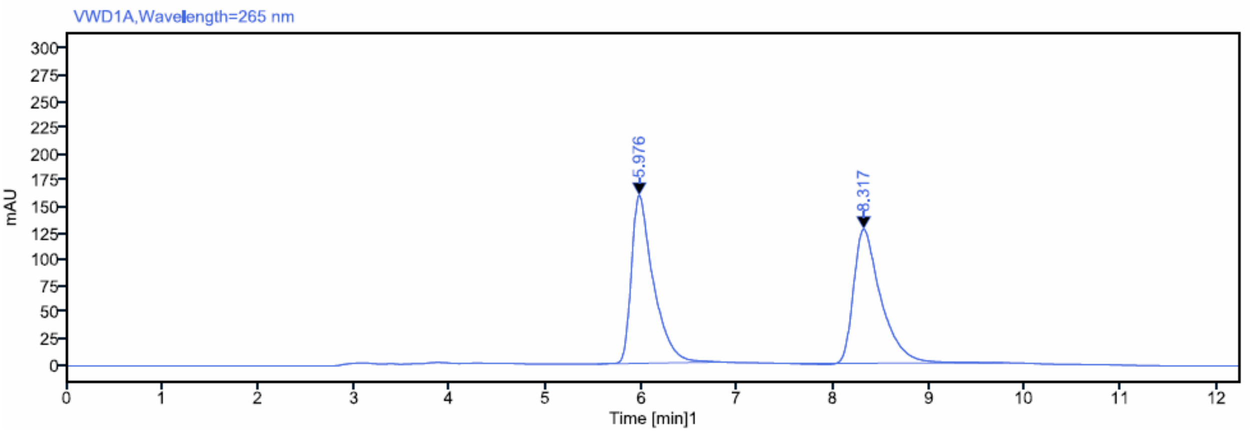

Signal: VWD1A,Wavelength=265 nm

RT [min]	Width [min]	Area	Height	Area%
5,976	1,32	2537,05	159,17	50,22
8,317	2,19	2514,59	126,96	49,78
	Sum	5051,64		

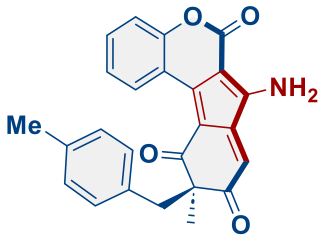

4a

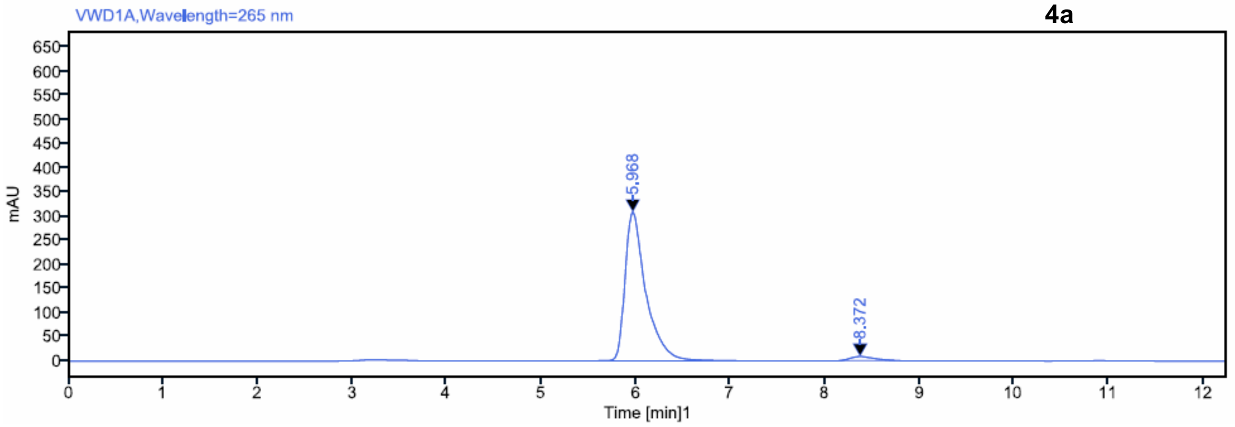

Signal: VWD1A,Wavelength=265 nm

RT [min]	Width [min]	Area	Height	Area%
5,968	1,93	4732,27	308,81	97,03
8,372	0,62	145,04	8,68	2,97
	Sum	4877,31		

Supplementary Fig. 180. HPLC traces of rac-**4m** (top) and chiral-**4m** (bottom).

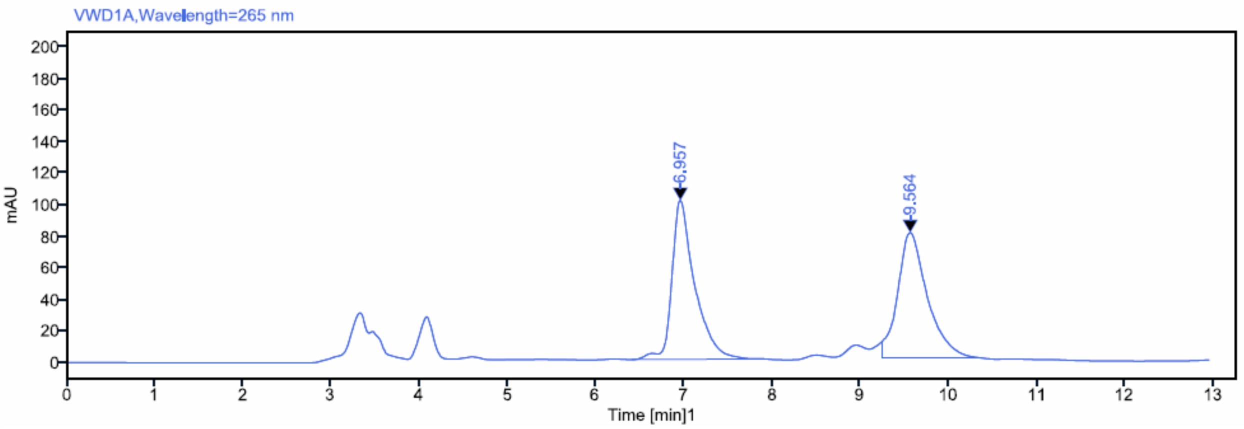

Signal: VWD1A,Wavelength=265 nm

RT [min]	Width [min]	Area	Height	Area%
6.957	1.55	1813.27	100.79	49.29
9.564	1.12	1865.16	79.51	50.71
	Sum	3678.43		

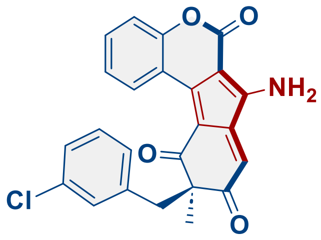

4m

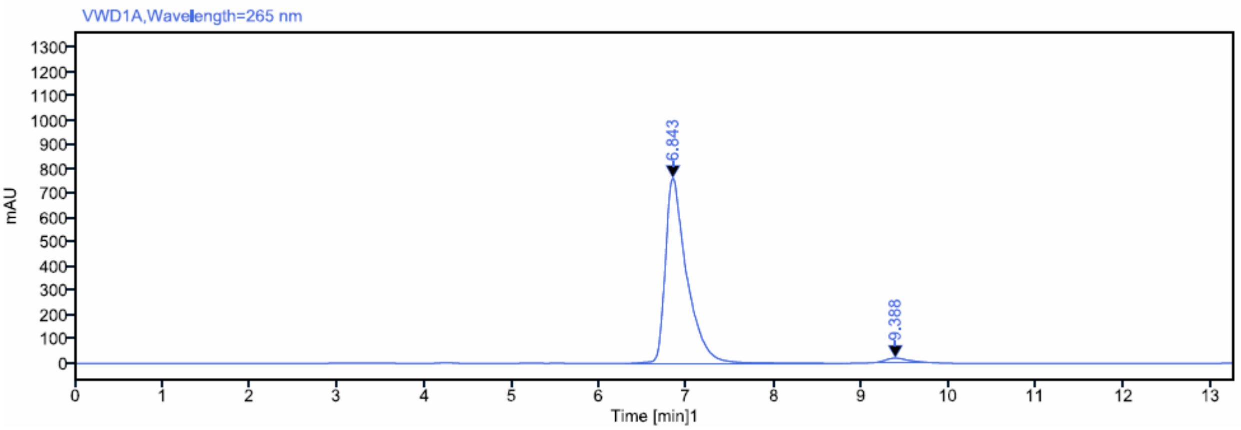

Signal: VWD1A,Wavelength=265 nm

RT [min]	Width [min]	Area	Height	Area%
6.843	2.47	12987.54	762.16	97.27
9.388	0.75	365.03	19.03	2.73
	Sum	13352.57		

Supplementary Fig. 181. HPLC traces of rac-**3o** (top) and chiral-**3o** (bottom)**.**

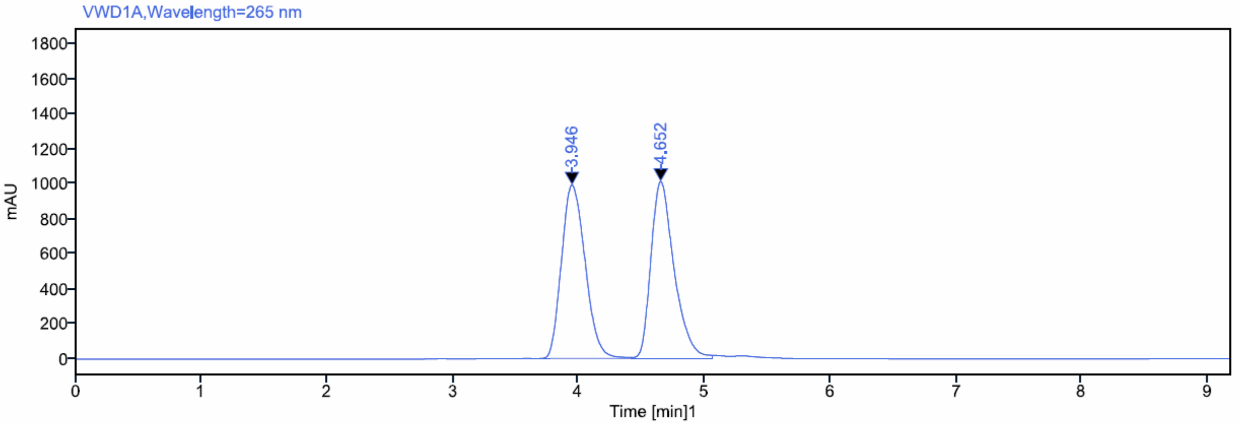

Signal: VWD1A,Wavelength=265 nm

RT [min]	Width [min]	Area	Height	Area%
3.946	0.76	12987.85	991.83	50.06
4.652	0.65	12955.05	1011.22	49.94
	Sum	25942.89		

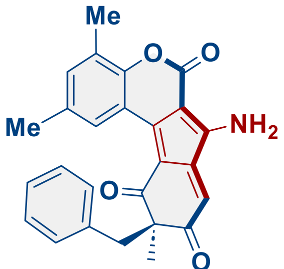

3o

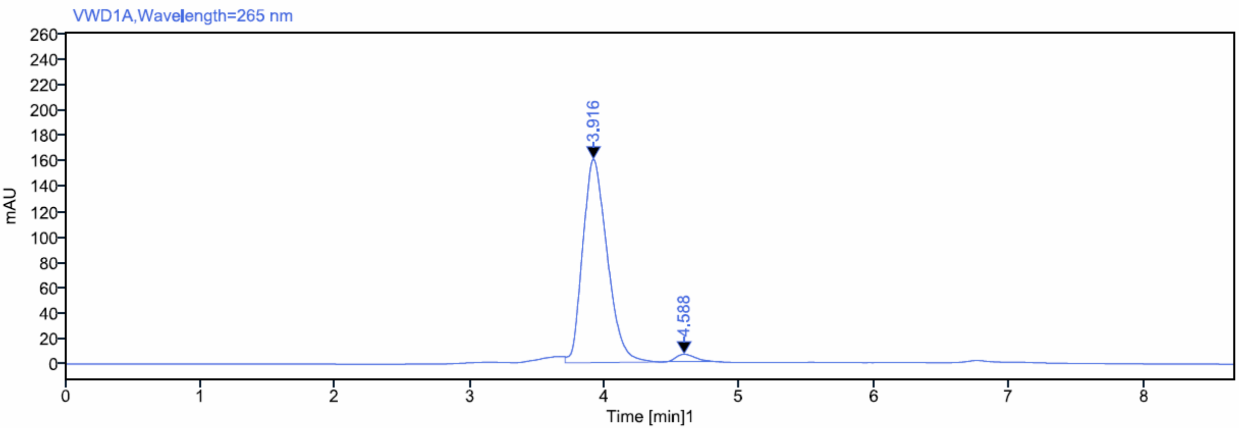

Signal: VWD1A,Wavelength=265 nm

RT [min]	Width [min]	Area	Height	Area%
3.916	0.73	2037.17	160.85	96.95
4.588	0.42	64.17	6.07	3.05
	Sum	2101.35		

Supplementary Fig. 182. HPLC traces of rac-**3s** (top) and chiral-**3s** (bottom).

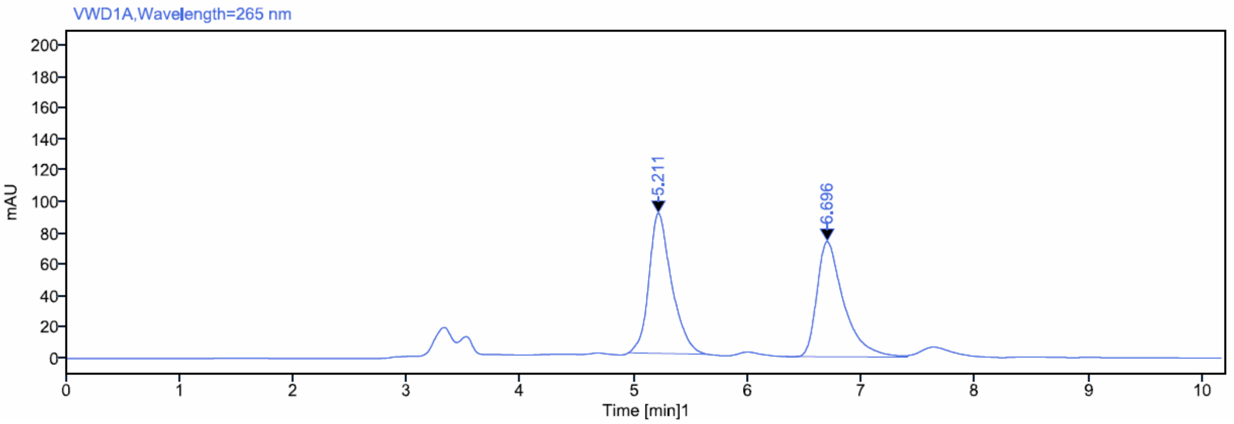

Signal: VWD1A,Wavelength=265 nm

RT [min]	Width [min]	Area	Height	Area%
5.211	0.69	1242.57	89.58	50.63
6.696	1.07	1211.64	73.78	49.37
	Sum	2454.20		

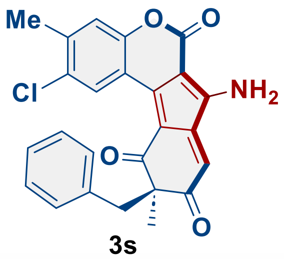

3s

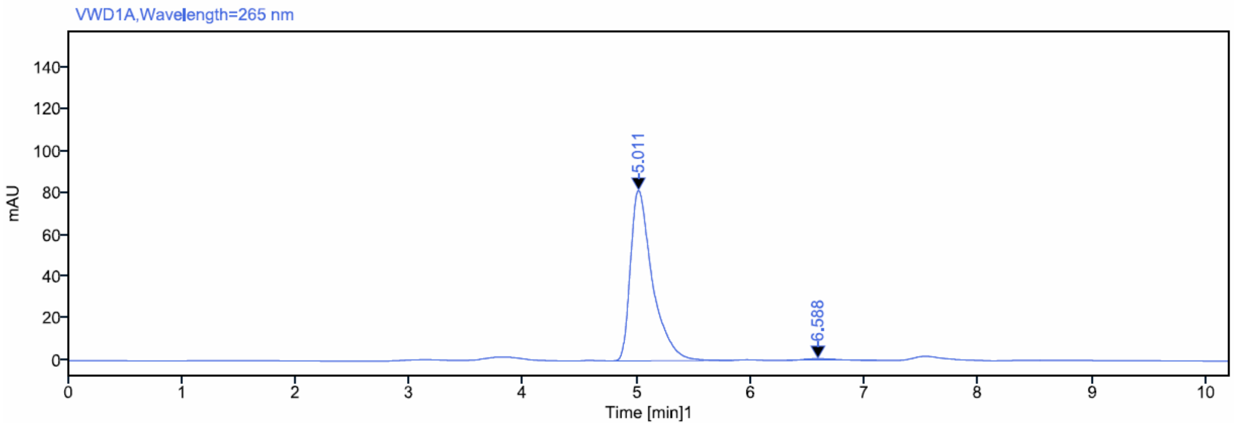

Signal: VWD1A,Wavelength=265 nm

RT [min]	Width [min]	Area	Height	Area%
5.011	1.05	1089.83	81.39	98.42
6.588	0.78	17.52	0.84	1.58
	Sum	1107.35		

Supplementary Fig. 183. HPLC traces of rac-**4i** (top) and chiral-**4i** (bottom).

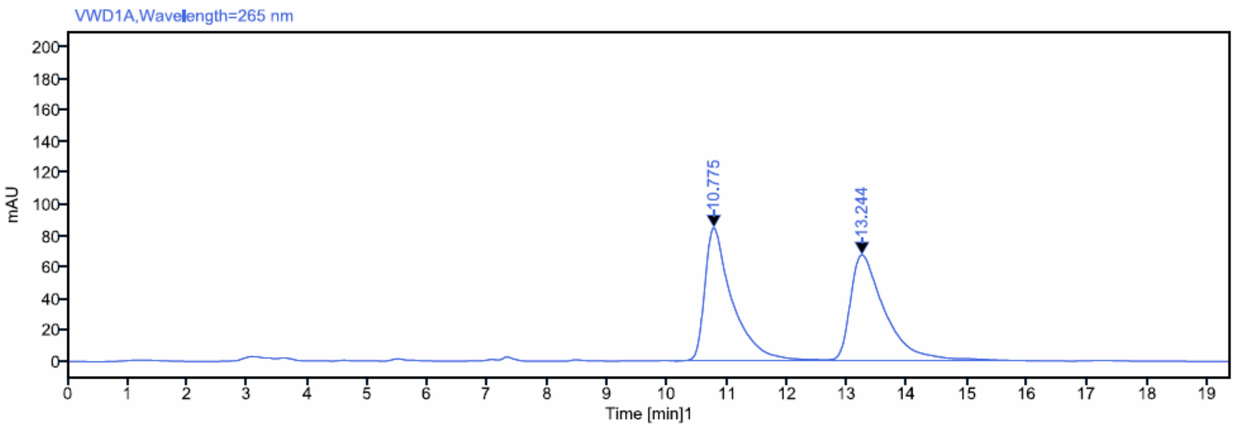

Signal: VWD1A,Wavelength=265 nm

RT [min]	Width [min]	Area	Height	Area%
10.775	2.47	2703.17	84.89	49.84
13.244	3.59	2720.33	67.60	50.16
	Sum	5423.51		

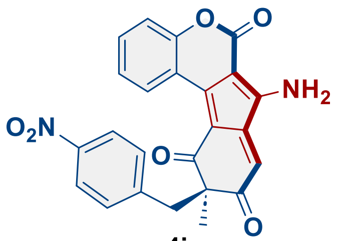

4i

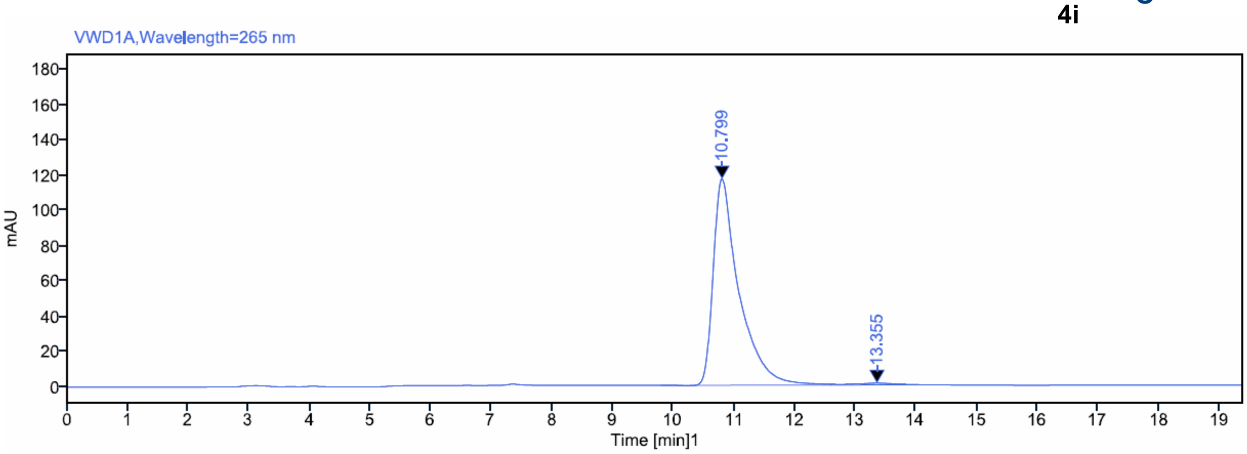

Signal: VWD1A,Wavelength=265 nm

RT [min]	Width [min]	Area	Height	Area%
10.799	2.81	3452.70	117.03	98.59
13.355	1.71	49.35	1.03	1.41
	Sum	3502.05		

Supplementary Fig. 184. HPLC traces of rac-**4o** (top) and chiral-**4o** (bottom)**.**

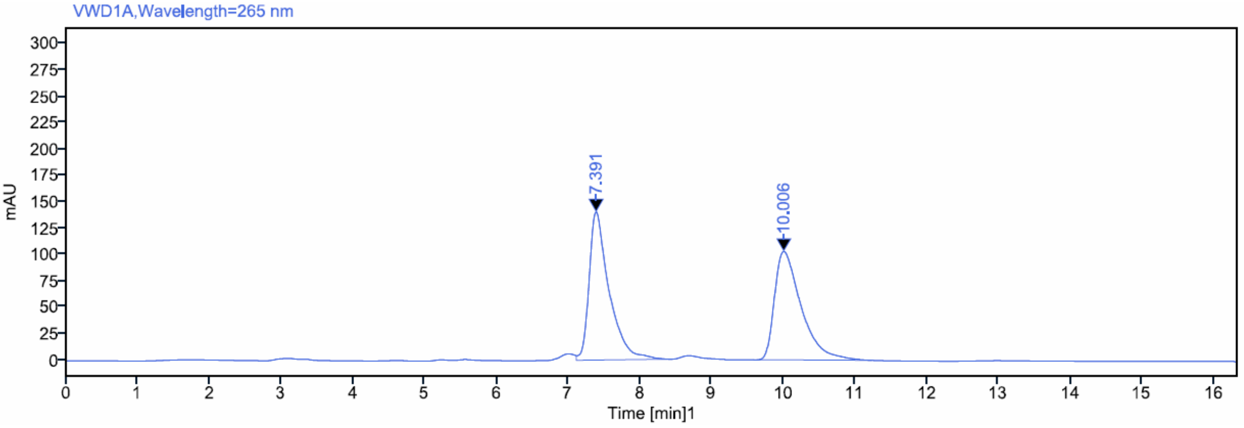

Signal: VWD1A,Wavelength=265 nm

RT [min]	Width [min]	Area	Height	Area%
7.391	1.35	2712.46	140.22	50.45
10.006	1.64	2664.20	102.63	49.55
	Sum	5376.66		

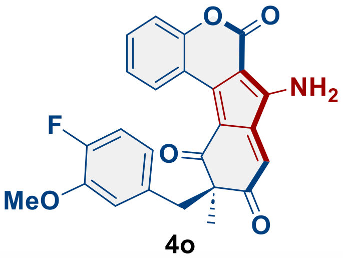

4o

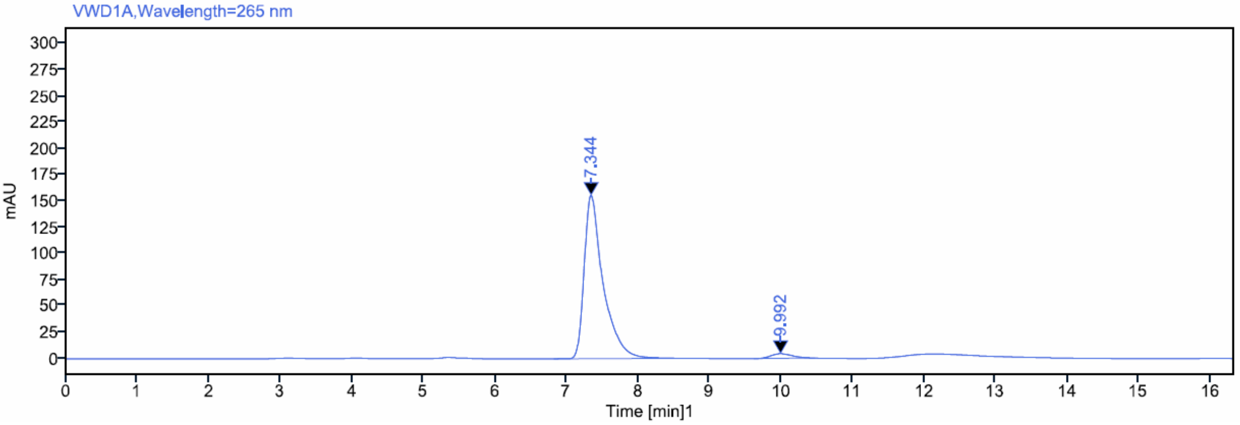

Signal: VWD1A,Wavelength=265 nm

RT [min]	Width [min]	Area	Height	Area%
7.344	1.70	2853.38	155.31	96.91
9.992	0.85	91.11	4.39	3.09
	Sum	2944.50		

9. Crystal data and ORTEP diagram for compound 3a (CCDC 2210447).

Bond precision: C-C = 0.0053 A Wavelength=0.71073

Cell:	a=5.717(2)	b=11.695(2)	c=27.553(6)
	alpha=90	beta=90.00(3)	gamma=90

Temperature: 303 K

	Calculated	Reported
Volume	1842.2(8)	1842.3(8)
Space group	P 21	P 21
Hall group	P 2yb	P 2yb
Moiety formula	C24 H17 N O4	C24 H17 N O4
Sum formula	C24 H17 N O4	C24 H17 N O4
Mr	383.39	383.39
Dx,g cm-3	1.382	1.382
Z	4	4
Mu (mm-1)	0.095	0.095
F000	800.0	800.0
F000'	800.40	
h,k,lmax	6,14,33	6,14,33
Nref	6733[3543]	6733
Tmin,Tmax	0.980,0.985	
Tmin'	0.977	

Correction method= Not given

Data completeness= 1.90/1.00 Theta(max)= 25.386

R(reflections)= 0.0374(4694) wR2(reflections)= 0.0897(6713)

S = 1.057 Npar= 525

_refine_ls_abs_structure_Flack -0.1(4)

_chemical_absolute_configuration '*R*'

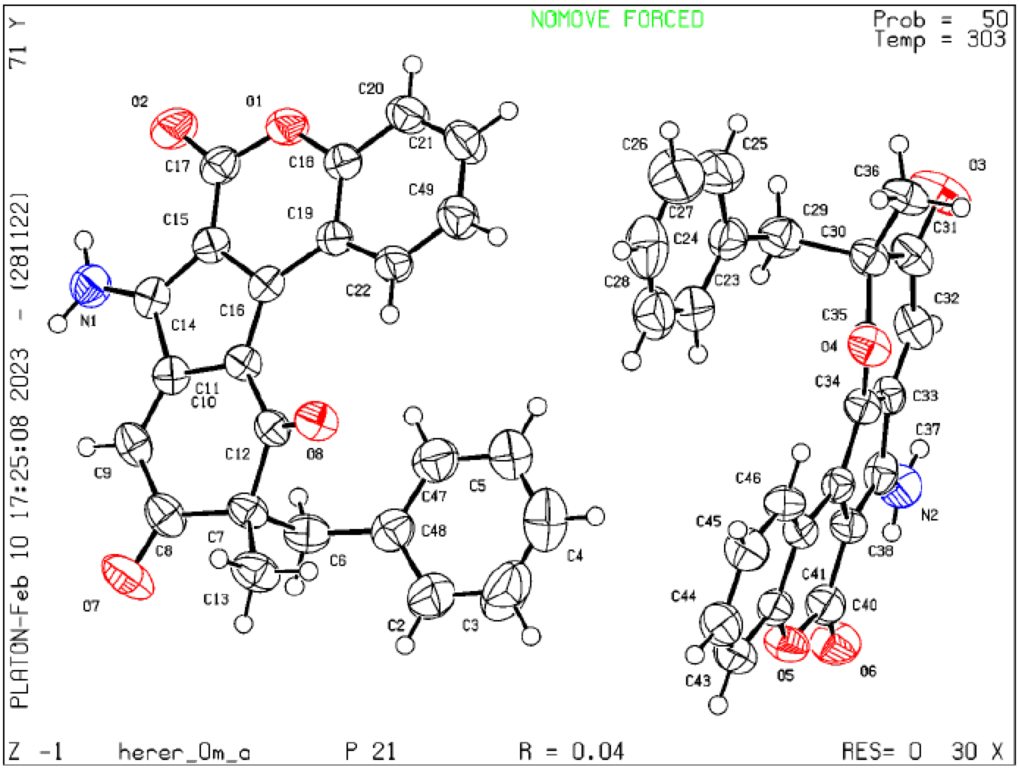

Supplementary Fig. 185. ORTEP drawing of **3a.**

10. Supplementary References

1. Loh, C. C. J., Schmid, M., Peters, B., Fang, X. & Lautens, M. Exploiting Distal Reactivity of Coumarins: A Rhodium-Catalyzed Vinylogous Asymmetric Ring-Opening Reaction. *Angew. Chem., Int. Ed.* **55**, 4600–4604 (2016).

2. Ni, Q., Zhu, Z., Fan, Y., Chen, X. & Song, X. Chiral Phosphoric Acid Catalyzed Desymmetrization of Cyclopentendiones via Friedel–Crafts Conjugate Addition of Indolizines. *Org. Lett.* **23**, 9548–9553 (2021).

3. Manna, M. S. & Mukherjee, S. Remarkable influence of secondary catalyst site on enantioselective desymmetrization of cyclopentenedione. *Chem. Sci.* **5**, 1627–1633 (2014).

4. Maurya, S., Jadhav, S. B. & Chegondi, R. Metal-free oxidative formal C(sp2)–H arylation of cyclopentene-1,3-diones with β-naphthols. *Org. Biomol. Chem.* **20**, 2059–2063 (2022).

5. Vakulya, B., Varga, S., Csámpai, A. & Soós, T. Highly Enantioselective Conjugate Addition of Nitromethane to Chalcones Using Bifunctional Cinchona Organocatalysts. *Org. Lett.* **7**, 1967–1969 (2005).

6. Svestka, D., Otevrel, J. & Bobal, P. Asymmetric Organocatalyzed Friedel–Crafts Reaction of Trihaloacetaldehydes and Phenols. *Adv. Synth. Catal.* **364**, 2174–2183 (2022).
