# Supplementary material for: Desymmetric homologating annulation to access chiral pentafulvenes and their application in bioimaging
Source: Nat Commun. 2024 Mar 7;15:2101. doi: 10.1038/s41467-024-45346-4 (PMC10920648; doi:10.1038/s41467-024-45346-4)
Supplement: Supplementary file 3 — Reporting Summary [file 41467_2024_45346_MOESM3_ESM.pdf]

nature portfolio

Corresponding author(s): Prof. Ravi P Singh

Last updated by author(s): 2023/12/12

Reporting Summary

Nature Portfolio wishes to improve the reproducibility of the work that we publish. This form provides structure for consistency and transparency in reporting. For further information on Nature Portfolio policies, see our Editorial Policies and the Editorial Policy Checklist.

Statistics

For all statistical analyses, confirm that the following items are present in the figure legend, table legend, main text, or Methods section.

n/a	Confirmed	
☐	☒	The exact sample size (*n*) for each experimental group/condition, given as a discrete number and unit of measurement
☐	☒	A statement on whether measurements were taken from distinct samples or whether the same sample was measured repeatedly
☒	☐	The statistical test(s) used AND whether they are one- or two-sided<br>*Only common tests should be described solely by name; describe more complex techniques in the Methods section.*
☒	☐	A description of all covariates tested
☒	☐	A description of any assumptions or corrections, such as tests of normality and adjustment for multiple comparisons
☐	☒	A full description of the statistical parameters including central tendency (e.g. means) or other basic estimates (e.g. regression coefficient) AND variation (e.g. standard deviation) or associated estimates of uncertainty (e.g. confidence intervals)
☒	☐	For null hypothesis testing, the test statistic (e.g. *F*, *t*, *r*) with confidence intervals, effect sizes, degrees of freedom and *P* value noted<br>*Give P values as exact values whenever suitable.*
☒	☐	For Bayesian analysis, information on the choice of priors and Markov chain Monte Carlo settings
☐	☐	For hierarchical and complex designs, identification of the appropriate level for tests and full reporting of outcomes
☒	☐	Estimates of effect sizes (e.g. Cohen's *d*, Pearson's *r*), indicating how they were calculated

Our web collection on statistics for biologists contains articles on many of the points above.

Software and code

Policy information about availability of computer code

Data collection	We used Bruker APEX 4 for single crystal XRD, Agilent OpenLab CDS for HPLC data collection, Bruker Topsin 4.1.3 for NMR data collection, Carl Zeiss Axio Observer microscope for fluorescence imaging, BioTek Synergy H1 Multimode reader for absorption and fluorescence spectra Detailed information regarding all data collection is provided in the Supporting Information.
Data analysis	The fluorescent images were processed using ImageJ, the excitation and emission spectra and MTT data was plotted using the Origin software 2018 from OriginLab. 1H, 13C, 19F NMR spectra were processed using MestReNova 14.1.1-24571 by Mestrelab Research SL, 2019.

For manuscripts utilizing custom algorithms or software that are central to the research but not yet described in published literature, software must be made available to editors and reviewers. We strongly encourage code deposition in a community repository (e.g. GitHub). See the Nature Portfolio guidelines for submitting code & software for further information.

Data

Policy information about availability of data

All manuscripts must include a data availability statement. This statement should provide the following information, where applicable:

- Accession codes, unique identifiers, or web links for publicly available datasets
- A description of any restrictions on data availability
- For clinical datasets or third party data, please ensure that the statement adheres to our policy

Materials and methods, experimental procedures, useful information, mechanistic studies, 1H NMR spectra, 13C NMR spectra, HPLC chromatograms, and mass

spectrometry data are available in Supplementary Information. Raw data is available from the corresponding author upon request. Crystallographic data for compound 3a has been deposited with the Cambridge Crystallographic Data Centre under the accession number CCDC 2210447. These data are provided free of charge from The Cambridge Crystallographic Data Centre via www.ccdc.cam.ac.uk/structures.

Research involving human participants, their data, or biological material

Policy information about studies with human participants or human data. See also policy information about sex, gender (identity/presentation), and sexual orientation and race, ethnicity and racism.

Reporting on sex and gender	N/A
Reporting on race, ethnicity, or other socially relevant groupings	N/A
Population characteristics	N/A
Recruitment	N/A
Ethics oversight	N/A

Note that full information on the approval of the study protocol must also be provided in the manuscript.

Field-specific reporting

Please select the one below that is the best fit for your research. If you are not sure, read the appropriate sections before making your selection.

[x] Life sciences [ ] Behavioural & social sciences [ ] Ecological, evolutionary & environmental sciences

For a reference copy of the document with all sections, see nature.com/documents/nr-reporting-summary-flat.pdf

Life sciences study design

All studies must disclose on these points even when the disclosure is negative.

Sample size	All the experiments were done in triplicates as for MTT studies, 3 samples are reliable and well accepted data for scientific evidence
Data exclusions	No data has been excluded
Replication	The experiments were repeated thrice to confirm the reproducibility of the results and all the attempts were successfull
Randomization	Since these were not clinical studies and we just evaluated the cell viability so this is not relevant to our studies
Blinding	This is not relevant to our studies as these were not clinical studies

Reporting for specific materials, systems and methods

We require information from authors about some types of materials, experimental systems and methods used in many studies. Here, indicate whether each material, system or method listed is relevant to your study. If you are not sure if a list item applies to your research, read the appropriate section before selecting a response.

Materials & experimental systems

n/a	Involved in the study	
[x]	[ ]	Antibodies
[ ]	[x]	Eukaryotic cell lines
[x]	[ ]	Palaeontology and archaeology
[x]	[ ]	Animals and other organisms
[x]	[ ]	Clinical data
[x]	[ ]	Dual use research of concern
[x]	[ ]	Plants

Methods

n/a	Involved in the study	
[x]	[ ]	ChIP-seq
[x]	[ ]	Flow cytometry
[x]	[ ]	MRI-based neuroimaging

Eukaryotic cell lines

Policy information about cell lines and Sex and Gender in Research

Cell line source(s)	NIH/3T3 fibroblast cell line isolated from a mouse NIH/Swiss embryo
Authentication	Cell lines used were not authenticated by us. These are purchased from National Center for Cell Sciences (a National Institute with cell repository in India), Pune, India, which probably keeps the cells authenticated.
Mycoplasma contamination	We do a routine testing of our cell lines for mycoplasma contamination and they tested negative for mycoplasma contamination
Commonly misidentified lines (See ICLAC register)	no misidentified cell lines used

Plants

Seed stocks	*Report on the source of all seed stocks or other plant material used. If applicable, state the seed stock centre and catalogue number. If plant specimens were collected from the field, describe the collection location, date and sampling procedures.*
Novel plant genotypes	*Describe the methods by which all novel plant genotypes were produced. This includes those generated by transgenic approaches, gene editing, chemical/radiation-based mutagenesis and hybridization. For transgenic lines, describe the transformation method, the number of independent lines analyzed and the generation upon which experiments were performed. For gene-edited lines, describe the editor used, the endogenous sequence targeted for editing, the targeting guide RNA sequence (if applicable) and how the editor was applied.*
Authentication	*Describe any authentication procedures for each seed stock used or novel genotype generated. Describe any experiments used to assess the effect of a mutation and, where applicable, how potential secondary effects (e.g. second site T-DNA insertions, mosiacism, off-target gene editing) were examined.*
